# Supplementary material for: PCAF-mediated acetylation regulates RAD51 dynamic localization on chromatin during HR repair
Source: EMBO Rep. 2025 Jul 15;26(16):4100–23. doi: 10.1038/s44319-025-00513-6 (PMC12373954; doi:10.1038/s44319-025-00513-6)
Supplement: Supplementary file 1 — Appendix [file 44319_2025_513_MOESM1_ESM.pdf]

PCAF-Mediated Acetylation Regulates RAD51 Dynamic Localization on Chromatin during HR Repair

Jiajia Hou[1a], Munan Shi[1a], Jialu Hong[1], Yuting Liu[1], Xinyi Song[1], Haipeng Rao[1], Ying Ma[1], Chunchun Huang[1], Zhigang Hu[1], Lingfeng He[1], Zhigang Guo[1]* and Feiyan Pan[1]*

[1]Jiangsu Key Laboratory for Molecular and Medical Biotechnology, College of Life Sciences, Nanjing Normal University, 1 Wen Yuan Road, Nanjing, 210023, China

[a] These authors contributed equally to this manuscript.
* To whom correspondence should be addressed:
guo@njnu.edu.cn or panfeiyan@njnu.edu.cn

Table of Contents

Appendix Figure S1 Page 2-3

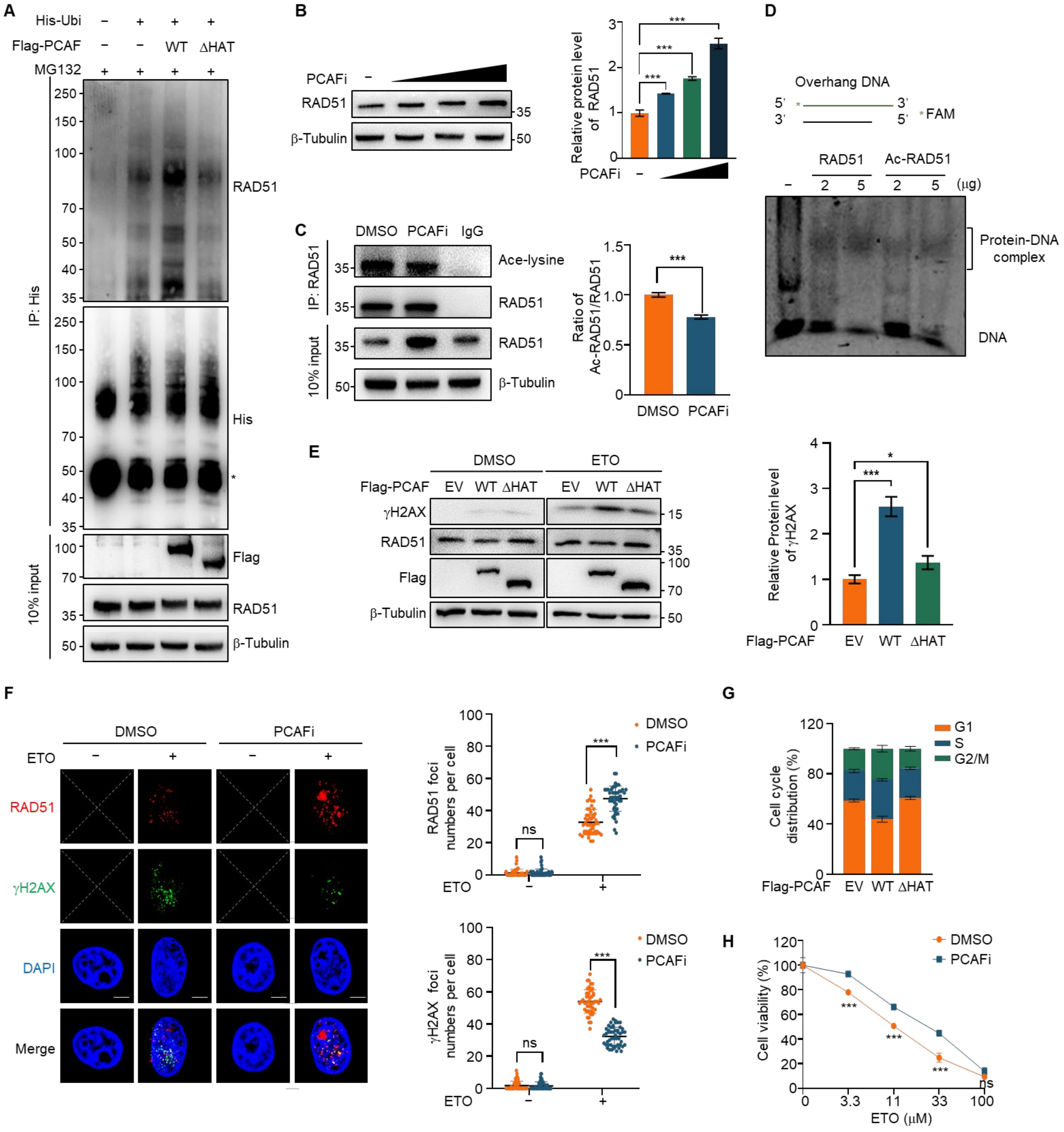

Appendix Figure S1 PCAF inhibitor promotes HR

A. Immunoblot of Ubi-RAD51 in anti-His-Ubi immunoprecipitates from HEK293T cells transfected with indicated vectors after treatment with 10 μM MG132 for 6 h. Asterisks indicate heavy chain.

B. Immunoblot (left) and quantification (right) of RAD51 from HeLa cells pretreated with DMSO and PCAFi at indicated doses (10 μM, 20 μM, 40 μM) for 24 hours. DMSO vs 10 μM (P=0.0005),

DMSO vs 20 μM (*P*<0.0001), DMSO vs 40 μM (*P*<0.0001).

C. Immunoblot (left) and quantification (right) of ac-RAD51 in anti-RAD51 immunoprecipitates from HEK293T cells treated with DMSO, PCAFi (40 μM, 24 h). *P*=0.0002.

D. RAD51 or Ac-RAD51 proteins were used to preform an EMSA ad indicated.

E. Immunoblot (left) and quantification (right) of γH2AX and RAD51 in HeLa cells transfected with the indicated vectors and treated with DMSO or ETO (20 μM, 2h). EV vs WT (*P*=0.0003), EV vs ΔHAT (*P*=0.0218).

F. Representative immunofluorescence images (left) and quantification (right) of RAD51 (red) and γH2AX (green) foci in HeLa cells pretreated with DMSO and PCAFi (40 μM, 24 h), followed by ETO exposure (20 μM, 2 h), with cell recovered for 4 h. DNA was stained by DAPI (blue). Scale bars, 10 μm, n=50. X indicated that with the chosen microscopy settings, no signal was obtained. RAD51, ETO− (*P*=0.835116), ETO+ (*P*<0.0001). γH2AX, ETO− (*P*=0.760353), ETO+ (*P*<0.0001).

G. Cell cycle analysis of HeLa cells transfected with PCAF-WT or PCAF-ΔHAT. EV vs WT, G1 (*P*=0.000677), S (*P*=0.001023), G2/M (*P*=0.011584). EV vs ΔHAT, G1 (*P*=0.108788), S (*P*=0.899971), G2/M (*P*=0.145449).

H. Cell survival assay in PCAFi-treated (40 μM, 24 h) HeLa cells in response to different doses of ETO. 3.3 μM (*P*=0.000378), 11 μM (*P*=0.000897), 33 μM (*P*=0.000981), 100 μM (*P*=0.069194).

All data are represented as mean ± S.D. of three independent experiments. *P* values are from student's t tests (B-C, E, G-H) or Mann-Whitney U-test (F). ***P*<0.01, ****P*<0.001, ns: not significant.
