# Supplementary figures and images for: PCAF-mediated acetylation regulates RAD51 dynamic localization on chromatin during HR repair (part 1 of 4)
Source: EMBO Rep. 2025 Jul 15;26(16):4100–23. doi: 10.1038/s44319-025-00513-6 (PMC12373954; doi:10.1038/s44319-025-00513-6)

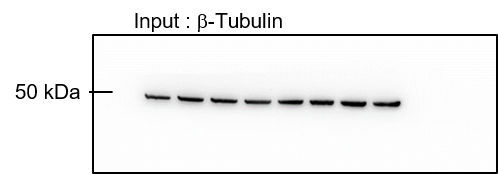

Supplement: Supplementary file 3 — Source data Fig. 1 [file 44319_2025_513_MOESM3_ESM.zip › Figure 1 Source Data/1A/Input bTubulin.tif]

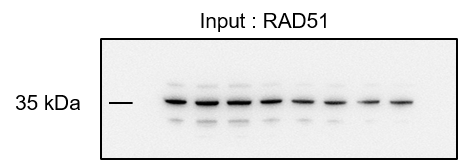

Supplement: Supplementary file 3 — Source data Fig. 1 [file 44319_2025_513_MOESM3_ESM.zip › Figure 1 Source Data/1A/Input RAD51.tif]

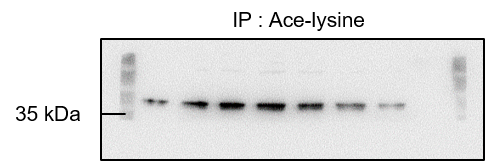

Supplement: Supplementary file 3 — Source data Fig. 1 [file 44319_2025_513_MOESM3_ESM.zip › Figure 1 Source Data/1A/IP Acelysine.tif]

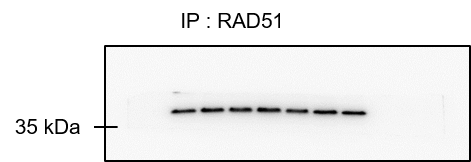

Supplement: Supplementary file 3 — Source data Fig. 1 [file 44319_2025_513_MOESM3_ESM.zip › Figure 1 Source Data/1A/IP RAD51.tif]

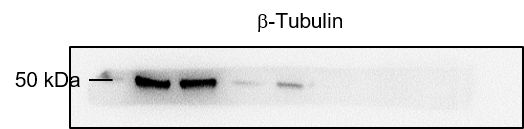

Supplement: Supplementary file 3 — Source data Fig. 1 [file 44319_2025_513_MOESM3_ESM.zip › Figure 1 Source Data/1B/bTubulin.tif]

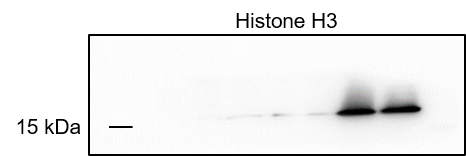

Supplement: Supplementary file 3 — Source data Fig. 1 [file 44319_2025_513_MOESM3_ESM.zip › Figure 1 Source Data/1B/Histone H3.tif]

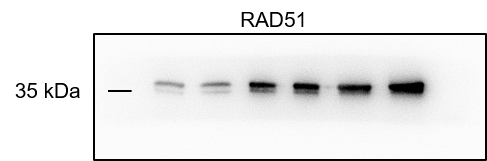

Supplement: Supplementary file 3 — Source data Fig. 1 [file 44319_2025_513_MOESM3_ESM.zip › Figure 1 Source Data/1B/RAD51.tif]

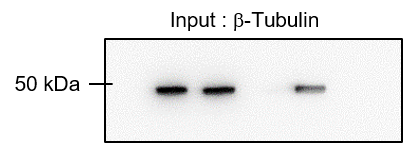

Supplement: Supplementary file 3 — Source data Fig. 1 [file 44319_2025_513_MOESM3_ESM.zip › Figure 1 Source Data/1C/Input bTubulin.tif]

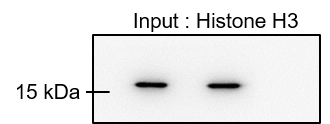

Supplement: Supplementary file 3 — Source data Fig. 1 [file 44319_2025_513_MOESM3_ESM.zip › Figure 1 Source Data/1C/Input Histone H3.tif]

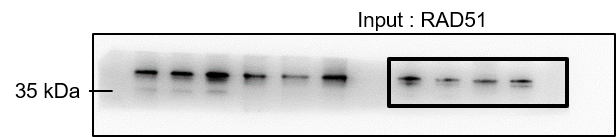

Supplement: Supplementary file 3 — Source data Fig. 1 [file 44319_2025_513_MOESM3_ESM.zip › Figure 1 Source Data/1C/Input RAD51.tif]

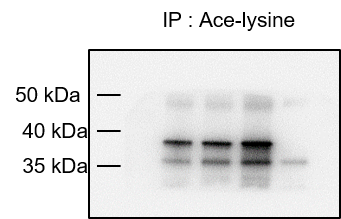

Supplement: Supplementary file 3 — Source data Fig. 1 [file 44319_2025_513_MOESM3_ESM.zip › Figure 1 Source Data/1C/IP Acelysine.tif]

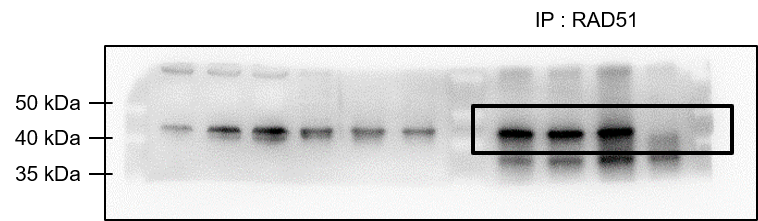

Supplement: Supplementary file 3 — Source data Fig. 1 [file 44319_2025_513_MOESM3_ESM.zip › Figure 1 Source Data/1C/IP RAD51.tif]

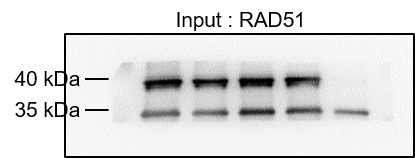

Supplement: Supplementary file 3 — Source data Fig. 1 [file 44319_2025_513_MOESM3_ESM.zip › Figure 1 Source Data/1D/Input RAD51.tif]

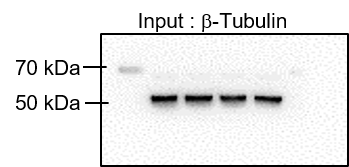

Supplement: Supplementary file 3 — Source data Fig. 1 [file 44319_2025_513_MOESM3_ESM.zip › Figure 1 Source Data/1D/Input bTubulin.tif]

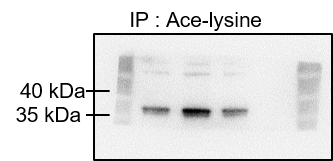

Supplement: Supplementary file 3 — Source data Fig. 1 [file 44319_2025_513_MOESM3_ESM.zip › Figure 1 Source Data/1D/IP Acelysine.tif]

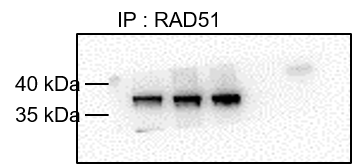

Supplement: Supplementary file 3 — Source data Fig. 1 [file 44319_2025_513_MOESM3_ESM.zip › Figure 1 Source Data/1D/IP RAD51.tif]

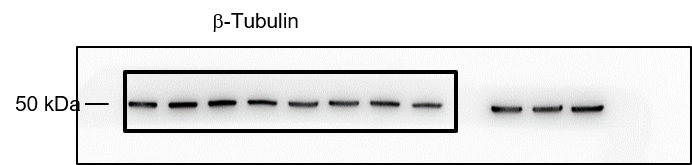

Supplement: Supplementary file 3 — Source data Fig. 1 [file 44319_2025_513_MOESM3_ESM.zip › Figure 1 Source Data/1E/bTubulin.tif]

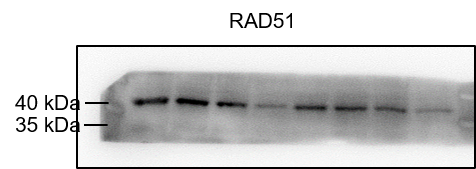

Supplement: Supplementary file 3 — Source data Fig. 1 [file 44319_2025_513_MOESM3_ESM.zip › Figure 1 Source Data/1E/RAD51.tif]

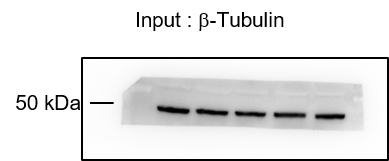

Supplement: Supplementary file 4 — Source data Fig. 2 [file 44319_2025_513_MOESM4_ESM.zip › Figure 2 Source Data/2A/Input bTubulin.tif]

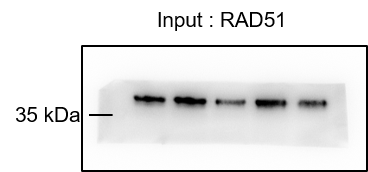

Supplement: Supplementary file 4 — Source data Fig. 2 [file 44319_2025_513_MOESM4_ESM.zip › Figure 2 Source Data/2A/Input RAD51.tif]

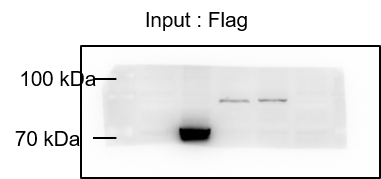

Supplement: Supplementary file 4 — Source data Fig. 2 [file 44319_2025_513_MOESM4_ESM.zip › Figure 2 Source Data/2A/Input Flag.tif]

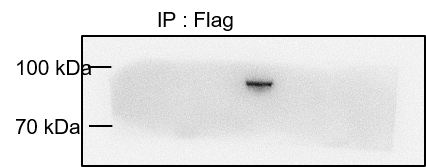

Supplement: Supplementary file 4 — Source data Fig. 2 [file 44319_2025_513_MOESM4_ESM.zip › Figure 2 Source Data/2A/IP Flag.tif]

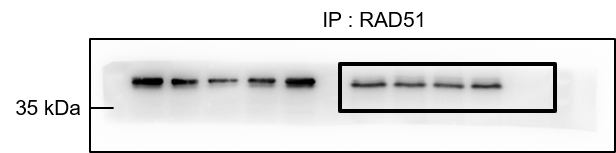

Supplement: Supplementary file 4 — Source data Fig. 2 [file 44319_2025_513_MOESM4_ESM.zip › Figure 2 Source Data/2A/IP RAD51.tif]

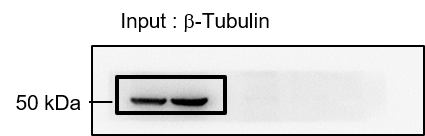

Supplement: Supplementary file 4 — Source data Fig. 2 [file 44319_2025_513_MOESM4_ESM.zip › Figure 2 Source Data/2B/Input bTubulin.tif]

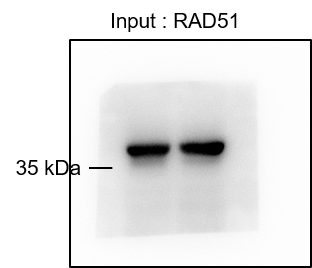

Supplement: Supplementary file 4 — Source data Fig. 2 [file 44319_2025_513_MOESM4_ESM.zip › Figure 2 Source Data/2B/input RAD51.tif]

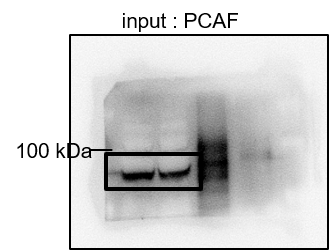

Supplement: Supplementary file 4 — Source data Fig. 2 [file 44319_2025_513_MOESM4_ESM.zip › Figure 2 Source Data/2B/input PCAF.tif]

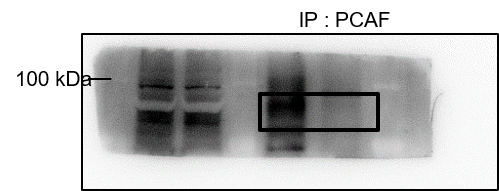

Supplement: Supplementary file 4 — Source data Fig. 2 [file 44319_2025_513_MOESM4_ESM.zip › Figure 2 Source Data/2B/IP PCAF.tif]

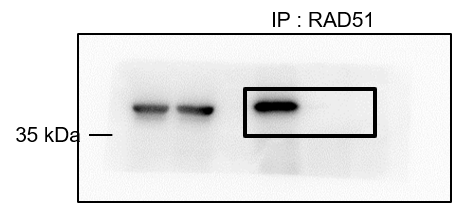

Supplement: Supplementary file 4 — Source data Fig. 2 [file 44319_2025_513_MOESM4_ESM.zip › Figure 2 Source Data/2B/IP RAD51.tif]

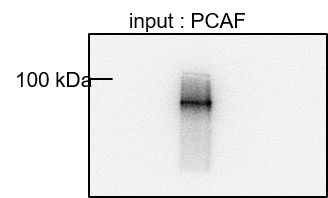

Supplement: Supplementary file 4 — Source data Fig. 2 [file 44319_2025_513_MOESM4_ESM.zip › Figure 2 Source Data/2C/input PCAF.tif]

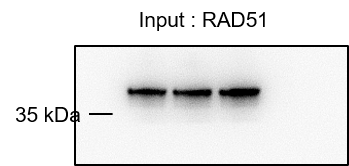

Supplement: Supplementary file 4 — Source data Fig. 2 [file 44319_2025_513_MOESM4_ESM.zip › Figure 2 Source Data/2C/input RAD51.tif]

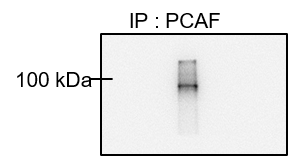

Supplement: Supplementary file 4 — Source data Fig. 2 [file 44319_2025_513_MOESM4_ESM.zip › Figure 2 Source Data/2C/IP PCAF.tif]

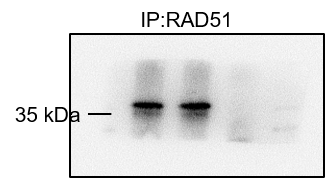

Supplement: Supplementary file 4 — Source data Fig. 2 [file 44319_2025_513_MOESM4_ESM.zip › Figure 2 Source Data/2C/IP RAD51.tif]

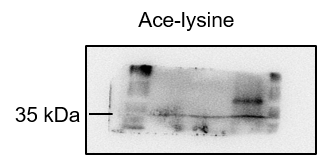

Supplement: Supplementary file 4 — Source data Fig. 2 [file 44319_2025_513_MOESM4_ESM.zip › Figure 2 Source Data/2D/Ace-lysine.tif]

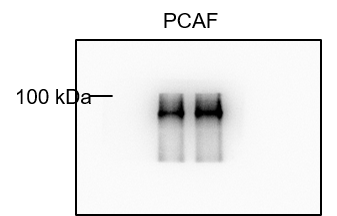

Supplement: Supplementary file 4 — Source data Fig. 2 [file 44319_2025_513_MOESM4_ESM.zip › Figure 2 Source Data/2D/PCAF.tif]

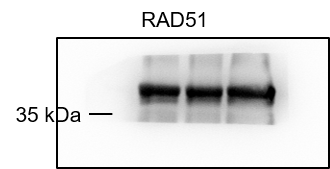

Supplement: Supplementary file 4 — Source data Fig. 2 [file 44319_2025_513_MOESM4_ESM.zip › Figure 2 Source Data/2D/RAD51.tif]

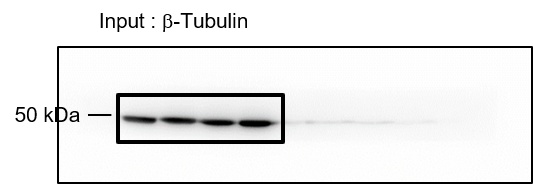

Supplement: Supplementary file 4 — Source data Fig. 2 [file 44319_2025_513_MOESM4_ESM.zip › Figure 2 Source Data/2E/Input bTubulin.tif]

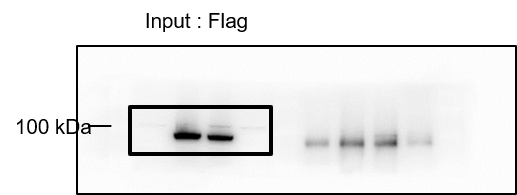

Supplement: Supplementary file 4 — Source data Fig. 2 [file 44319_2025_513_MOESM4_ESM.zip › Figure 2 Source Data/2E/Input Flag.tif]

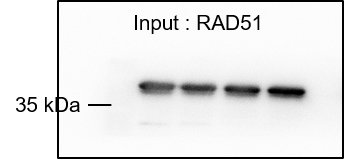

Supplement: Supplementary file 4 — Source data Fig. 2 [file 44319_2025_513_MOESM4_ESM.zip › Figure 2 Source Data/2E/Input RAD51.tif]

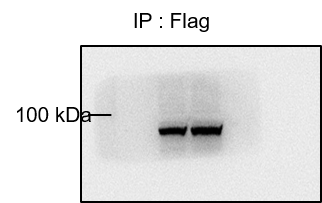

Supplement: Supplementary file 4 — Source data Fig. 2 [file 44319_2025_513_MOESM4_ESM.zip › Figure 2 Source Data/2E/IP Flag.tif]

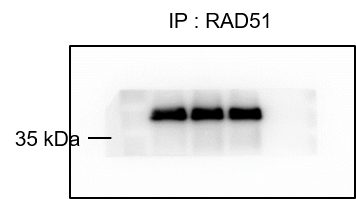

Supplement: Supplementary file 4 — Source data Fig. 2 [file 44319_2025_513_MOESM4_ESM.zip › Figure 2 Source Data/2E/IP RAD51.tif]

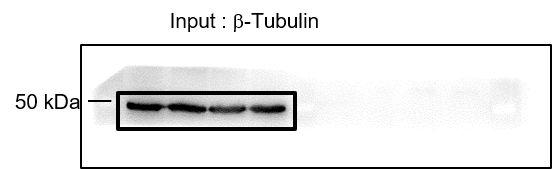

Supplement: Supplementary file 4 — Source data Fig. 2 [file 44319_2025_513_MOESM4_ESM.zip › Figure 2 Source Data/2F/Input bTubulin.tif]

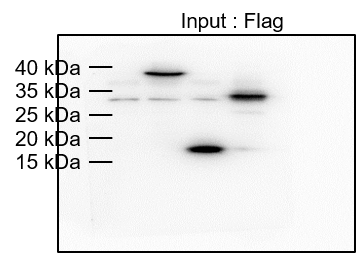

Supplement: Supplementary file 4 — Source data Fig. 2 [file 44319_2025_513_MOESM4_ESM.zip › Figure 2 Source Data/2F/Input Flag.tif]

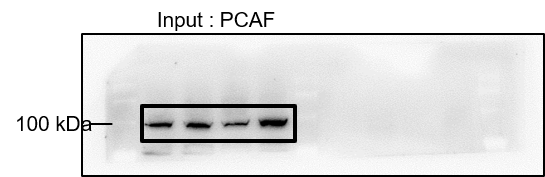

Supplement: Supplementary file 4 — Source data Fig. 2 [file 44319_2025_513_MOESM4_ESM.zip › Figure 2 Source Data/2F/Input PCAF.tif]

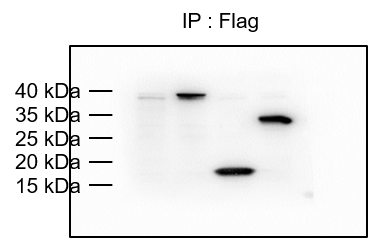

Supplement: Supplementary file 4 — Source data Fig. 2 [file 44319_2025_513_MOESM4_ESM.zip › Figure 2 Source Data/2F/IP Flag.tif]

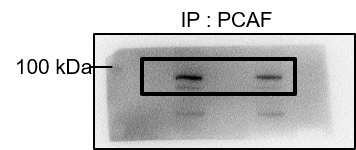

Supplement: Supplementary file 4 — Source data Fig. 2 [file 44319_2025_513_MOESM4_ESM.zip › Figure 2 Source Data/2F/IP PCAF.tif]

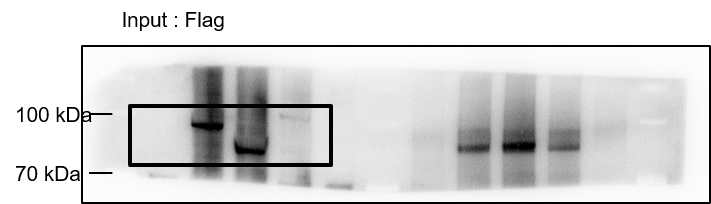

Supplement: Supplementary file 4 — Source data Fig. 2 [file 44319_2025_513_MOESM4_ESM.zip › Figure 2 Source Data/2G/Input Flag.tif]

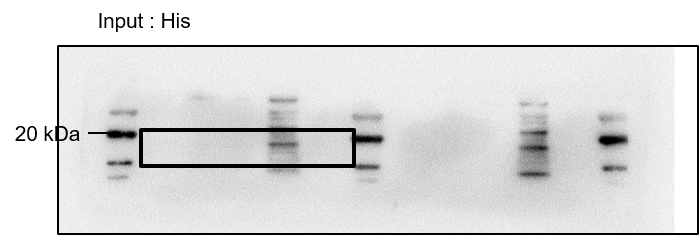

Supplement: Supplementary file 4 — Source data Fig. 2 [file 44319_2025_513_MOESM4_ESM.zip › Figure 2 Source Data/2G/Input His.tif]

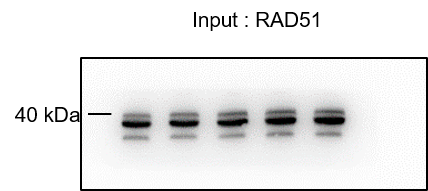

Supplement: Supplementary file 4 — Source data Fig. 2 [file 44319_2025_513_MOESM4_ESM.zip › Figure 2 Source Data/2G/Input RAD51.tif]

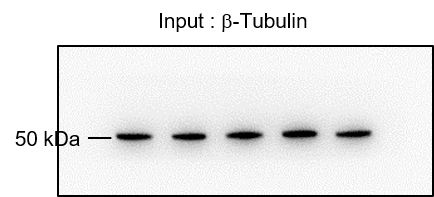

Supplement: Supplementary file 4 — Source data Fig. 2 [file 44319_2025_513_MOESM4_ESM.zip › Figure 2 Source Data/2G/Input bTubulin.tif]

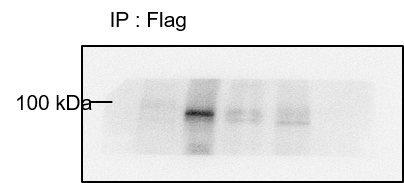

Supplement: Supplementary file 4 — Source data Fig. 2 [file 44319_2025_513_MOESM4_ESM.zip › Figure 2 Source Data/2G/IP Flag.tif]

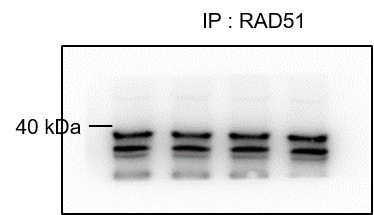

Supplement: Supplementary file 4 — Source data Fig. 2 [file 44319_2025_513_MOESM4_ESM.zip › Figure 2 Source Data/2G/IP RAD51.tif]

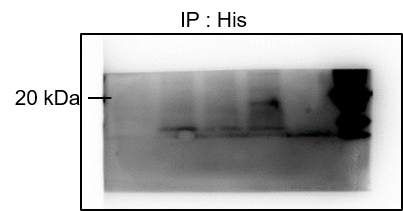

Supplement: Supplementary file 4 — Source data Fig. 2 [file 44319_2025_513_MOESM4_ESM.zip › Figure 2 Source Data/2G/IP His.tif]

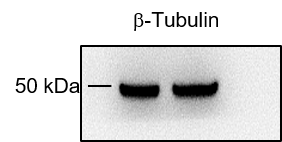

Supplement: Supplementary file 5 — Source data Fig. 3 [file 44319_2025_513_MOESM5_ESM.zip › Figure 3 Source Data/3A/bTubulin.tif]

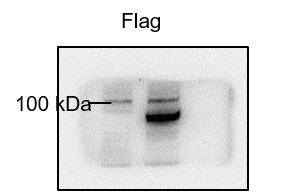

Supplement: Supplementary file 5 — Source data Fig. 3 [file 44319_2025_513_MOESM5_ESM.zip › Figure 3 Source Data/3A/Flag.tif]

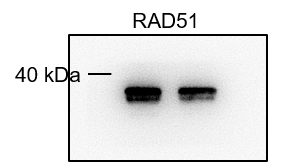

Supplement: Supplementary file 5 — Source data Fig. 3 [file 44319_2025_513_MOESM5_ESM.zip › Figure 3 Source Data/3A/RAD51.tif]

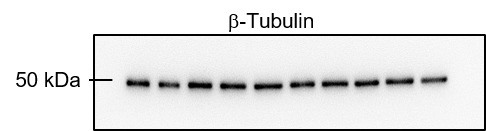

Supplement: Supplementary file 5 — Source data Fig. 3 [file 44319_2025_513_MOESM5_ESM.zip › Figure 3 Source Data/3B/bTubulin.tif]

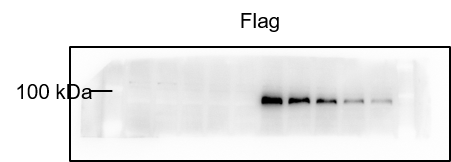

Supplement: Supplementary file 5 — Source data Fig. 3 [file 44319_2025_513_MOESM5_ESM.zip › Figure 3 Source Data/3B/Flag.tif]

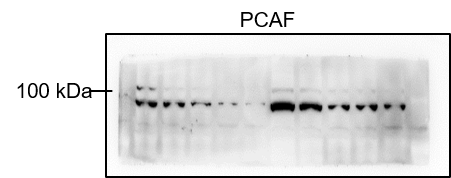

Supplement: Supplementary file 5 — Source data Fig. 3 [file 44319_2025_513_MOESM5_ESM.zip › Figure 3 Source Data/3B/PCAF.tif]

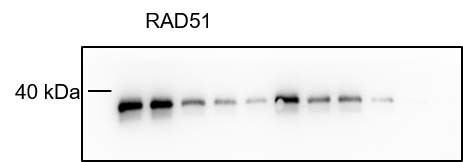

Supplement: Supplementary file 5 — Source data Fig. 3 [file 44319_2025_513_MOESM5_ESM.zip › Figure 3 Source Data/3B/RAD51.tif]

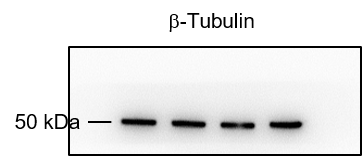

Supplement: Supplementary file 5 — Source data Fig. 3 [file 44319_2025_513_MOESM5_ESM.zip › Figure 3 Source Data/3C/bTubulin.tif]

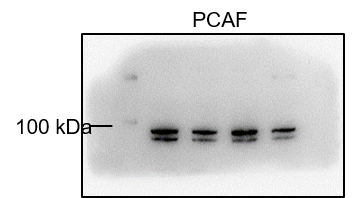

Supplement: Supplementary file 5 — Source data Fig. 3 [file 44319_2025_513_MOESM5_ESM.zip › Figure 3 Source Data/3C/PCAF.tif]

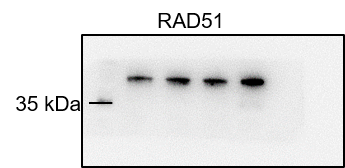

Supplement: Supplementary file 5 — Source data Fig. 3 [file 44319_2025_513_MOESM5_ESM.zip › Figure 3 Source Data/3C/RAD51.tif]

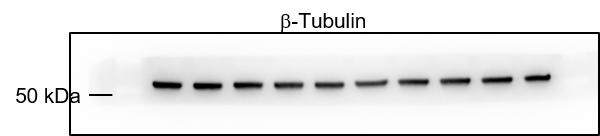

Supplement: Supplementary file 5 — Source data Fig. 3 [file 44319_2025_513_MOESM5_ESM.zip › Figure 3 Source Data/3D/bTubulin.tif]

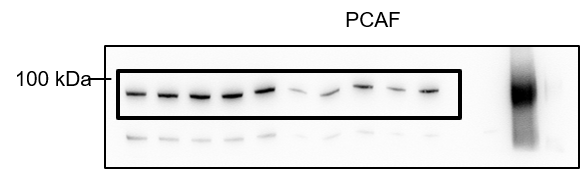

Supplement: Supplementary file 5 — Source data Fig. 3 [file 44319_2025_513_MOESM5_ESM.zip › Figure 3 Source Data/3D/PCAF.tif]

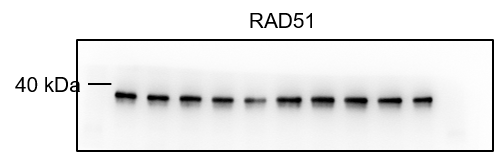

Supplement: Supplementary file 5 — Source data Fig. 3 [file 44319_2025_513_MOESM5_ESM.zip › Figure 3 Source Data/3D/RAD51.tif]

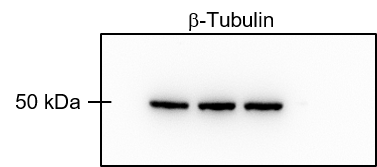

Supplement: Supplementary file 5 — Source data Fig. 3 [file 44319_2025_513_MOESM5_ESM.zip › Figure 3 Source Data/3E/bTubulin.tif]

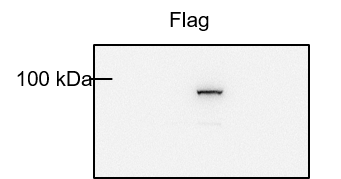

Supplement: Supplementary file 5 — Source data Fig. 3 [file 44319_2025_513_MOESM5_ESM.zip › Figure 3 Source Data/3E/Flag.tif]

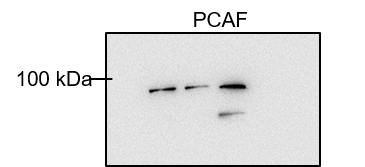

Supplement: Supplementary file 5 — Source data Fig. 3 [file 44319_2025_513_MOESM5_ESM.zip › Figure 3 Source Data/3E/PCAF.tif]

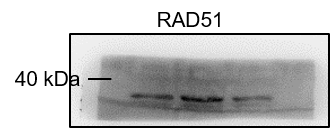

Supplement: Supplementary file 5 — Source data Fig. 3 [file 44319_2025_513_MOESM5_ESM.zip › Figure 3 Source Data/3E/RAD51.tif]

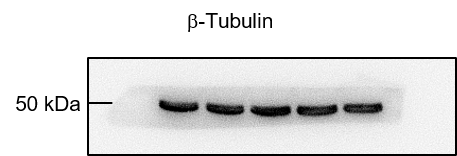

Supplement: Supplementary file 5 — Source data Fig. 3 [file 44319_2025_513_MOESM5_ESM.zip › Figure 3 Source Data/3F/bTubulin.tif]

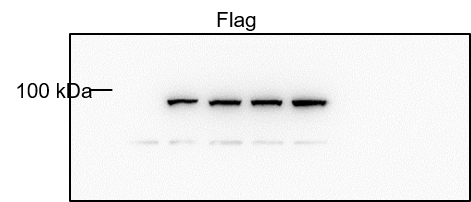

Supplement: Supplementary file 5 — Source data Fig. 3 [file 44319_2025_513_MOESM5_ESM.zip › Figure 3 Source Data/3F/Flag.tif]

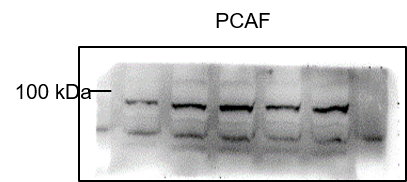

Supplement: Supplementary file 5 — Source data Fig. 3 [file 44319_2025_513_MOESM5_ESM.zip › Figure 3 Source Data/3F/PCAF.tif]

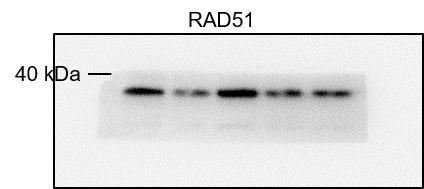

Supplement: Supplementary file 5 — Source data Fig. 3 [file 44319_2025_513_MOESM5_ESM.zip › Figure 3 Source Data/3F/RAD51.tif]

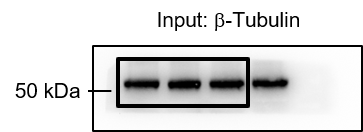

Supplement: Supplementary file 5 — Source data Fig. 3 [file 44319_2025_513_MOESM5_ESM.zip › Figure 3 Source Data/3G/Input bTubulin.tif]

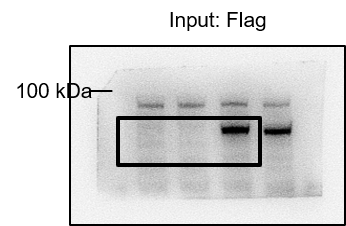

Supplement: Supplementary file 5 — Source data Fig. 3 [file 44319_2025_513_MOESM5_ESM.zip › Figure 3 Source Data/3G/Input Flag.tif]

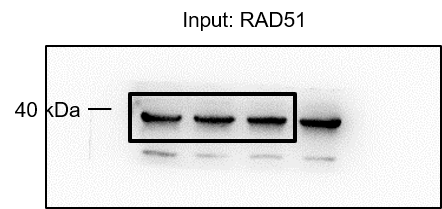

Supplement: Supplementary file 5 — Source data Fig. 3 [file 44319_2025_513_MOESM5_ESM.zip › Figure 3 Source Data/3G/Input RAD51.tif]

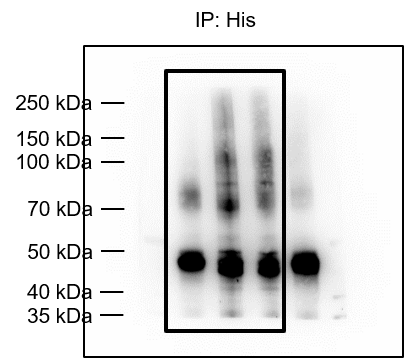

Supplement: Supplementary file 5 — Source data Fig. 3 [file 44319_2025_513_MOESM5_ESM.zip › Figure 3 Source Data/3G/IP His.tif]

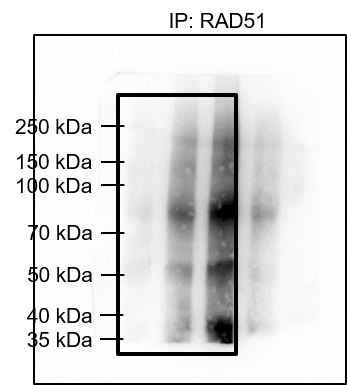

Supplement: Supplementary file 5 — Source data Fig. 3 [file 44319_2025_513_MOESM5_ESM.zip › Figure 3 Source Data/3G/IP RAD51.tif]

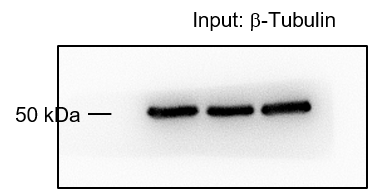

Supplement: Supplementary file 5 — Source data Fig. 3 [file 44319_2025_513_MOESM5_ESM.zip › Figure 3 Source Data/3H/Input bTubulin.tif]

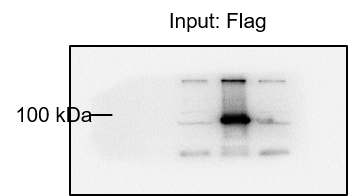

Supplement: Supplementary file 5 — Source data Fig. 3 [file 44319_2025_513_MOESM5_ESM.zip › Figure 3 Source Data/3H/Input Flag.tif]

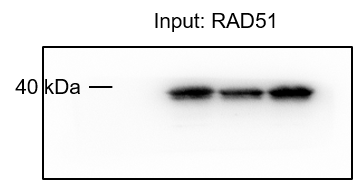

Supplement: Supplementary file 5 — Source data Fig. 3 [file 44319_2025_513_MOESM5_ESM.zip › Figure 3 Source Data/3H/Input RAD51.tif]

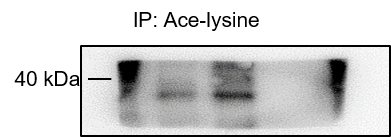

Supplement: Supplementary file 5 — Source data Fig. 3 [file 44319_2025_513_MOESM5_ESM.zip › Figure 3 Source Data/3H/IP Acelysine.tif]

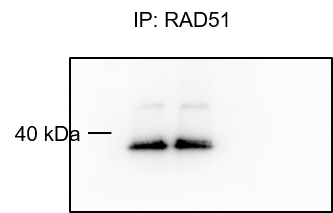

Supplement: Supplementary file 5 — Source data Fig. 3 [file 44319_2025_513_MOESM5_ESM.zip › Figure 3 Source Data/3H/IP RAD51.tif]

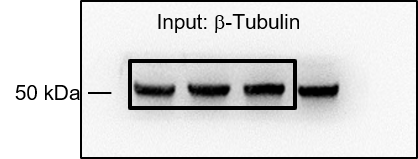

Supplement: Supplementary file 5 — Source data Fig. 3 [file 44319_2025_513_MOESM5_ESM.zip › Figure 3 Source Data/3J/Input bTubulin.tif]

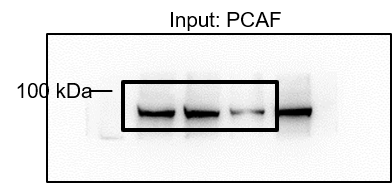

Supplement: Supplementary file 5 — Source data Fig. 3 [file 44319_2025_513_MOESM5_ESM.zip › Figure 3 Source Data/3J/Input PCAF.tif]

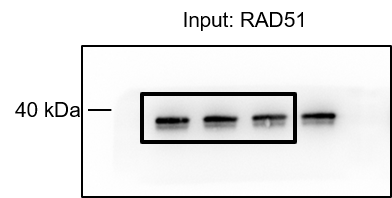

Supplement: Supplementary file 5 — Source data Fig. 3 [file 44319_2025_513_MOESM5_ESM.zip › Figure 3 Source Data/3J/Input RAD51.tif]

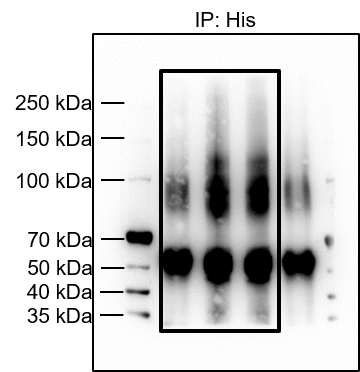

Supplement: Supplementary file 5 — Source data Fig. 3 [file 44319_2025_513_MOESM5_ESM.zip › Figure 3 Source Data/3J/IP His.tif]

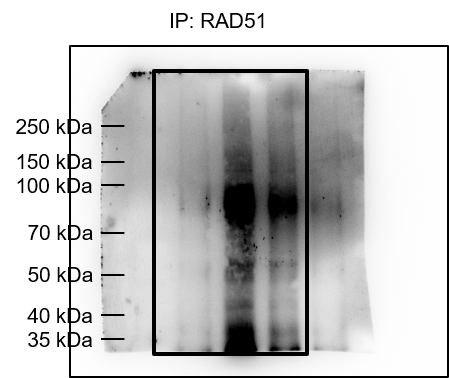

Supplement: Supplementary file 5 — Source data Fig. 3 [file 44319_2025_513_MOESM5_ESM.zip › Figure 3 Source Data/3J/IP RAD51.tif]

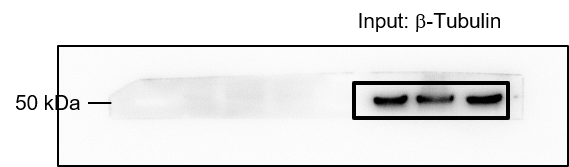

Supplement: Supplementary file 5 — Source data Fig. 3 [file 44319_2025_513_MOESM5_ESM.zip › Figure 3 Source Data/3K/Input bTubulin.tif]

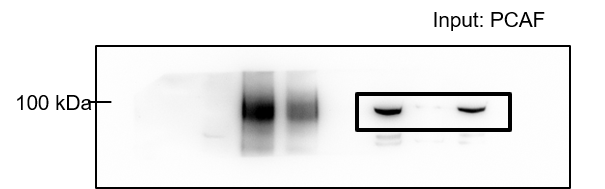

Supplement: Supplementary file 5 — Source data Fig. 3 [file 44319_2025_513_MOESM5_ESM.zip › Figure 3 Source Data/3K/Input PCAF.tif]

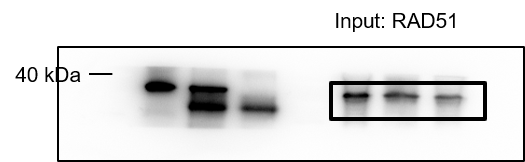

Supplement: Supplementary file 5 — Source data Fig. 3 [file 44319_2025_513_MOESM5_ESM.zip › Figure 3 Source Data/3K/Input RAD51.tif]

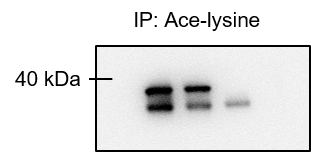

Supplement: Supplementary file 5 — Source data Fig. 3 [file 44319_2025_513_MOESM5_ESM.zip › Figure 3 Source Data/3K/IP Acelysine.tif]

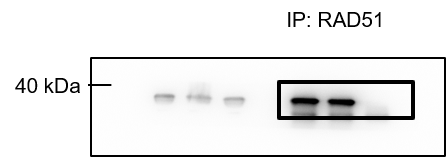

Supplement: Supplementary file 5 — Source data Fig. 3 [file 44319_2025_513_MOESM5_ESM.zip › Figure 3 Source Data/3K/IP RAD51.tif]

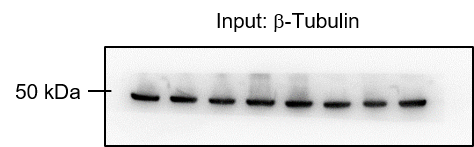

Supplement: Supplementary file 6 — Source data Fig. 4 [file 44319_2025_513_MOESM6_ESM.zip › Figure 4 Source Data/4A/Input bTubulin.tif]

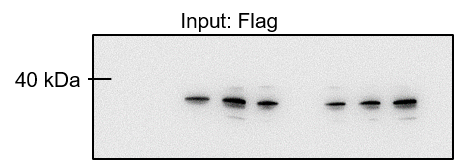

Supplement: Supplementary file 6 — Source data Fig. 4 [file 44319_2025_513_MOESM6_ESM.zip › Figure 4 Source Data/4A/Input Flag.tif]

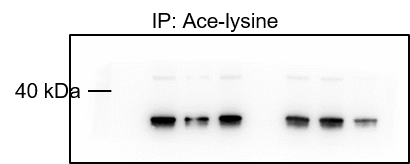

Supplement: Supplementary file 6 — Source data Fig. 4 [file 44319_2025_513_MOESM6_ESM.zip › Figure 4 Source Data/4A/IP Acelysine.tif]

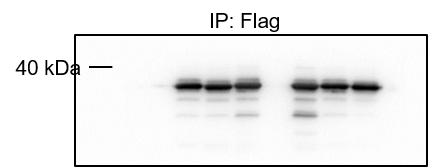

Supplement: Supplementary file 6 — Source data Fig. 4 [file 44319_2025_513_MOESM6_ESM.zip › Figure 4 Source Data/4A/IP Flag.tif]

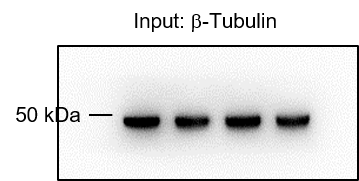

Supplement: Supplementary file 6 — Source data Fig. 4 [file 44319_2025_513_MOESM6_ESM.zip › Figure 4 Source Data/4B/Input bTubulin.tif]

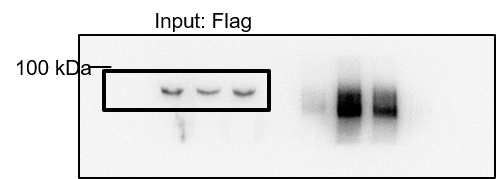

Supplement: Supplementary file 6 — Source data Fig. 4 [file 44319_2025_513_MOESM6_ESM.zip › Figure 4 Source Data/4B/Input Flag.tif]

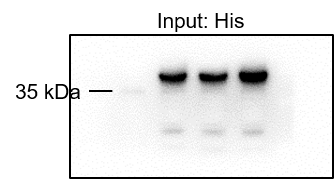

Supplement: Supplementary file 6 — Source data Fig. 4 [file 44319_2025_513_MOESM6_ESM.zip › Figure 4 Source Data/4B/Input His.tif]
