# Supplementary figures and images for: PCAF-mediated acetylation regulates RAD51 dynamic localization on chromatin during HR repair (part 2 of 4)
Source: EMBO Rep. 2025 Jul 15;26(16):4100–23. doi: 10.1038/s44319-025-00513-6 (PMC12373954; doi:10.1038/s44319-025-00513-6)

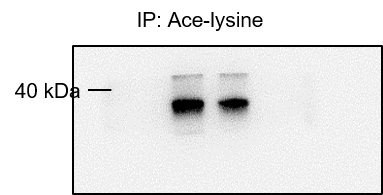

Supplement: Supplementary file 6 — Source data Fig. 4 [file 44319_2025_513_MOESM6_ESM.zip › Figure 4 Source Data/4B/IP Acelysine.tif]

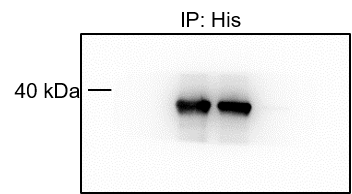

Supplement: Supplementary file 6 — Source data Fig. 4 [file 44319_2025_513_MOESM6_ESM.zip › Figure 4 Source Data/4B/IP His.tif]

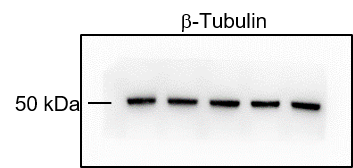

Supplement: Supplementary file 6 — Source data Fig. 4 [file 44319_2025_513_MOESM6_ESM.zip › Figure 4 Source Data/4C/b-Tubulin.tif]

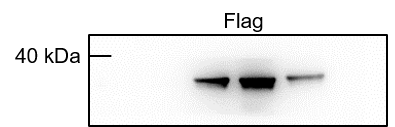

Supplement: Supplementary file 6 — Source data Fig. 4 [file 44319_2025_513_MOESM6_ESM.zip › Figure 4 Source Data/4C/Flag.tif]

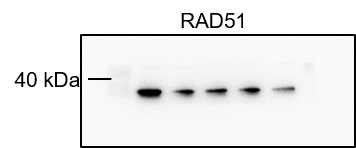

Supplement: Supplementary file 6 — Source data Fig. 4 [file 44319_2025_513_MOESM6_ESM.zip › Figure 4 Source Data/4C/RAD51.tif]

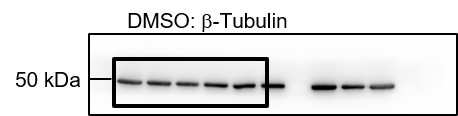

Supplement: Supplementary file 6 — Source data Fig. 4 [file 44319_2025_513_MOESM6_ESM.zip › Figure 4 Source Data/4D/DMSO bTubulin.tif]

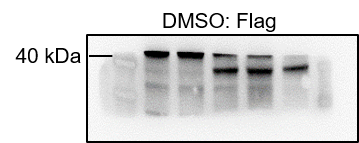

Supplement: Supplementary file 6 — Source data Fig. 4 [file 44319_2025_513_MOESM6_ESM.zip › Figure 4 Source Data/4D/DMSO Flag.tif]

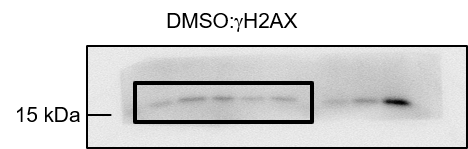

Supplement: Supplementary file 6 — Source data Fig. 4 [file 44319_2025_513_MOESM6_ESM.zip › Figure 4 Source Data/4D/DMSO gH2AX.tif]

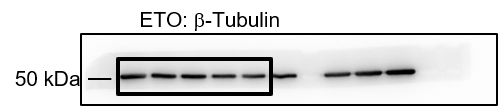

Supplement: Supplementary file 6 — Source data Fig. 4 [file 44319_2025_513_MOESM6_ESM.zip › Figure 4 Source Data/4D/ETO bTubulin.tif]

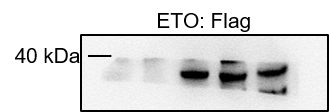

Supplement: Supplementary file 6 — Source data Fig. 4 [file 44319_2025_513_MOESM6_ESM.zip › Figure 4 Source Data/4D/ETO Flag.tif]

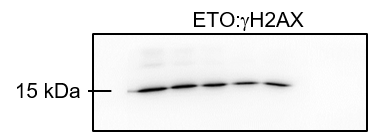

Supplement: Supplementary file 6 — Source data Fig. 4 [file 44319_2025_513_MOESM6_ESM.zip › Figure 4 Source Data/4D/ETO gH2AX.tif]

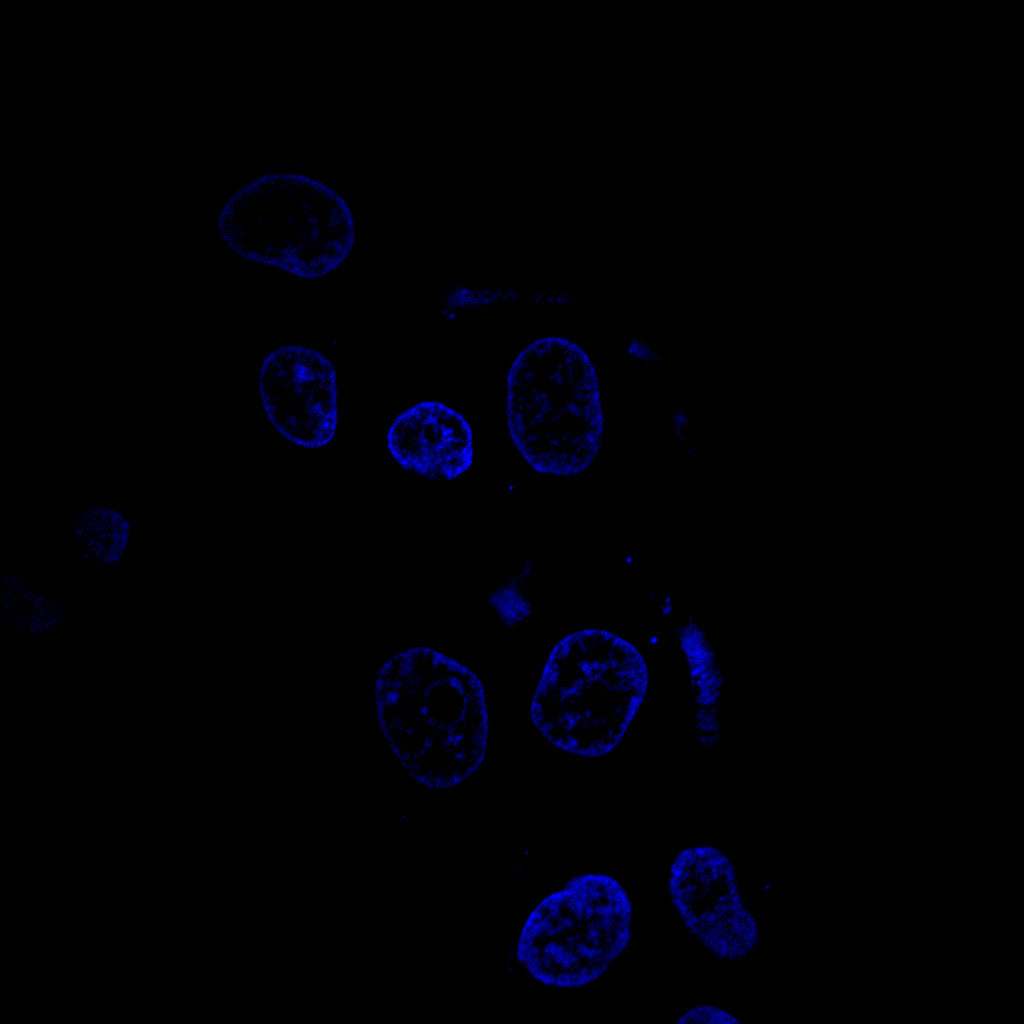

Supplement: Supplementary file 6 — Source data Fig. 4 [file 44319_2025_513_MOESM6_ESM.zip › Figure 4 Source Data/4E/EV ETO+/DAPI.tif]

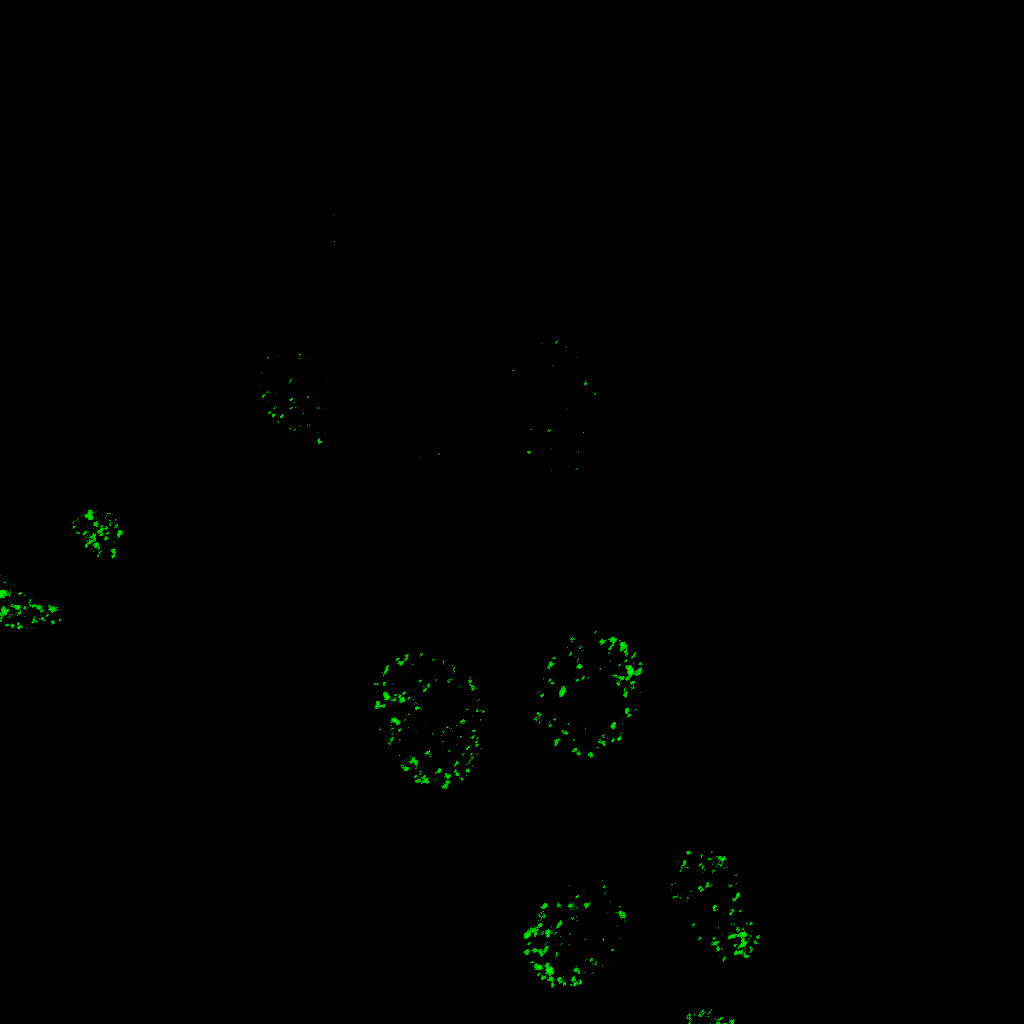

Supplement: Supplementary file 6 — Source data Fig. 4 [file 44319_2025_513_MOESM6_ESM.zip › Figure 4 Source Data/4E/EV ETO+/GH2AX.tif]

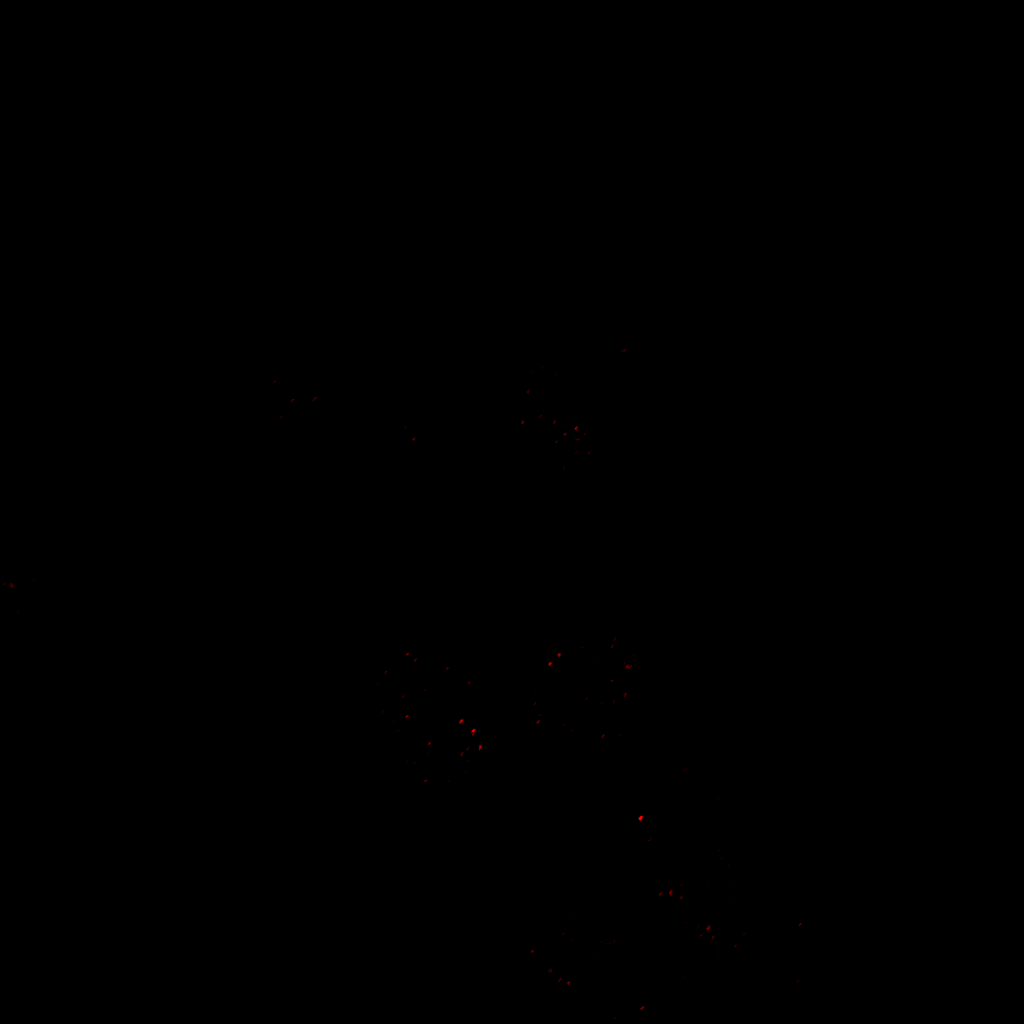

Supplement: Supplementary file 6 — Source data Fig. 4 [file 44319_2025_513_MOESM6_ESM.zip › Figure 4 Source Data/4E/EV ETO+/RAD51.tif]

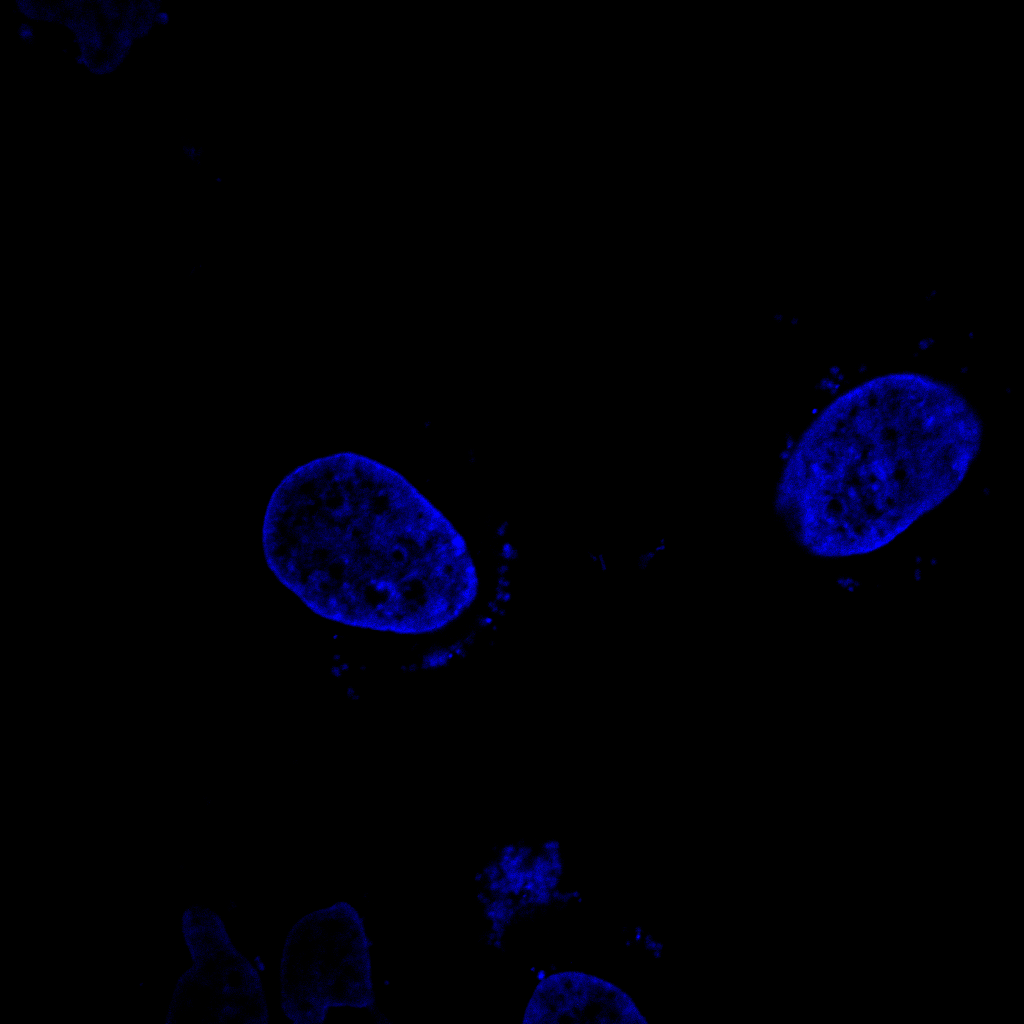

Supplement: Supplementary file 6 — Source data Fig. 4 [file 44319_2025_513_MOESM6_ESM.zip › Figure 4 Source Data/4E/EV ETO-/DAPI.tif]

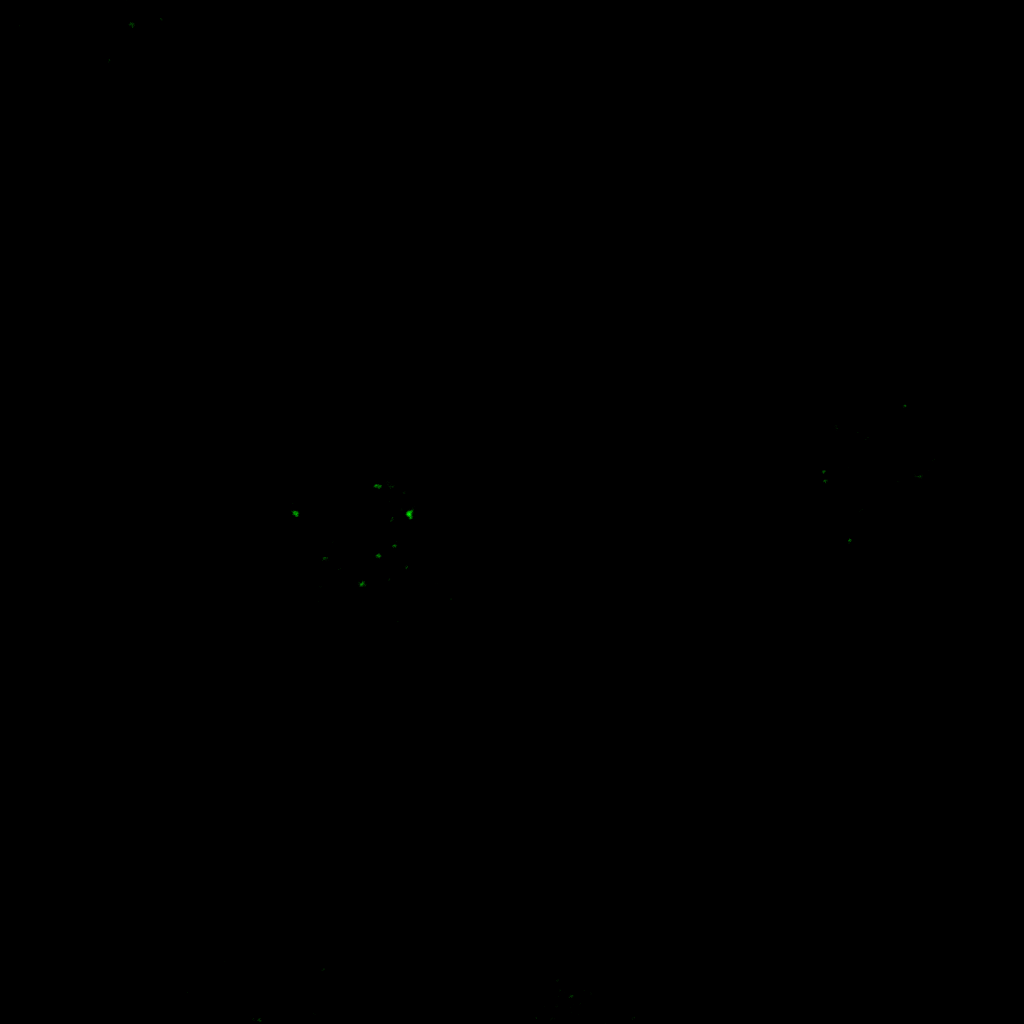

Supplement: Supplementary file 6 — Source data Fig. 4 [file 44319_2025_513_MOESM6_ESM.zip › Figure 4 Source Data/4E/EV ETO-/GH2AX.tif]

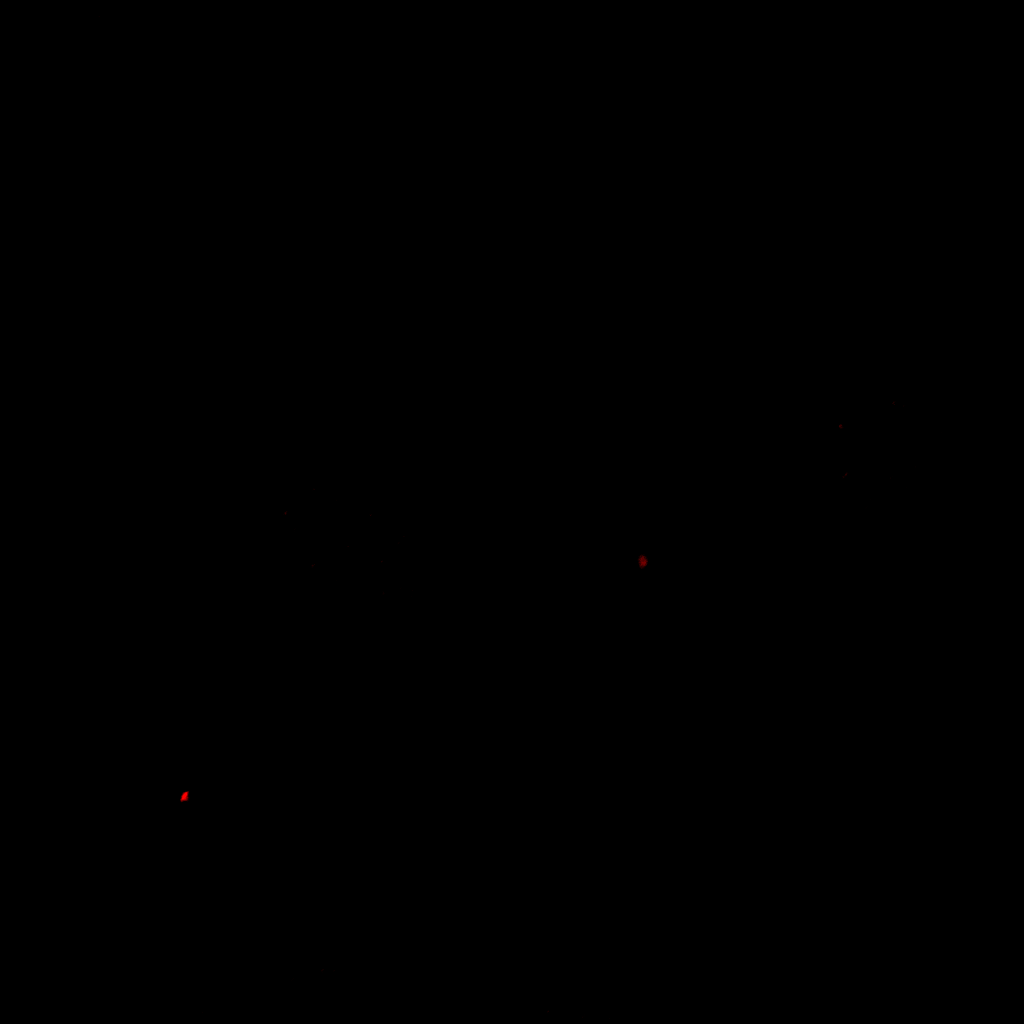

Supplement: Supplementary file 6 — Source data Fig. 4 [file 44319_2025_513_MOESM6_ESM.zip › Figure 4 Source Data/4E/EV ETO-/RAD51.tif]

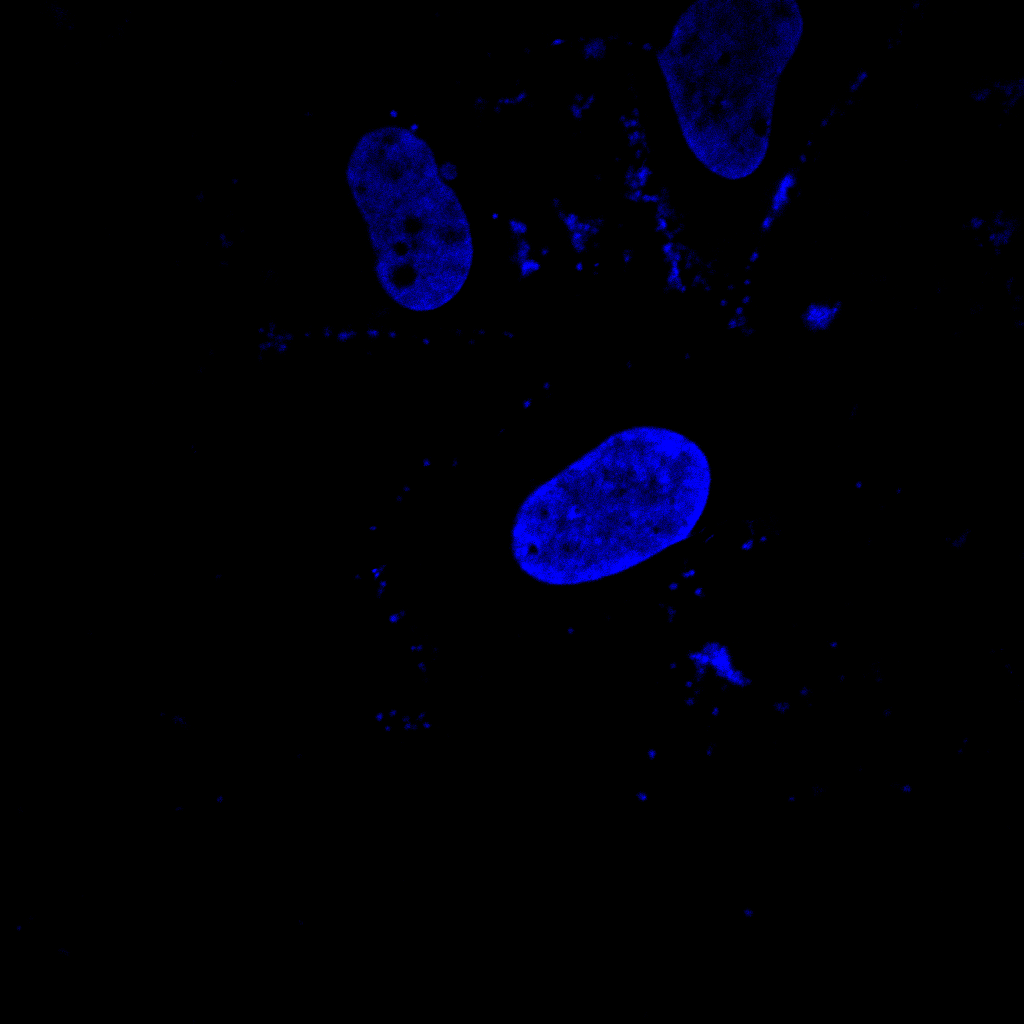

Supplement: Supplementary file 6 — Source data Fig. 4 [file 44319_2025_513_MOESM6_ESM.zip › Figure 4 Source Data/4E/K40Q ETO+/DAPI.tif]

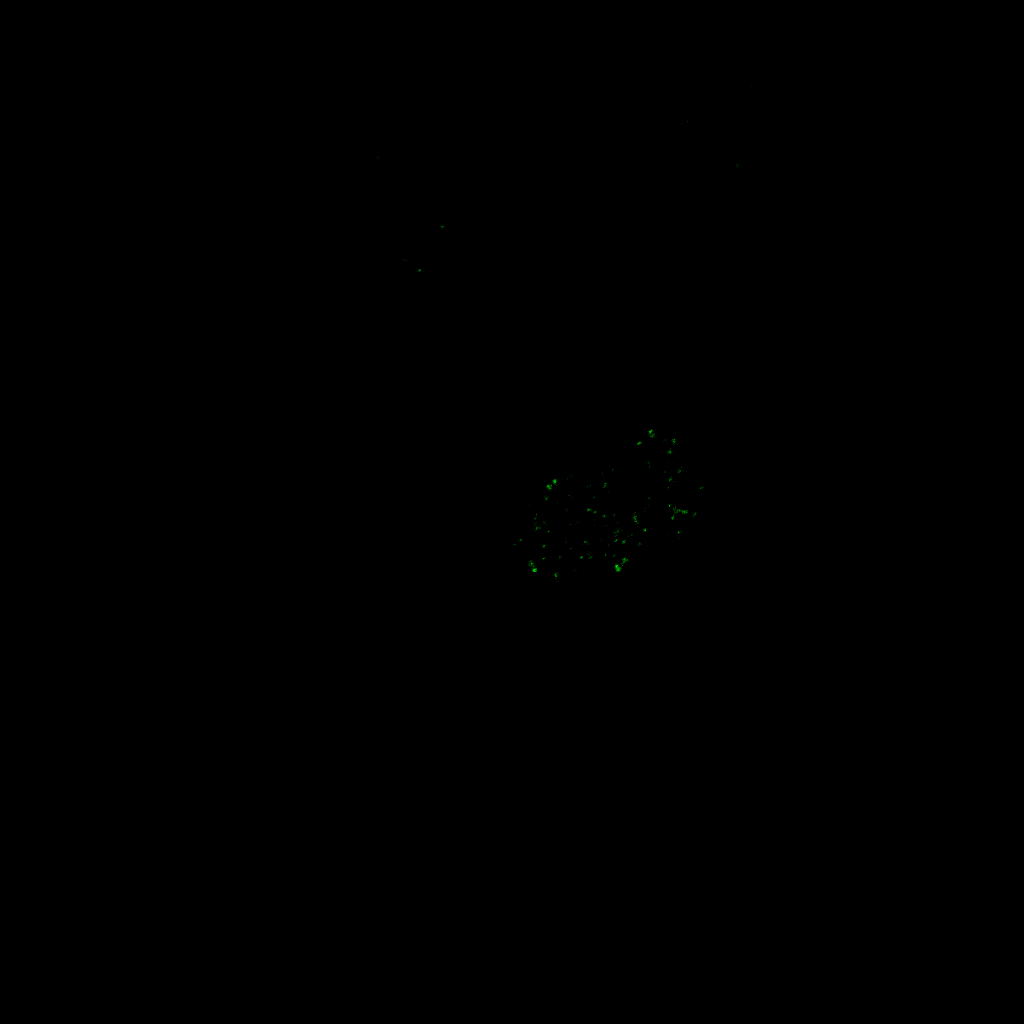

Supplement: Supplementary file 6 — Source data Fig. 4 [file 44319_2025_513_MOESM6_ESM.zip › Figure 4 Source Data/4E/K40Q ETO+/GHA2X.tif]

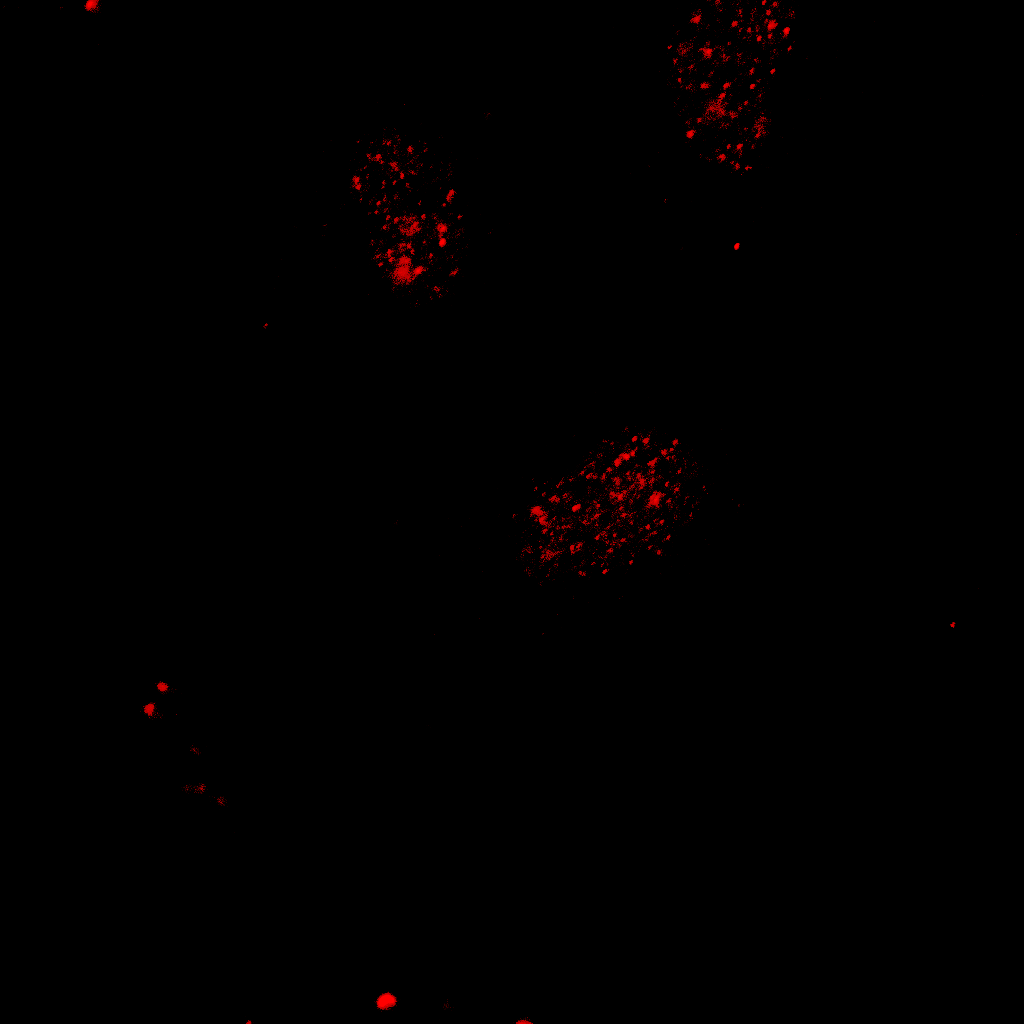

Supplement: Supplementary file 6 — Source data Fig. 4 [file 44319_2025_513_MOESM6_ESM.zip › Figure 4 Source Data/4E/K40Q ETO+/RAD51.tif]

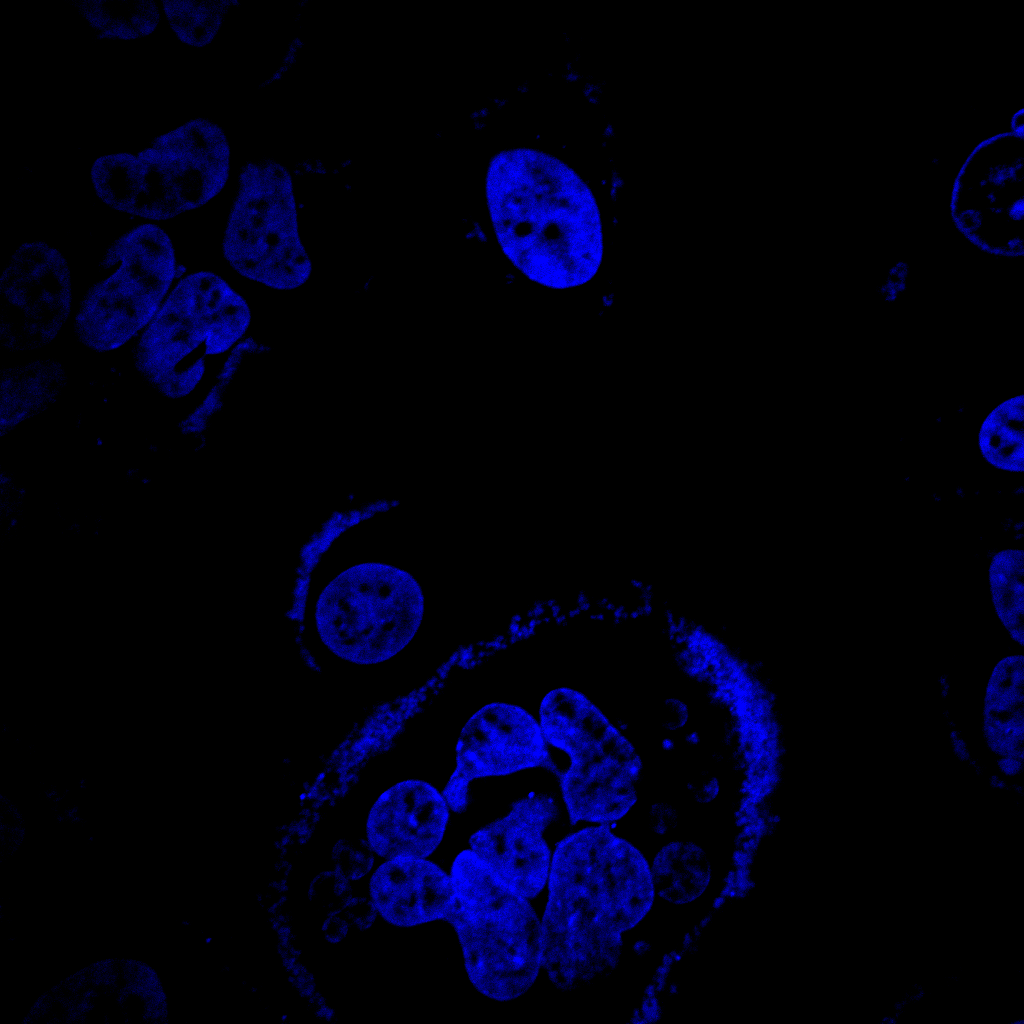

Supplement: Supplementary file 6 — Source data Fig. 4 [file 44319_2025_513_MOESM6_ESM.zip › Figure 4 Source Data/4E/K40Q ETO-/DAPI.tif]

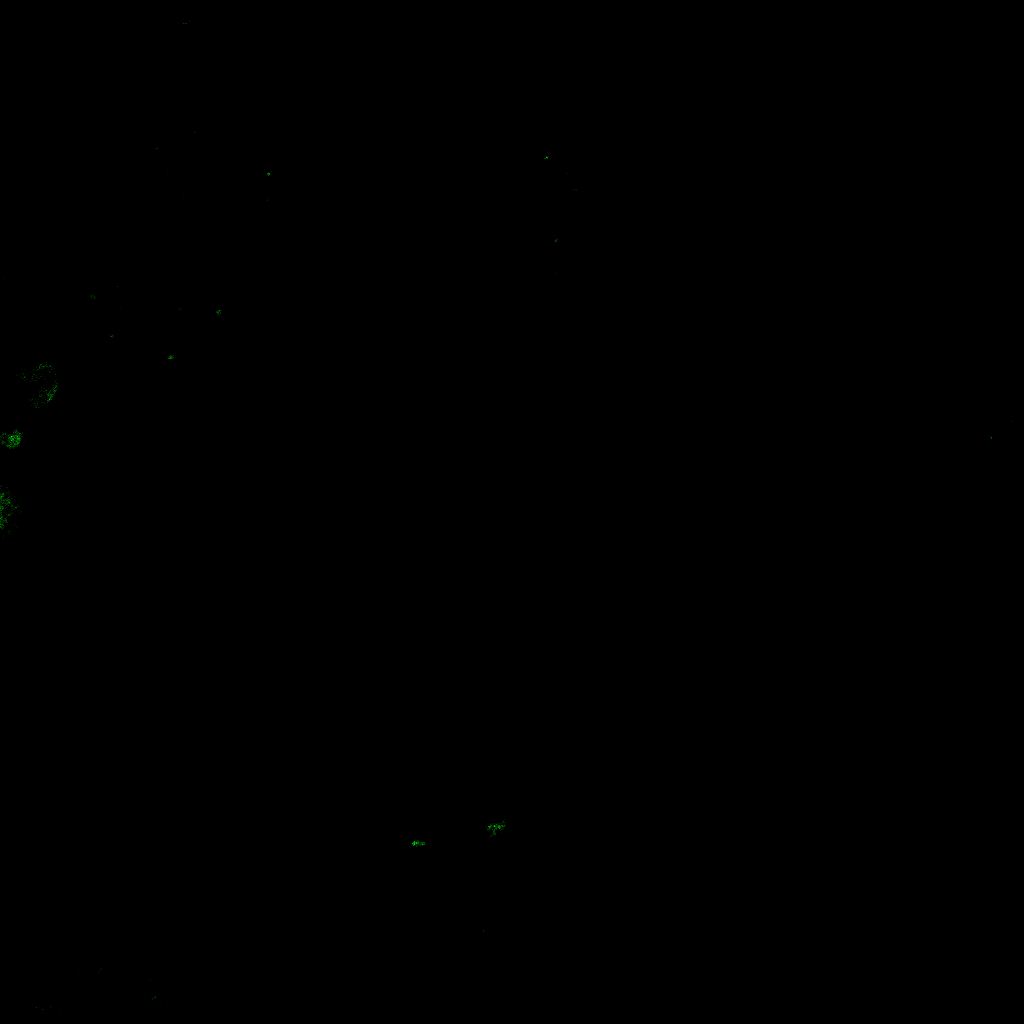

Supplement: Supplementary file 6 — Source data Fig. 4 [file 44319_2025_513_MOESM6_ESM.zip › Figure 4 Source Data/4E/K40Q ETO-/GH2AX.tif]

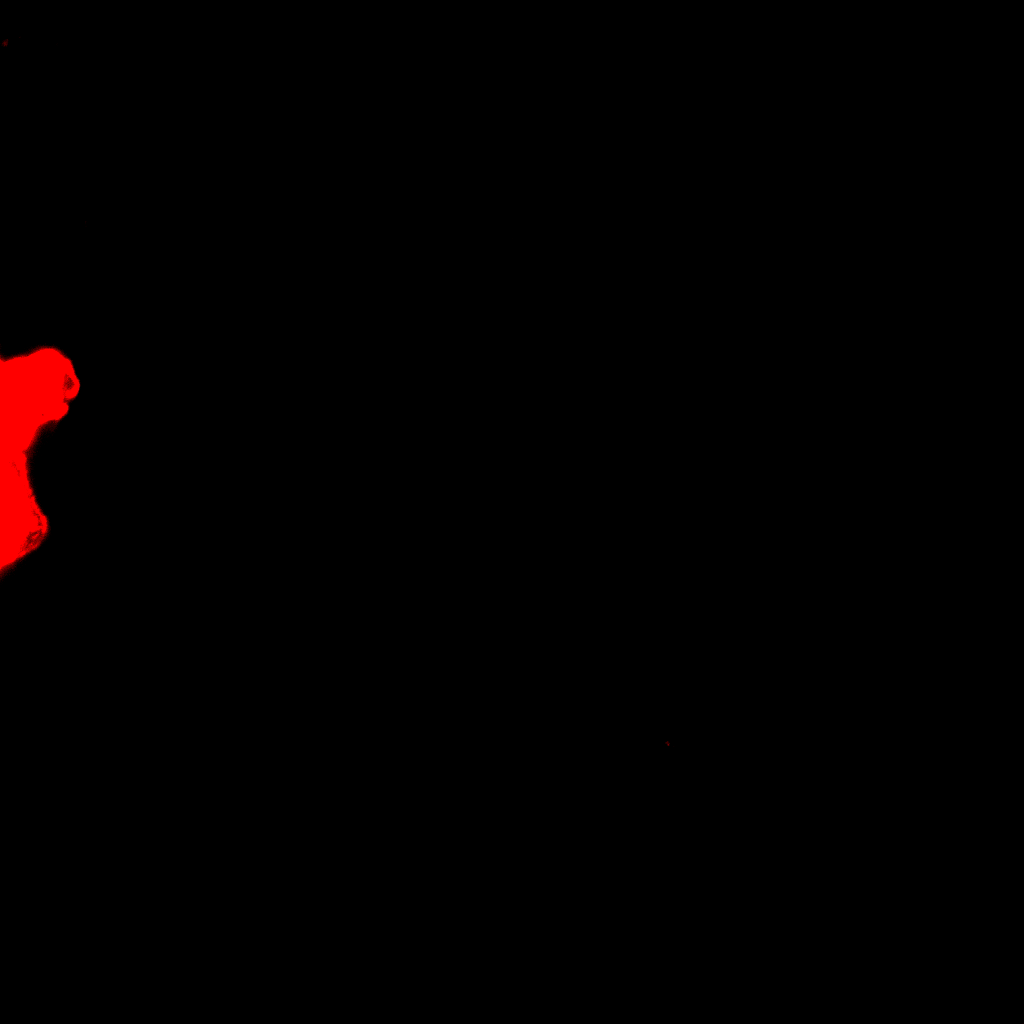

Supplement: Supplementary file 6 — Source data Fig. 4 [file 44319_2025_513_MOESM6_ESM.zip › Figure 4 Source Data/4E/K40Q ETO-/RAD51.tif]

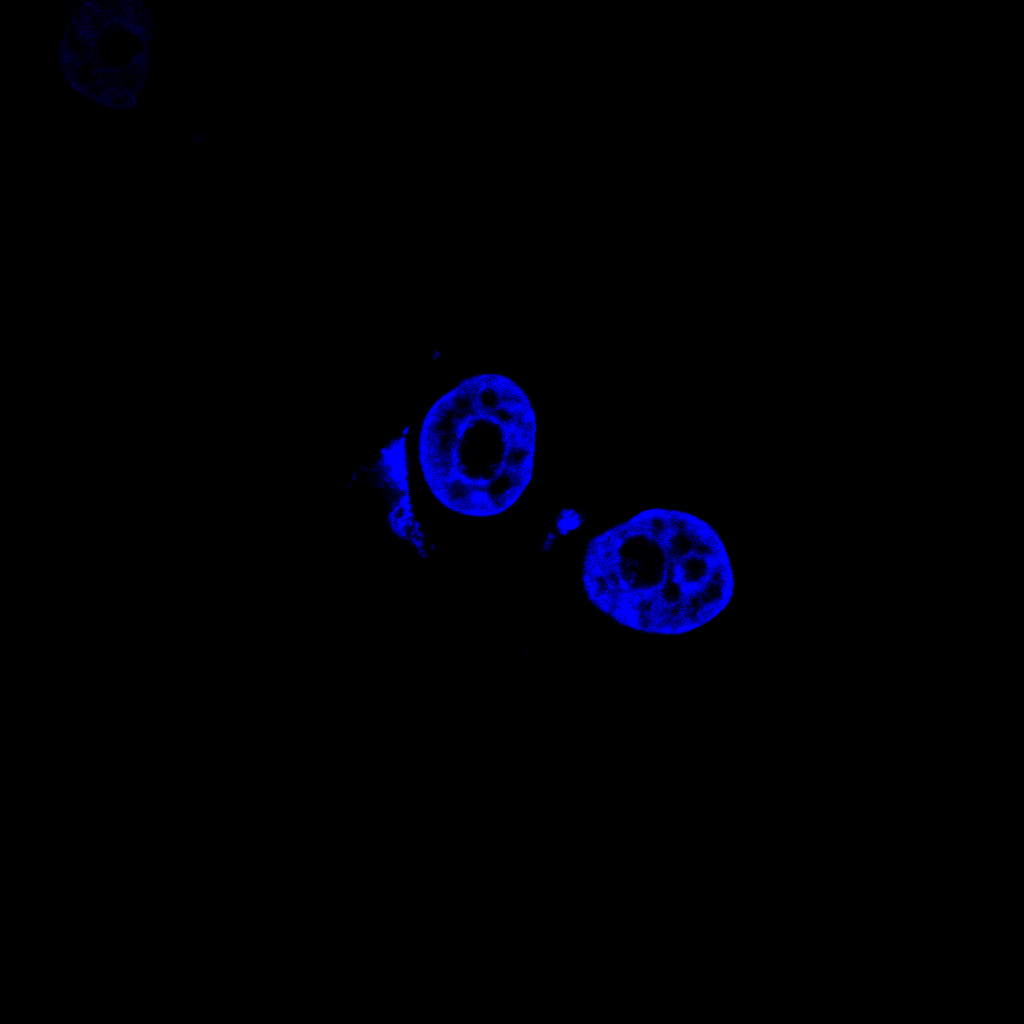

Supplement: Supplementary file 6 — Source data Fig. 4 [file 44319_2025_513_MOESM6_ESM.zip › Figure 4 Source Data/4E/K40R ETO+/DAPI.tif]

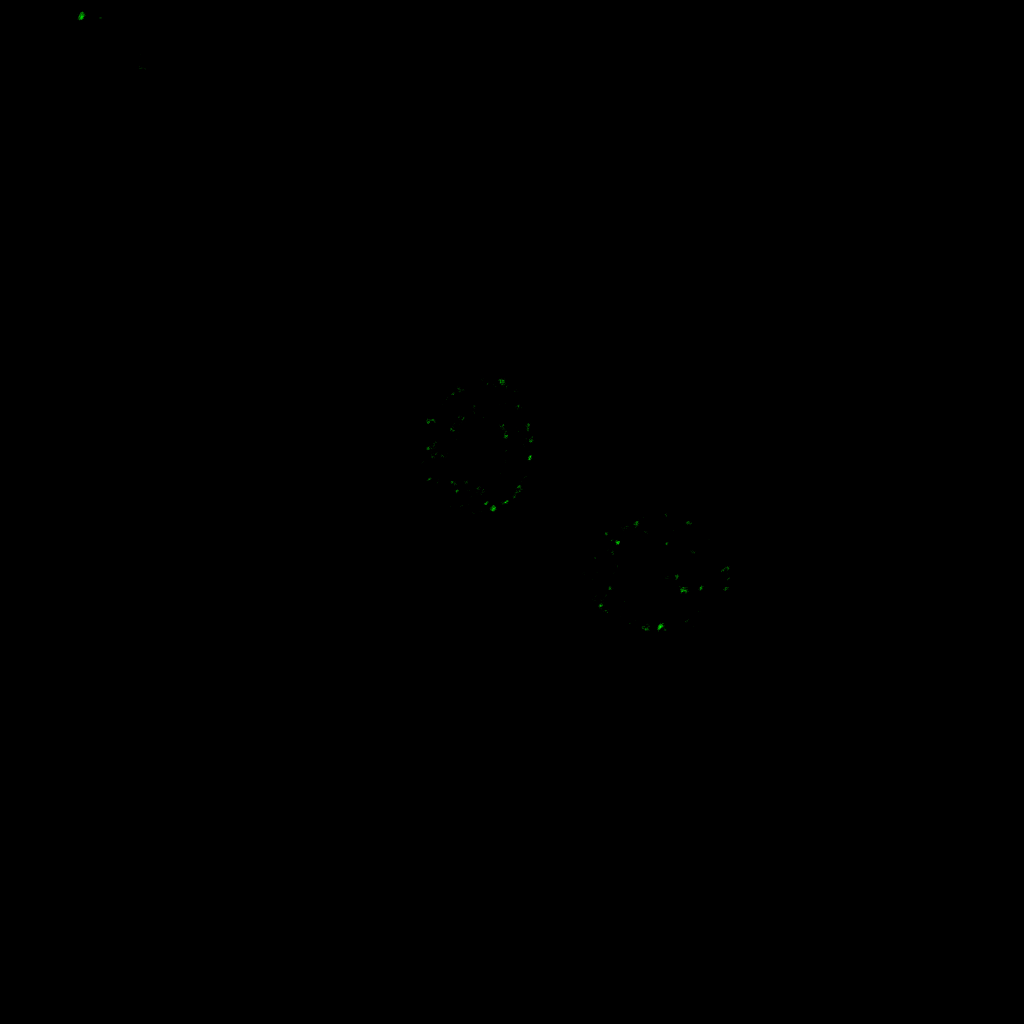

Supplement: Supplementary file 6 — Source data Fig. 4 [file 44319_2025_513_MOESM6_ESM.zip › Figure 4 Source Data/4E/K40R ETO+/GH2AX.tif]

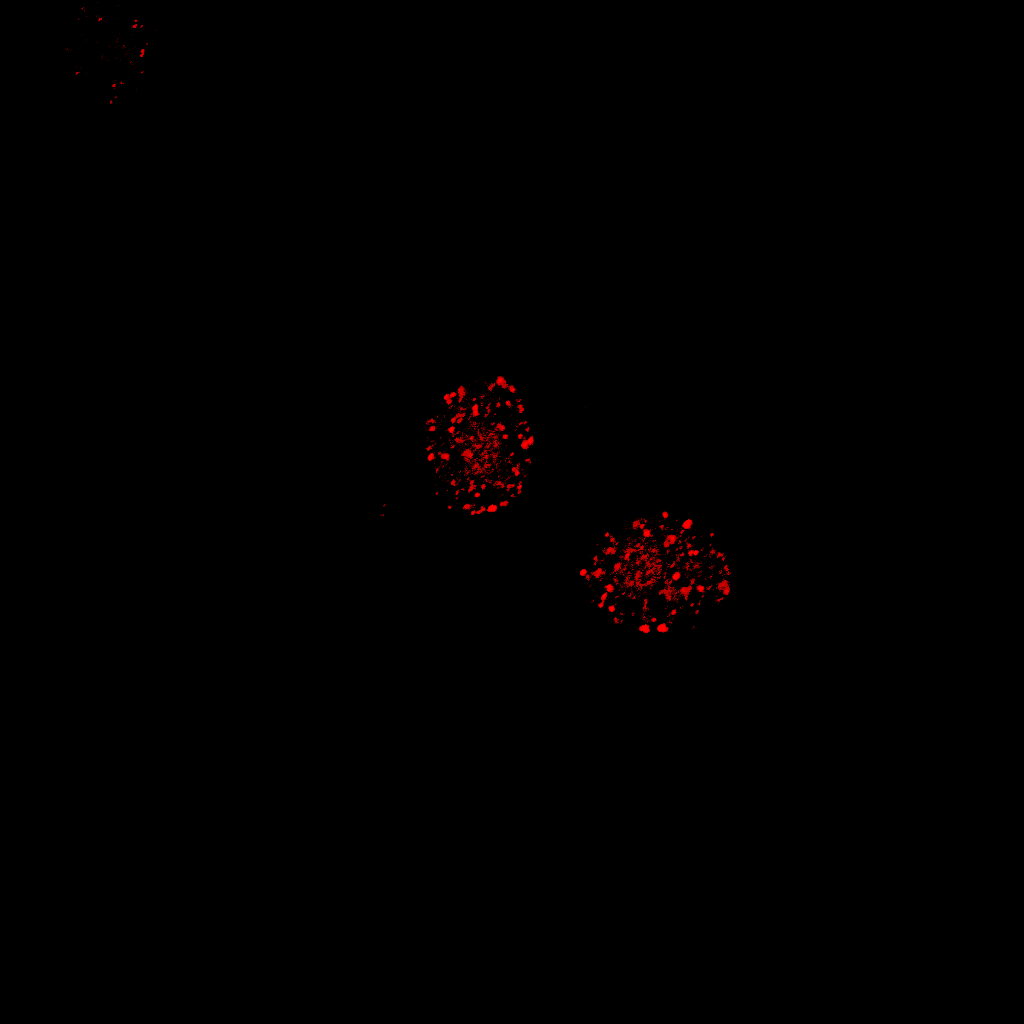

Supplement: Supplementary file 6 — Source data Fig. 4 [file 44319_2025_513_MOESM6_ESM.zip › Figure 4 Source Data/4E/K40R ETO+/RAD51.tif]

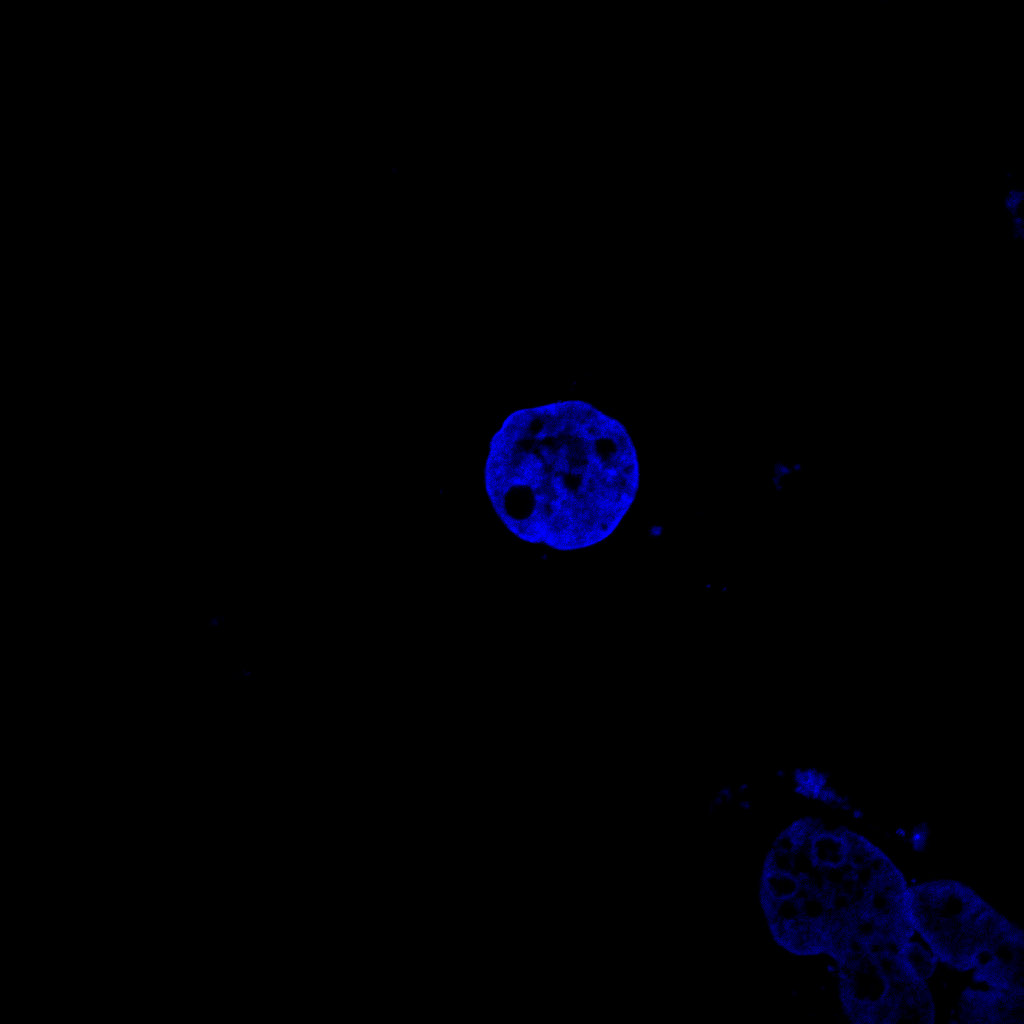

Supplement: Supplementary file 6 — Source data Fig. 4 [file 44319_2025_513_MOESM6_ESM.zip › Figure 4 Source Data/4E/K40R ETO-/DAPI.tif]

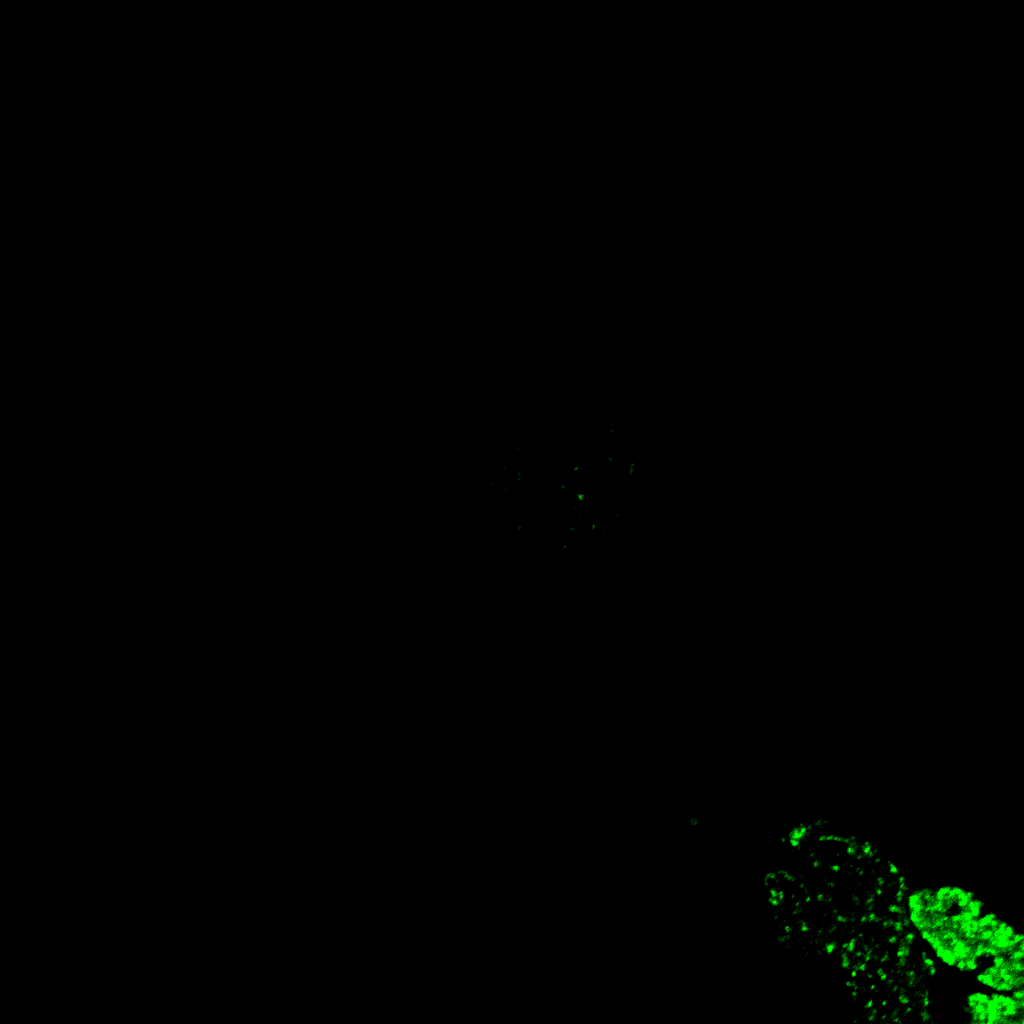

Supplement: Supplementary file 6 — Source data Fig. 4 [file 44319_2025_513_MOESM6_ESM.zip › Figure 4 Source Data/4E/K40R ETO-/GH2AX.tif]

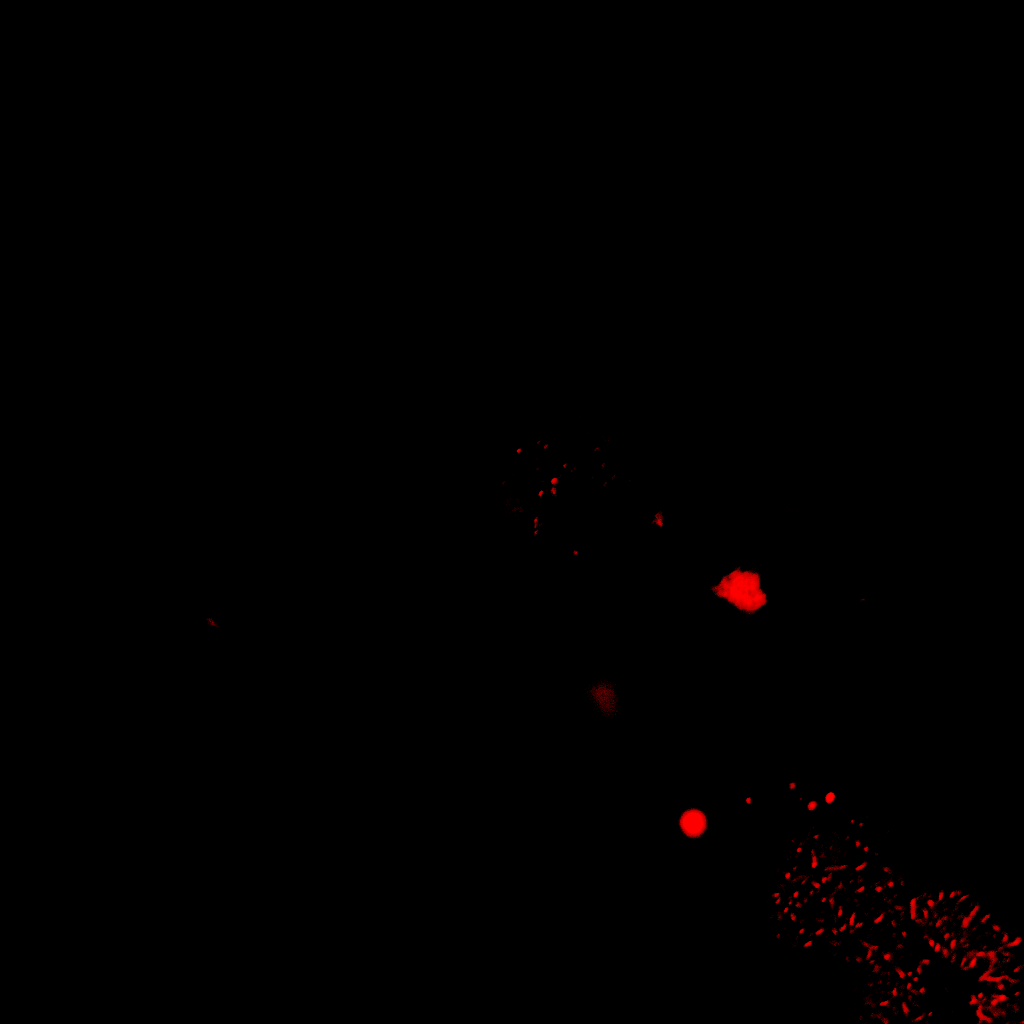

Supplement: Supplementary file 6 — Source data Fig. 4 [file 44319_2025_513_MOESM6_ESM.zip › Figure 4 Source Data/4E/K40R ETO-/RAD51.tif]

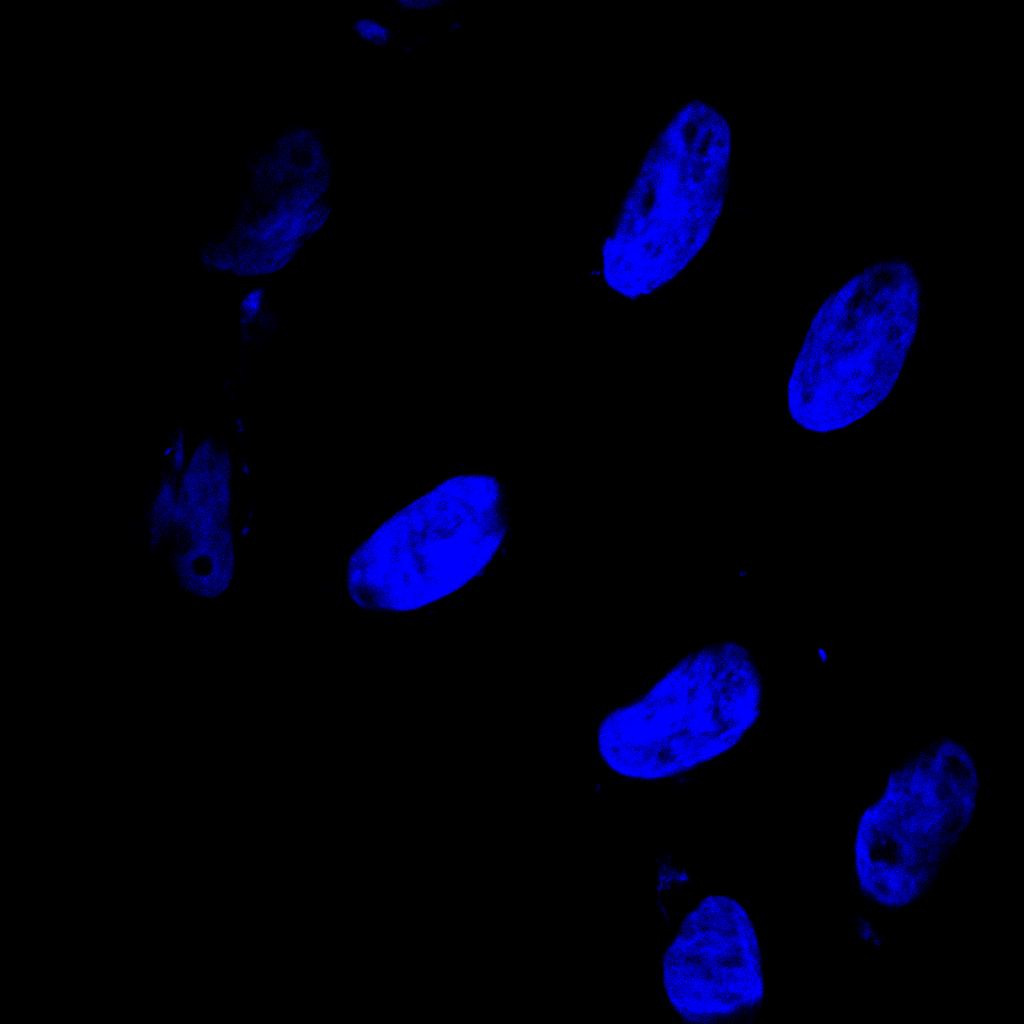

Supplement: Supplementary file 6 — Source data Fig. 4 [file 44319_2025_513_MOESM6_ESM.zip › Figure 4 Source Data/4E/NC ETO+/DAPI.tif]

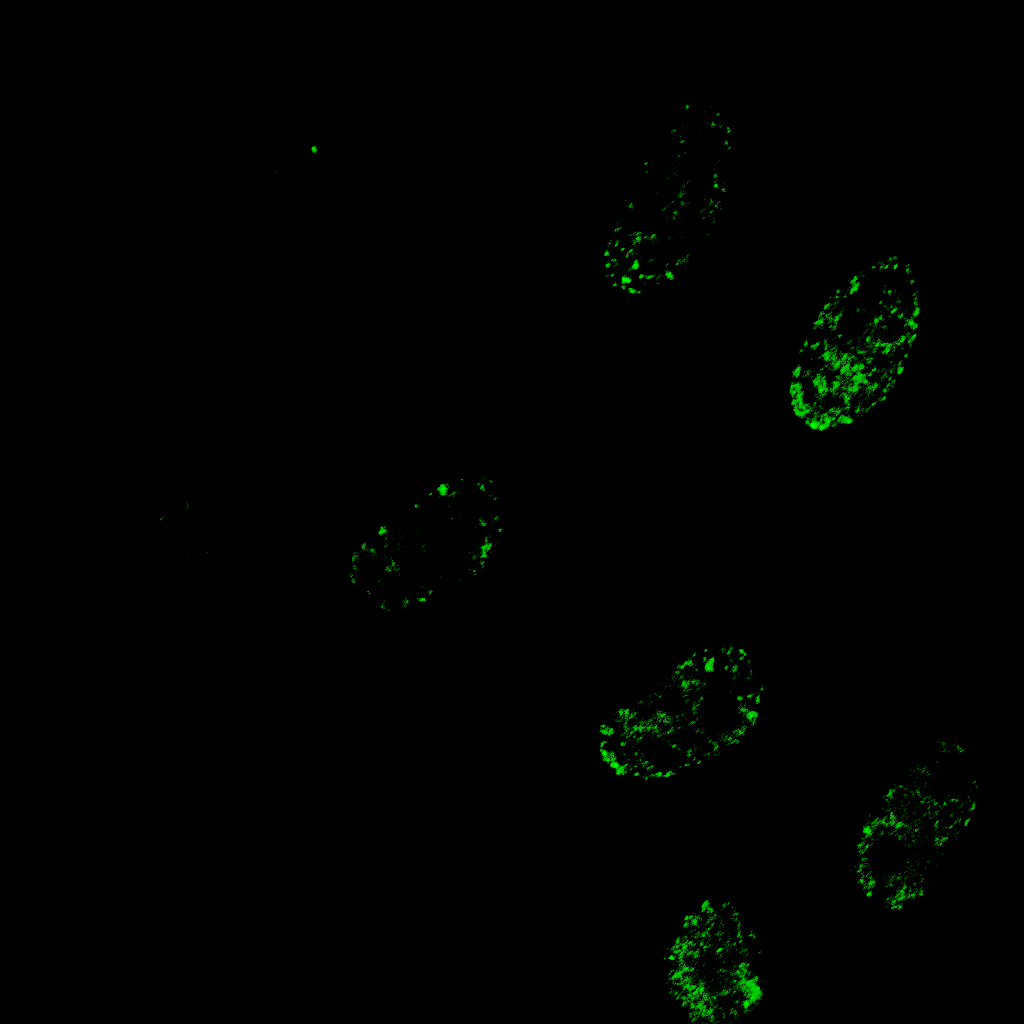

Supplement: Supplementary file 6 — Source data Fig. 4 [file 44319_2025_513_MOESM6_ESM.zip › Figure 4 Source Data/4E/NC ETO+/GH2AX.tif]

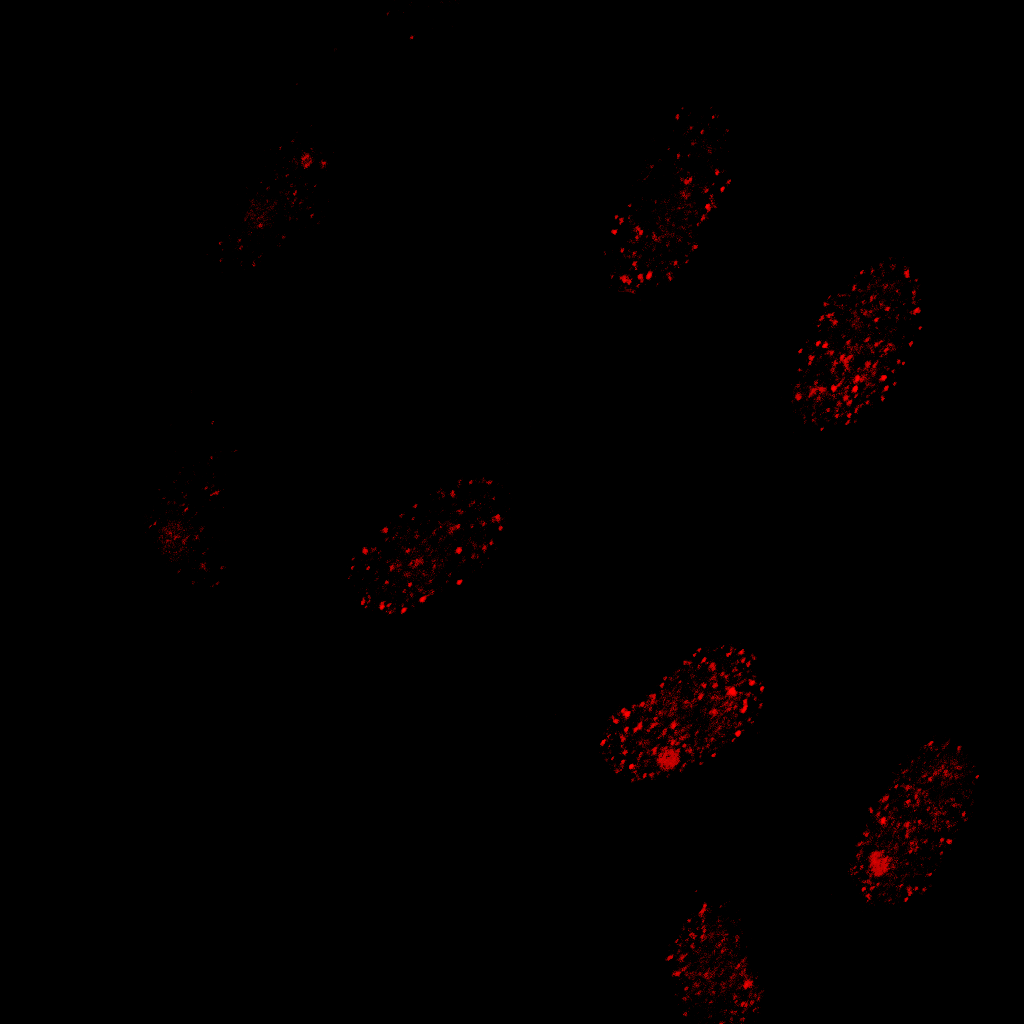

Supplement: Supplementary file 6 — Source data Fig. 4 [file 44319_2025_513_MOESM6_ESM.zip › Figure 4 Source Data/4E/NC ETO+/RAD51.tif]

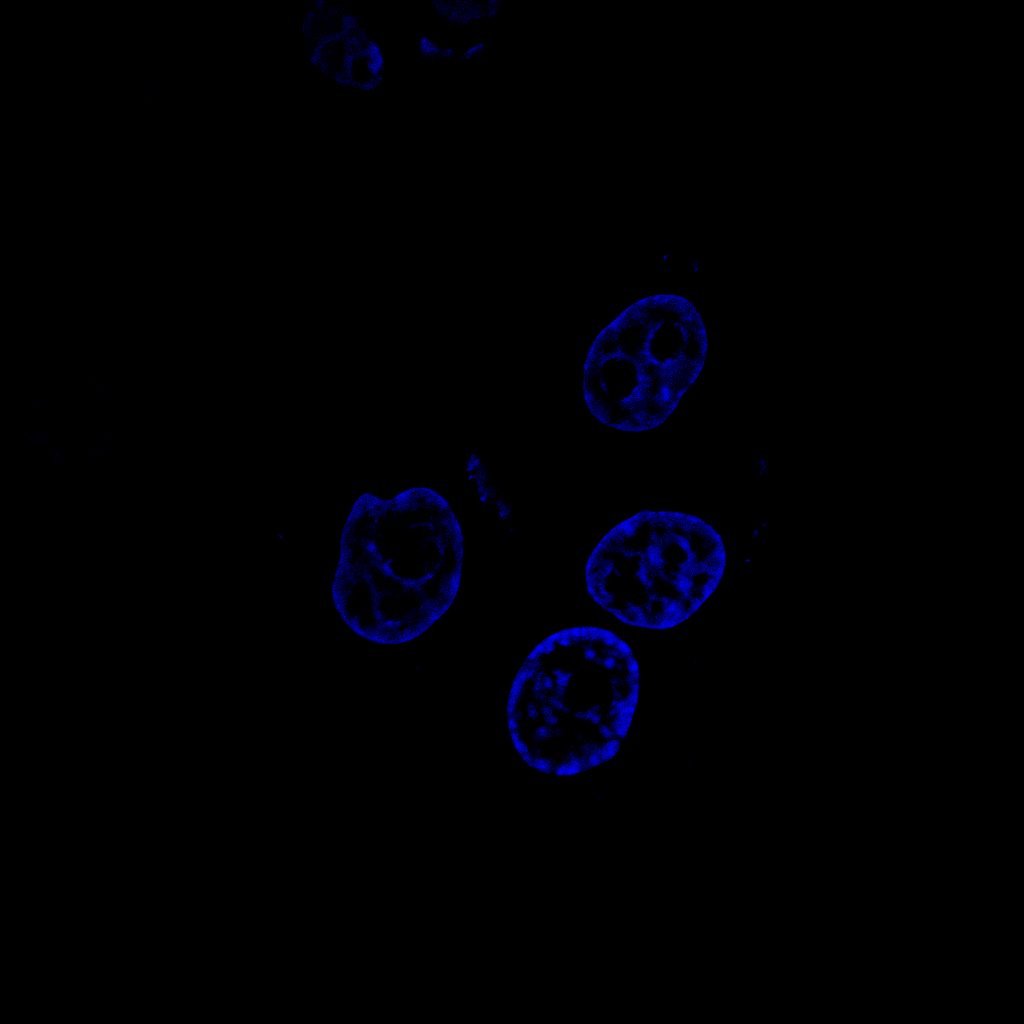

Supplement: Supplementary file 6 — Source data Fig. 4 [file 44319_2025_513_MOESM6_ESM.zip › Figure 4 Source Data/4E/NC ETO-/DAPI.tif]

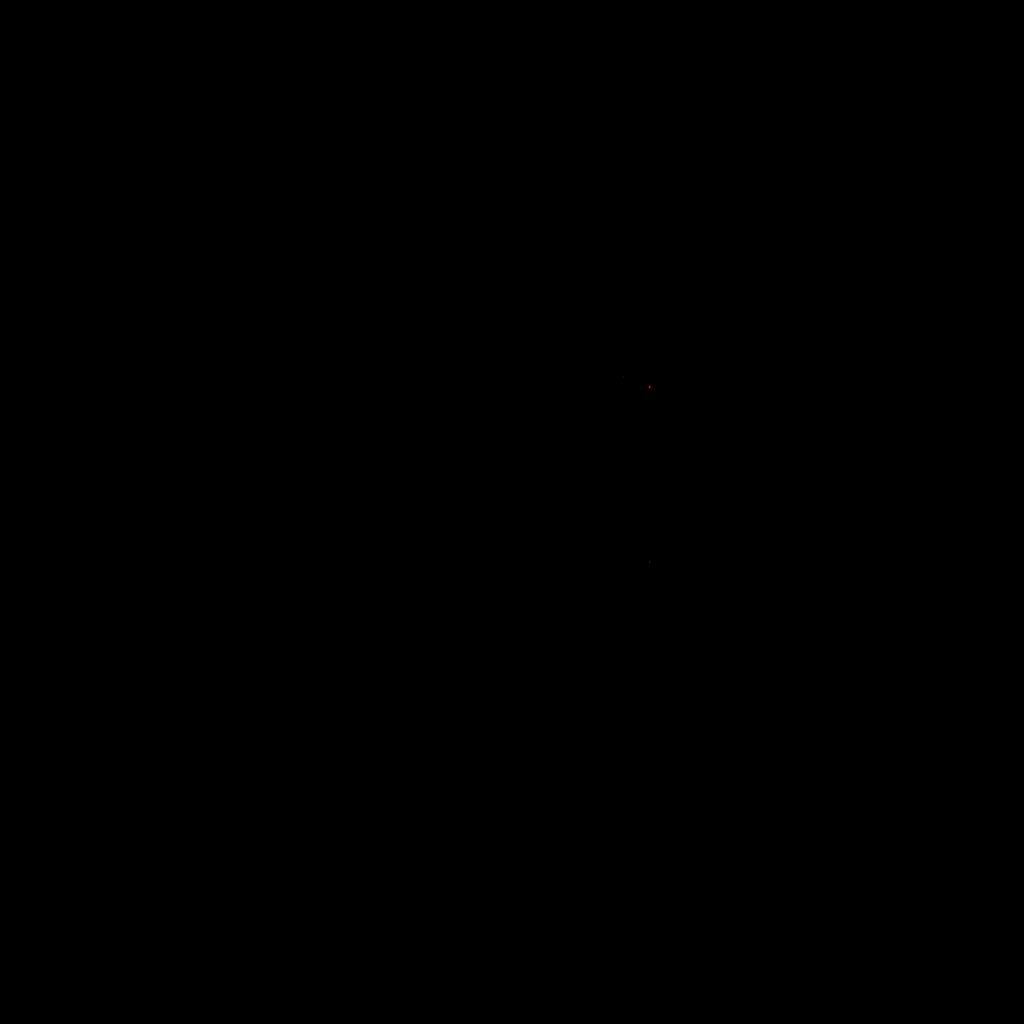

Supplement: Supplementary file 6 — Source data Fig. 4 [file 44319_2025_513_MOESM6_ESM.zip › Figure 4 Source Data/4E/NC ETO-/GH2AX.tif]

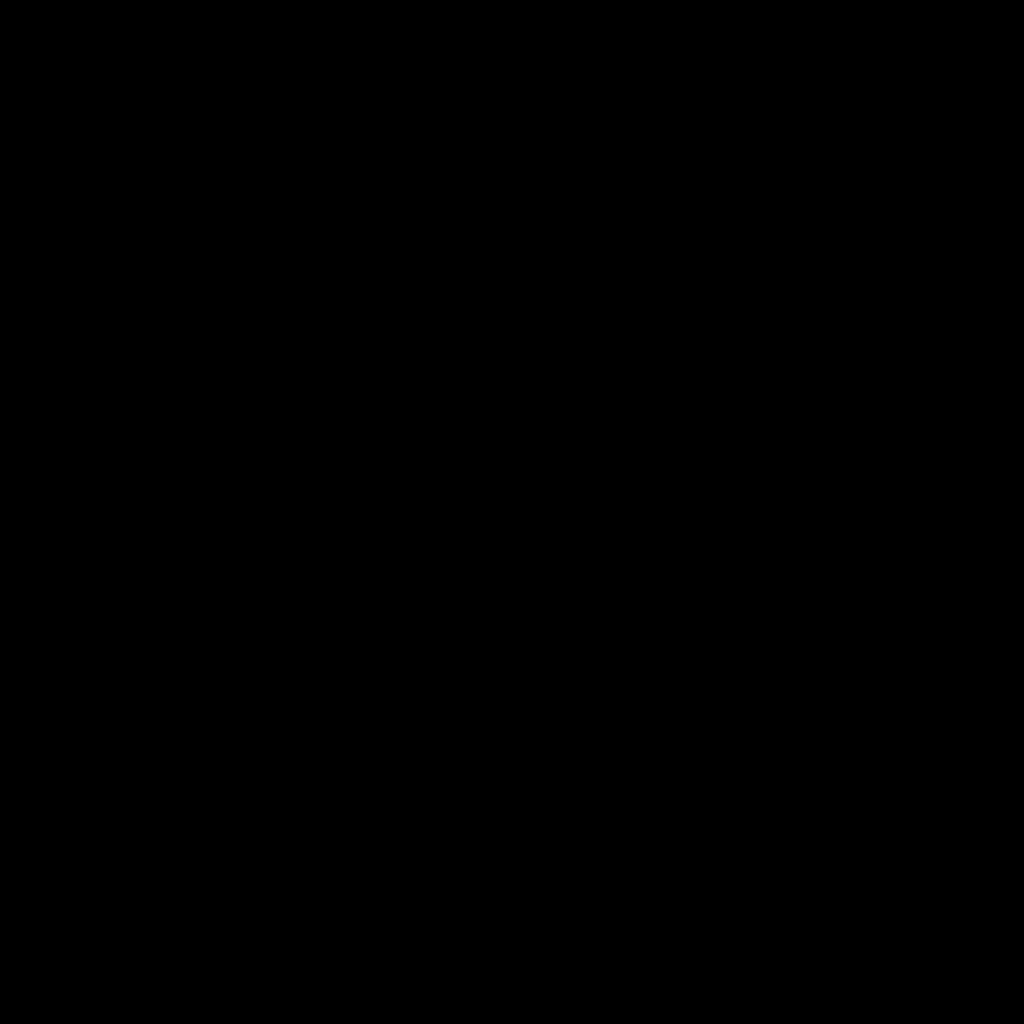

Supplement: Supplementary file 6 — Source data Fig. 4 [file 44319_2025_513_MOESM6_ESM.zip › Figure 4 Source Data/4E/NC ETO-/RAD51.tif]

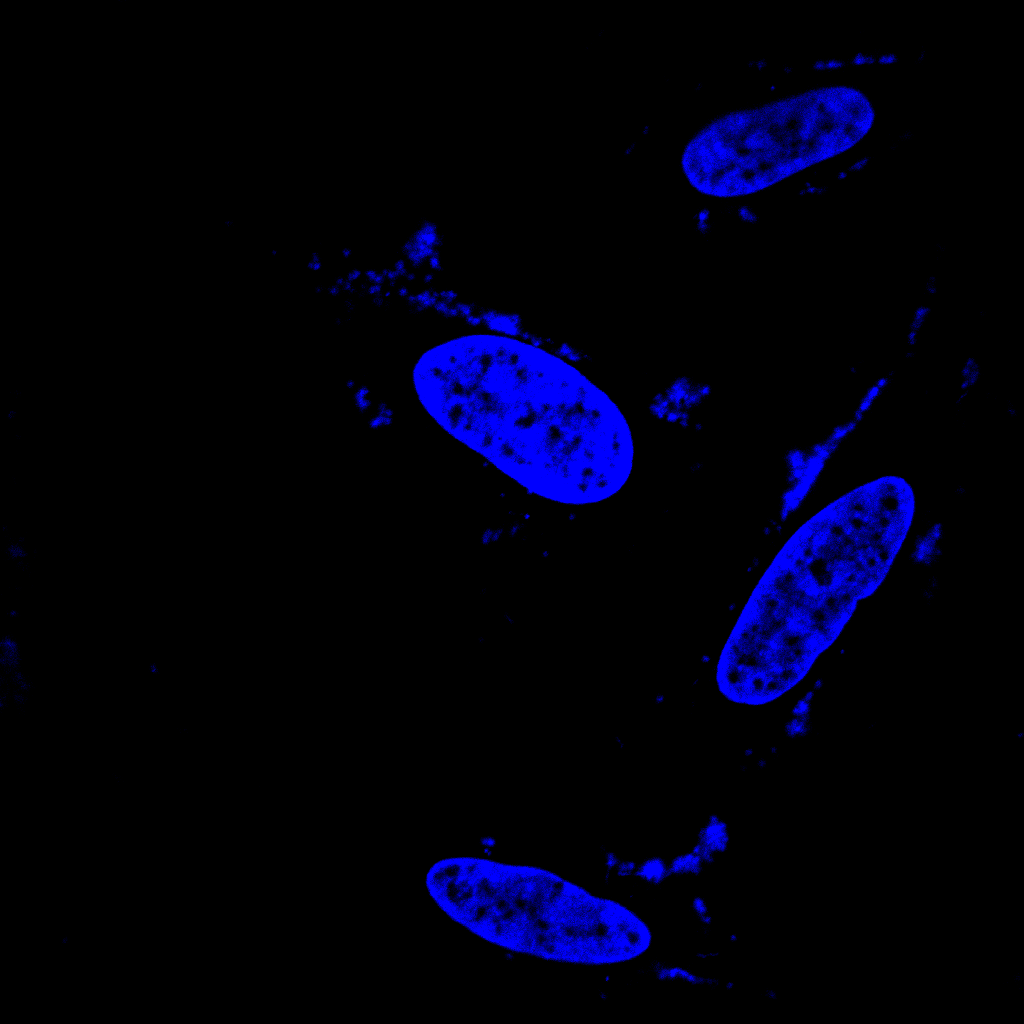

Supplement: Supplementary file 6 — Source data Fig. 4 [file 44319_2025_513_MOESM6_ESM.zip › Figure 4 Source Data/4E/WT ETO+/DAPI.tif]

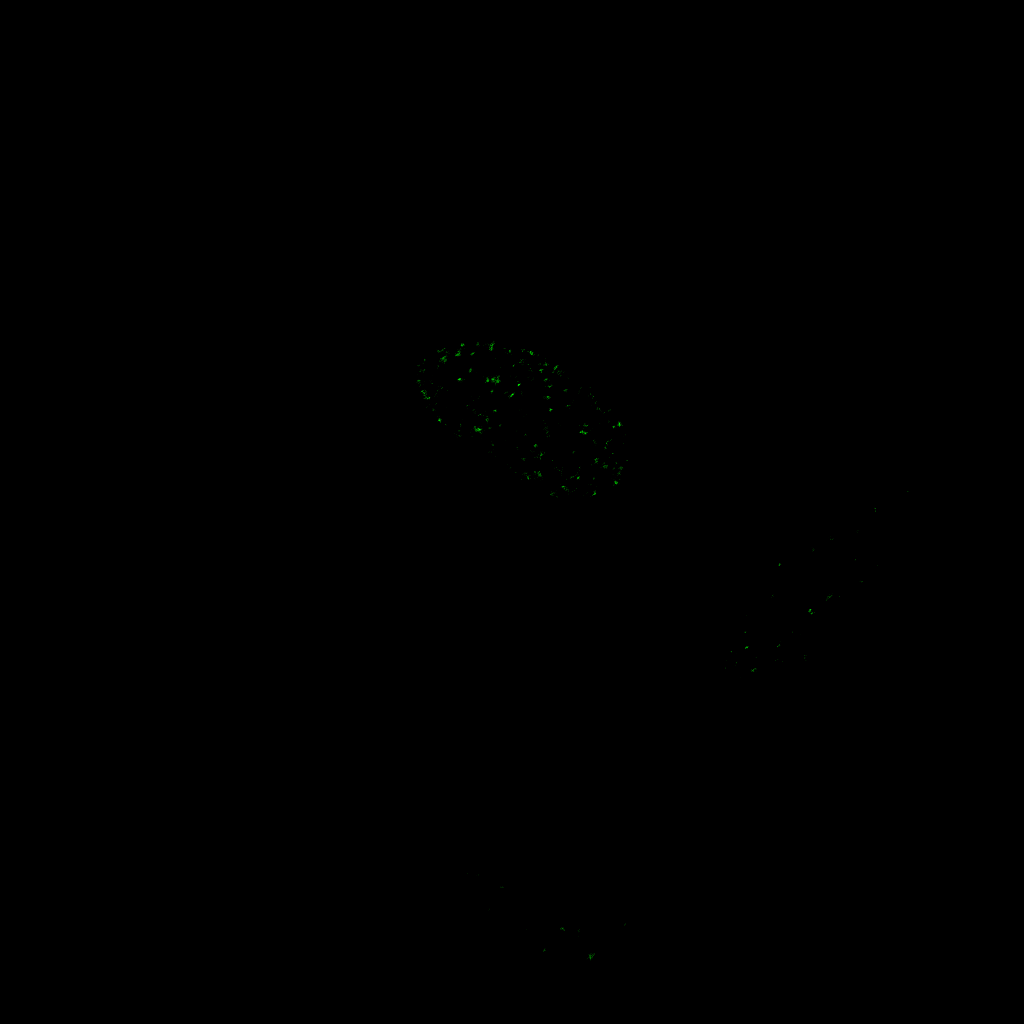

Supplement: Supplementary file 6 — Source data Fig. 4 [file 44319_2025_513_MOESM6_ESM.zip › Figure 4 Source Data/4E/WT ETO+/GH2AX.tif]

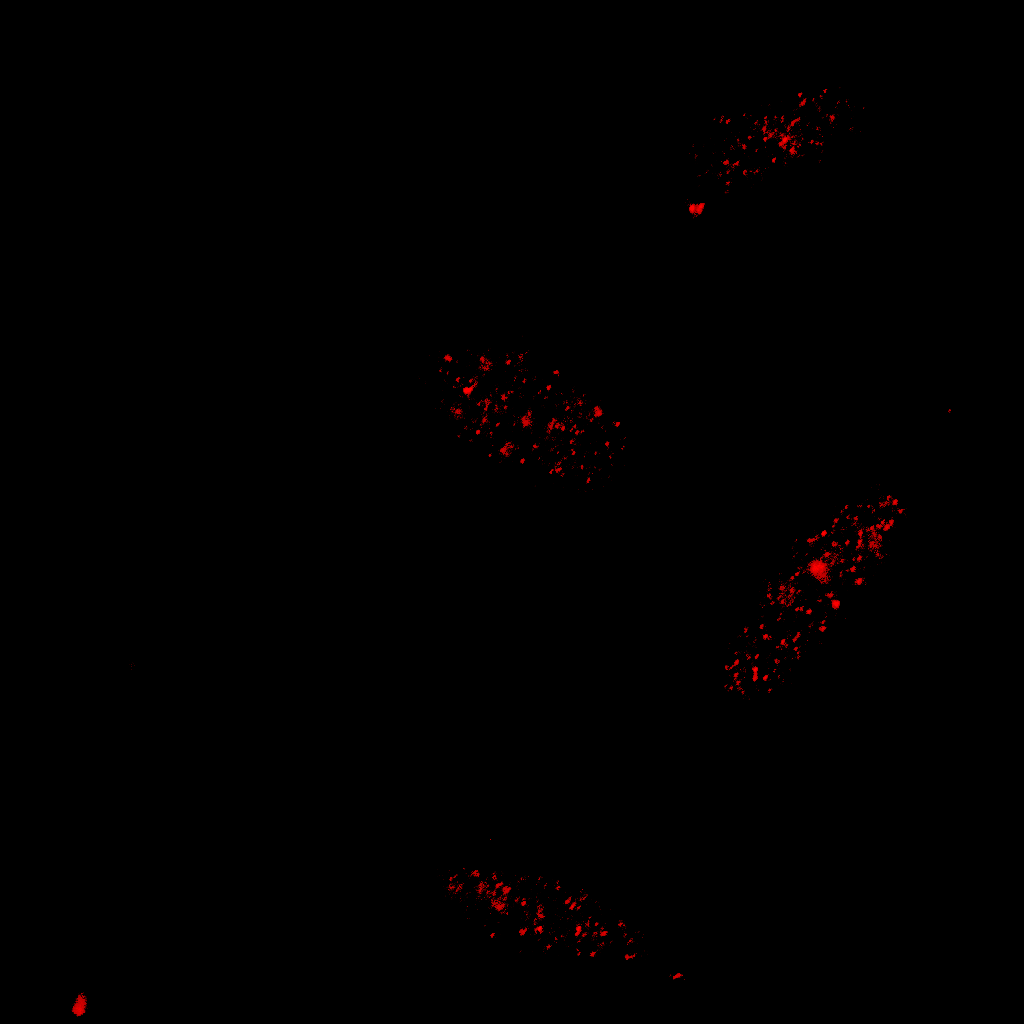

Supplement: Supplementary file 6 — Source data Fig. 4 [file 44319_2025_513_MOESM6_ESM.zip › Figure 4 Source Data/4E/WT ETO+/RAD51.tif]

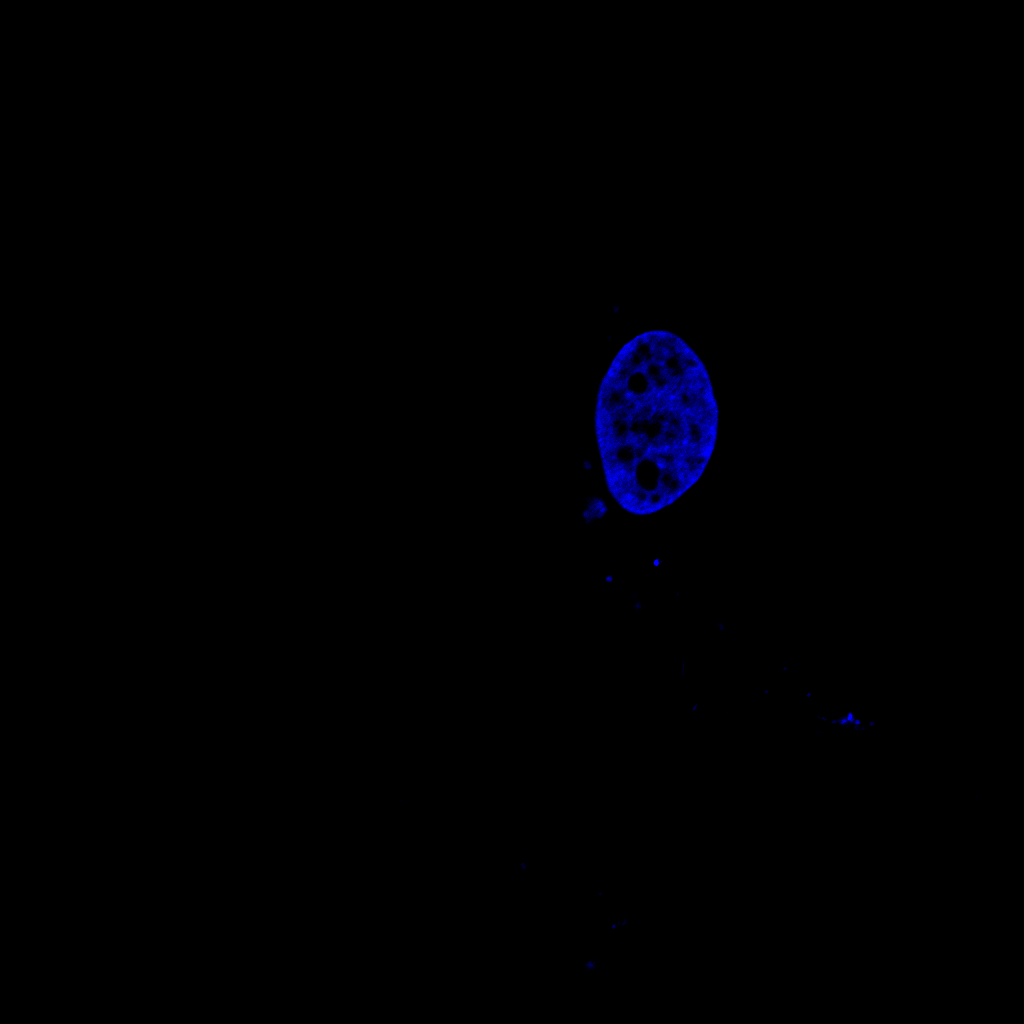

Supplement: Supplementary file 6 — Source data Fig. 4 [file 44319_2025_513_MOESM6_ESM.zip › Figure 4 Source Data/4E/WT ETO-/DAPI.tif]

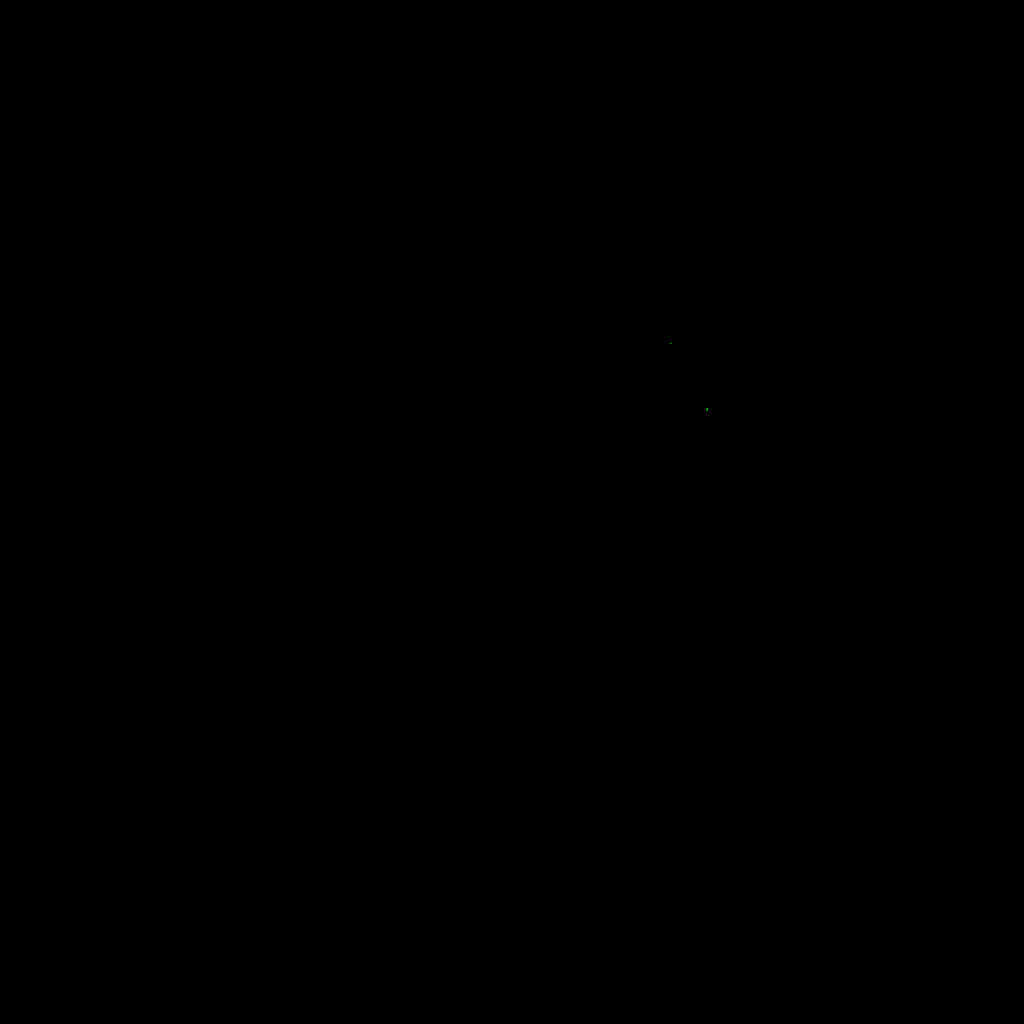

Supplement: Supplementary file 6 — Source data Fig. 4 [file 44319_2025_513_MOESM6_ESM.zip › Figure 4 Source Data/4E/WT ETO-/GH2AX.tif]

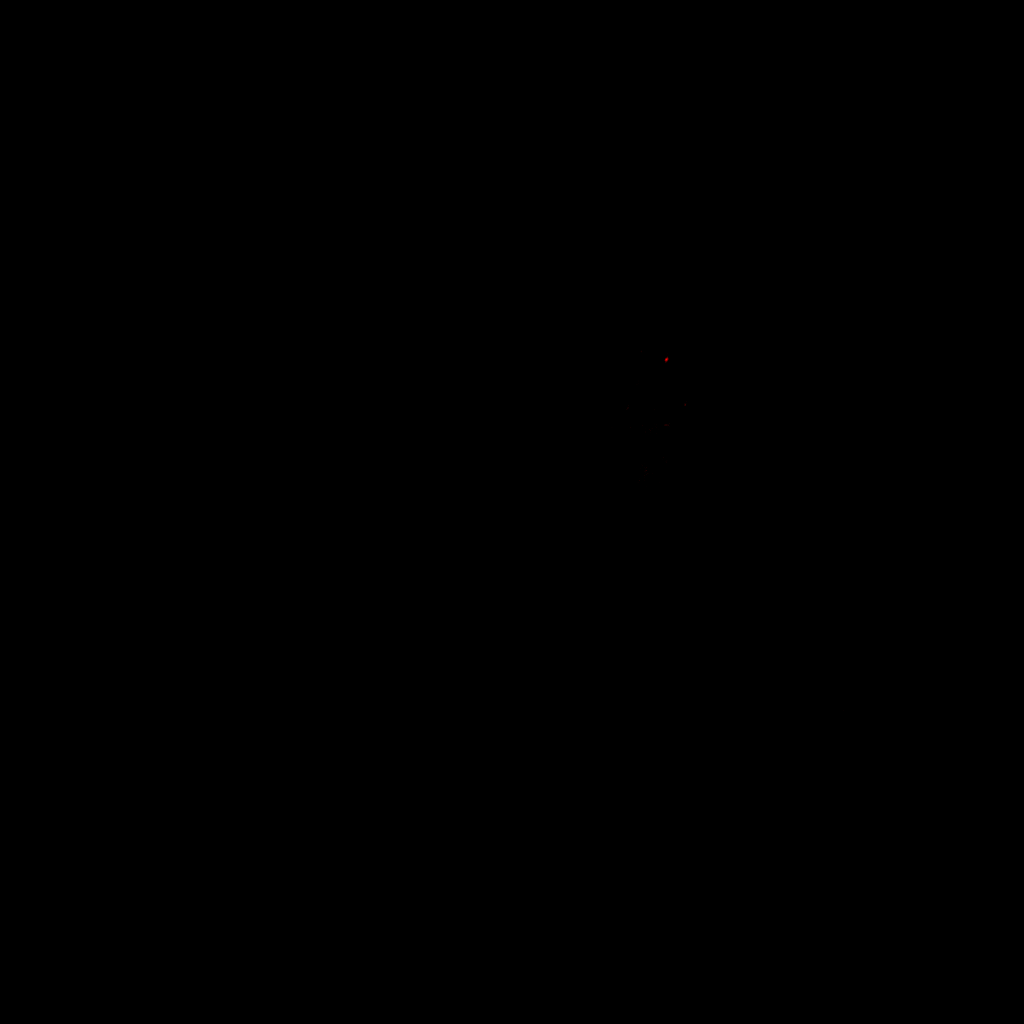

Supplement: Supplementary file 6 — Source data Fig. 4 [file 44319_2025_513_MOESM6_ESM.zip › Figure 4 Source Data/4E/WT ETO-/RAD51.tif]

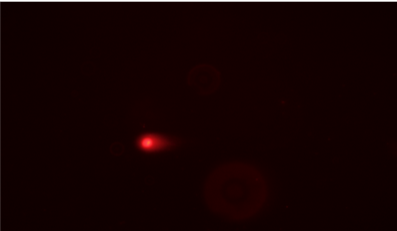

Supplement: Supplementary file 7 — Source data Fig. 5 [file 44319_2025_513_MOESM7_ESM.zip › Figure 5 Source Data/5E/EV ETO+.tif]

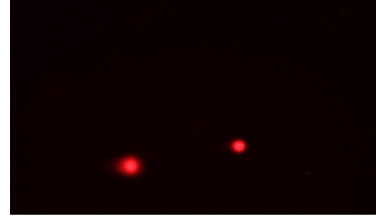

Supplement: Supplementary file 7 — Source data Fig. 5 [file 44319_2025_513_MOESM7_ESM.zip › Figure 5 Source Data/5E/EV ETO-.tif]

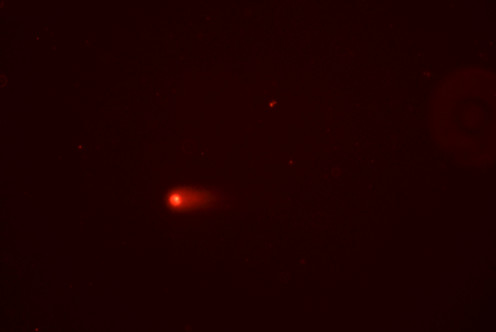

Supplement: Supplementary file 7 — Source data Fig. 5 [file 44319_2025_513_MOESM7_ESM.zip › Figure 5 Source Data/5E/PCAF ETO+.tif]

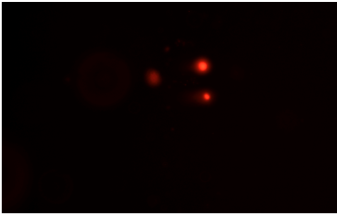

Supplement: Supplementary file 7 — Source data Fig. 5 [file 44319_2025_513_MOESM7_ESM.zip › Figure 5 Source Data/5E/PCAF ETO-.tif]

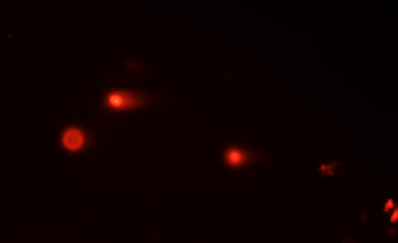

Supplement: Supplementary file 7 — Source data Fig. 5 [file 44319_2025_513_MOESM7_ESM.zip › Figure 5 Source Data/5F/sgNC ETO+.tif]

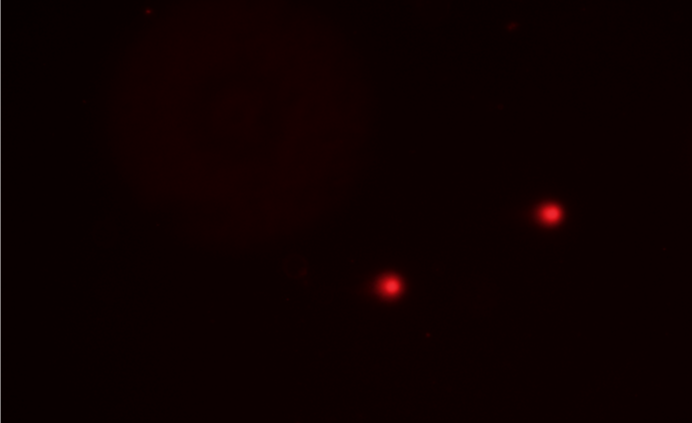

Supplement: Supplementary file 7 — Source data Fig. 5 [file 44319_2025_513_MOESM7_ESM.zip › Figure 5 Source Data/5F/sgNC ETO-.tif]

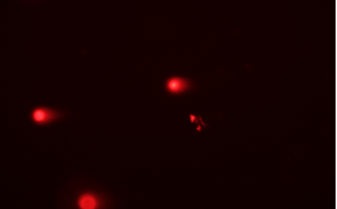

Supplement: Supplementary file 7 — Source data Fig. 5 [file 44319_2025_513_MOESM7_ESM.zip › Figure 5 Source Data/5F/sgPCAF ETO+.tif]

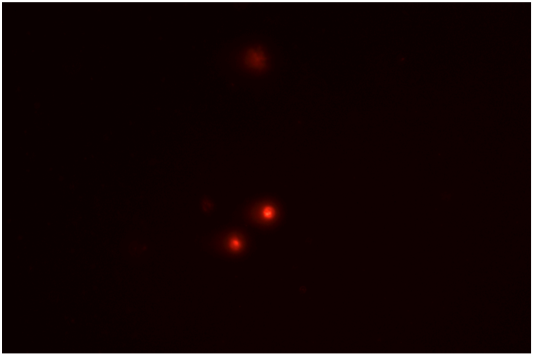

Supplement: Supplementary file 7 — Source data Fig. 5 [file 44319_2025_513_MOESM7_ESM.zip › Figure 5 Source Data/5F/sgPCAF ETO-.tif]

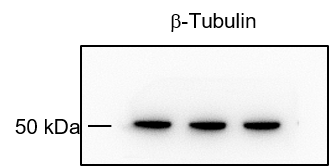

Supplement: Supplementary file 8 — Source data Fig. 6 [file 44319_2025_513_MOESM8_ESM.zip › Figure 6 Source Data/6A/bTubulin.tif]

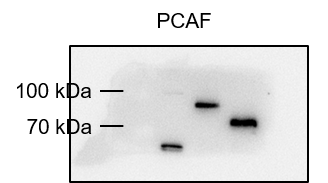

Supplement: Supplementary file 8 — Source data Fig. 6 [file 44319_2025_513_MOESM8_ESM.zip › Figure 6 Source Data/6A/PCAF.tif]

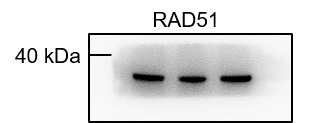

Supplement: Supplementary file 8 — Source data Fig. 6 [file 44319_2025_513_MOESM8_ESM.zip › Figure 6 Source Data/6A/RAD51.tif]

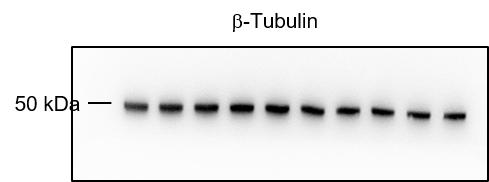

Supplement: Supplementary file 8 — Source data Fig. 6 [file 44319_2025_513_MOESM8_ESM.zip › Figure 6 Source Data/6B/bTubulin.tif]

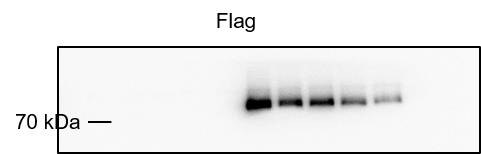

Supplement: Supplementary file 8 — Source data Fig. 6 [file 44319_2025_513_MOESM8_ESM.zip › Figure 6 Source Data/6B/Flag.tif]

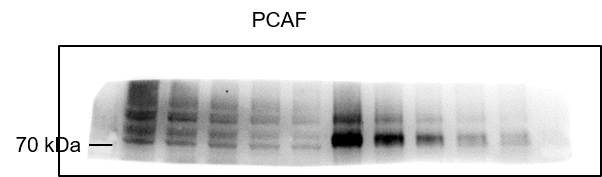

Supplement: Supplementary file 8 — Source data Fig. 6 [file 44319_2025_513_MOESM8_ESM.zip › Figure 6 Source Data/6B/PCAF.tif]

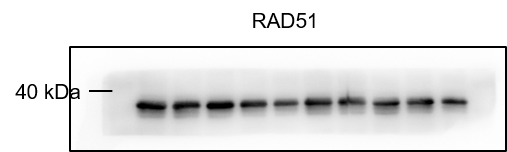

Supplement: Supplementary file 8 — Source data Fig. 6 [file 44319_2025_513_MOESM8_ESM.zip › Figure 6 Source Data/6B/RAD51.tif]

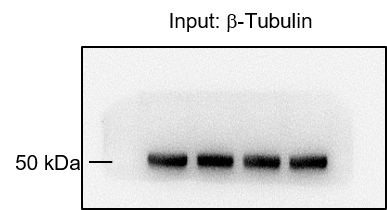

Supplement: Supplementary file 8 — Source data Fig. 6 [file 44319_2025_513_MOESM8_ESM.zip › Figure 6 Source Data/6C/Input bTubulin.tif]

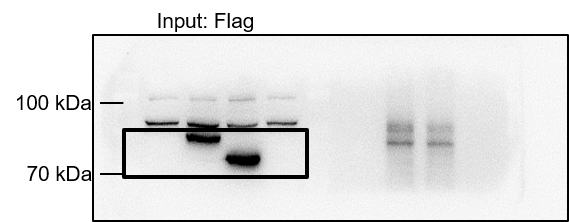

Supplement: Supplementary file 8 — Source data Fig. 6 [file 44319_2025_513_MOESM8_ESM.zip › Figure 6 Source Data/6C/Input Flag.tif]

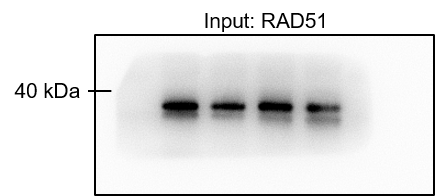

Supplement: Supplementary file 8 — Source data Fig. 6 [file 44319_2025_513_MOESM8_ESM.zip › Figure 6 Source Data/6C/Input RAD51.tif]

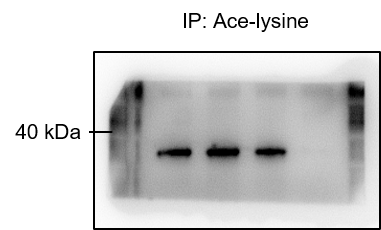

Supplement: Supplementary file 8 — Source data Fig. 6 [file 44319_2025_513_MOESM8_ESM.zip › Figure 6 Source Data/6C/IP Acelysine.tif]

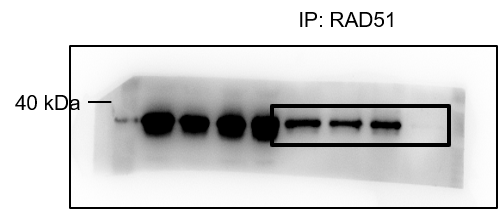

Supplement: Supplementary file 8 — Source data Fig. 6 [file 44319_2025_513_MOESM8_ESM.zip › Figure 6 Source Data/6C/IP RAD51.tif]

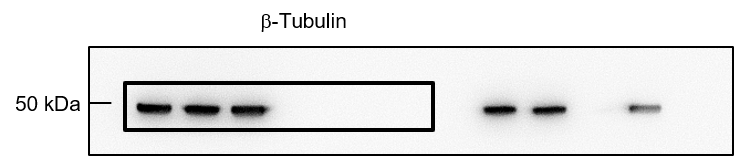

Supplement: Supplementary file 8 — Source data Fig. 6 [file 44319_2025_513_MOESM8_ESM.zip › Figure 6 Source Data/6D/bTubulin.tif]

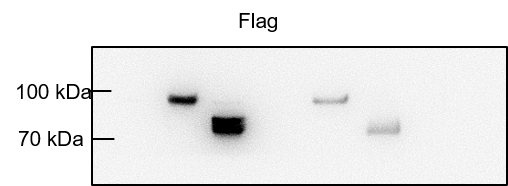

Supplement: Supplementary file 8 — Source data Fig. 6 [file 44319_2025_513_MOESM8_ESM.zip › Figure 6 Source Data/6D/Flag.tif]

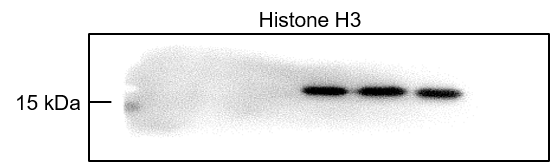

Supplement: Supplementary file 8 — Source data Fig. 6 [file 44319_2025_513_MOESM8_ESM.zip › Figure 6 Source Data/6D/Histone H3.tif]

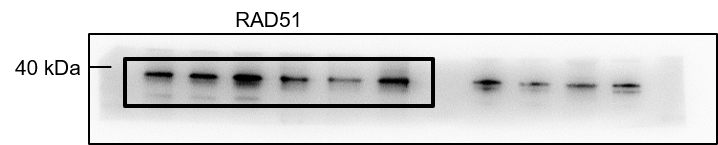

Supplement: Supplementary file 8 — Source data Fig. 6 [file 44319_2025_513_MOESM8_ESM.zip › Figure 6 Source Data/6D/RAD51.tif]

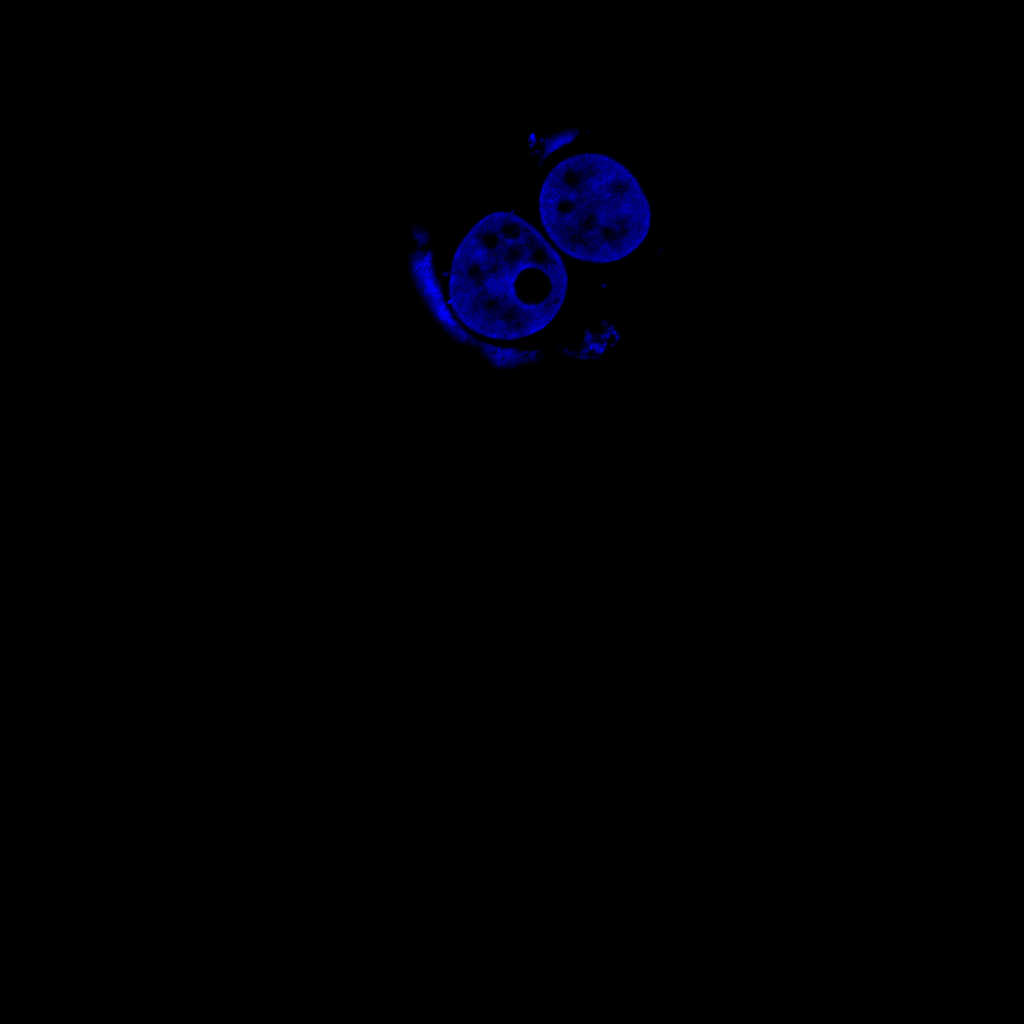

Supplement: Supplementary file 8 — Source data Fig. 6 [file 44319_2025_513_MOESM8_ESM.zip › Figure 6 Source Data/6E/DHAT ETO+/DAPI.tif]

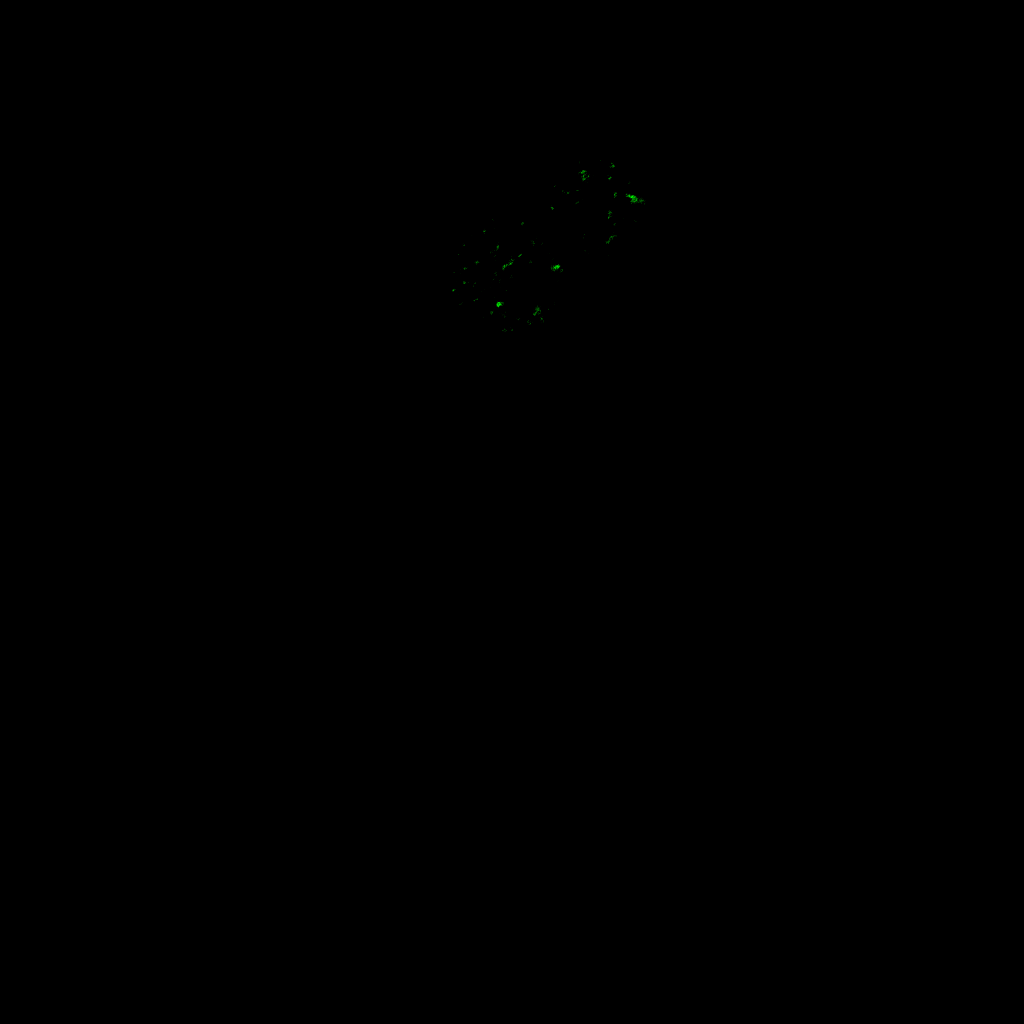

Supplement: Supplementary file 8 — Source data Fig. 6 [file 44319_2025_513_MOESM8_ESM.zip › Figure 6 Source Data/6E/DHAT ETO+/GH2AX.tif]

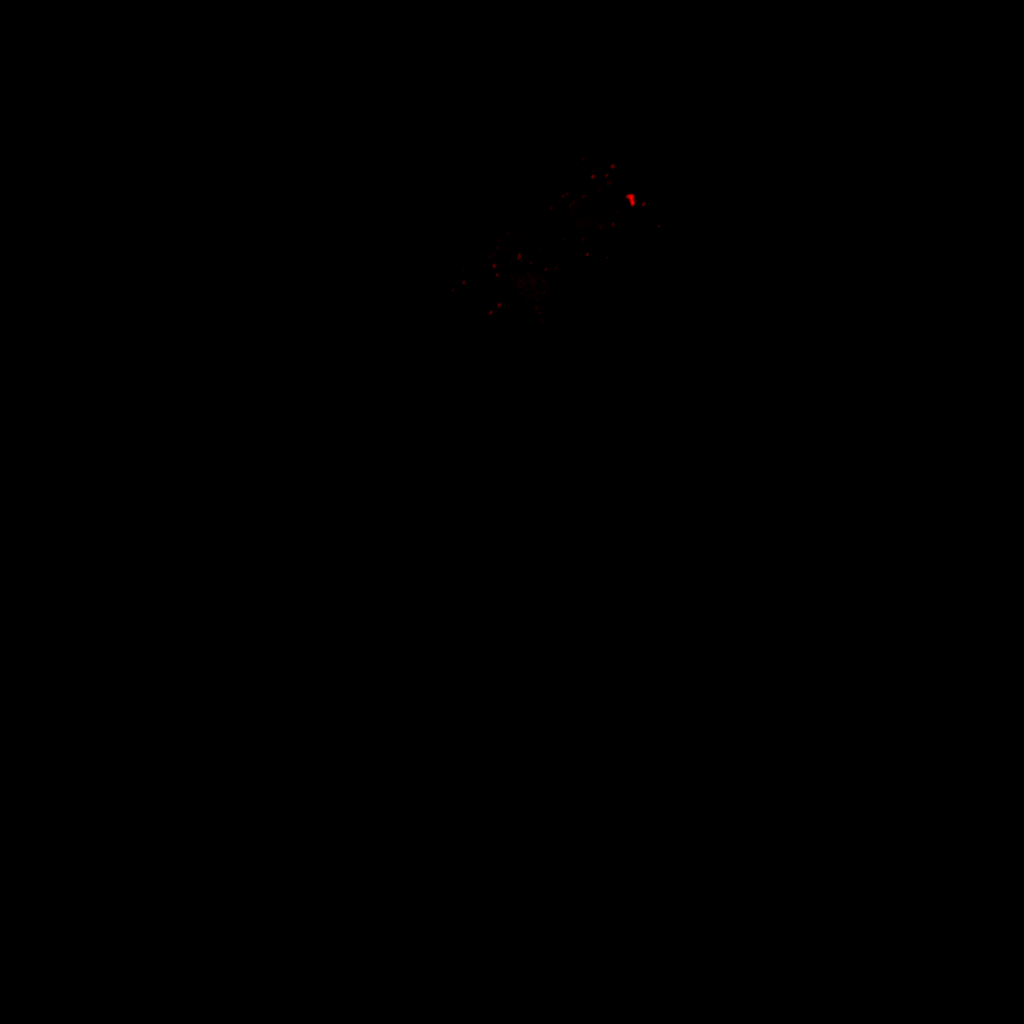

Supplement: Supplementary file 8 — Source data Fig. 6 [file 44319_2025_513_MOESM8_ESM.zip › Figure 6 Source Data/6E/DHAT ETO+/RAD51.tif]

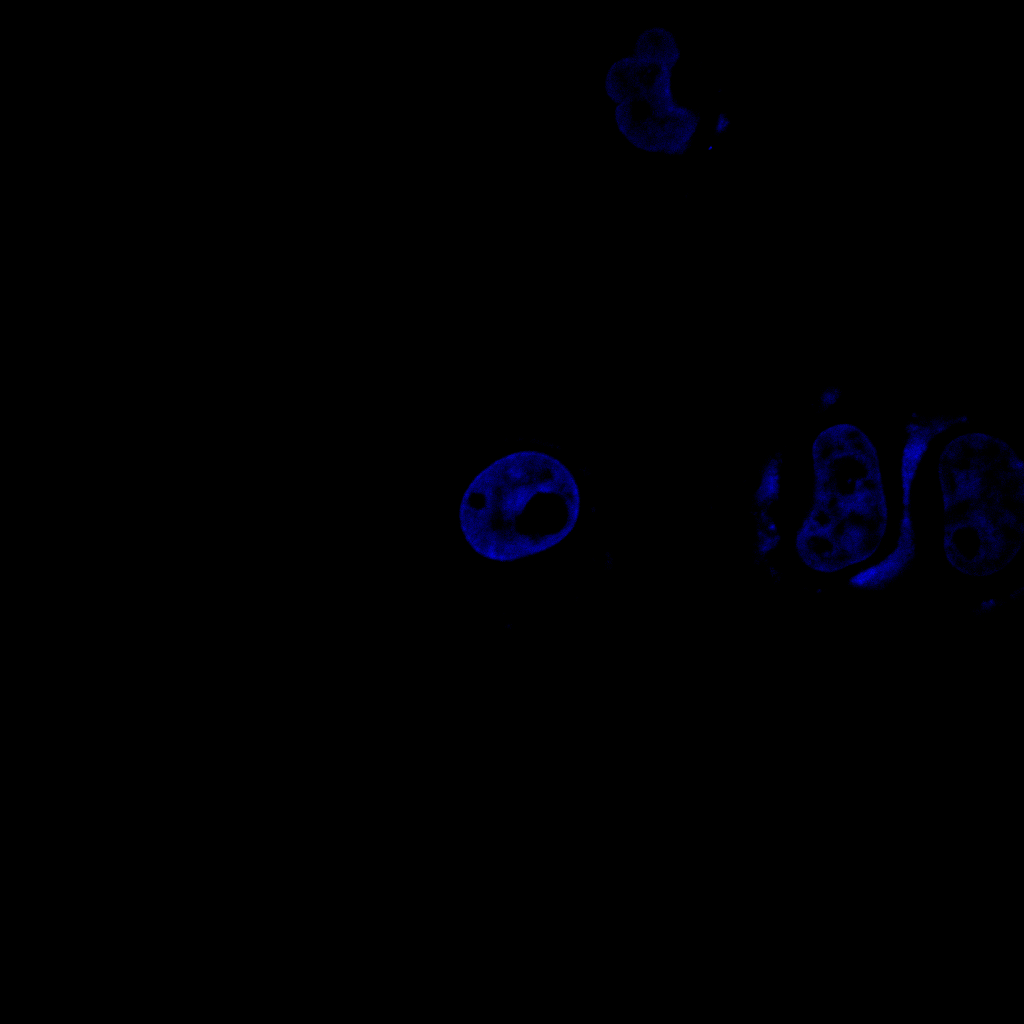

Supplement: Supplementary file 8 — Source data Fig. 6 [file 44319_2025_513_MOESM8_ESM.zip › Figure 6 Source Data/6E/DHAT ETO-/DAPI.tif]

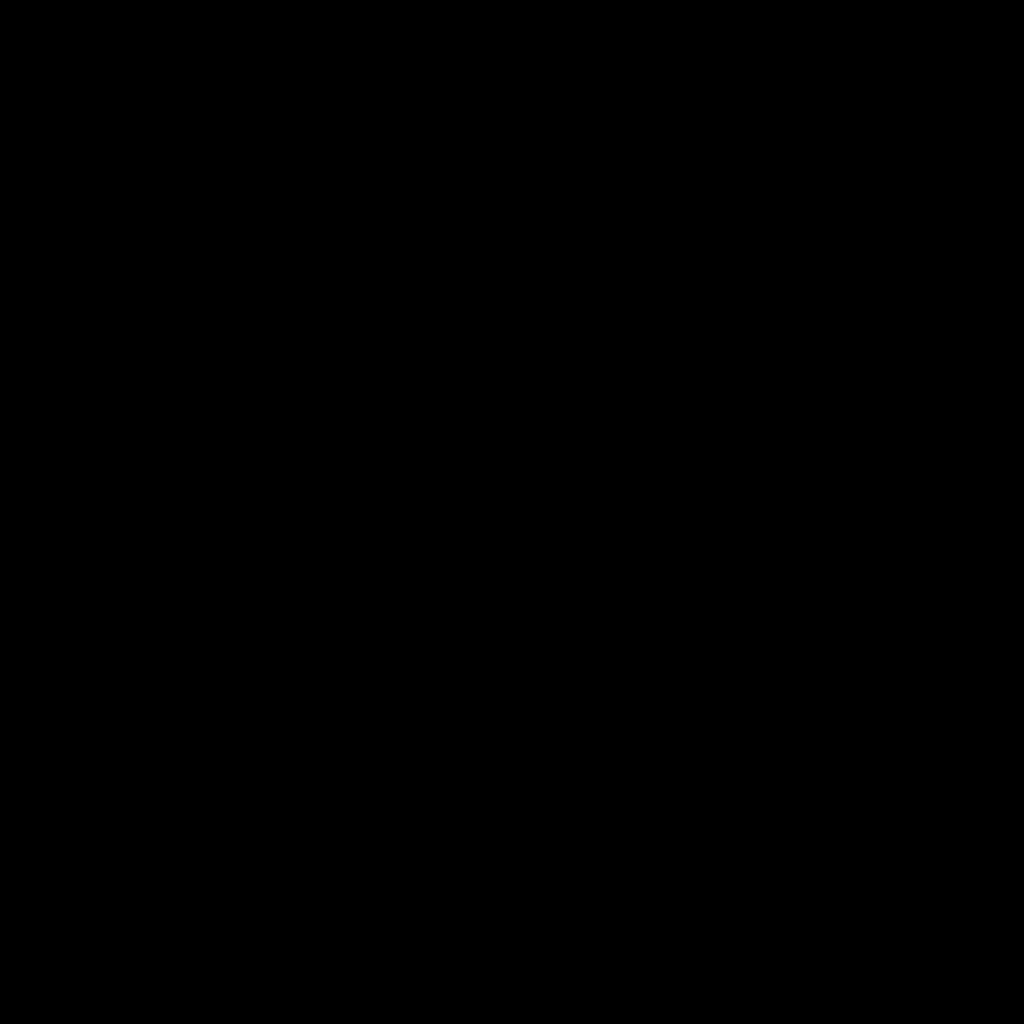

Supplement: Supplementary file 8 — Source data Fig. 6 [file 44319_2025_513_MOESM8_ESM.zip › Figure 6 Source Data/6E/DHAT ETO-/GH2AX.tif]

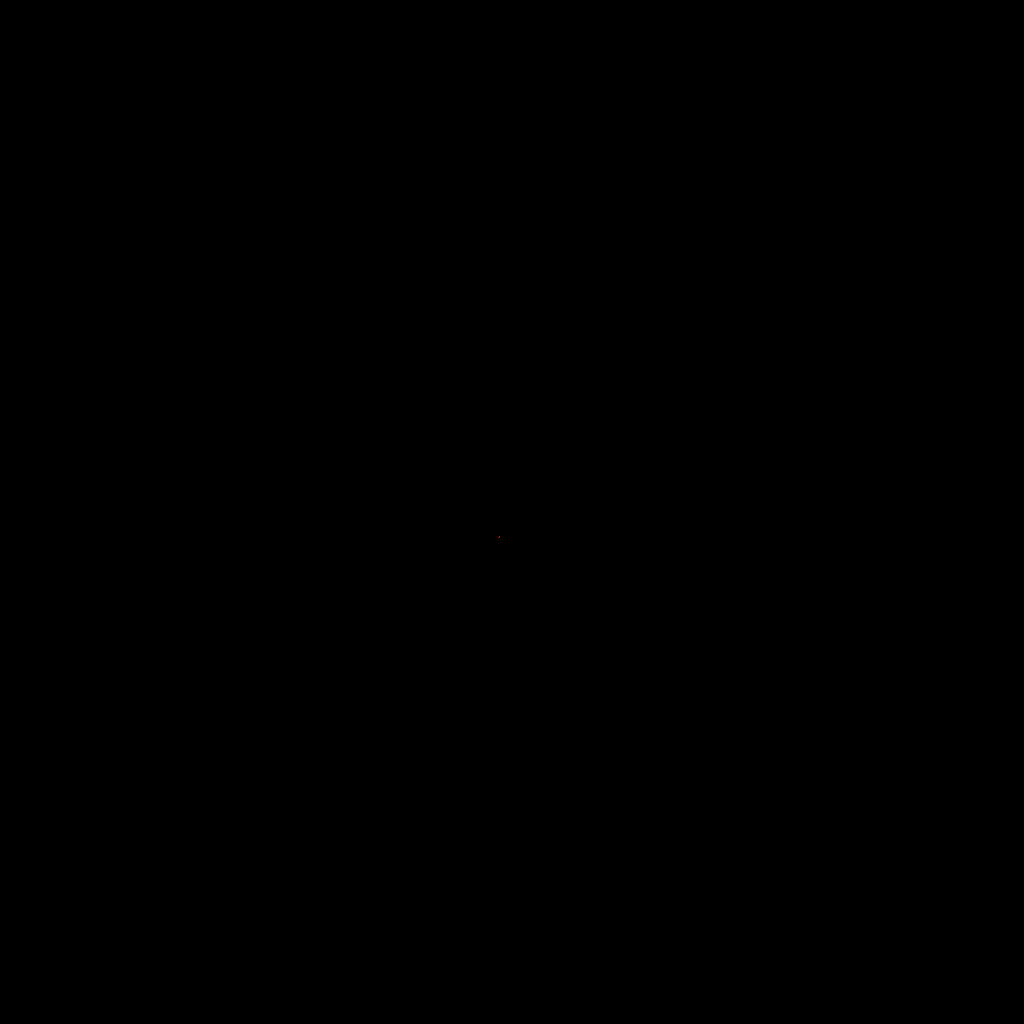

Supplement: Supplementary file 8 — Source data Fig. 6 [file 44319_2025_513_MOESM8_ESM.zip › Figure 6 Source Data/6E/DHAT ETO-/RAD51.tif]

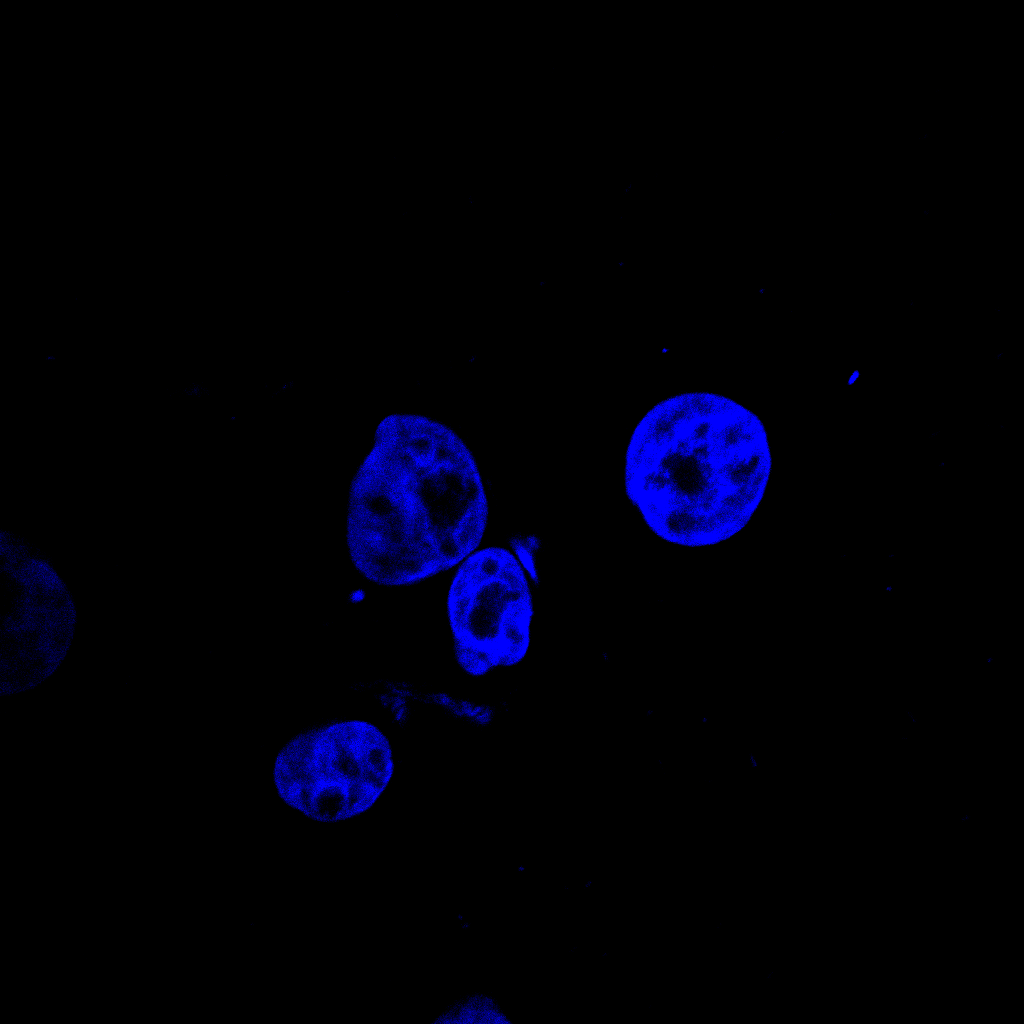

Supplement: Supplementary file 8 — Source data Fig. 6 [file 44319_2025_513_MOESM8_ESM.zip › Figure 6 Source Data/6E/EV ETO+/DAPI.tif]

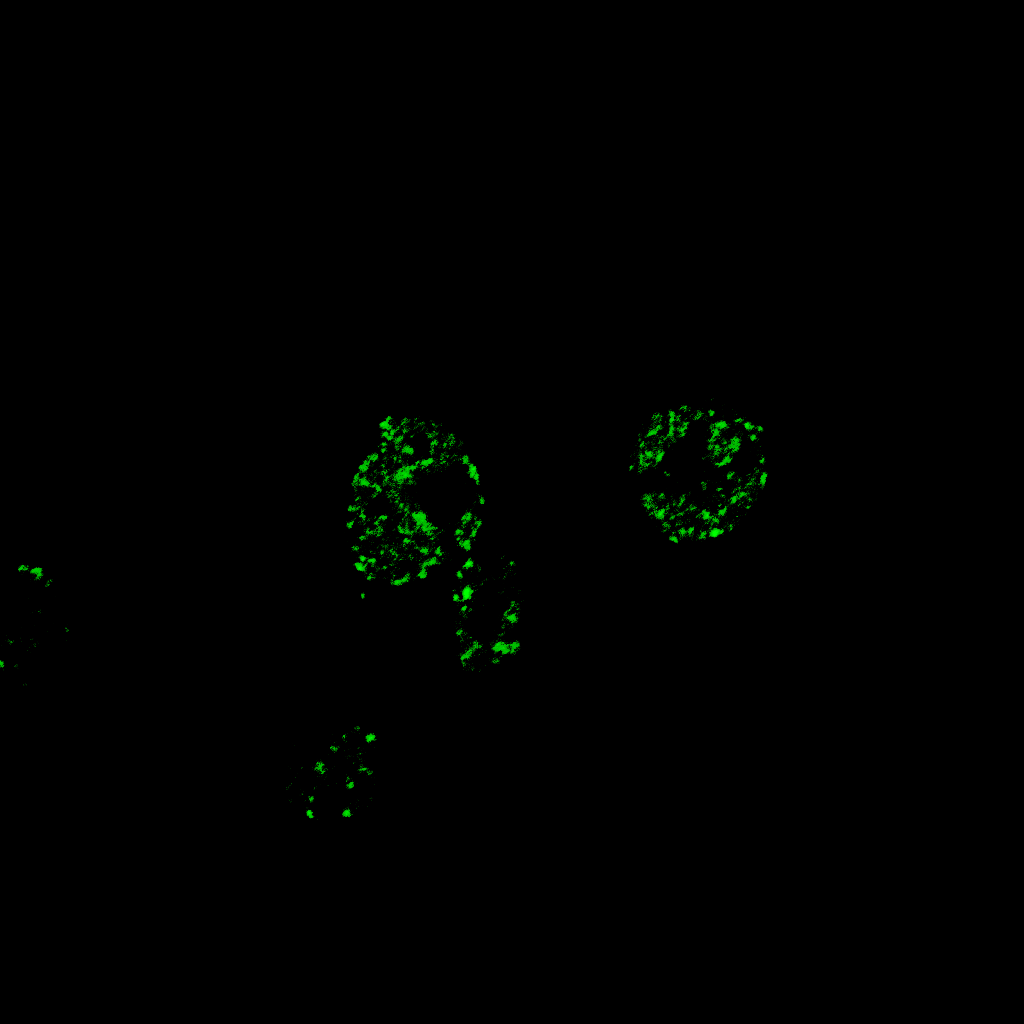

Supplement: Supplementary file 8 — Source data Fig. 6 [file 44319_2025_513_MOESM8_ESM.zip › Figure 6 Source Data/6E/EV ETO+/GH2AX.tif]

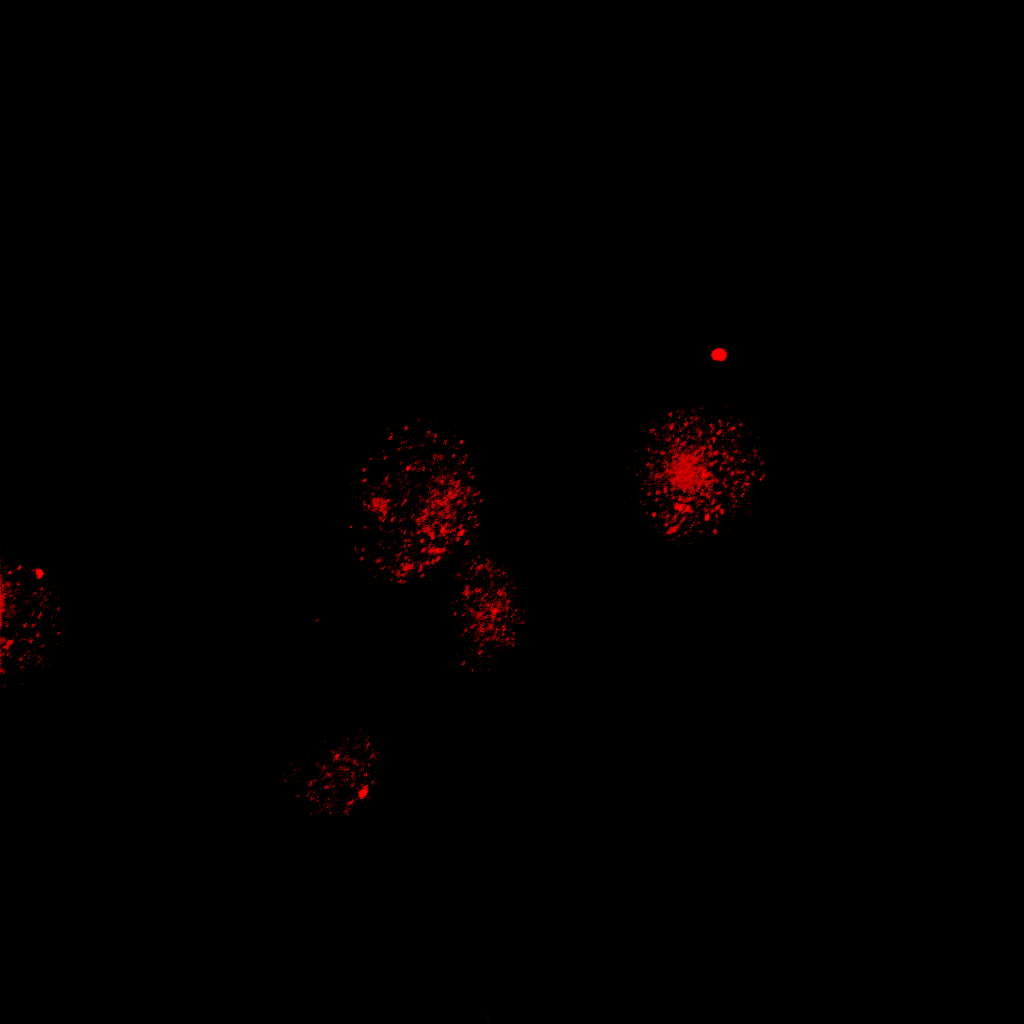

Supplement: Supplementary file 8 — Source data Fig. 6 [file 44319_2025_513_MOESM8_ESM.zip › Figure 6 Source Data/6E/EV ETO+/RAD51.tif]

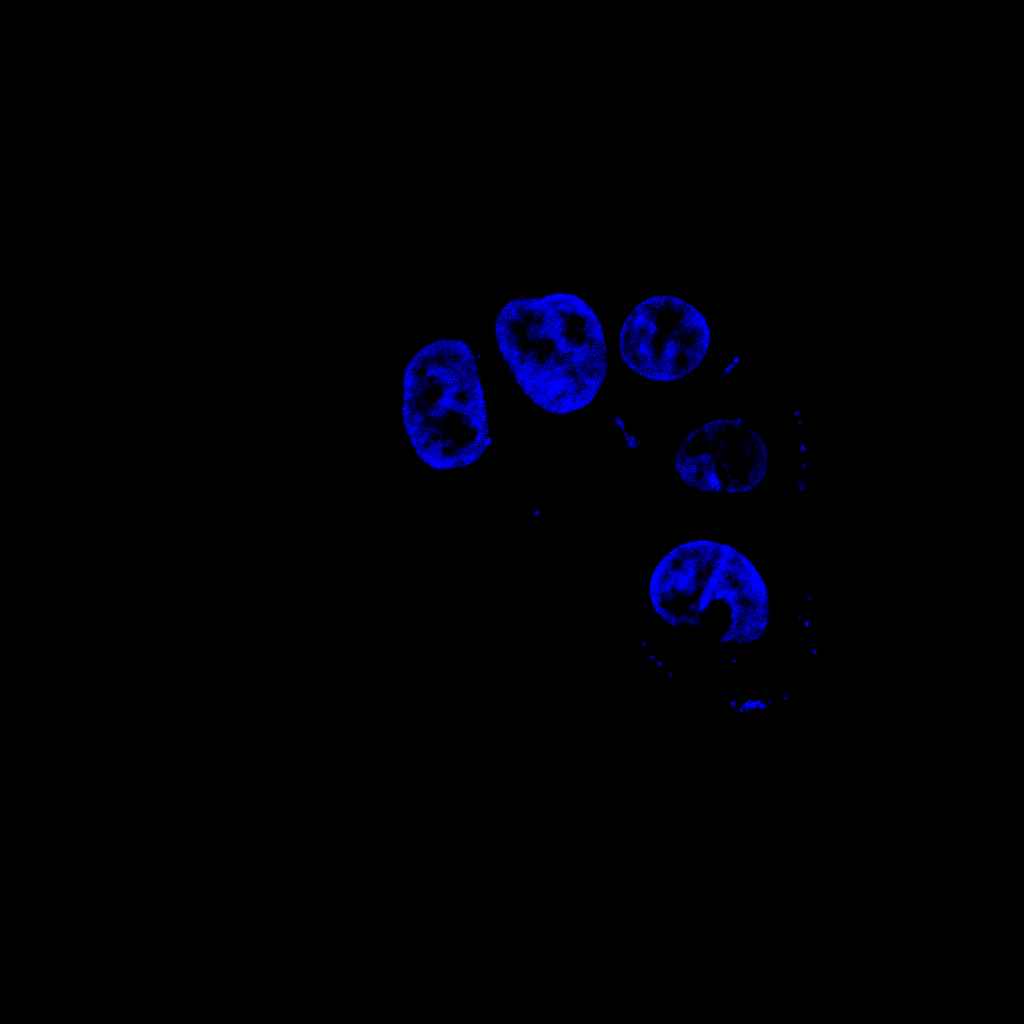

Supplement: Supplementary file 8 — Source data Fig. 6 [file 44319_2025_513_MOESM8_ESM.zip › Figure 6 Source Data/6E/EV ETO-/DAPI.tif]

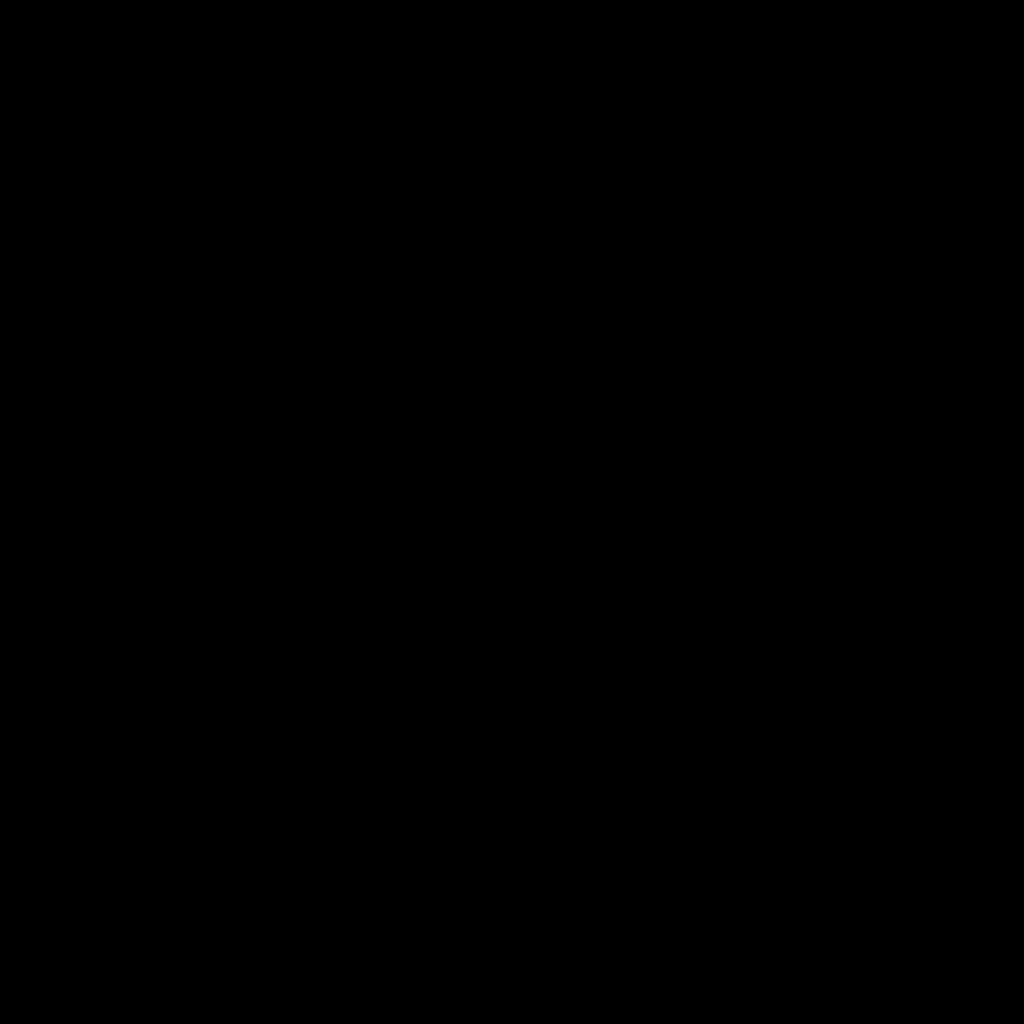

Supplement: Supplementary file 8 — Source data Fig. 6 [file 44319_2025_513_MOESM8_ESM.zip › Figure 6 Source Data/6E/EV ETO-/GH2AX.tif]

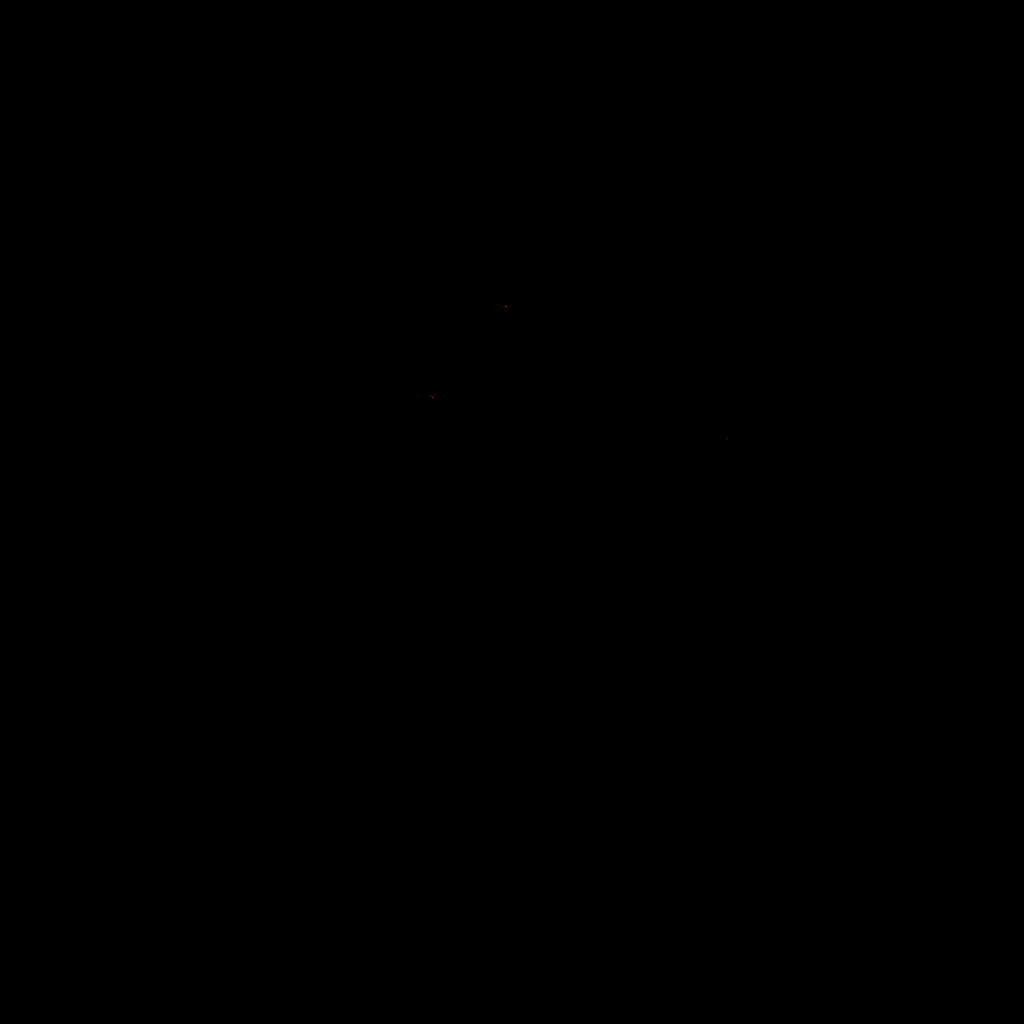

Supplement: Supplementary file 8 — Source data Fig. 6 [file 44319_2025_513_MOESM8_ESM.zip › Figure 6 Source Data/6E/EV ETO-/RAD51.tif]

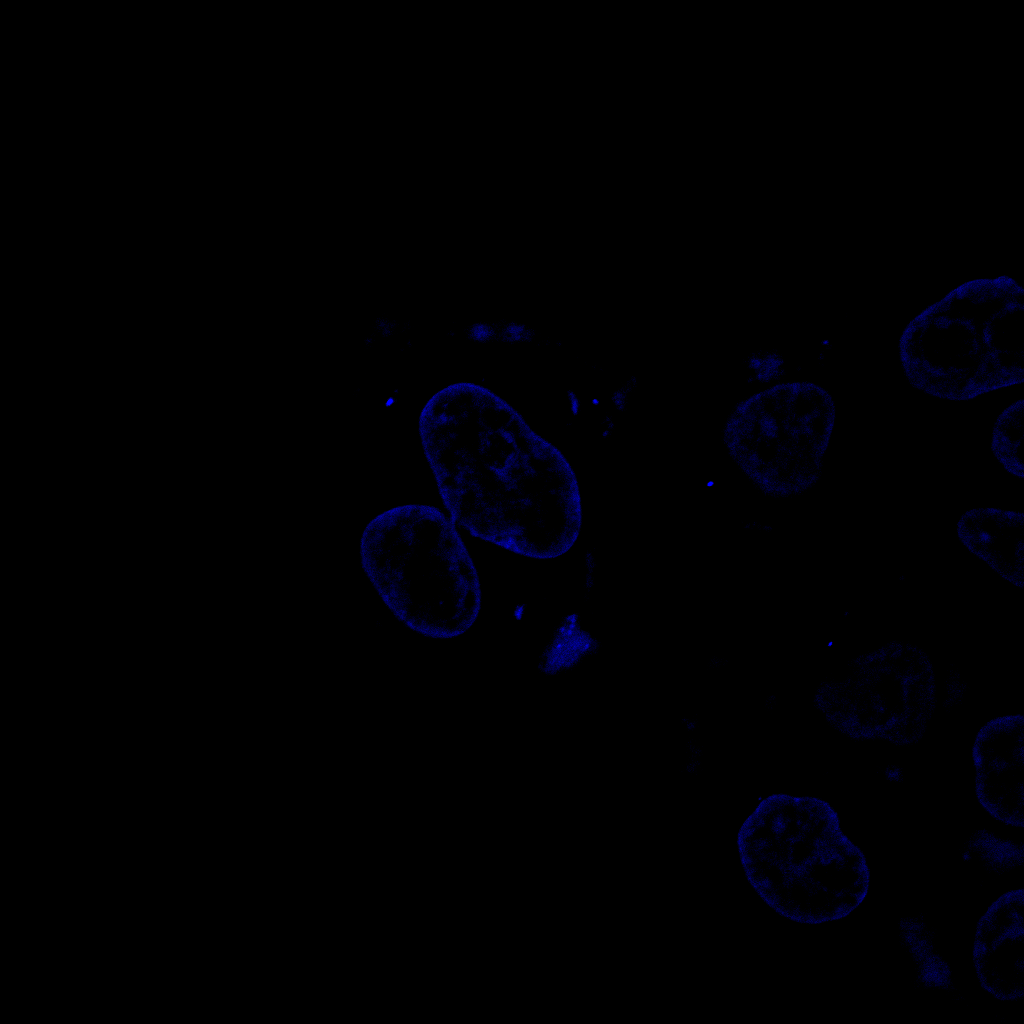

Supplement: Supplementary file 8 — Source data Fig. 6 [file 44319_2025_513_MOESM8_ESM.zip › Figure 6 Source Data/6E/PCAF ETO+/DAPI.tif]

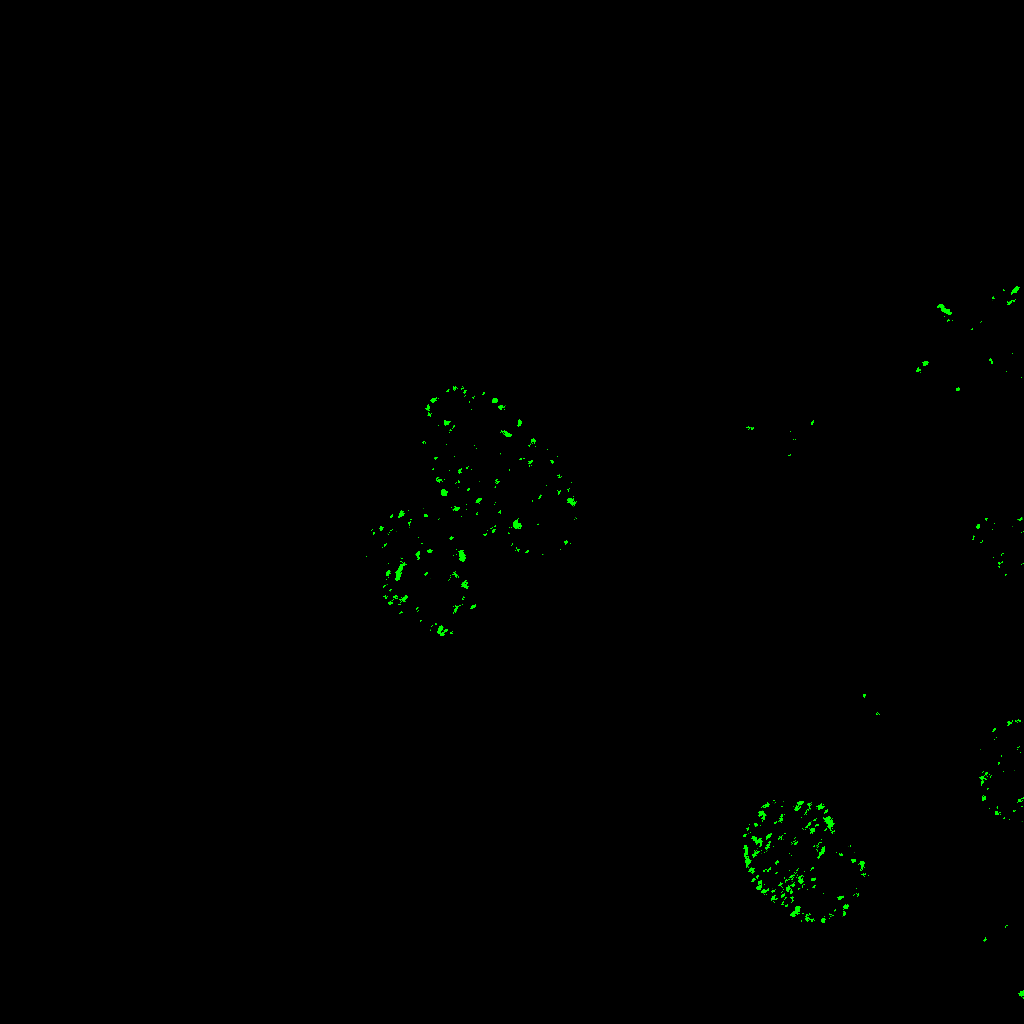

Supplement: Supplementary file 8 — Source data Fig. 6 [file 44319_2025_513_MOESM8_ESM.zip › Figure 6 Source Data/6E/PCAF ETO+/GH2AX.tif]

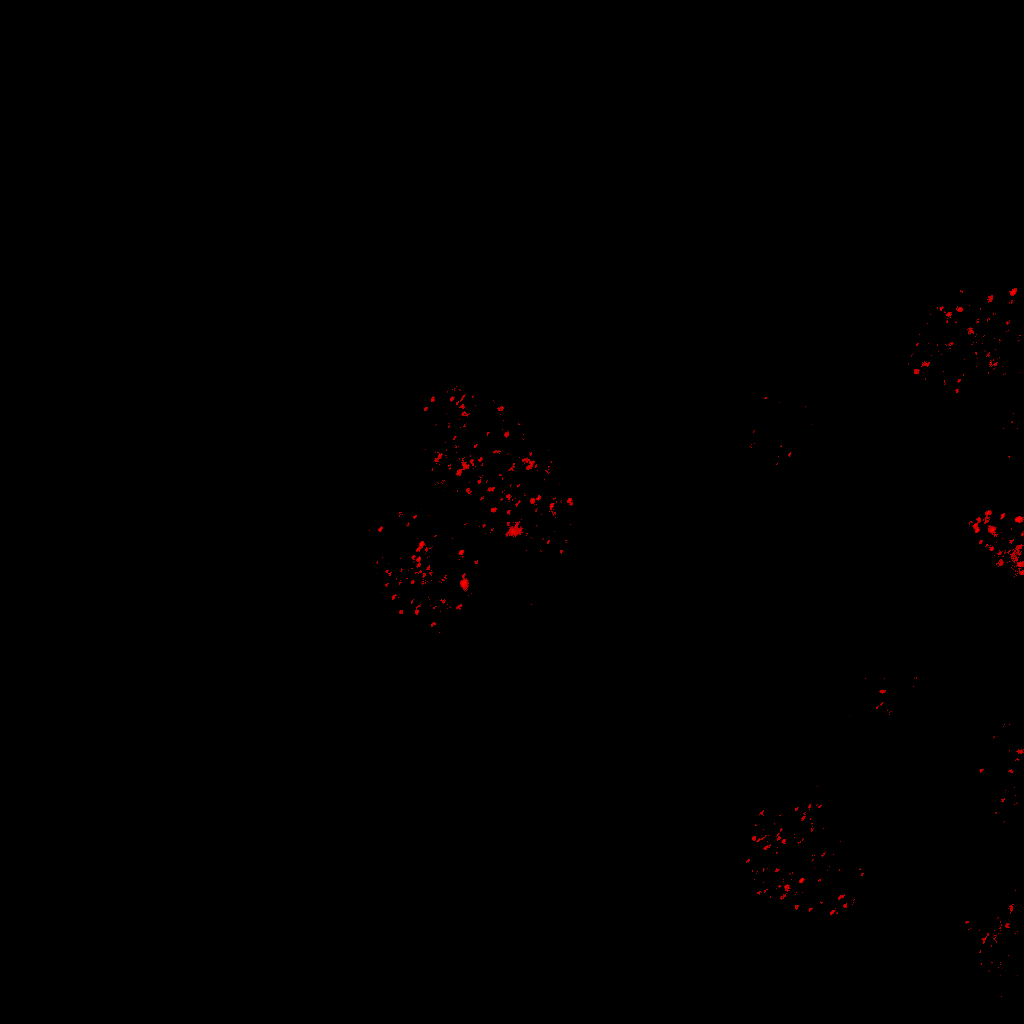

Supplement: Supplementary file 8 — Source data Fig. 6 [file 44319_2025_513_MOESM8_ESM.zip › Figure 6 Source Data/6E/PCAF ETO+/RAD51.tif]

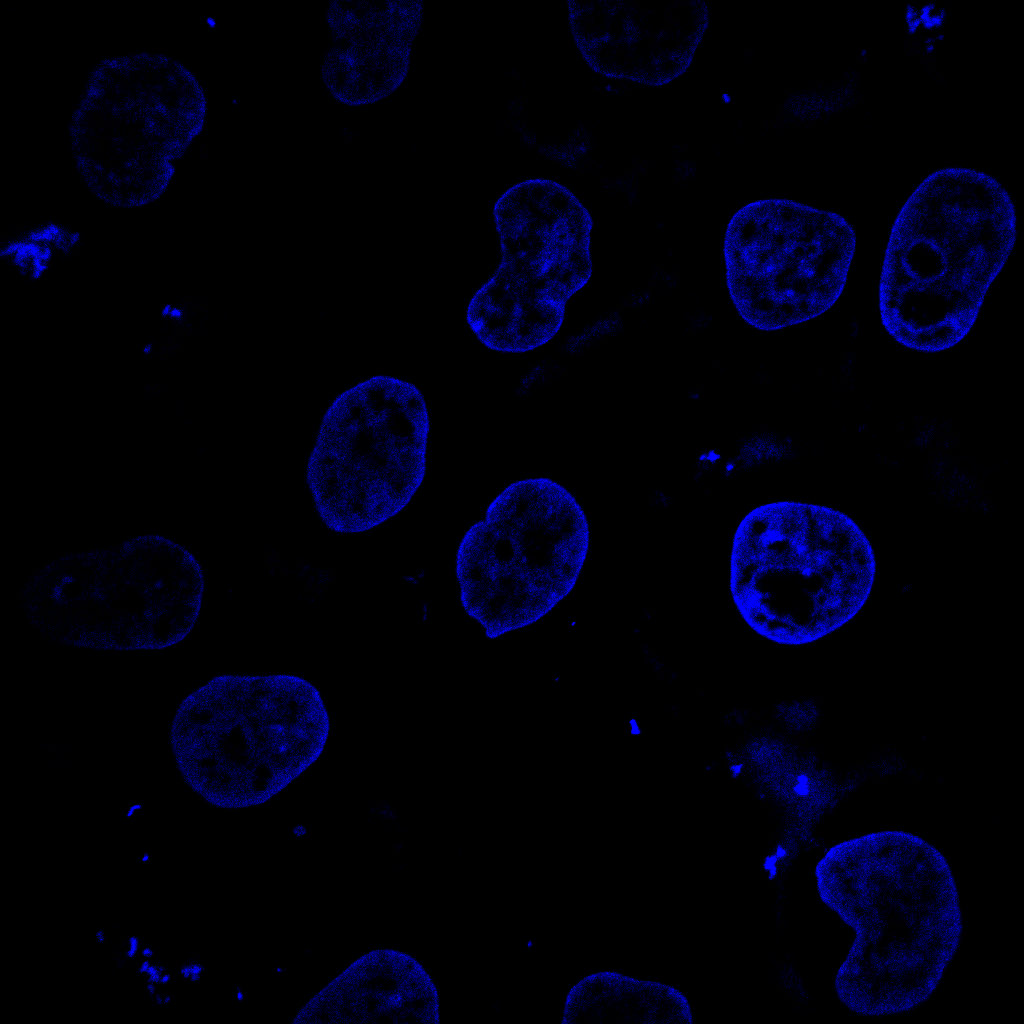

Supplement: Supplementary file 8 — Source data Fig. 6 [file 44319_2025_513_MOESM8_ESM.zip › Figure 6 Source Data/6E/PCAF ETO-/DAPI.tif]

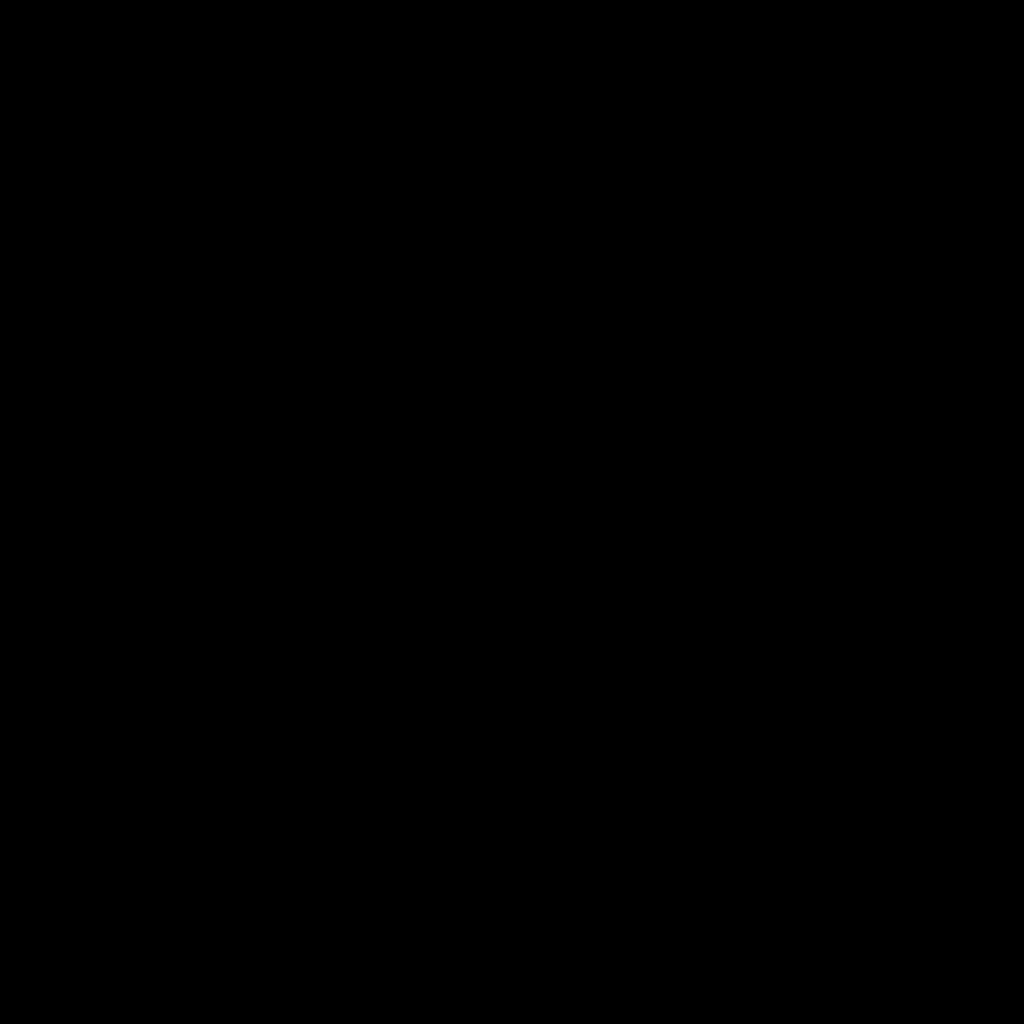

Supplement: Supplementary file 8 — Source data Fig. 6 [file 44319_2025_513_MOESM8_ESM.zip › Figure 6 Source Data/6E/PCAF ETO-/GH2AX.tif]

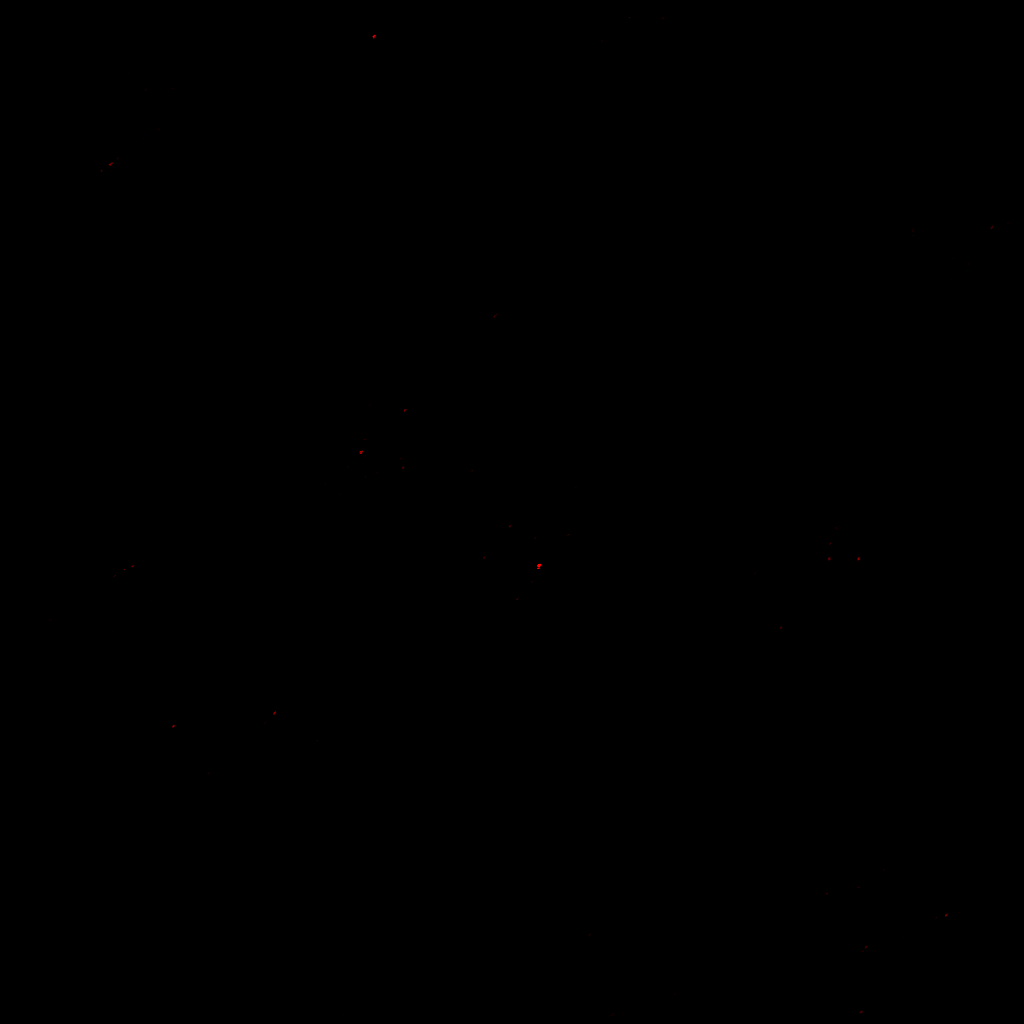

Supplement: Supplementary file 8 — Source data Fig. 6 [file 44319_2025_513_MOESM8_ESM.zip › Figure 6 Source Data/6E/PCAF ETO-/RAD51.tif]

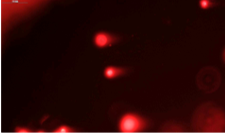

Supplement: Supplementary file 8 — Source data Fig. 6 [file 44319_2025_513_MOESM8_ESM.zip › Figure 6 Source Data/6F/DHAT ETO+.tif]

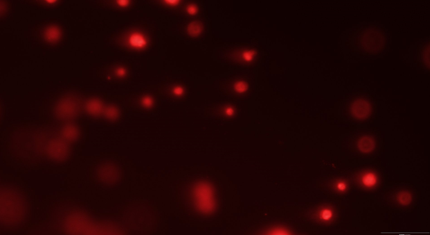

Supplement: Supplementary file 8 — Source data Fig. 6 [file 44319_2025_513_MOESM8_ESM.zip › Figure 6 Source Data/6F/DHAT ETO-.tif]

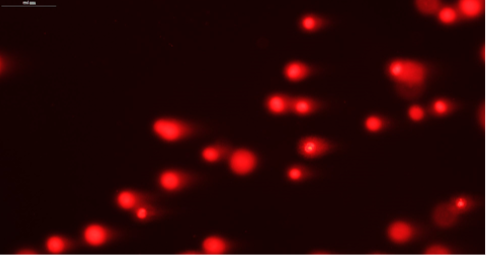

Supplement: Supplementary file 8 — Source data Fig. 6 [file 44319_2025_513_MOESM8_ESM.zip › Figure 6 Source Data/6F/EV ETO+.tif]

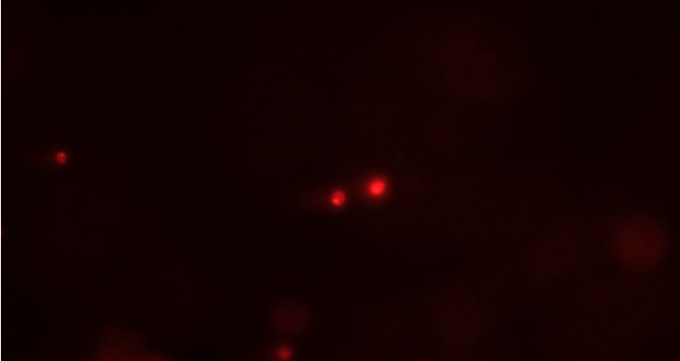

Supplement: Supplementary file 8 — Source data Fig. 6 [file 44319_2025_513_MOESM8_ESM.zip › Figure 6 Source Data/6F/EV ETO-.tif]

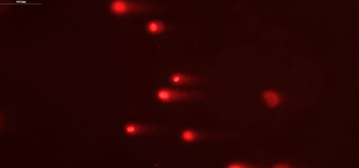

Supplement: Supplementary file 8 — Source data Fig. 6 [file 44319_2025_513_MOESM8_ESM.zip › Figure 6 Source Data/6F/WT ETO+.tif]

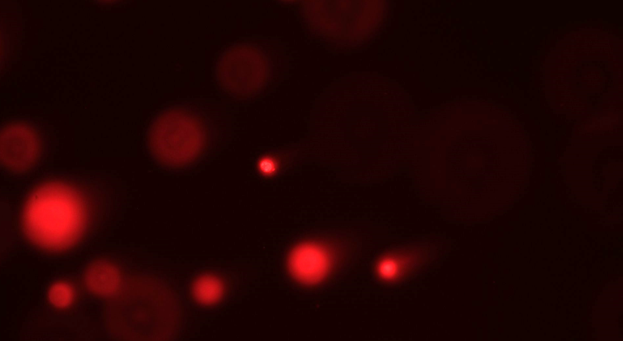

Supplement: Supplementary file 8 — Source data Fig. 6 [file 44319_2025_513_MOESM8_ESM.zip › Figure 6 Source Data/6F/WT ETO-.tif]

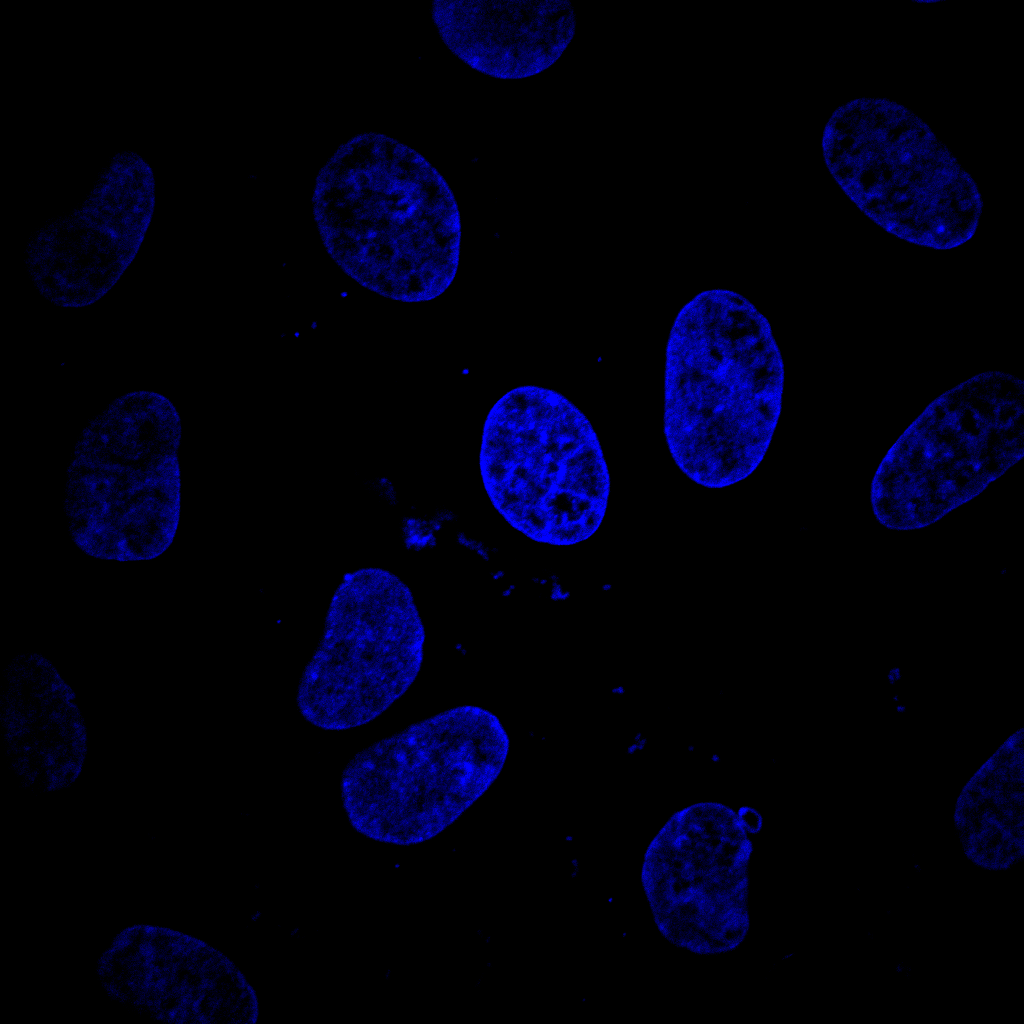

Supplement: Supplementary file 9 — Expanded View Figure and Appendix source data [file 44319_2025_513_MOESM9_ESM.zip › Expanded View Figure and Appendix source data/Appendix source data/S1 F/DMSO ETO+/DAPI.tif]

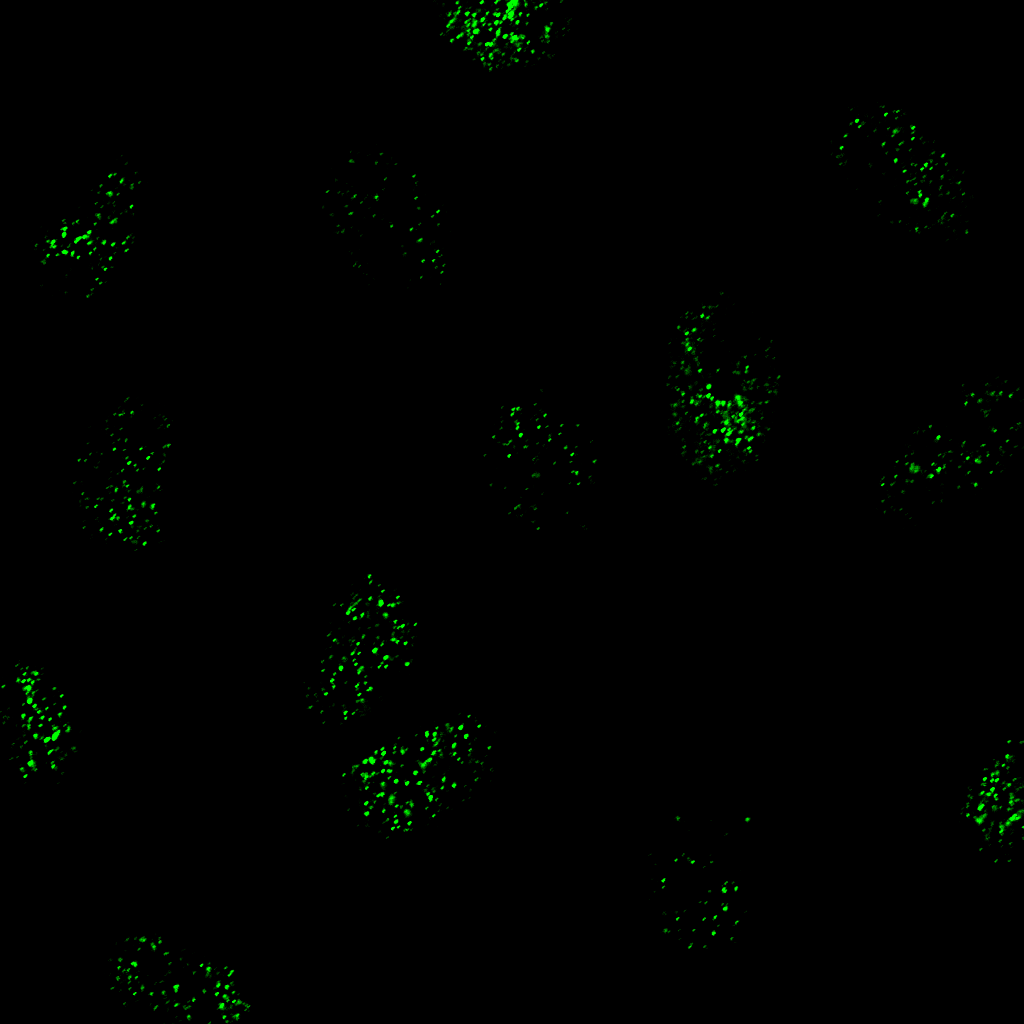

Supplement: Supplementary file 9 — Expanded View Figure and Appendix source data [file 44319_2025_513_MOESM9_ESM.zip › Expanded View Figure and Appendix source data/Appendix source data/S1 F/DMSO ETO+/GH2AX.tif]

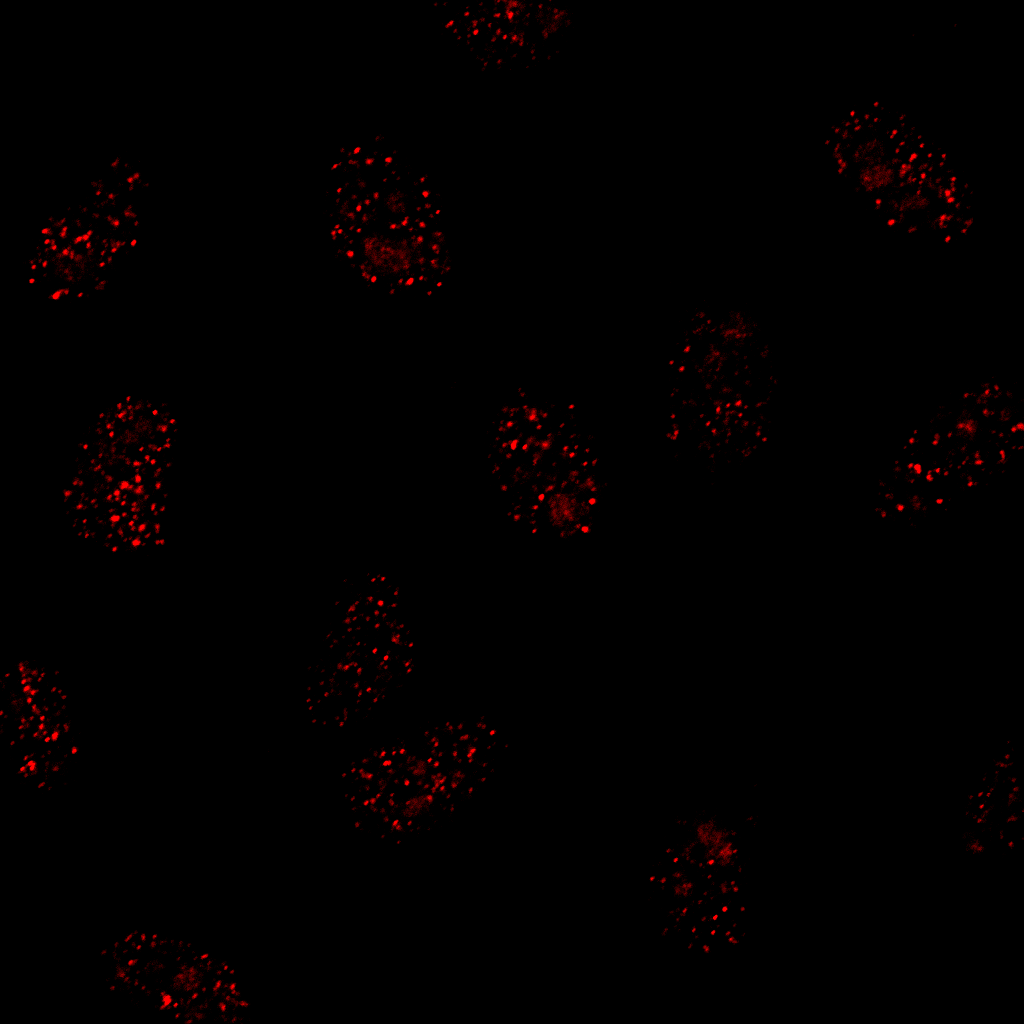

Supplement: Supplementary file 9 — Expanded View Figure and Appendix source data [file 44319_2025_513_MOESM9_ESM.zip › Expanded View Figure and Appendix source data/Appendix source data/S1 F/DMSO ETO+/RAD51.tif]

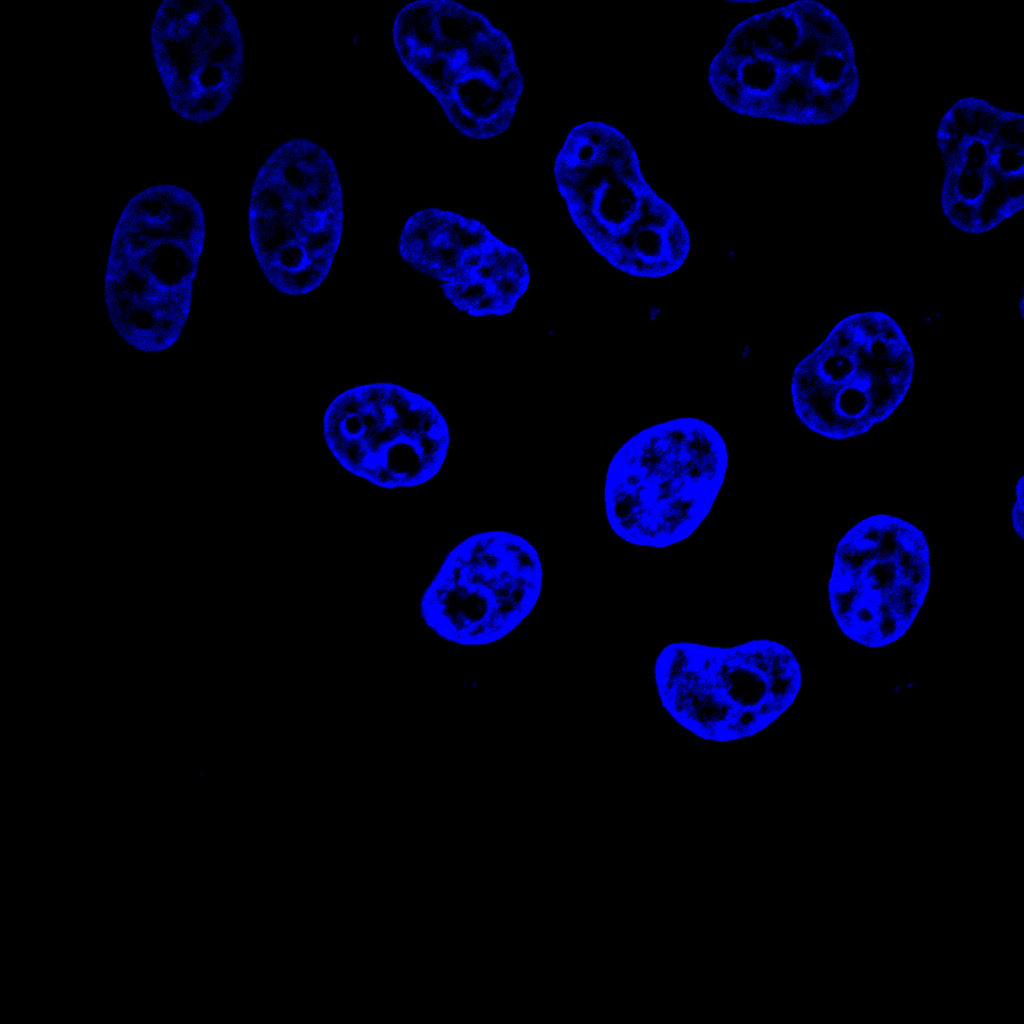

Supplement: Supplementary file 9 — Expanded View Figure and Appendix source data [file 44319_2025_513_MOESM9_ESM.zip › Expanded View Figure and Appendix source data/Appendix source data/S1 F/DMSO ETO-/DAPI.tif]

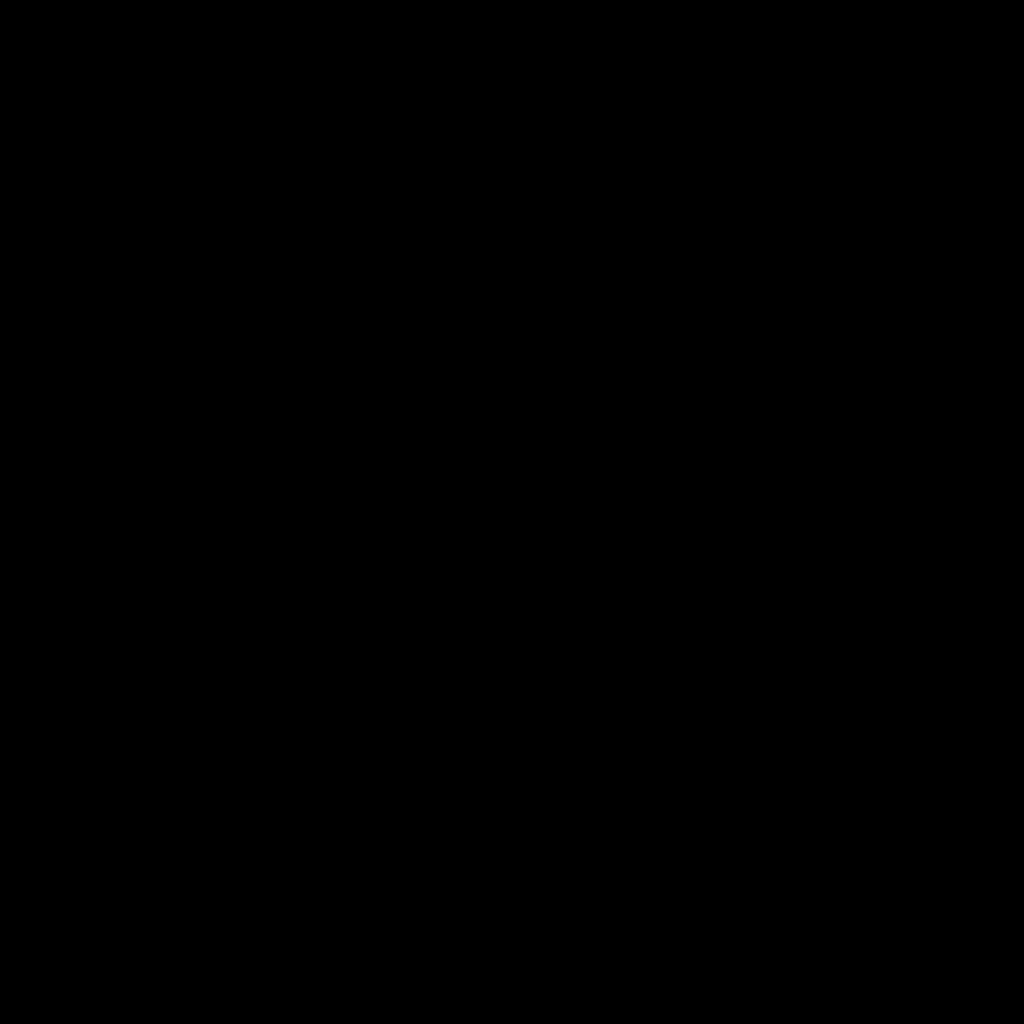

Supplement: Supplementary file 9 — Expanded View Figure and Appendix source data [file 44319_2025_513_MOESM9_ESM.zip › Expanded View Figure and Appendix source data/Appendix source data/S1 F/DMSO ETO-/GH2AX.tif]

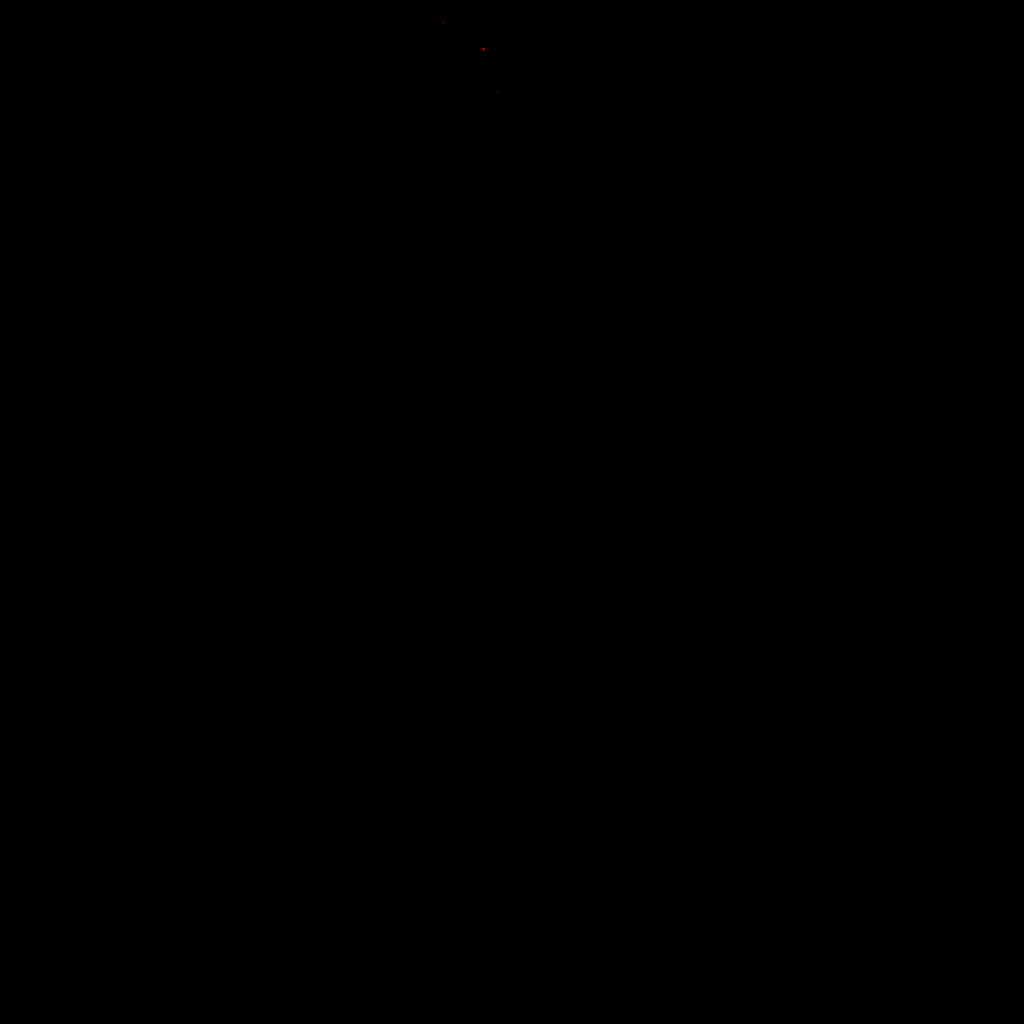

Supplement: Supplementary file 9 — Expanded View Figure and Appendix source data [file 44319_2025_513_MOESM9_ESM.zip › Expanded View Figure and Appendix source data/Appendix source data/S1 F/DMSO ETO-/RAD51.tif]

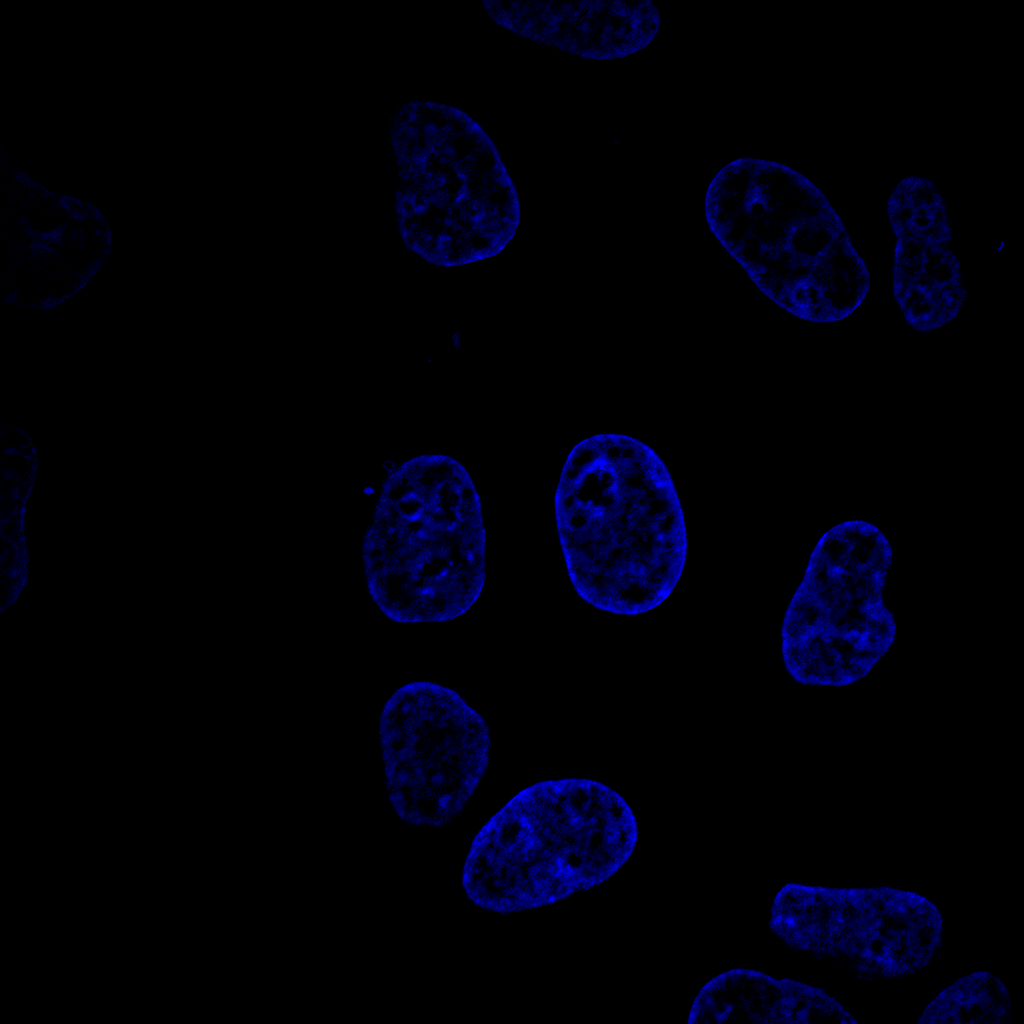

Supplement: Supplementary file 9 — Expanded View Figure and Appendix source data [file 44319_2025_513_MOESM9_ESM.zip › Expanded View Figure and Appendix source data/Appendix source data/S1 F/PCAFi ETO+/DAPI.tif]

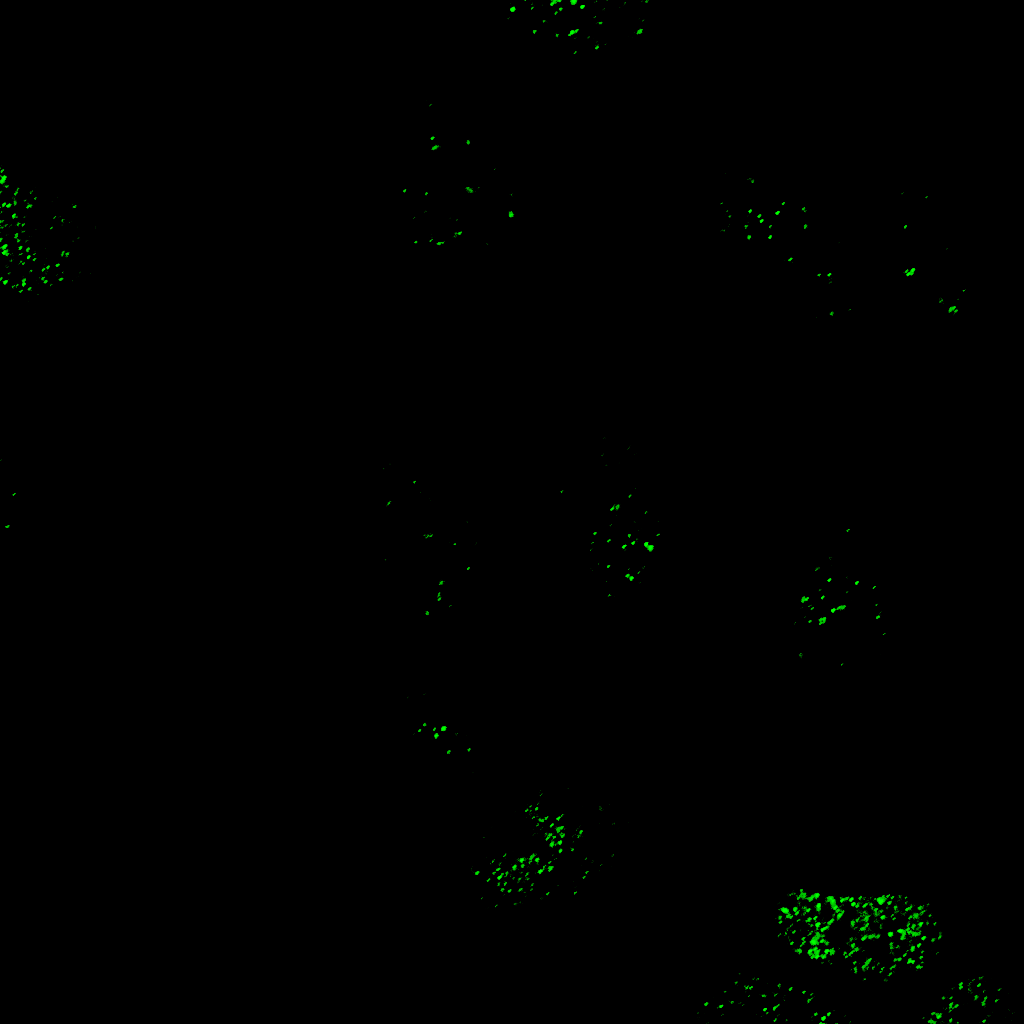

Supplement: Supplementary file 9 — Expanded View Figure and Appendix source data [file 44319_2025_513_MOESM9_ESM.zip › Expanded View Figure and Appendix source data/Appendix source data/S1 F/PCAFi ETO+/GH2AX.tif]

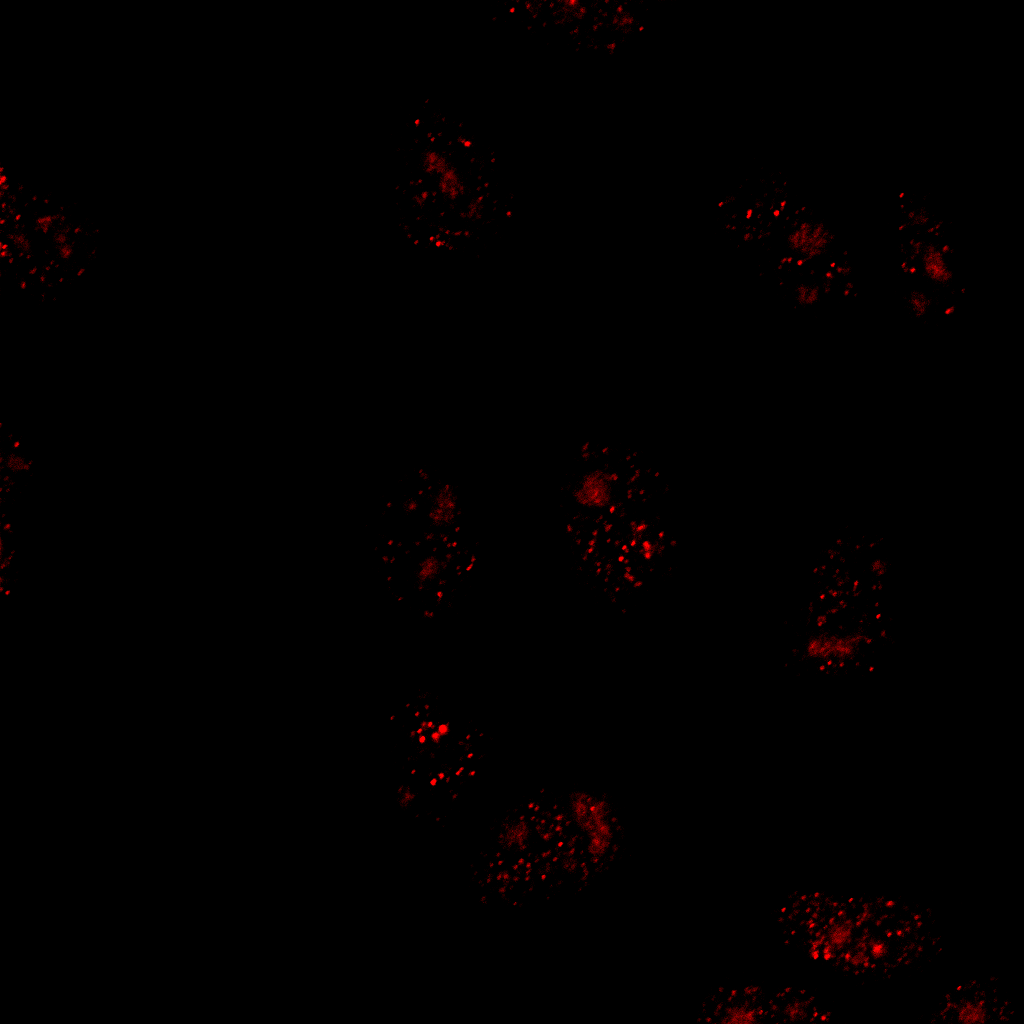

Supplement: Supplementary file 9 — Expanded View Figure and Appendix source data [file 44319_2025_513_MOESM9_ESM.zip › Expanded View Figure and Appendix source data/Appendix source data/S1 F/PCAFi ETO+/RAD51.tif]

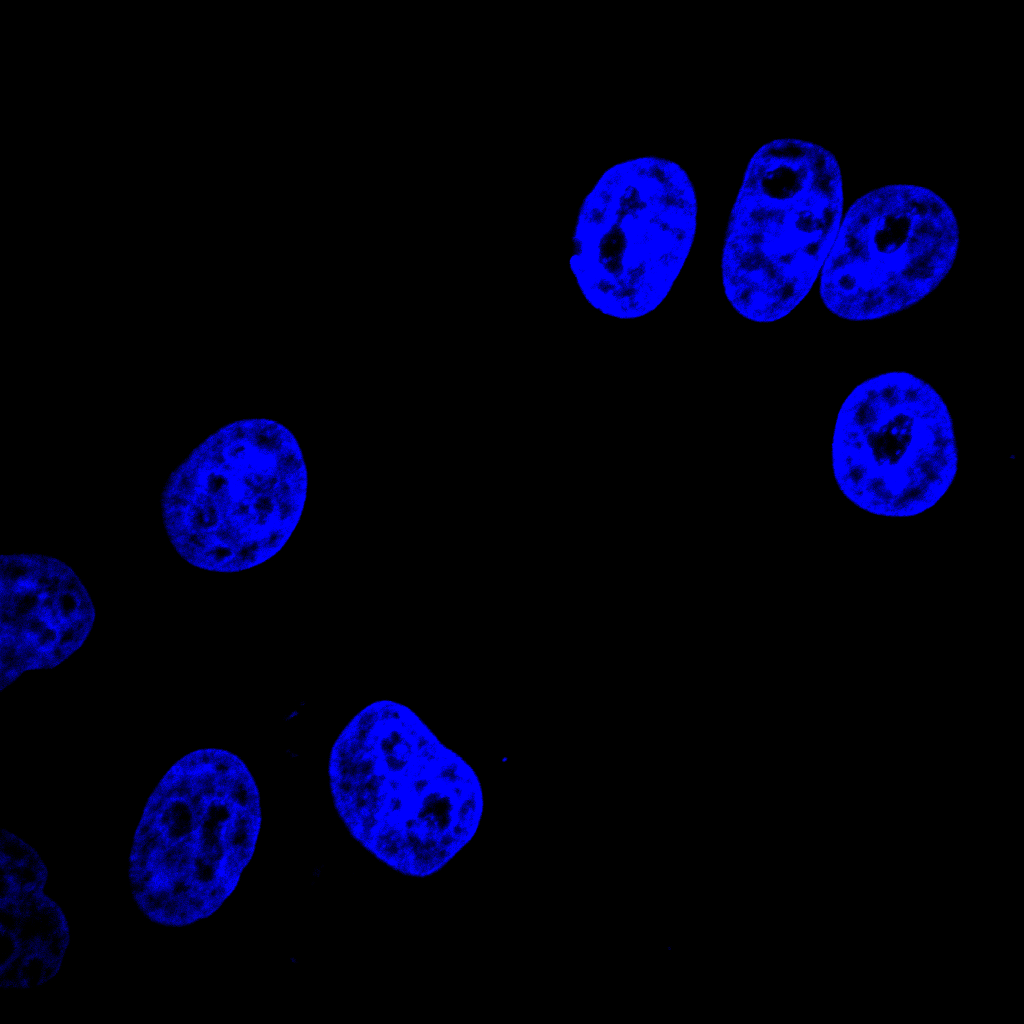

Supplement: Supplementary file 9 — Expanded View Figure and Appendix source data [file 44319_2025_513_MOESM9_ESM.zip › Expanded View Figure and Appendix source data/Appendix source data/S1 F/PCAFi ETO-/DAPI.tif]

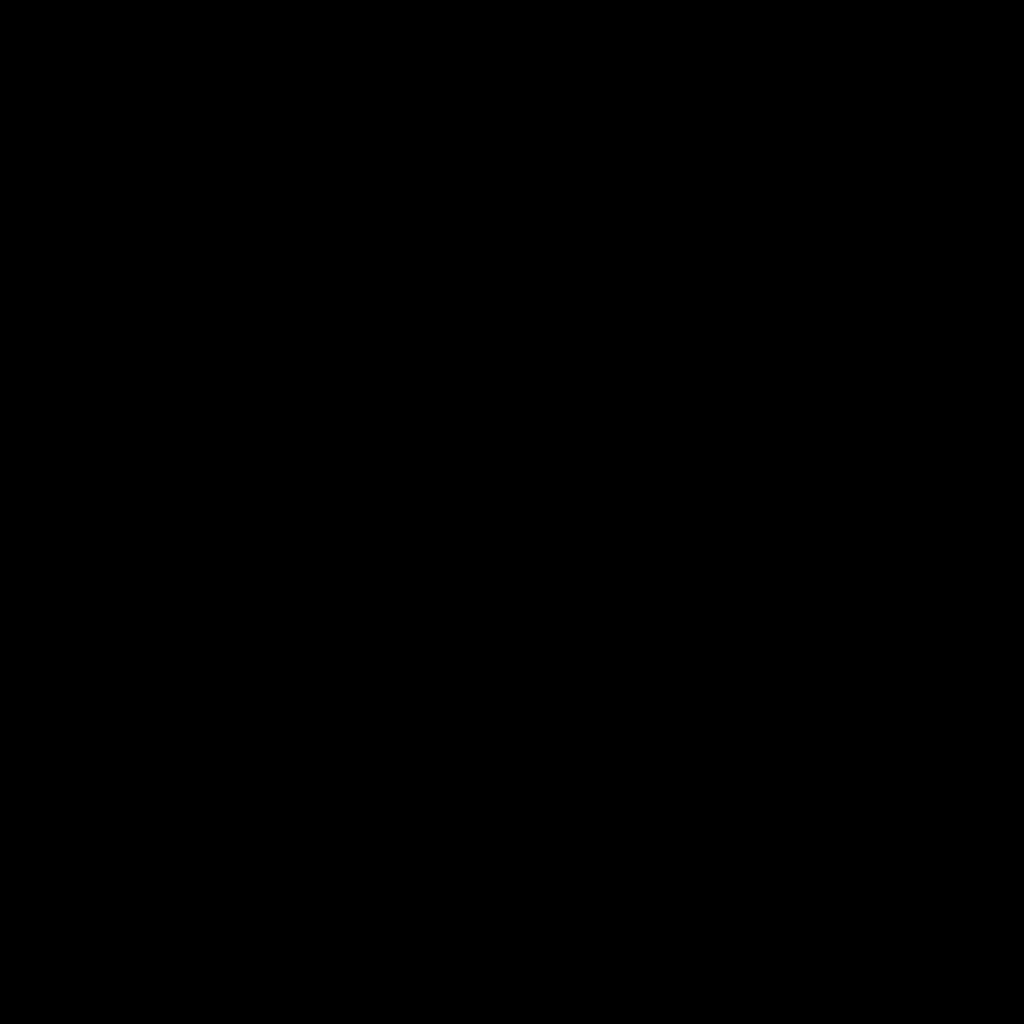

Supplement: Supplementary file 9 — Expanded View Figure and Appendix source data [file 44319_2025_513_MOESM9_ESM.zip › Expanded View Figure and Appendix source data/Appendix source data/S1 F/PCAFi ETO-/GH2AX.tif]
