# Supplementary figures and images for: PCAF-mediated acetylation regulates RAD51 dynamic localization on chromatin during HR repair (part 4 of 4)
Source: EMBO Rep. 2025 Jul 15;26(16):4100–23. doi: 10.1038/s44319-025-00513-6 (PMC12373954; doi:10.1038/s44319-025-00513-6)

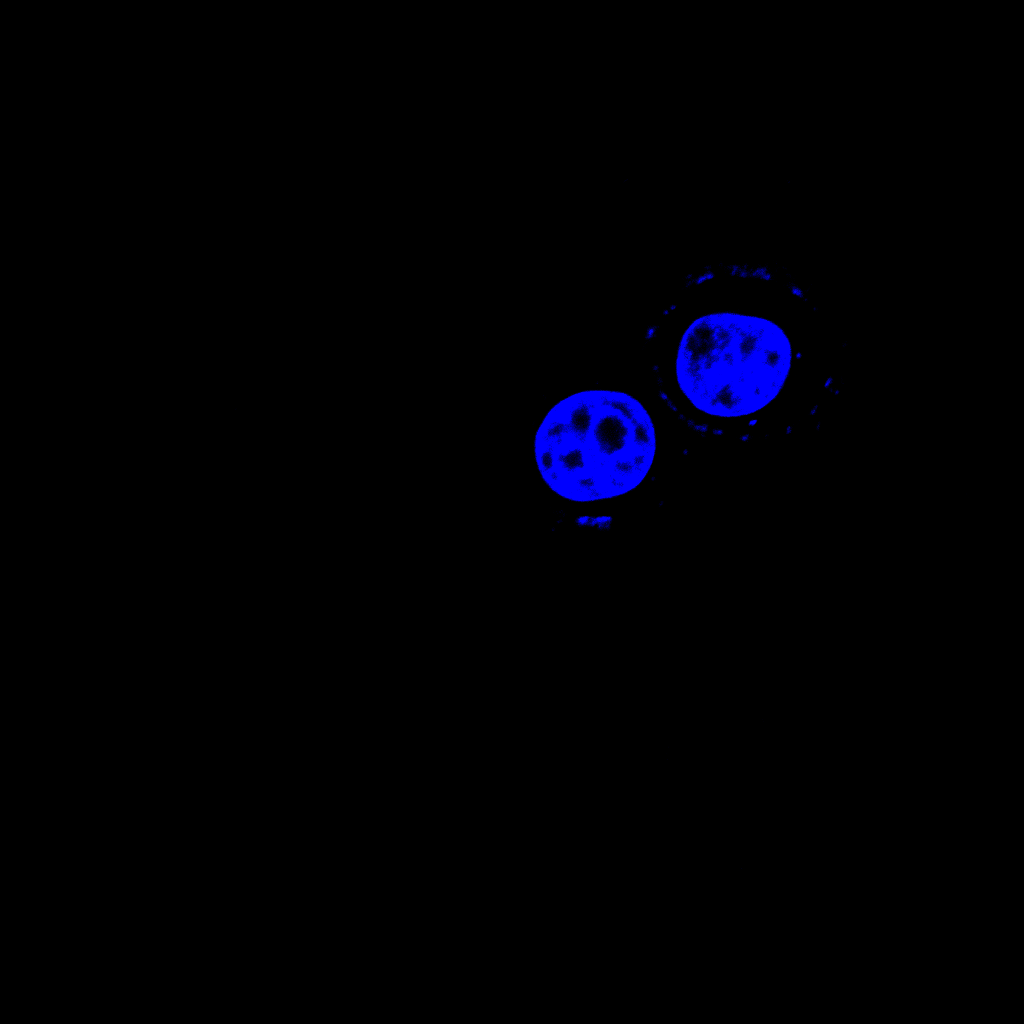

Supplement: Supplementary file 9 — Expanded View Figure and Appendix source data [file 44319_2025_513_MOESM9_ESM.zip › Expanded View Figure and Appendix source data/Expanded View Figure 5/EV 5D/sgPCAF 1 h/DAPI.tif]

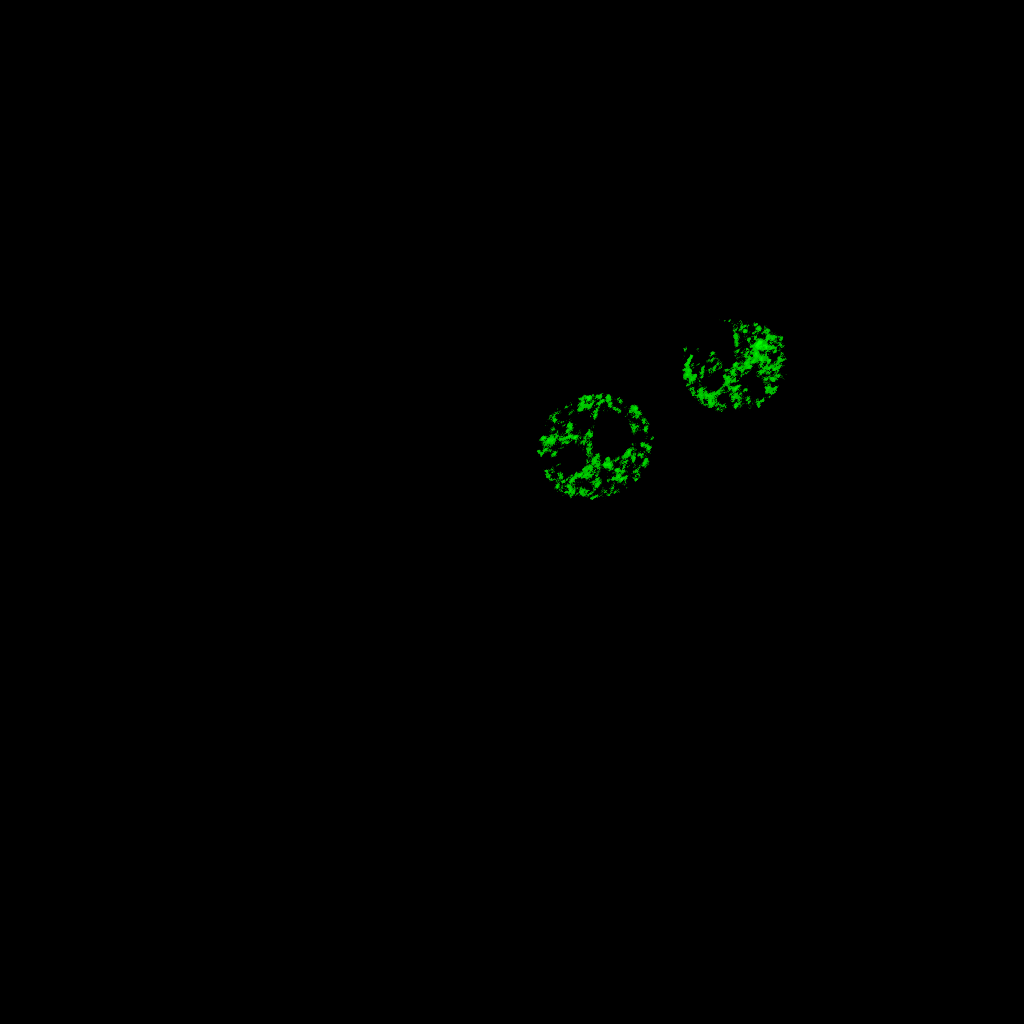

Supplement: Supplementary file 9 — Expanded View Figure and Appendix source data [file 44319_2025_513_MOESM9_ESM.zip › Expanded View Figure and Appendix source data/Expanded View Figure 5/EV 5D/sgPCAF 1 h/GH2AX.tif]

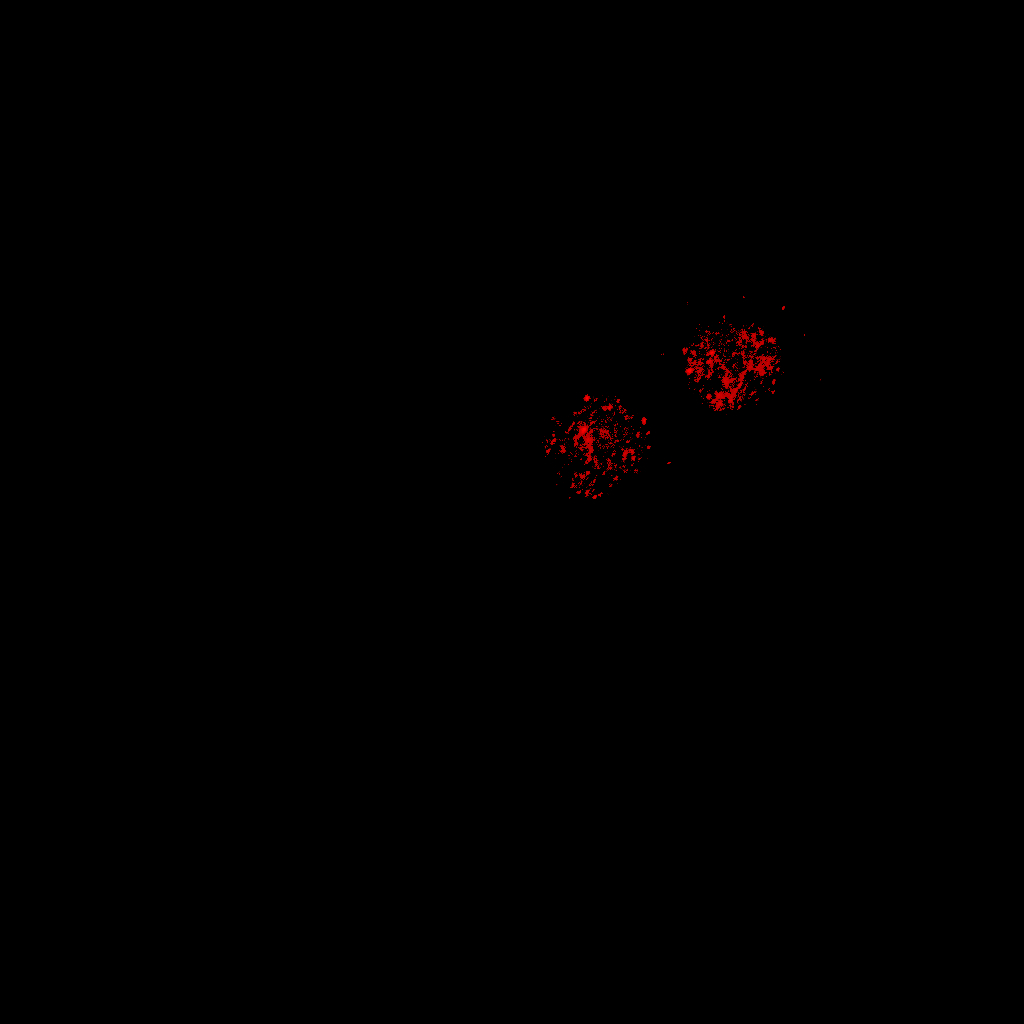

Supplement: Supplementary file 9 — Expanded View Figure and Appendix source data [file 44319_2025_513_MOESM9_ESM.zip › Expanded View Figure and Appendix source data/Expanded View Figure 5/EV 5D/sgPCAF 1 h/RAD51.tif]

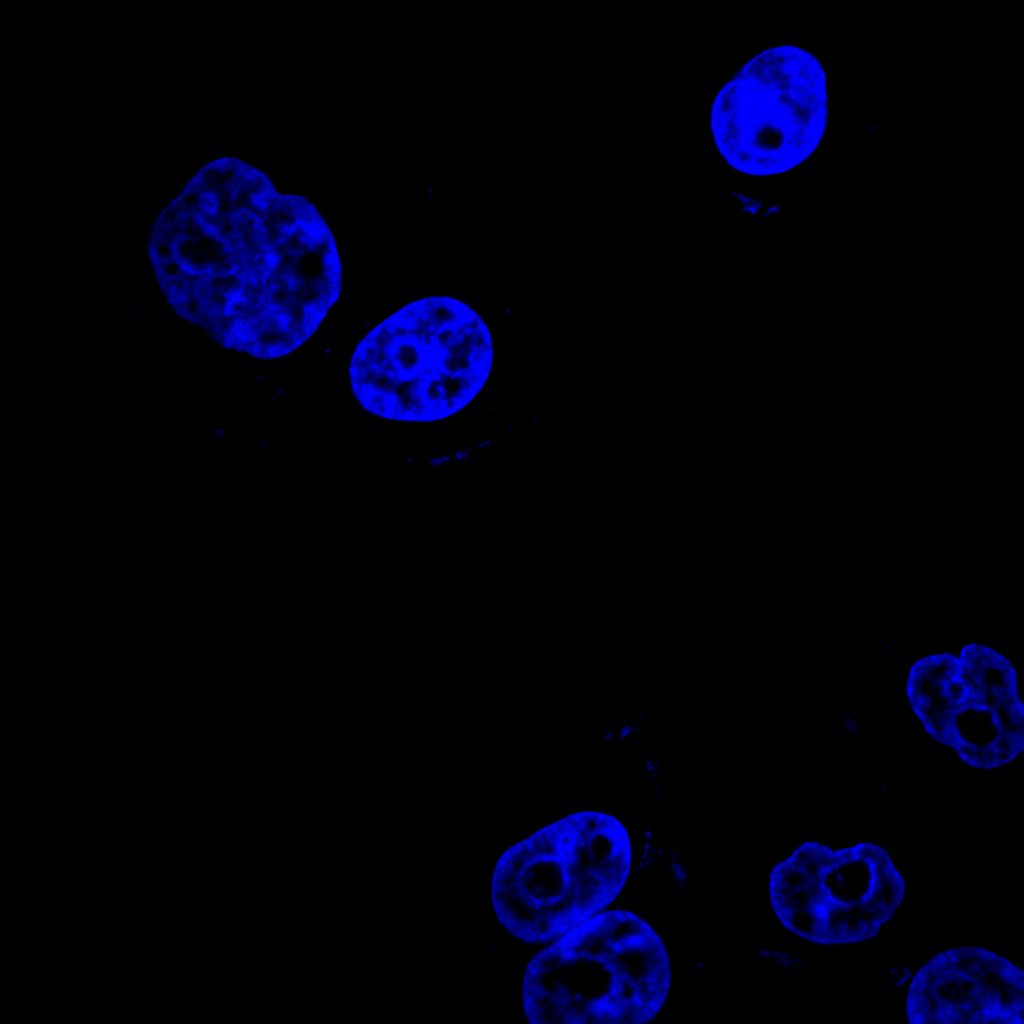

Supplement: Supplementary file 9 — Expanded View Figure and Appendix source data [file 44319_2025_513_MOESM9_ESM.zip › Expanded View Figure and Appendix source data/Expanded View Figure 5/EV 5D/sgPCAF 4 h/DAPI.tif]

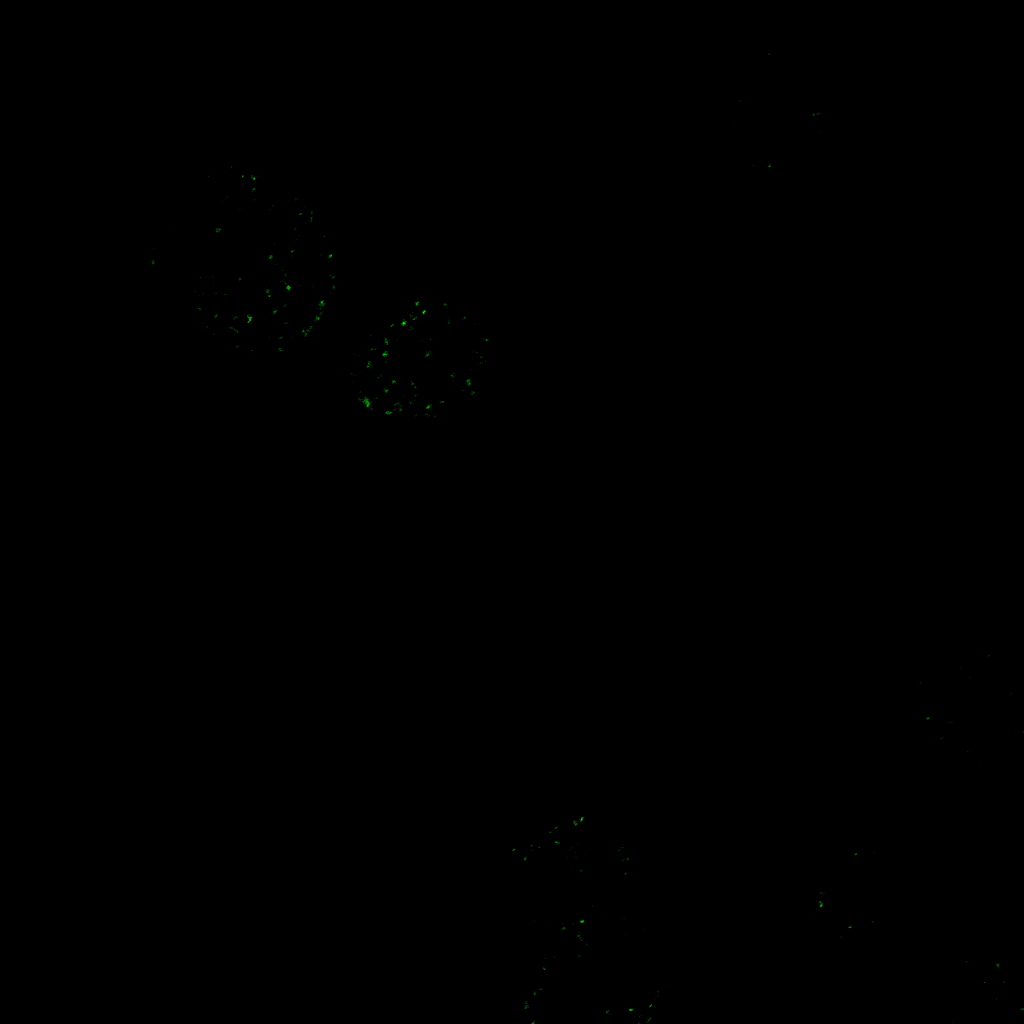

Supplement: Supplementary file 9 — Expanded View Figure and Appendix source data [file 44319_2025_513_MOESM9_ESM.zip › Expanded View Figure and Appendix source data/Expanded View Figure 5/EV 5D/sgPCAF 4 h/GH2AX.tif]

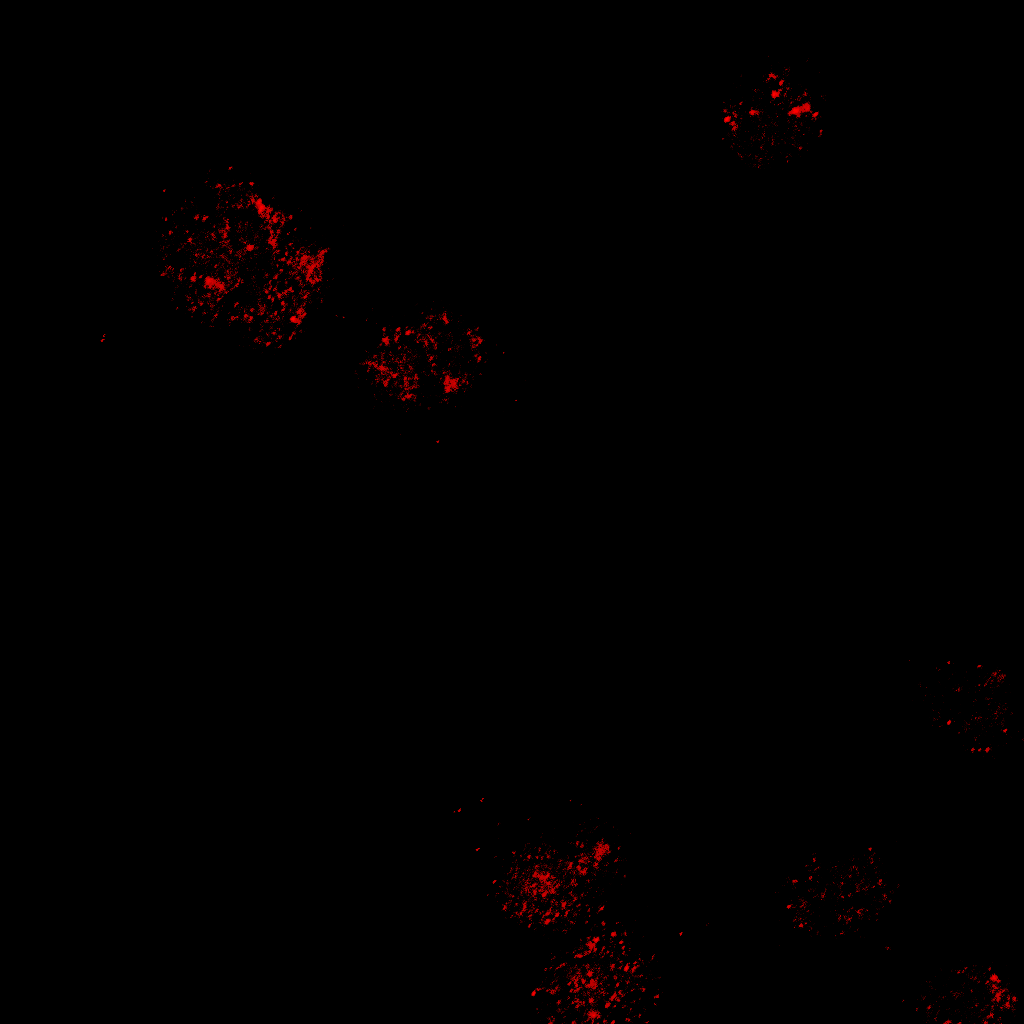

Supplement: Supplementary file 9 — Expanded View Figure and Appendix source data [file 44319_2025_513_MOESM9_ESM.zip › Expanded View Figure and Appendix source data/Expanded View Figure 5/EV 5D/sgPCAF 4 h/RAD51.tif]

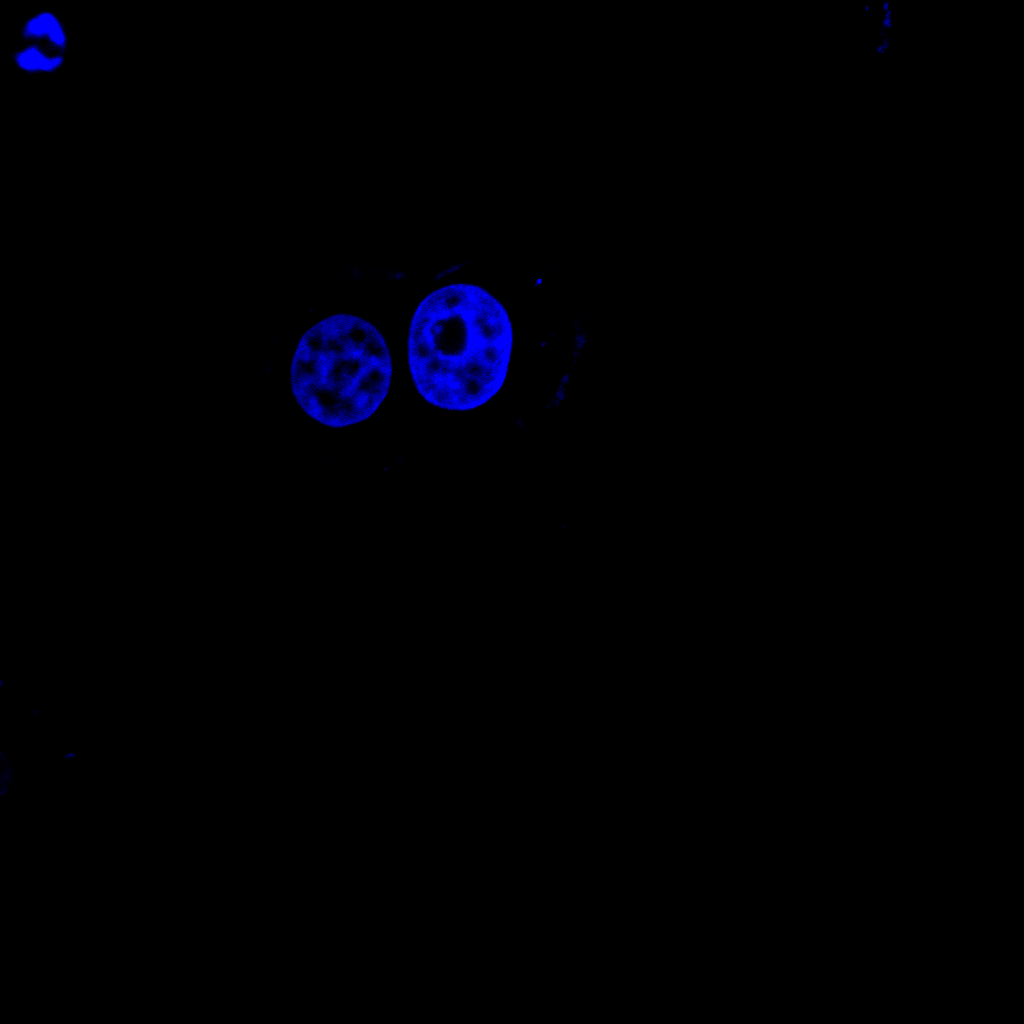

Supplement: Supplementary file 9 — Expanded View Figure and Appendix source data [file 44319_2025_513_MOESM9_ESM.zip › Expanded View Figure and Appendix source data/Expanded View Figure 5/EV 5D/sgPCAF 8 h/DAPI.tif]

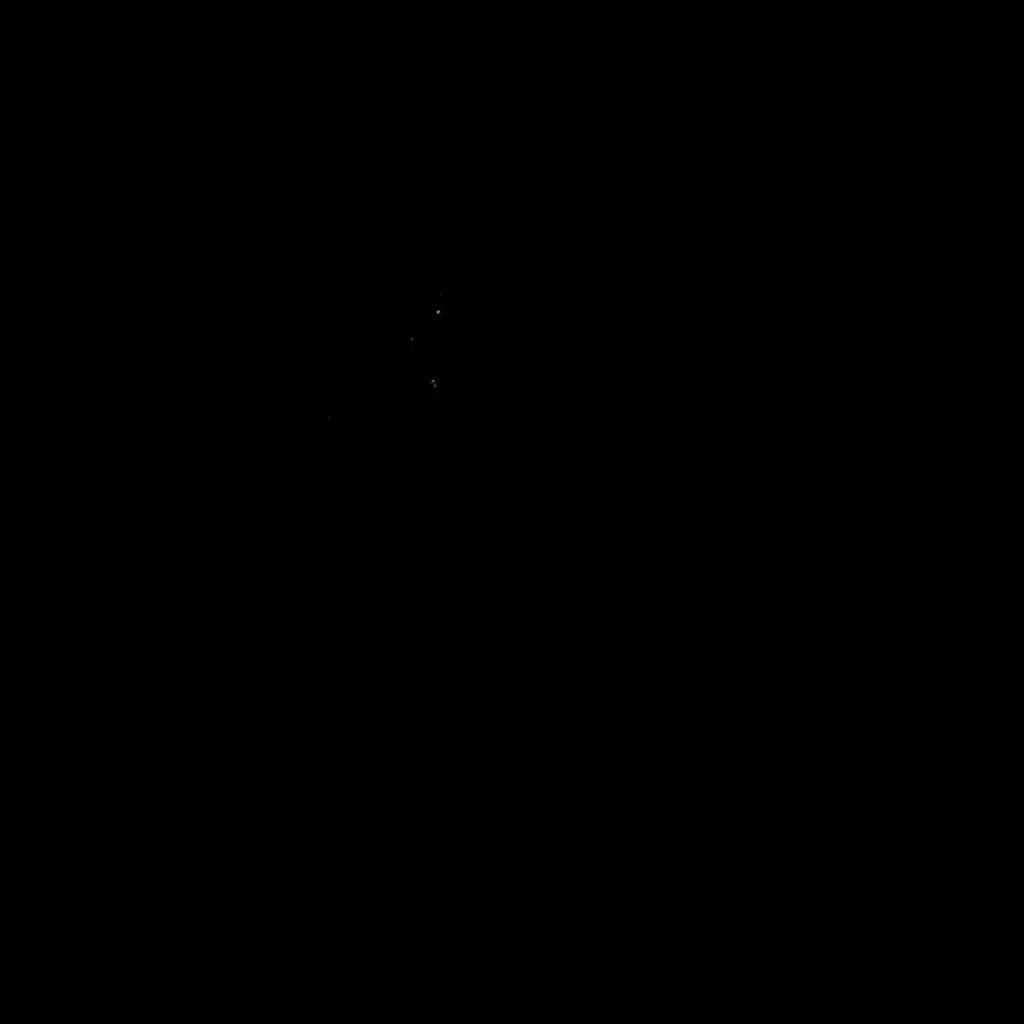

Supplement: Supplementary file 9 — Expanded View Figure and Appendix source data [file 44319_2025_513_MOESM9_ESM.zip › Expanded View Figure and Appendix source data/Expanded View Figure 5/EV 5D/sgPCAF 8 h/GH2AX.tif]

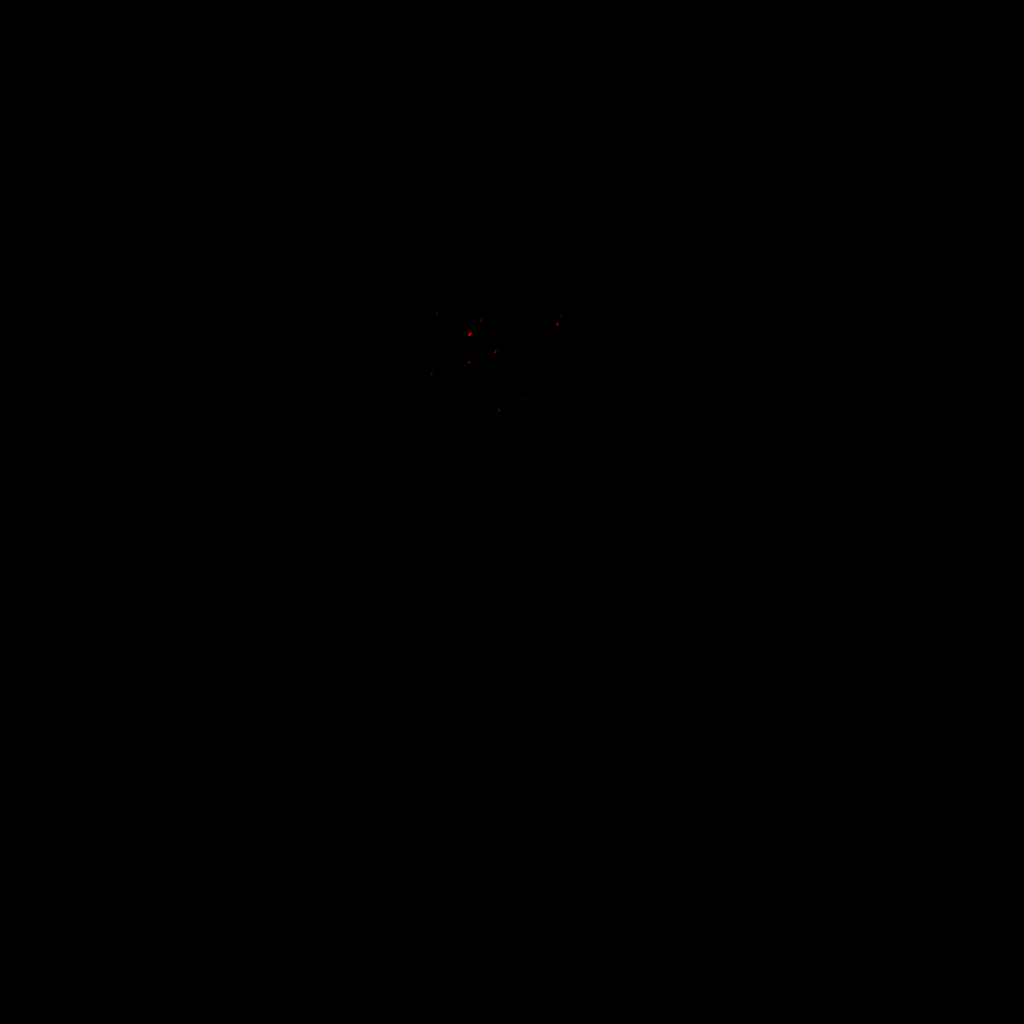

Supplement: Supplementary file 9 — Expanded View Figure and Appendix source data [file 44319_2025_513_MOESM9_ESM.zip › Expanded View Figure and Appendix source data/Expanded View Figure 5/EV 5D/sgPCAF 8 h/RAD51.tif]

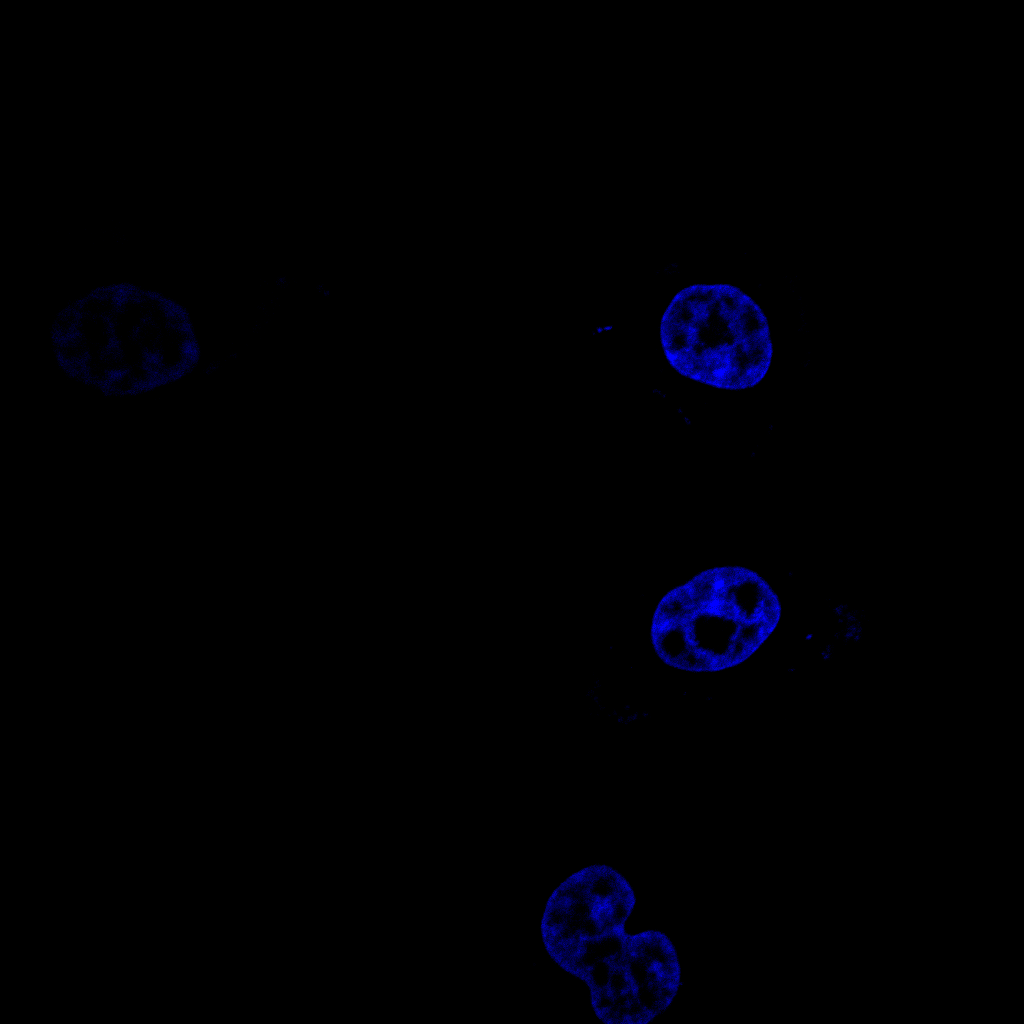

Supplement: Supplementary file 9 — Expanded View Figure and Appendix source data [file 44319_2025_513_MOESM9_ESM.zip › Expanded View Figure and Appendix source data/Expanded View Figure 5/EV 5D/sgPCAF ETO-/DAPI.tif]

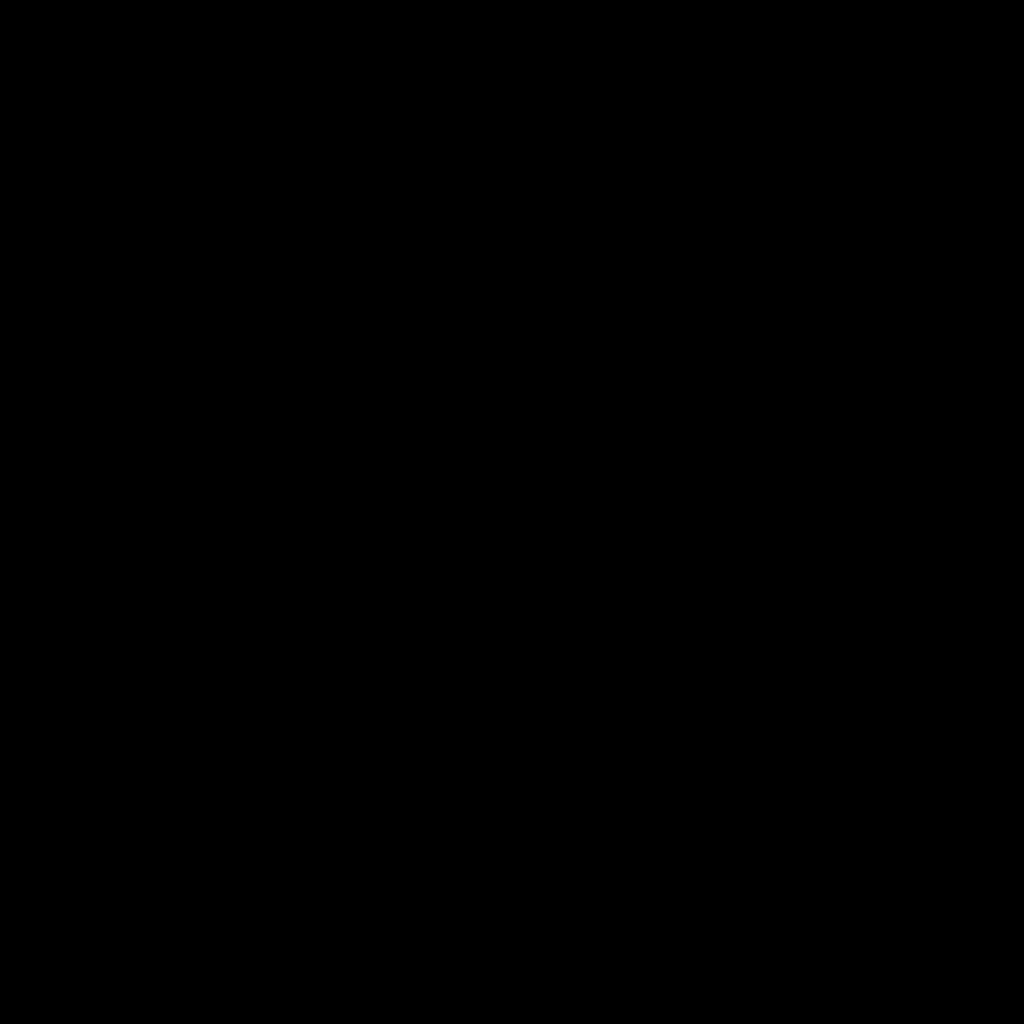

Supplement: Supplementary file 9 — Expanded View Figure and Appendix source data [file 44319_2025_513_MOESM9_ESM.zip › Expanded View Figure and Appendix source data/Expanded View Figure 5/EV 5D/sgPCAF ETO-/GH2AX.tif]

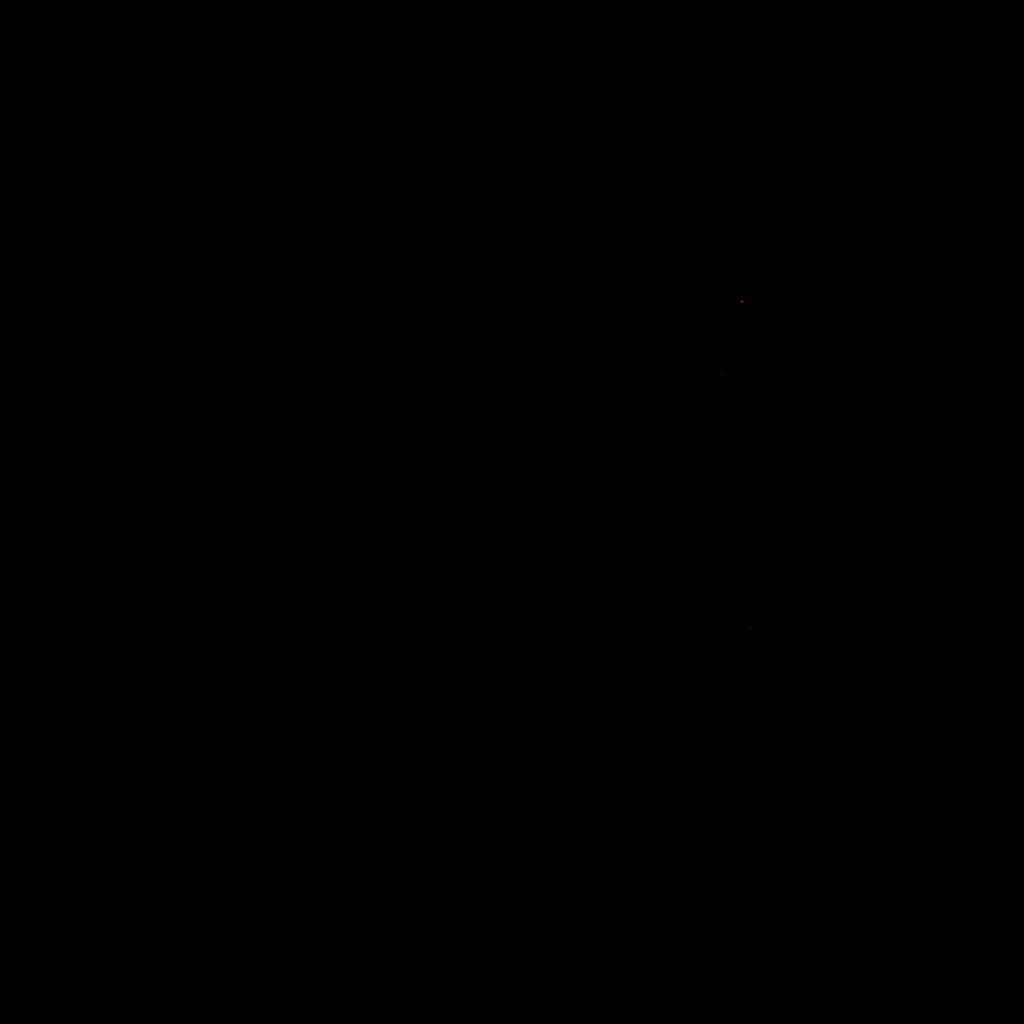

Supplement: Supplementary file 9 — Expanded View Figure and Appendix source data [file 44319_2025_513_MOESM9_ESM.zip › Expanded View Figure and Appendix source data/Expanded View Figure 5/EV 5D/sgPCAF ETO-/RAD51.tif]

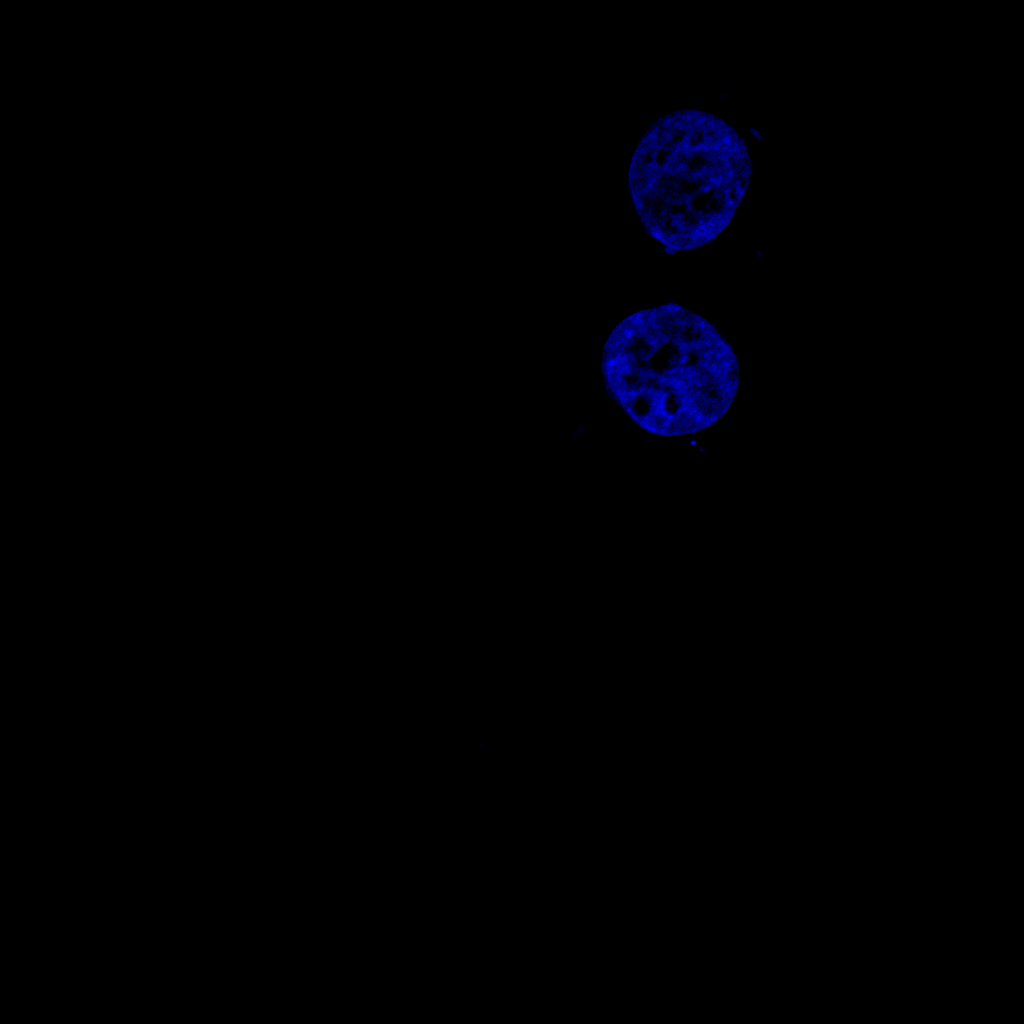

Supplement: Supplementary file 9 — Expanded View Figure and Appendix source data [file 44319_2025_513_MOESM9_ESM.zip › Expanded View Figure and Appendix source data/Expanded View Figure 5/EV 5E/EV ETO+/DAPI.tif]

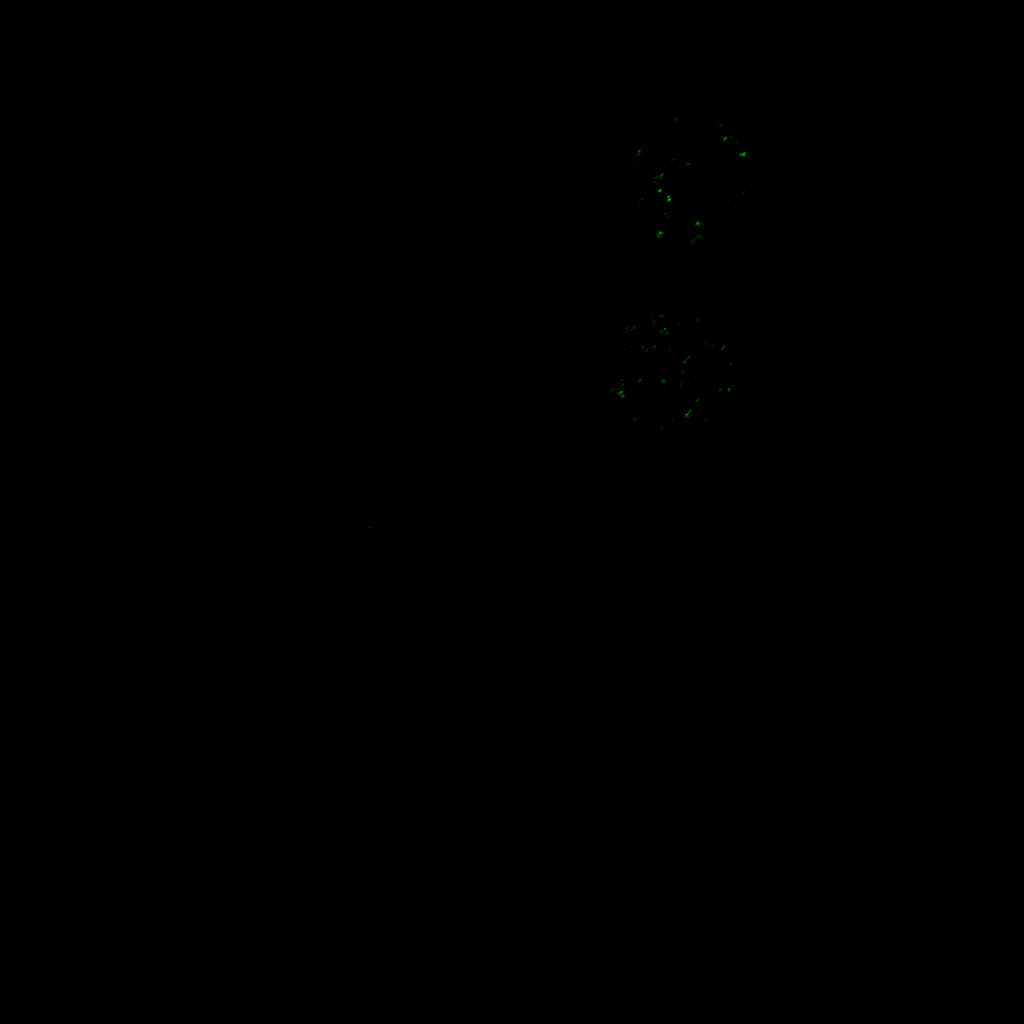

Supplement: Supplementary file 9 — Expanded View Figure and Appendix source data [file 44319_2025_513_MOESM9_ESM.zip › Expanded View Figure and Appendix source data/Expanded View Figure 5/EV 5E/EV ETO+/GH2AX.tif]

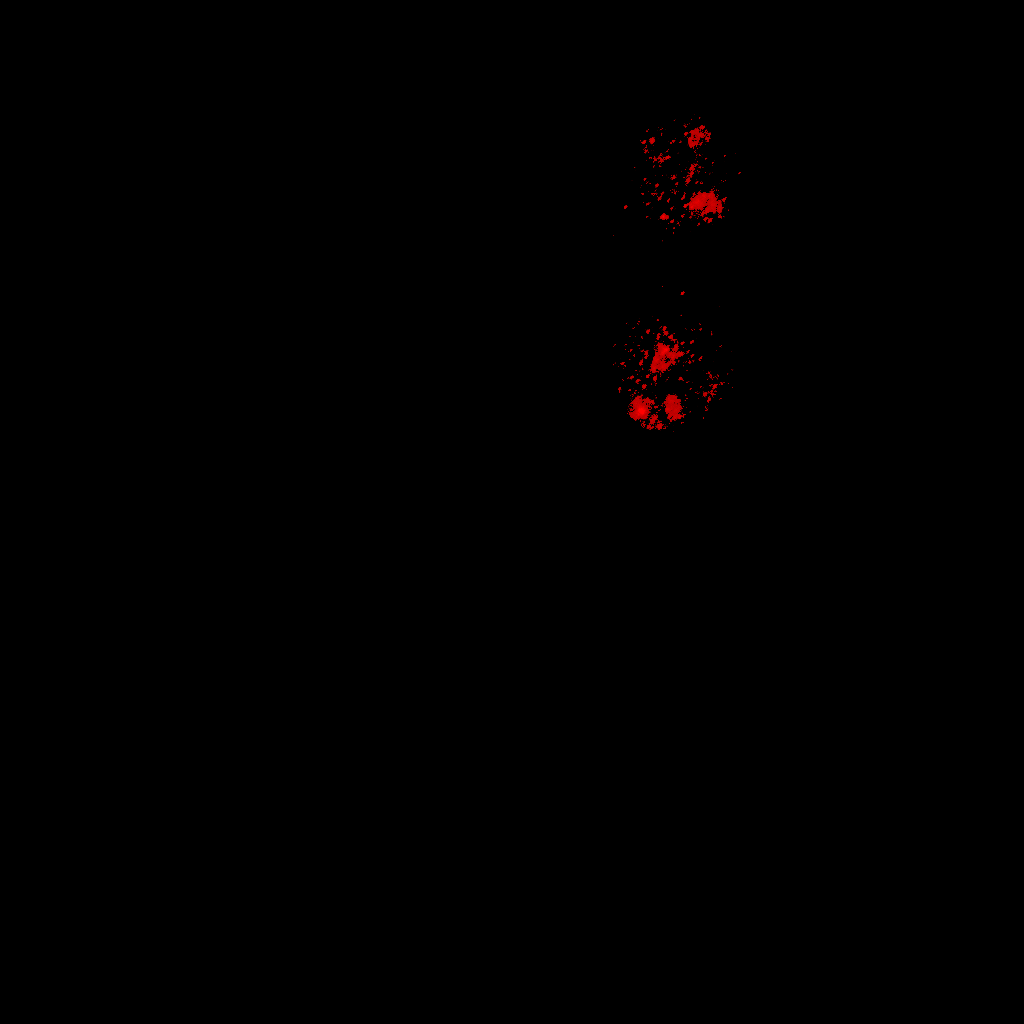

Supplement: Supplementary file 9 — Expanded View Figure and Appendix source data [file 44319_2025_513_MOESM9_ESM.zip › Expanded View Figure and Appendix source data/Expanded View Figure 5/EV 5E/EV ETO+/RAD51.tif]

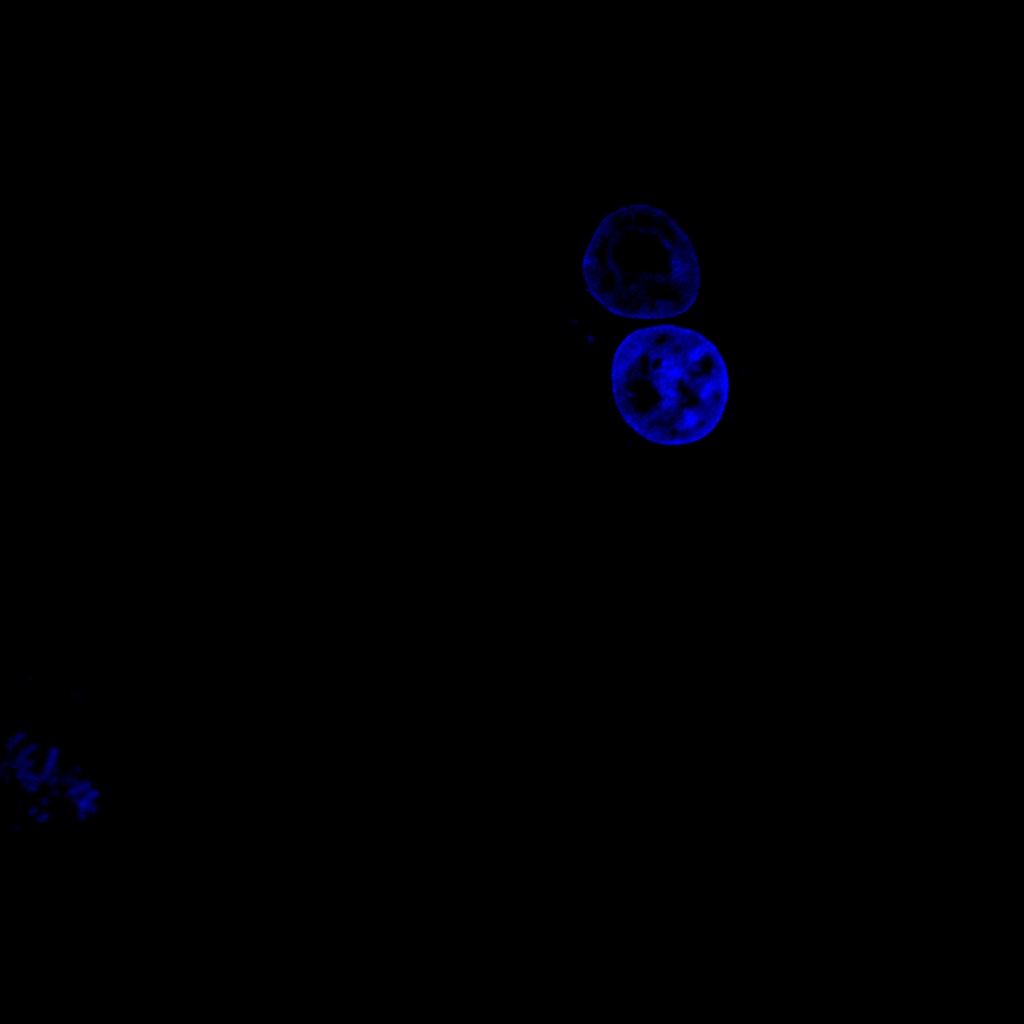

Supplement: Supplementary file 9 — Expanded View Figure and Appendix source data [file 44319_2025_513_MOESM9_ESM.zip › Expanded View Figure and Appendix source data/Expanded View Figure 5/EV 5E/EV ETO-/DAPI.tif]

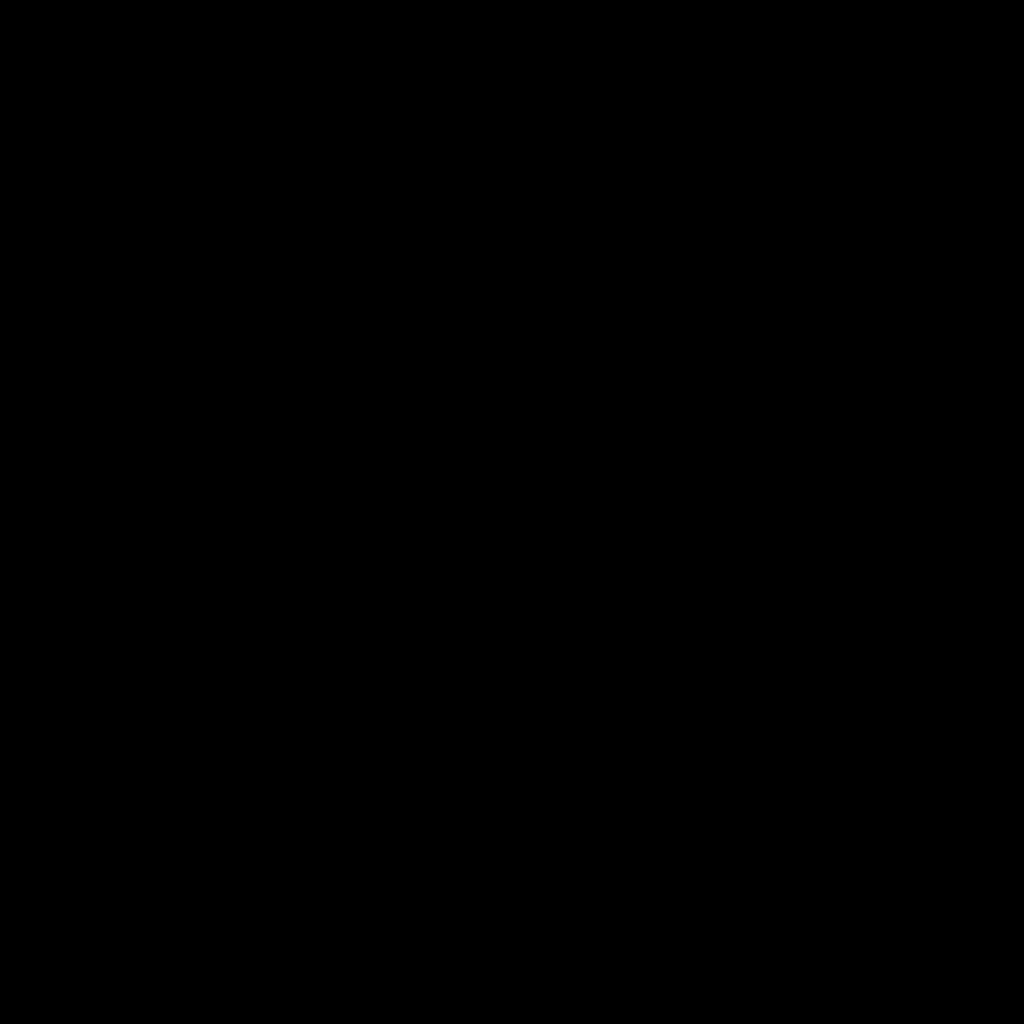

Supplement: Supplementary file 9 — Expanded View Figure and Appendix source data [file 44319_2025_513_MOESM9_ESM.zip › Expanded View Figure and Appendix source data/Expanded View Figure 5/EV 5E/EV ETO-/GH2AX.tif]

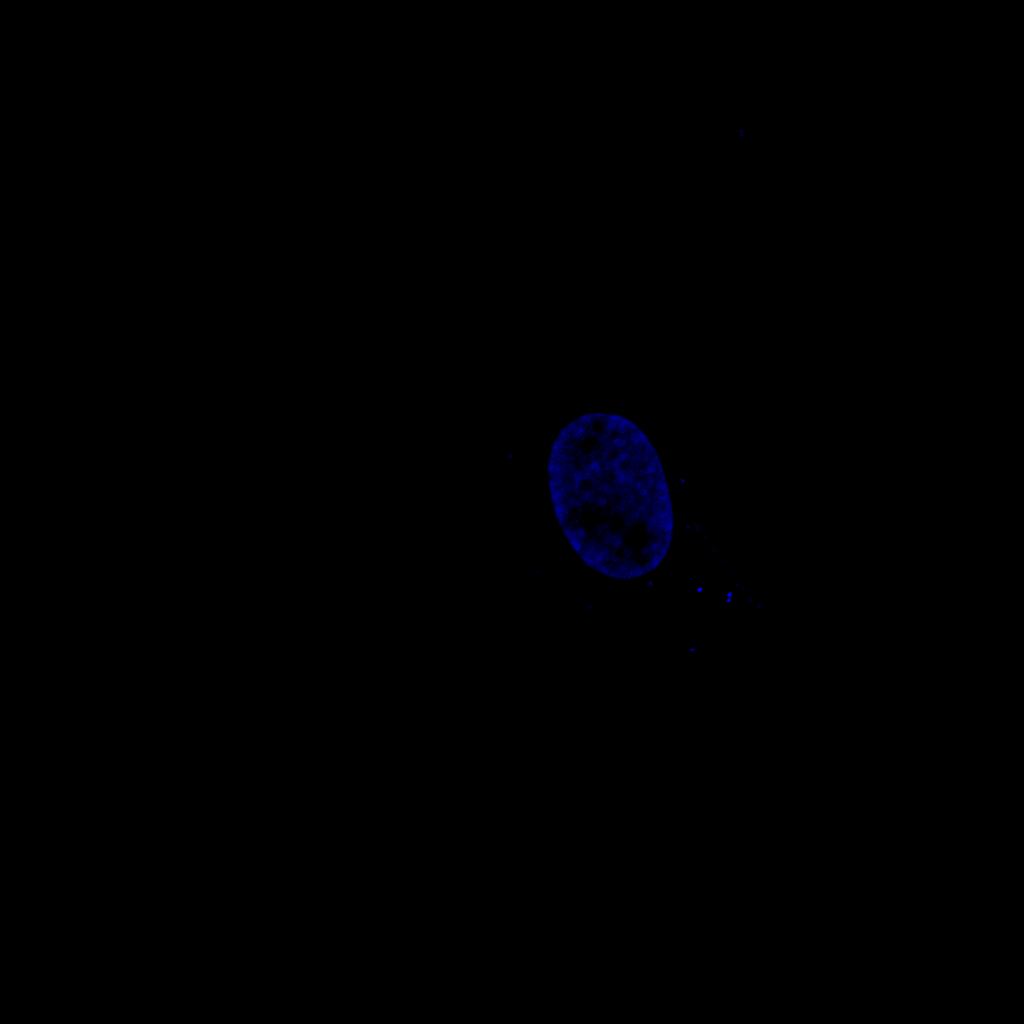

Supplement: Supplementary file 9 — Expanded View Figure and Appendix source data [file 44319_2025_513_MOESM9_ESM.zip › Expanded View Figure and Appendix source data/Expanded View Figure 5/EV 5E/PCAF ETO+/DAPI.tif]

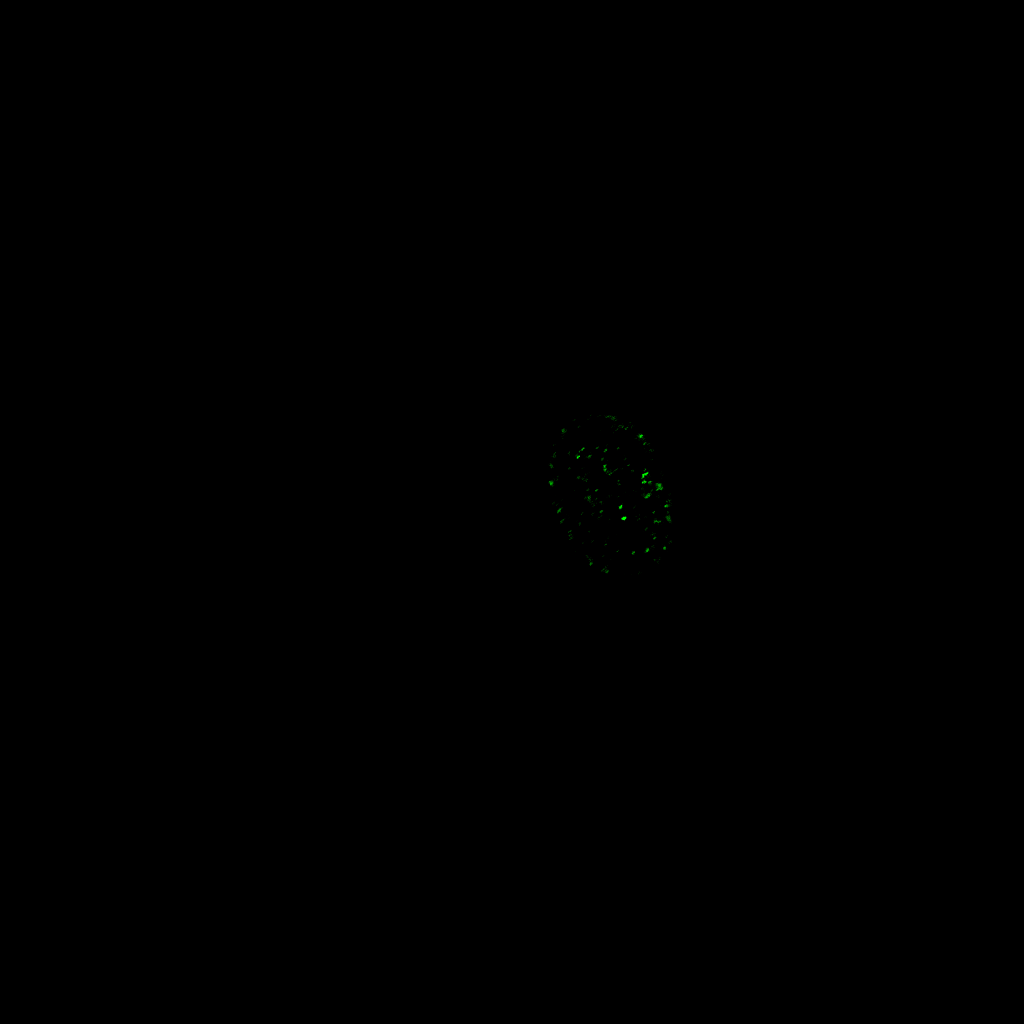

Supplement: Supplementary file 9 — Expanded View Figure and Appendix source data [file 44319_2025_513_MOESM9_ESM.zip › Expanded View Figure and Appendix source data/Expanded View Figure 5/EV 5E/PCAF ETO+/GH2AX.tif]

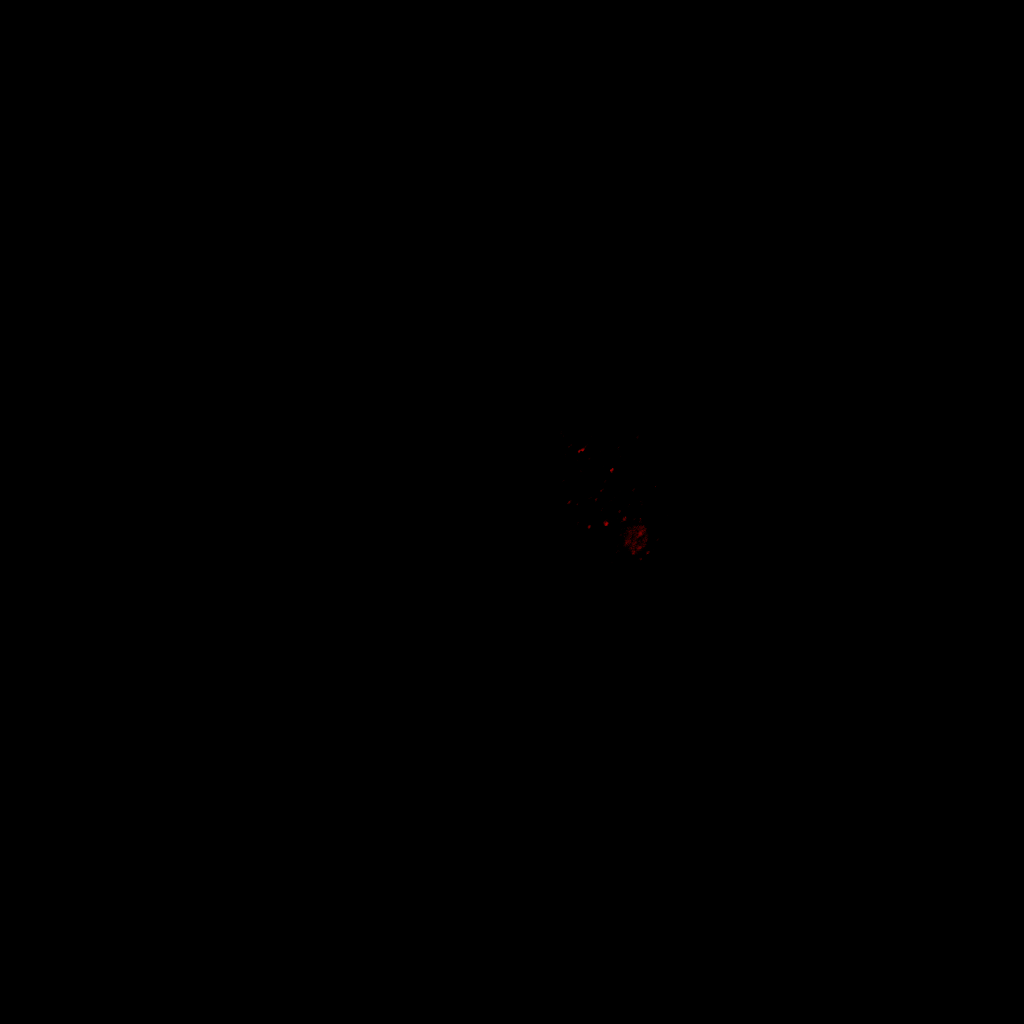

Supplement: Supplementary file 9 — Expanded View Figure and Appendix source data [file 44319_2025_513_MOESM9_ESM.zip › Expanded View Figure and Appendix source data/Expanded View Figure 5/EV 5E/PCAF ETO+/RAD51.tif]

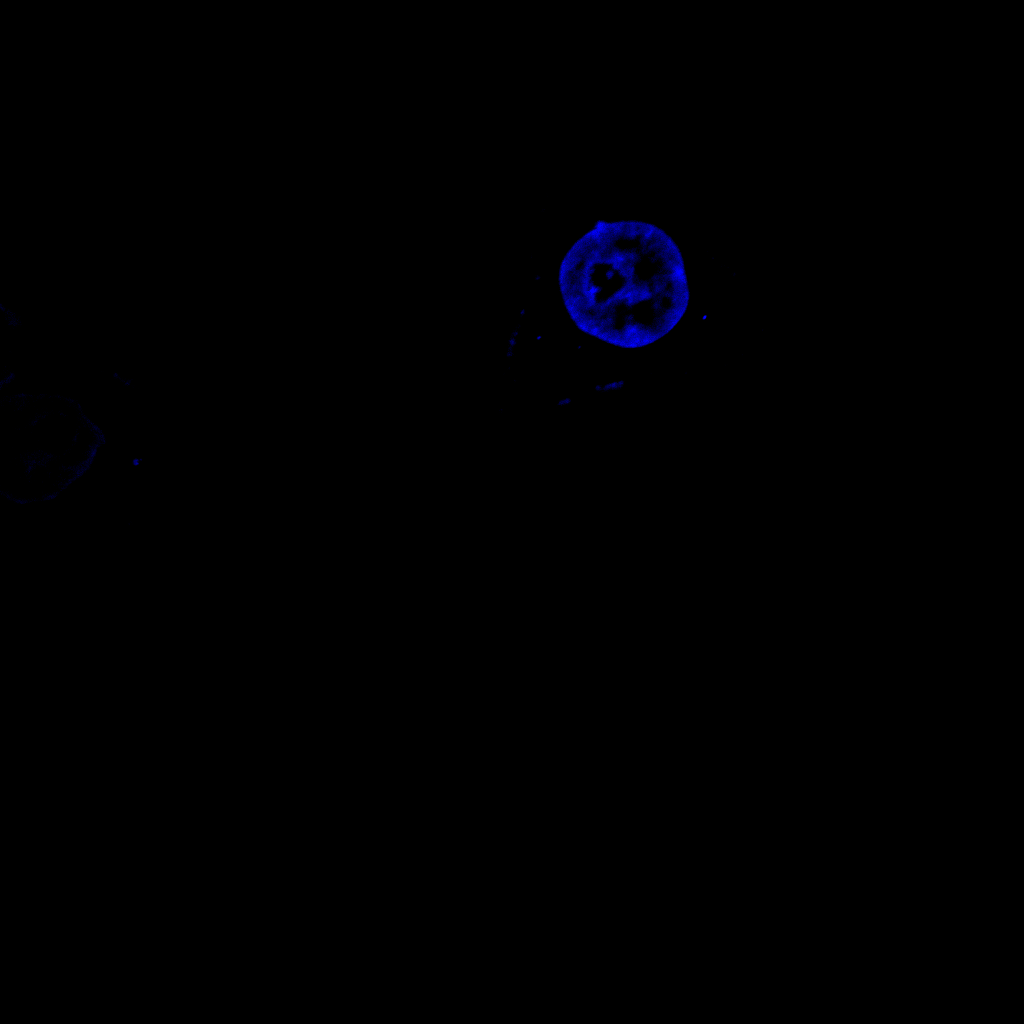

Supplement: Supplementary file 9 — Expanded View Figure and Appendix source data [file 44319_2025_513_MOESM9_ESM.zip › Expanded View Figure and Appendix source data/Expanded View Figure 5/EV 5E/PCAF ETO-/DAPI.tif]

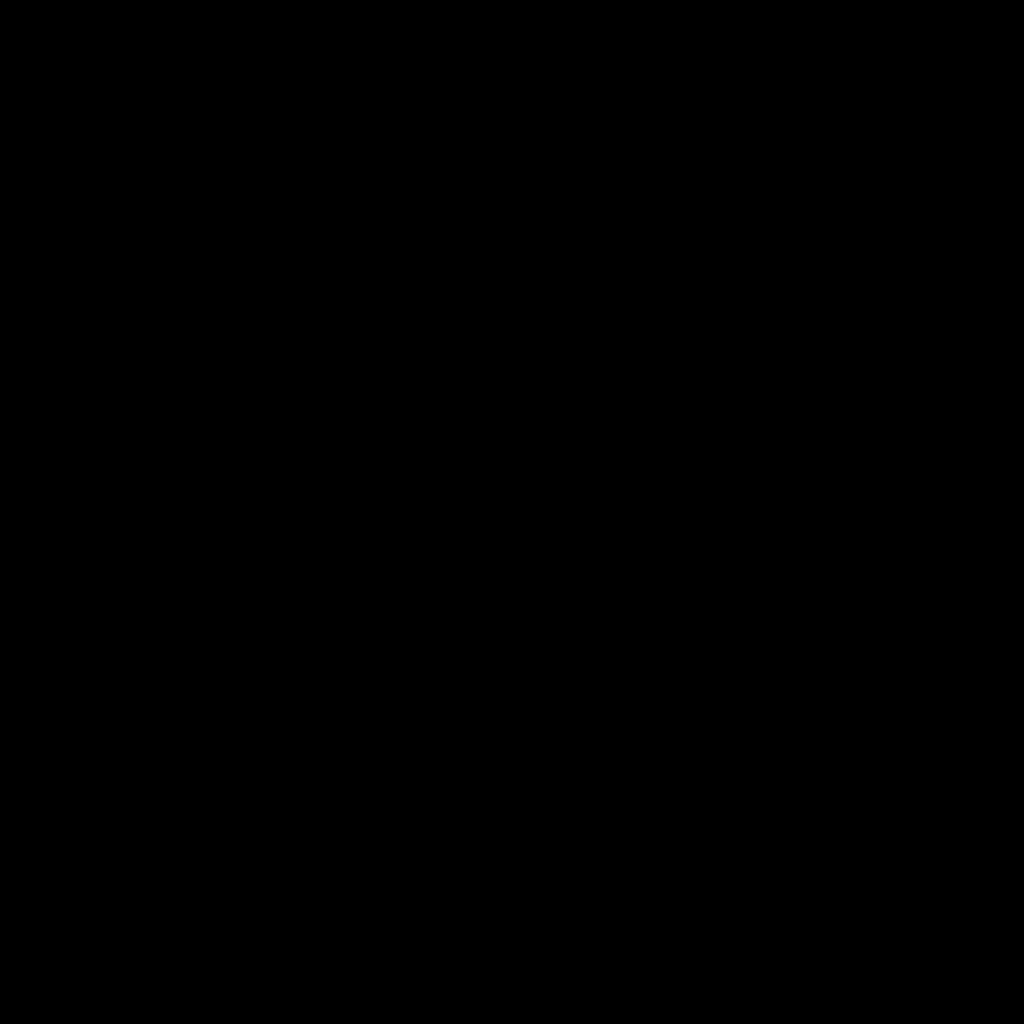

Supplement: Supplementary file 9 — Expanded View Figure and Appendix source data [file 44319_2025_513_MOESM9_ESM.zip › Expanded View Figure and Appendix source data/Expanded View Figure 5/EV 5E/PCAF ETO-/GH2AX.tif]

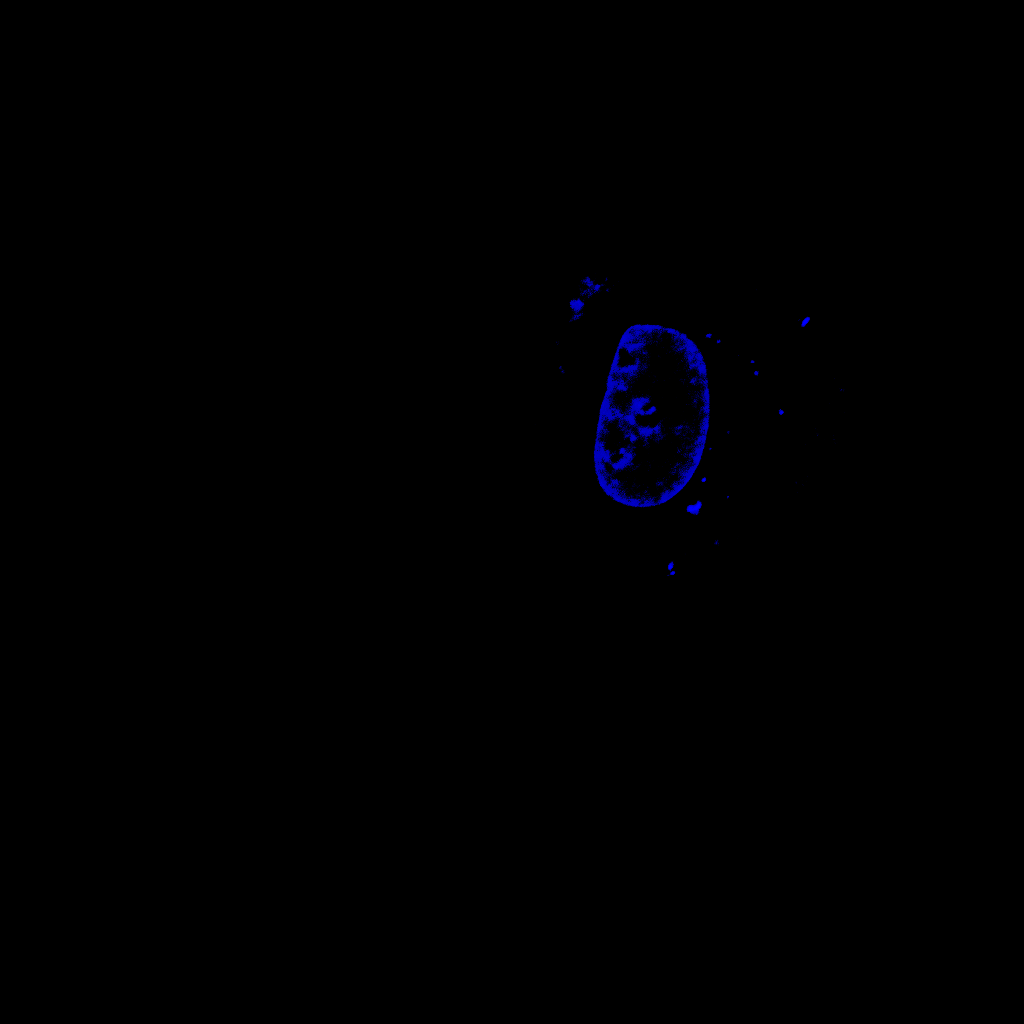

Supplement: Supplementary file 9 — Expanded View Figure and Appendix source data [file 44319_2025_513_MOESM9_ESM.zip › Expanded View Figure and Appendix source data/Expanded View Figure 5/EV 5E/PCAF+RAD51 ETO+/DAPI.tif]

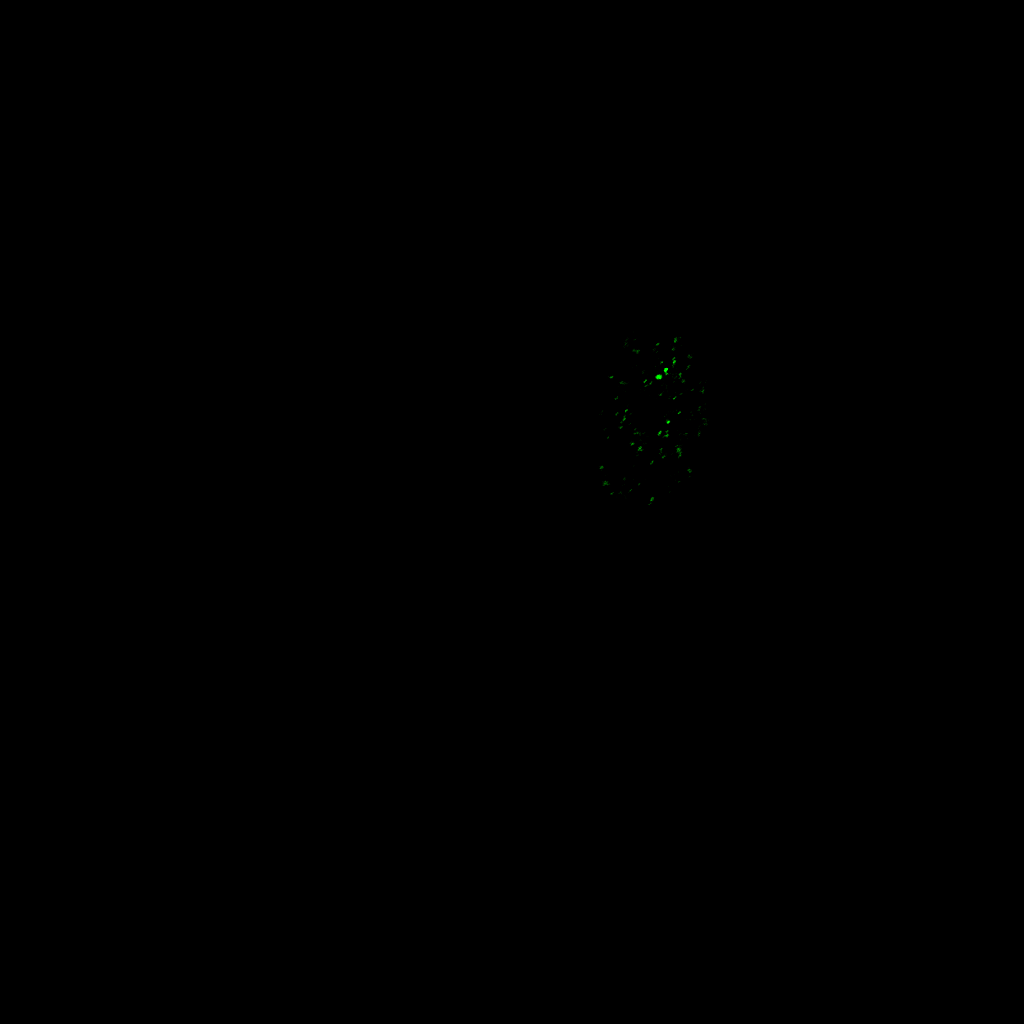

Supplement: Supplementary file 9 — Expanded View Figure and Appendix source data [file 44319_2025_513_MOESM9_ESM.zip › Expanded View Figure and Appendix source data/Expanded View Figure 5/EV 5E/PCAF+RAD51 ETO+/GH2AX.tif]

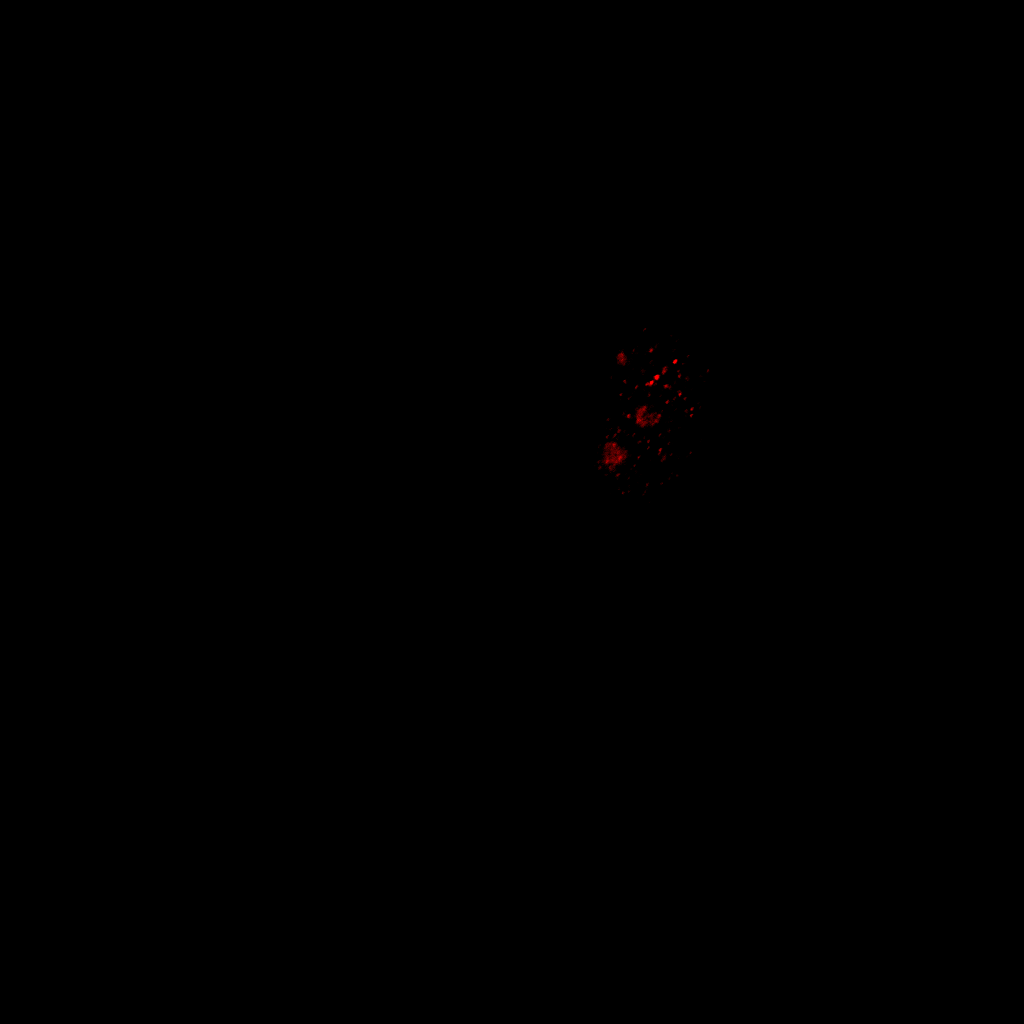

Supplement: Supplementary file 9 — Expanded View Figure and Appendix source data [file 44319_2025_513_MOESM9_ESM.zip › Expanded View Figure and Appendix source data/Expanded View Figure 5/EV 5E/PCAF+RAD51 ETO+/RAD51.tif]

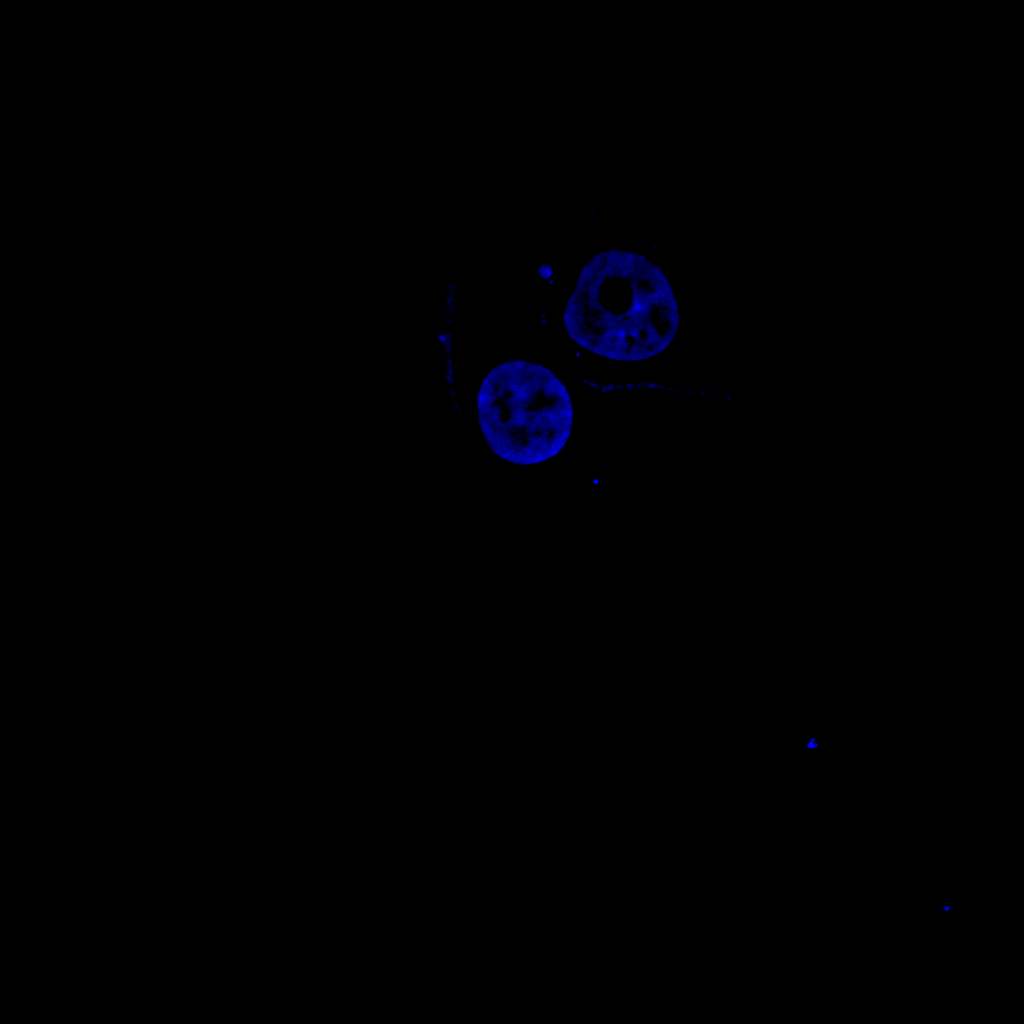

Supplement: Supplementary file 9 — Expanded View Figure and Appendix source data [file 44319_2025_513_MOESM9_ESM.zip › Expanded View Figure and Appendix source data/Expanded View Figure 5/EV 5E/PCAF+RAD51 ETO-/DAPI.tif]

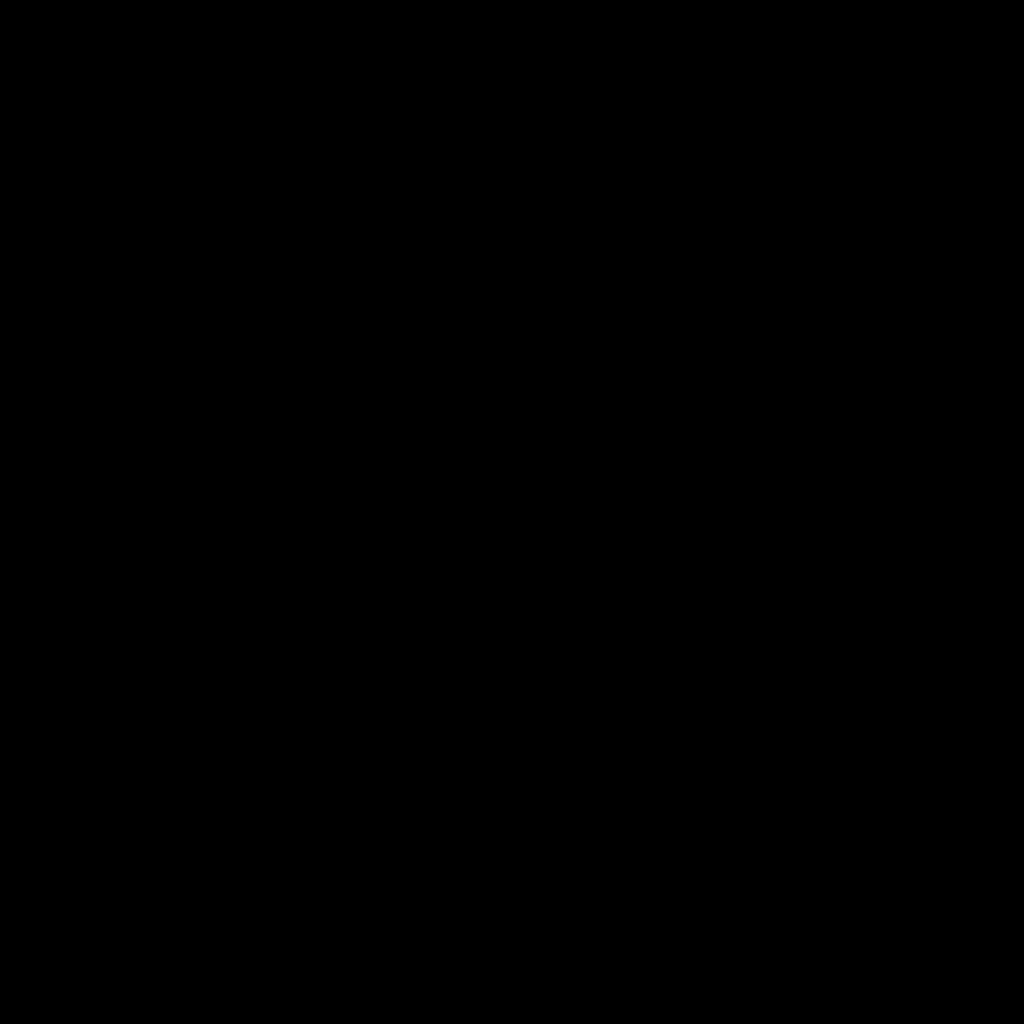

Supplement: Supplementary file 9 — Expanded View Figure and Appendix source data [file 44319_2025_513_MOESM9_ESM.zip › Expanded View Figure and Appendix source data/Expanded View Figure 5/EV 5E/PCAF+RAD51 ETO-/GH2AX.tif]

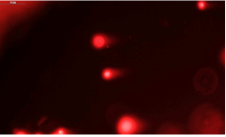

Supplement: Supplementary file 9 — Expanded View Figure and Appendix source data [file 44319_2025_513_MOESM9_ESM.zip › Expanded View Figure and Appendix source data/Expanded View Figure 5/EV 5F/DETA ETO+.tif]

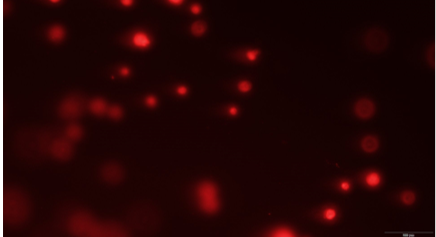

Supplement: Supplementary file 9 — Expanded View Figure and Appendix source data [file 44319_2025_513_MOESM9_ESM.zip › Expanded View Figure and Appendix source data/Expanded View Figure 5/EV 5F/DETA ETO-.tif]

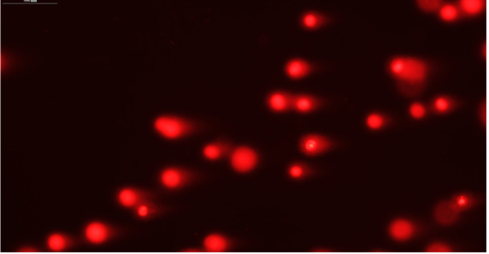

Supplement: Supplementary file 9 — Expanded View Figure and Appendix source data [file 44319_2025_513_MOESM9_ESM.zip › Expanded View Figure and Appendix source data/Expanded View Figure 5/EV 5F/NC ETO+.tif]

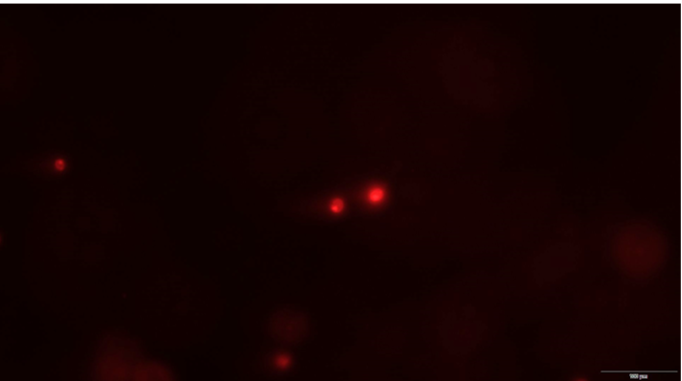

Supplement: Supplementary file 9 — Expanded View Figure and Appendix source data [file 44319_2025_513_MOESM9_ESM.zip › Expanded View Figure and Appendix source data/Expanded View Figure 5/EV 5F/NC ETO-.tif]

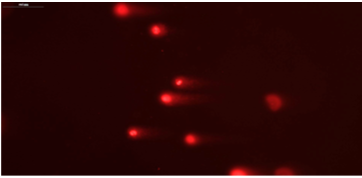

Supplement: Supplementary file 9 — Expanded View Figure and Appendix source data [file 44319_2025_513_MOESM9_ESM.zip › Expanded View Figure and Appendix source data/Expanded View Figure 5/EV 5F/WT ETO+.tif]

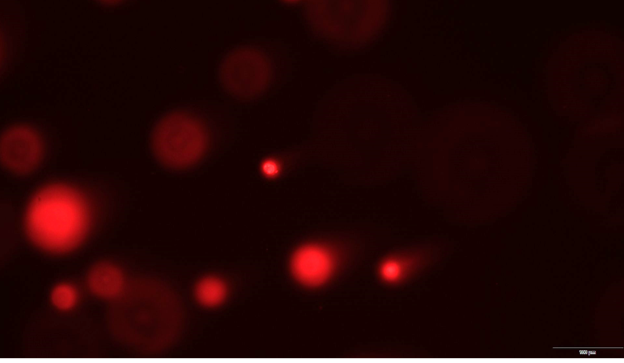

Supplement: Supplementary file 9 — Expanded View Figure and Appendix source data [file 44319_2025_513_MOESM9_ESM.zip › Expanded View Figure and Appendix source data/Expanded View Figure 5/EV 5F/WT ETO-.tif]

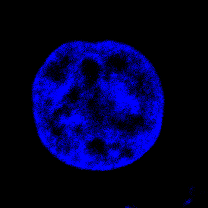

Supplement: Supplementary file 9 — Expanded View Figure and Appendix source data [file 44319_2025_513_MOESM9_ESM.zip › Expanded View Figure and Appendix source data/Expanded View Figure 2/EV 2F/ETO+ DAPI.tif]

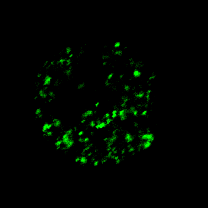

Supplement: Supplementary file 9 — Expanded View Figure and Appendix source data [file 44319_2025_513_MOESM9_ESM.zip › Expanded View Figure and Appendix source data/Expanded View Figure 2/EV 2F/ETO+ GH2AX.tif]

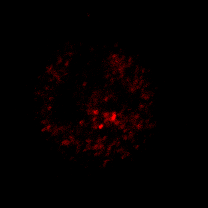

Supplement: Supplementary file 9 — Expanded View Figure and Appendix source data [file 44319_2025_513_MOESM9_ESM.zip › Expanded View Figure and Appendix source data/Expanded View Figure 2/EV 2F/ETO+ PCAF.tif]

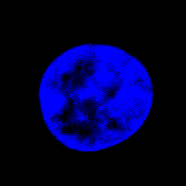

Supplement: Supplementary file 9 — Expanded View Figure and Appendix source data [file 44319_2025_513_MOESM9_ESM.zip › Expanded View Figure and Appendix source data/Expanded View Figure 2/EV 2F/ETO- DAPI.tif]

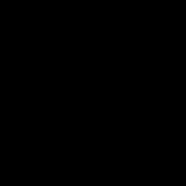

Supplement: Supplementary file 9 — Expanded View Figure and Appendix source data [file 44319_2025_513_MOESM9_ESM.zip › Expanded View Figure and Appendix source data/Expanded View Figure 2/EV 2F/ETO- GH2AX.tif]

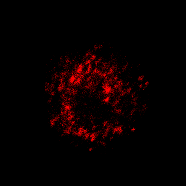

Supplement: Supplementary file 9 — Expanded View Figure and Appendix source data [file 44319_2025_513_MOESM9_ESM.zip › Expanded View Figure and Appendix source data/Expanded View Figure 2/EV 2F/ETO- PCAF.tif]

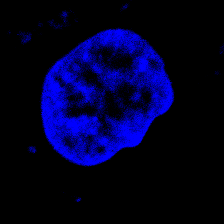

Supplement: Supplementary file 9 — Expanded View Figure and Appendix source data [file 44319_2025_513_MOESM9_ESM.zip › Expanded View Figure and Appendix source data/Expanded View Figure 2/EV 2G/eto+ dapi.tif]

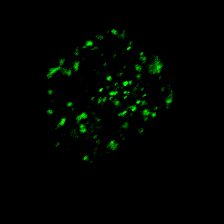

Supplement: Supplementary file 9 — Expanded View Figure and Appendix source data [file 44319_2025_513_MOESM9_ESM.zip › Expanded View Figure and Appendix source data/Expanded View Figure 2/EV 2G/eto+ pcaf.tif]

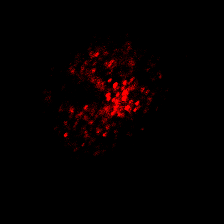

Supplement: Supplementary file 9 — Expanded View Figure and Appendix source data [file 44319_2025_513_MOESM9_ESM.zip › Expanded View Figure and Appendix source data/Expanded View Figure 2/EV 2G/eto+ rad.tif]

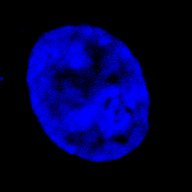

Supplement: Supplementary file 9 — Expanded View Figure and Appendix source data [file 44319_2025_513_MOESM9_ESM.zip › Expanded View Figure and Appendix source data/Expanded View Figure 2/EV 2G/eto- dapi.tif]

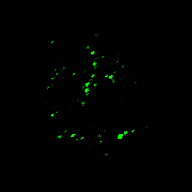

Supplement: Supplementary file 9 — Expanded View Figure and Appendix source data [file 44319_2025_513_MOESM9_ESM.zip › Expanded View Figure and Appendix source data/Expanded View Figure 2/EV 2G/eto- pcaf-1.tif]

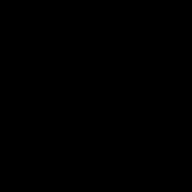

Supplement: Supplementary file 9 — Expanded View Figure and Appendix source data [file 44319_2025_513_MOESM9_ESM.zip › Expanded View Figure and Appendix source data/Expanded View Figure 2/EV 2G/eto- rad.tif]
