# Supplementary figures and images for: PCAF-mediated acetylation regulates RAD51 dynamic localization on chromatin during HR repair (part 3 of 4)
Source: EMBO Rep. 2025 Jul 15;26(16):4100–23. doi: 10.1038/s44319-025-00513-6 (PMC12373954; doi:10.1038/s44319-025-00513-6)

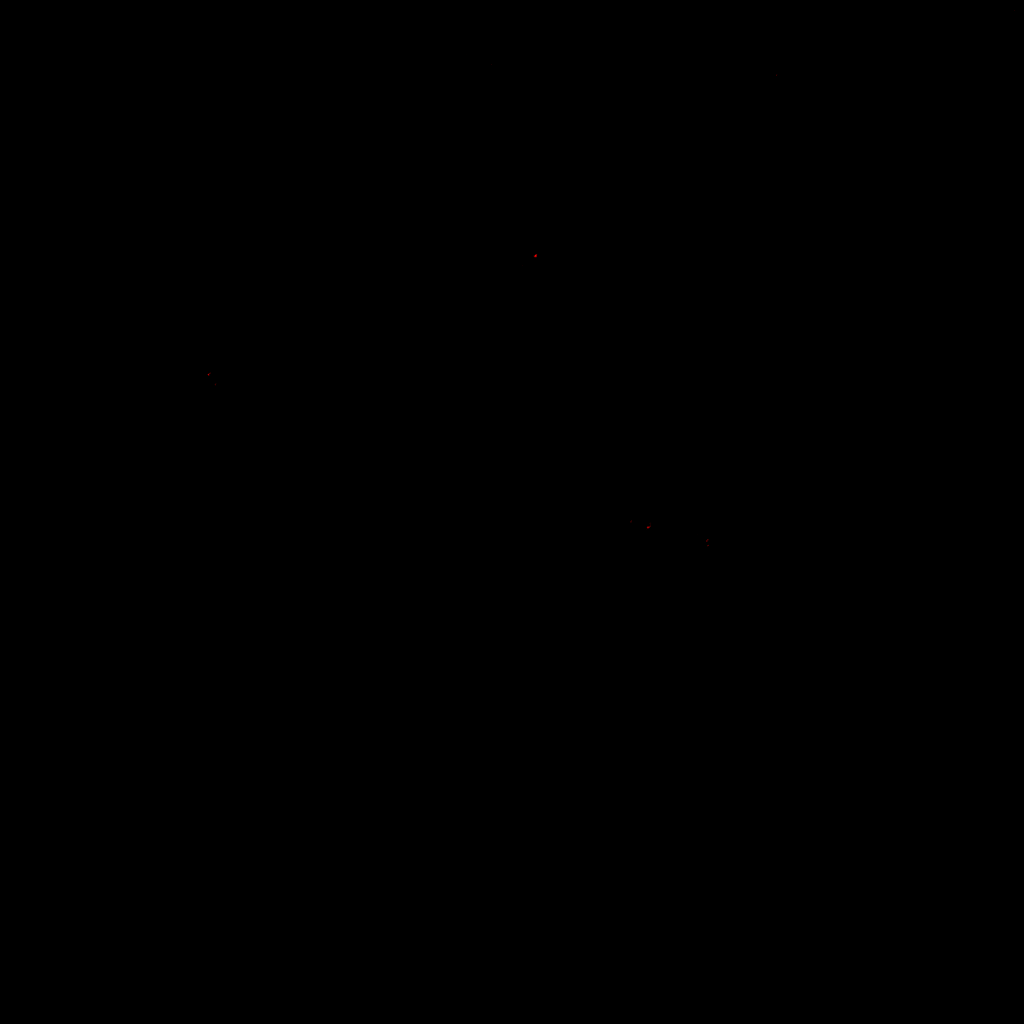

Supplement: Supplementary file 9 — Expanded View Figure and Appendix source data [file 44319_2025_513_MOESM9_ESM.zip › Expanded View Figure and Appendix source data/Appendix source data/S1 F/PCAFi ETO-/RAD51.tif]

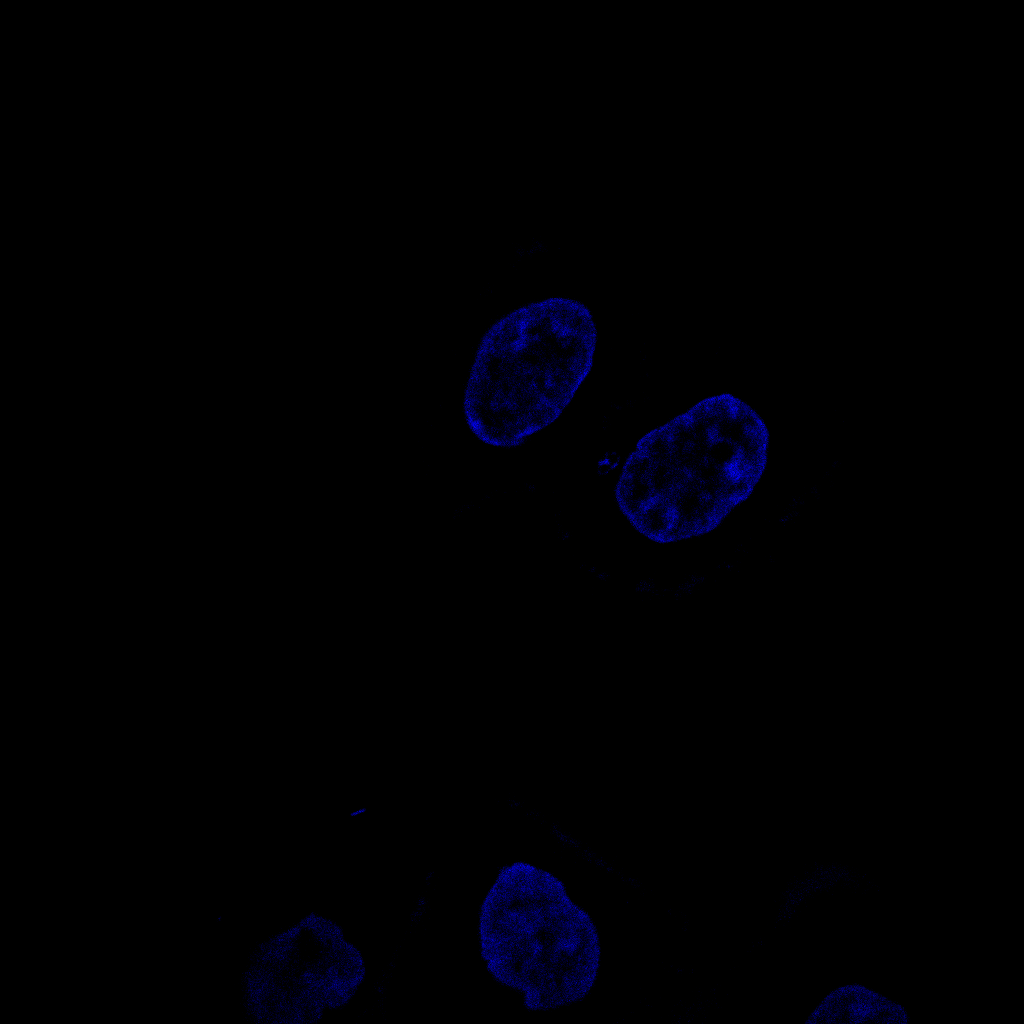

Supplement: Supplementary file 9 — Expanded View Figure and Appendix source data [file 44319_2025_513_MOESM9_ESM.zip › Expanded View Figure and Appendix source data/Expanded View Figure 1/EV 1B/DMSO 1 h/DAPI.tif]

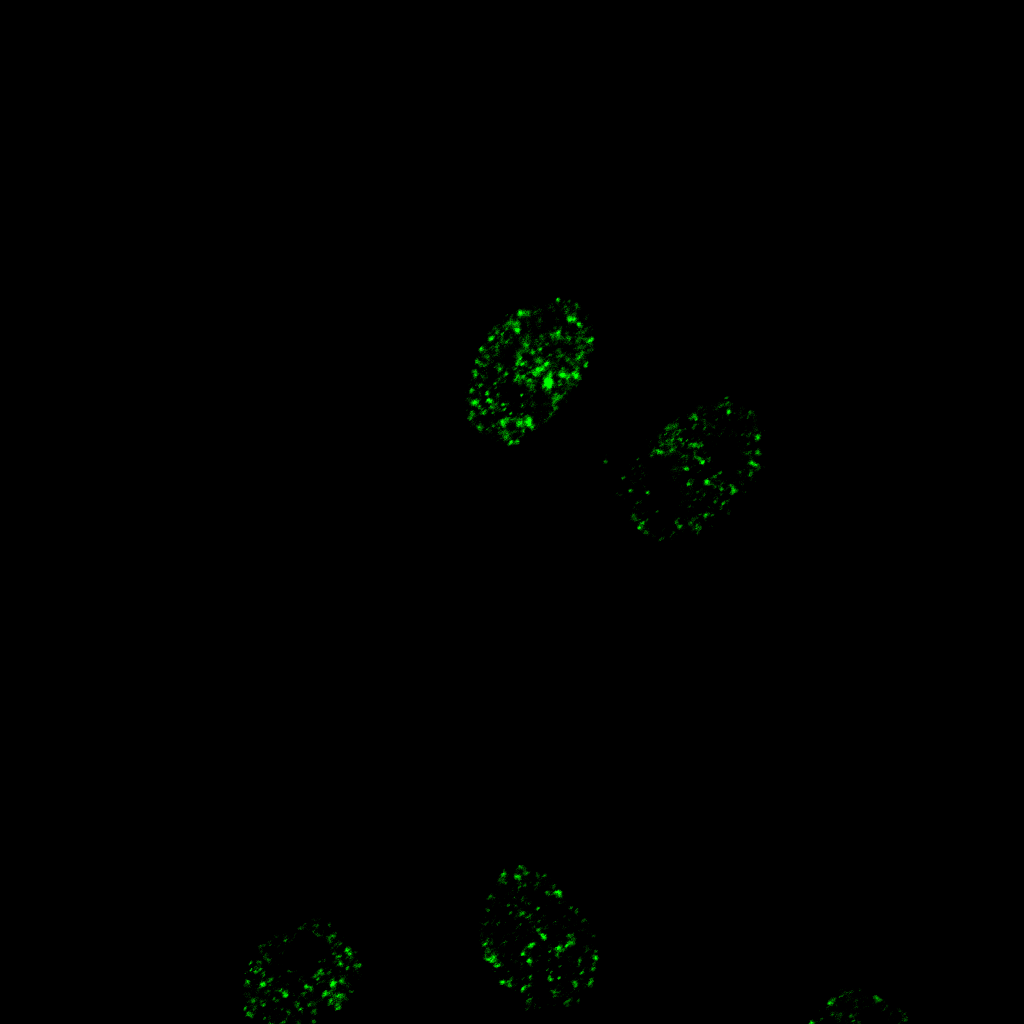

Supplement: Supplementary file 9 — Expanded View Figure and Appendix source data [file 44319_2025_513_MOESM9_ESM.zip › Expanded View Figure and Appendix source data/Expanded View Figure 1/EV 1B/DMSO 1 h/GH2AX.tif]

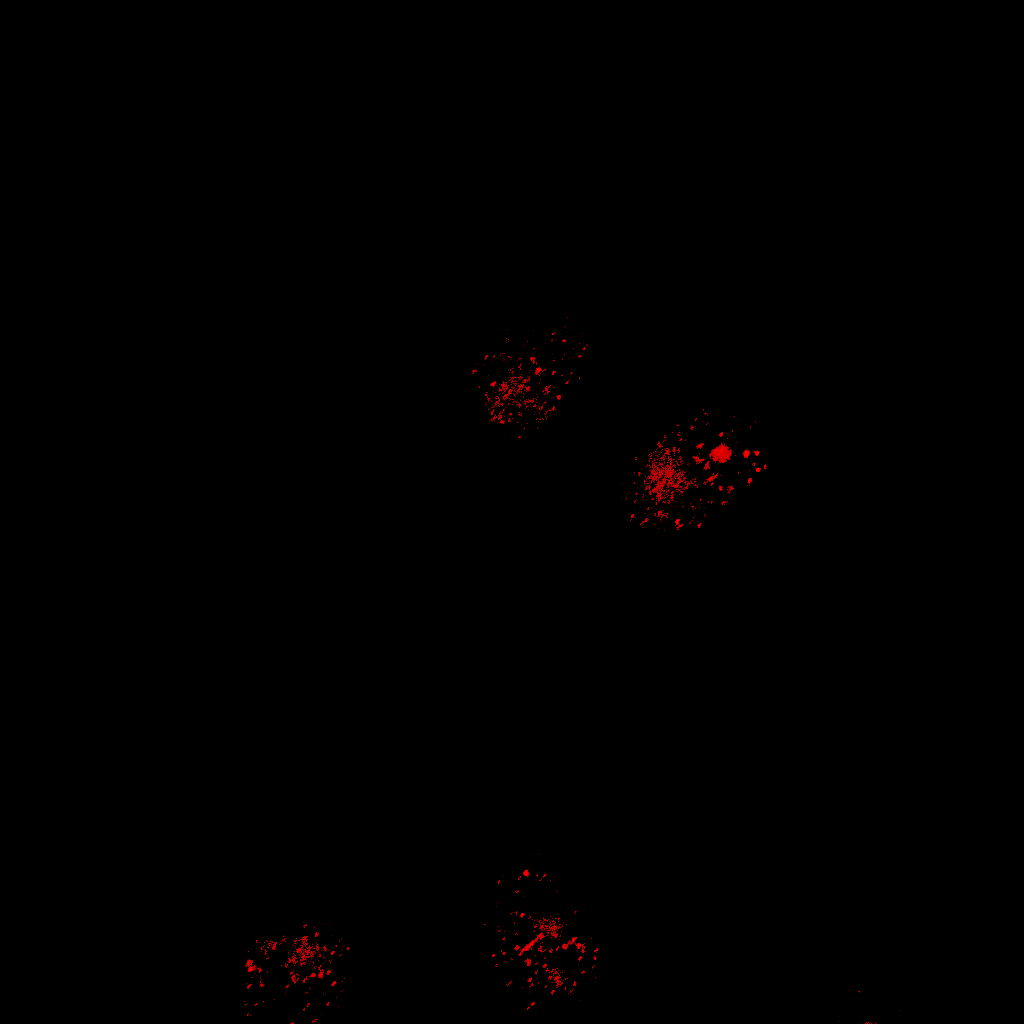

Supplement: Supplementary file 9 — Expanded View Figure and Appendix source data [file 44319_2025_513_MOESM9_ESM.zip › Expanded View Figure and Appendix source data/Expanded View Figure 1/EV 1B/DMSO 1 h/RAD51.tif]

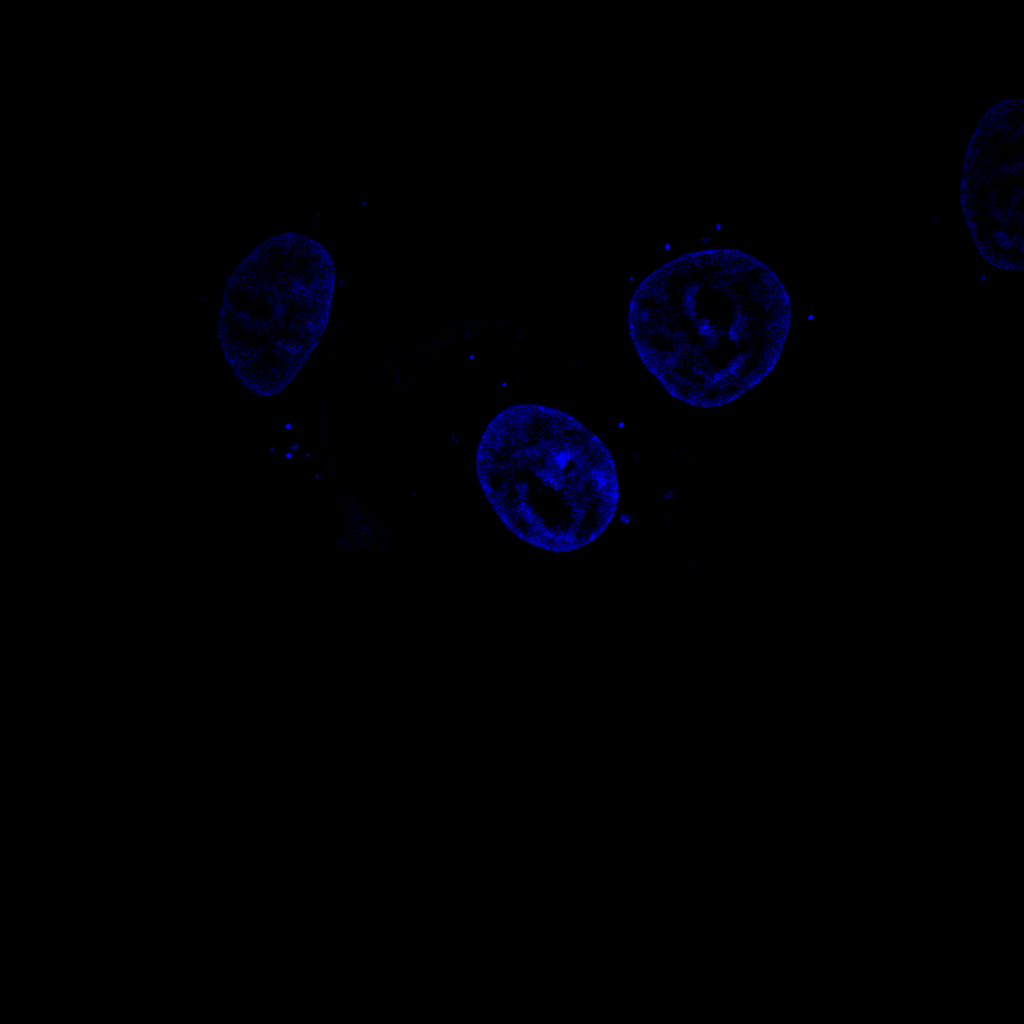

Supplement: Supplementary file 9 — Expanded View Figure and Appendix source data [file 44319_2025_513_MOESM9_ESM.zip › Expanded View Figure and Appendix source data/Expanded View Figure 1/EV 1B/DMSO 4 h/DAPI.tif]

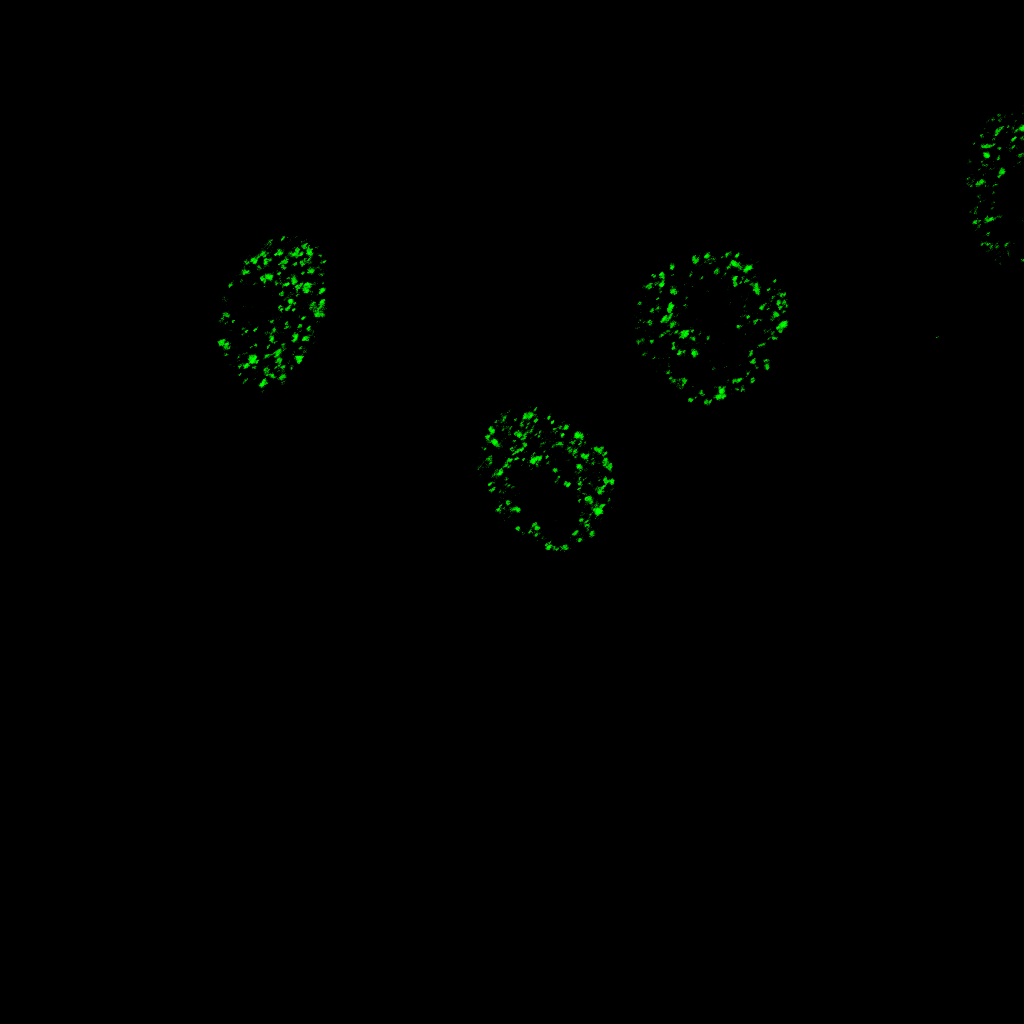

Supplement: Supplementary file 9 — Expanded View Figure and Appendix source data [file 44319_2025_513_MOESM9_ESM.zip › Expanded View Figure and Appendix source data/Expanded View Figure 1/EV 1B/DMSO 4 h/GH2AX.tif]

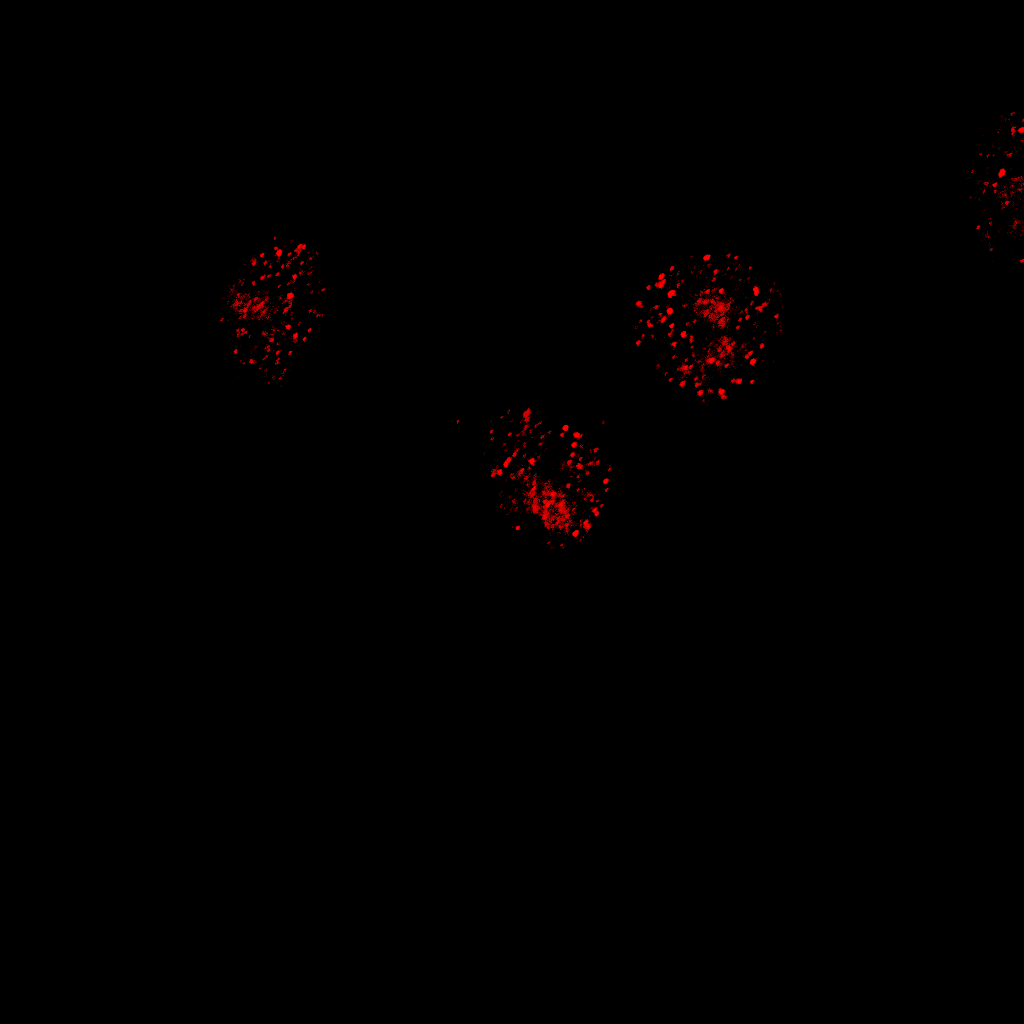

Supplement: Supplementary file 9 — Expanded View Figure and Appendix source data [file 44319_2025_513_MOESM9_ESM.zip › Expanded View Figure and Appendix source data/Expanded View Figure 1/EV 1B/DMSO 4 h/RAD51.tif]

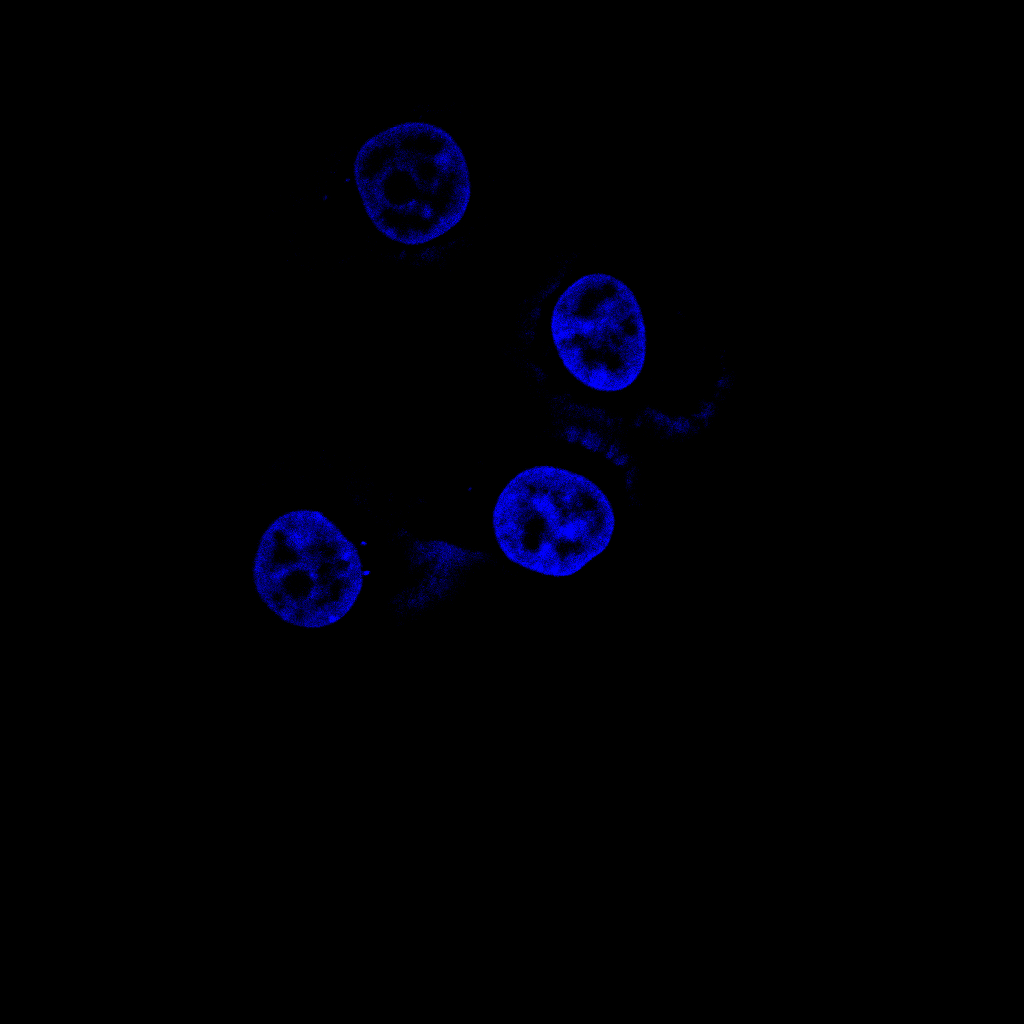

Supplement: Supplementary file 9 — Expanded View Figure and Appendix source data [file 44319_2025_513_MOESM9_ESM.zip › Expanded View Figure and Appendix source data/Expanded View Figure 1/EV 1B/DMSO 8 h/DAPI.tif]

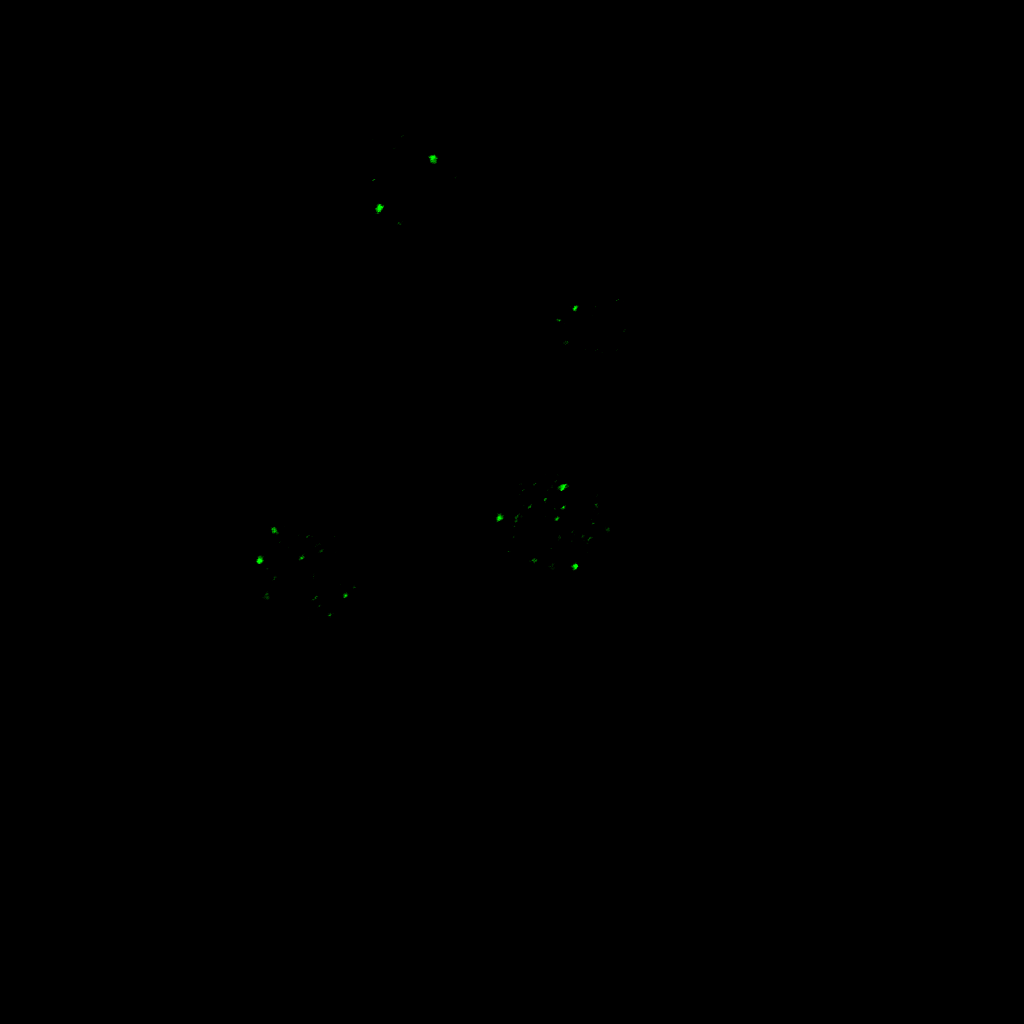

Supplement: Supplementary file 9 — Expanded View Figure and Appendix source data [file 44319_2025_513_MOESM9_ESM.zip › Expanded View Figure and Appendix source data/Expanded View Figure 1/EV 1B/DMSO 8 h/GH2AX.tif]

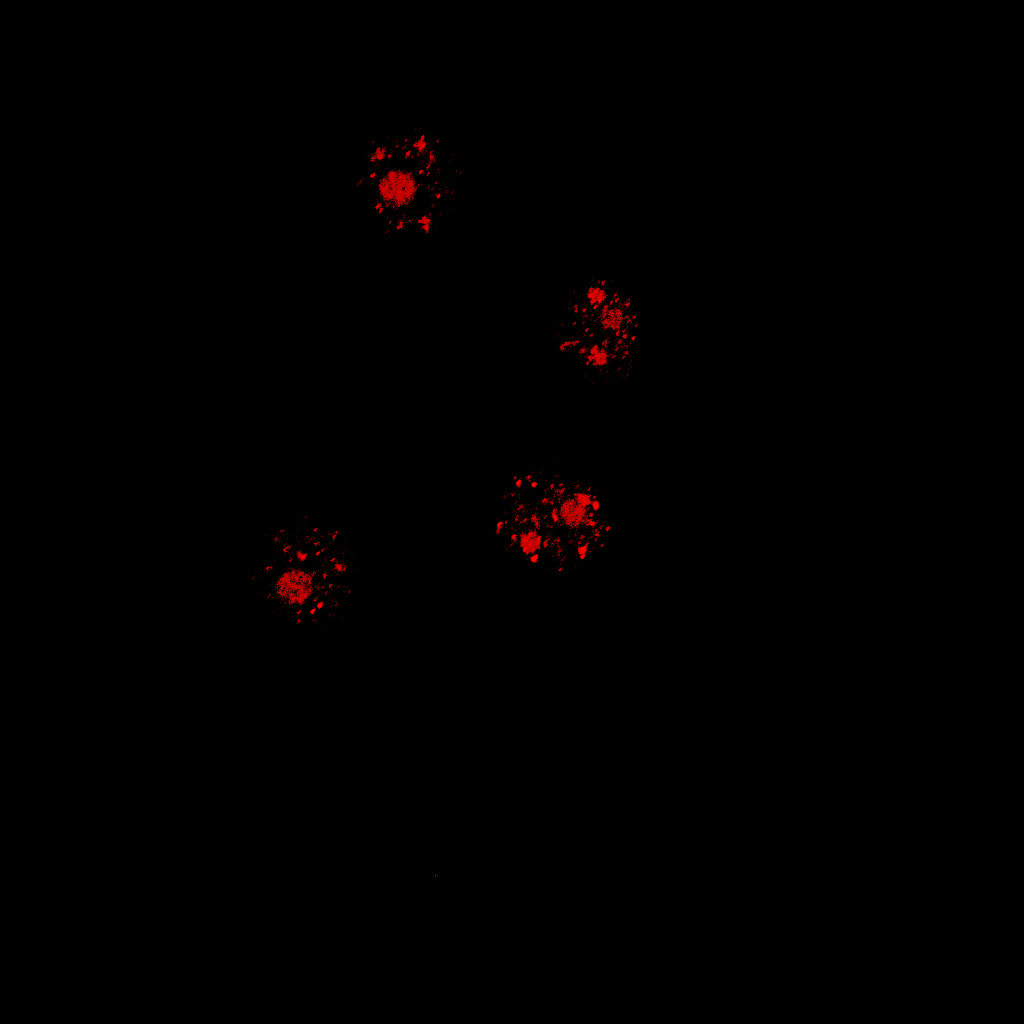

Supplement: Supplementary file 9 — Expanded View Figure and Appendix source data [file 44319_2025_513_MOESM9_ESM.zip › Expanded View Figure and Appendix source data/Expanded View Figure 1/EV 1B/DMSO 8 h/RAD51.tif]

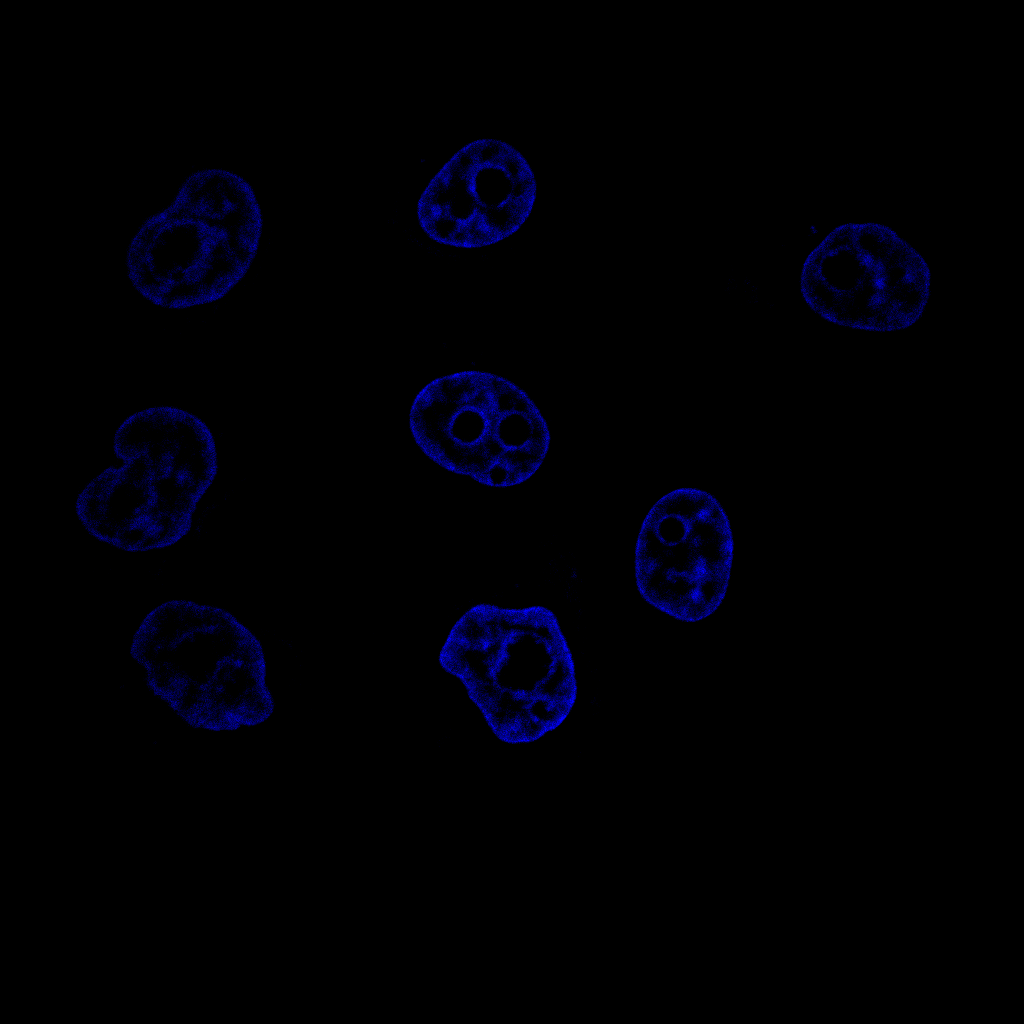

Supplement: Supplementary file 9 — Expanded View Figure and Appendix source data [file 44319_2025_513_MOESM9_ESM.zip › Expanded View Figure and Appendix source data/Expanded View Figure 1/EV 1B/DMSO ETO-/DAPI.tif]

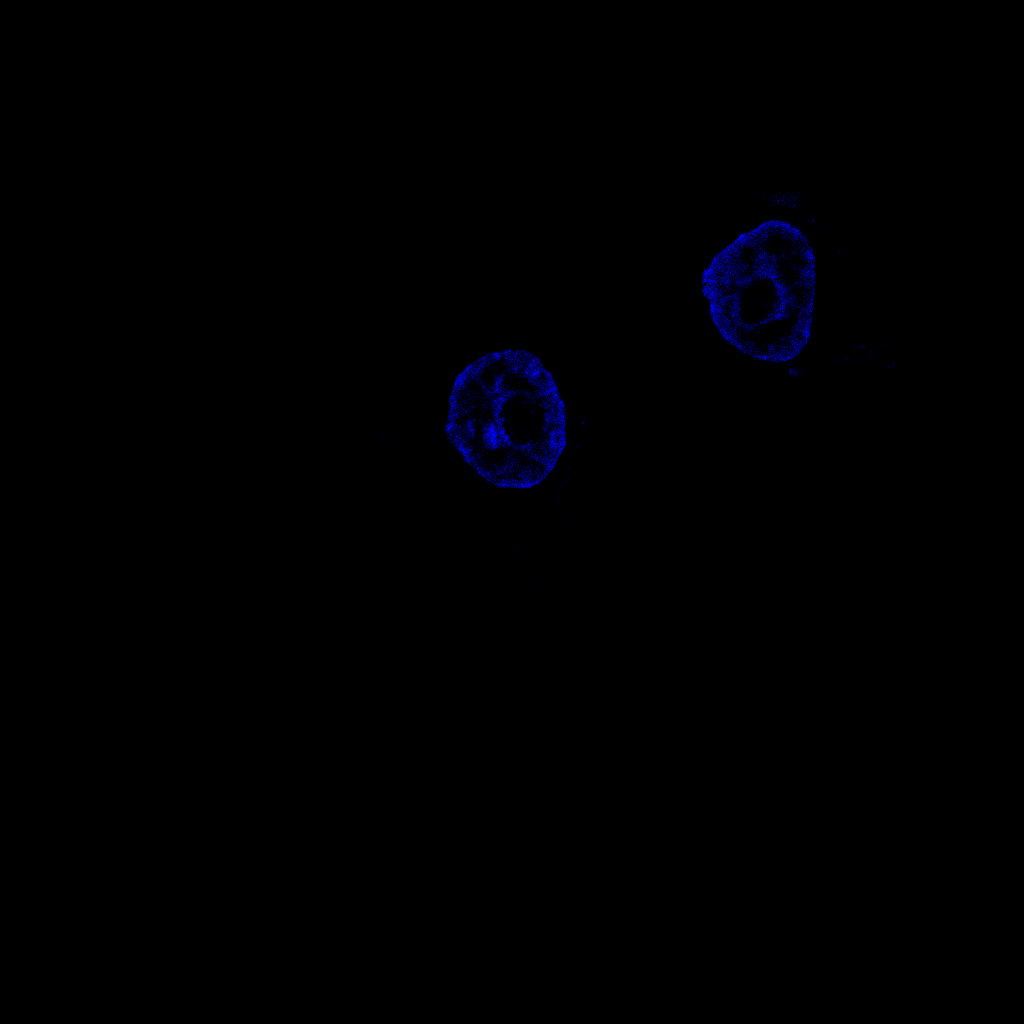

Supplement: Supplementary file 9 — Expanded View Figure and Appendix source data [file 44319_2025_513_MOESM9_ESM.zip › Expanded View Figure and Appendix source data/Expanded View Figure 1/EV 1B/TSA 1 h/DAPI.tif]

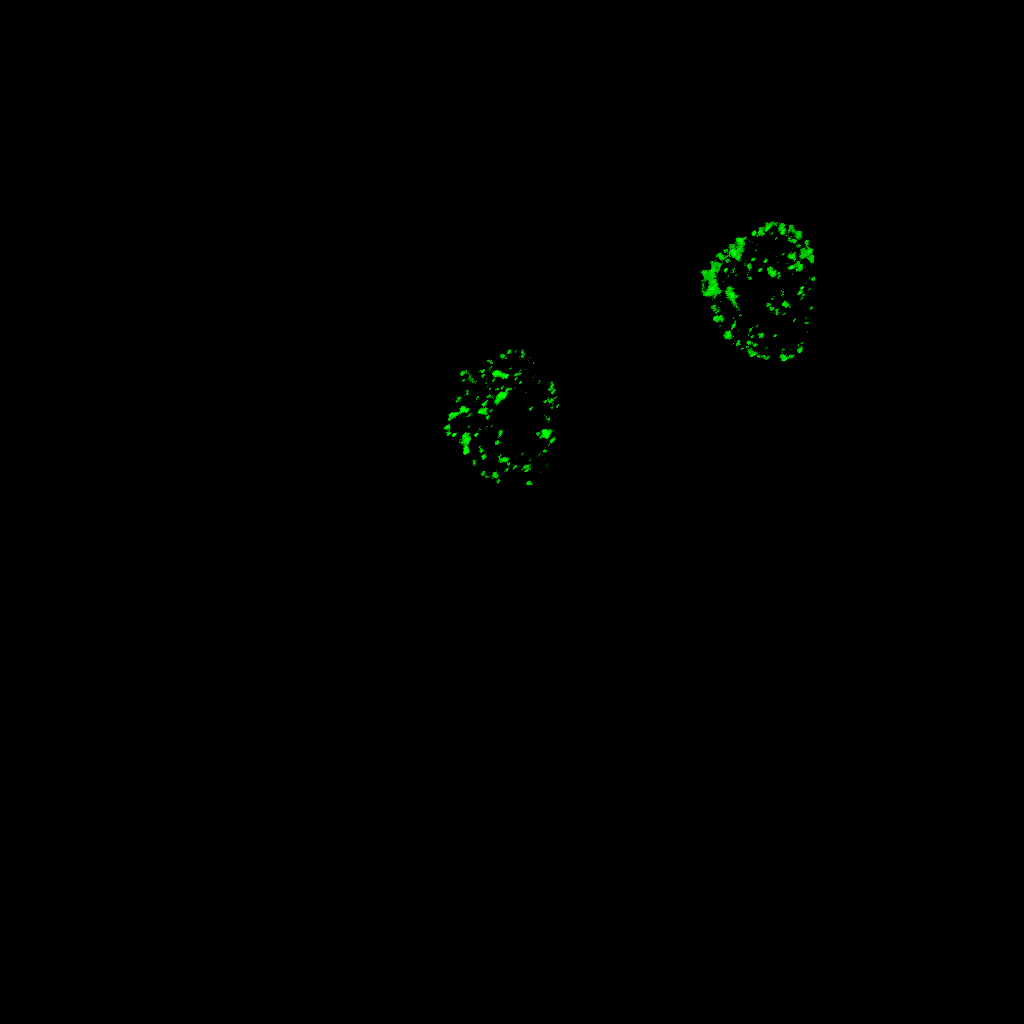

Supplement: Supplementary file 9 — Expanded View Figure and Appendix source data [file 44319_2025_513_MOESM9_ESM.zip › Expanded View Figure and Appendix source data/Expanded View Figure 1/EV 1B/TSA 1 h/GH2AX.tif]

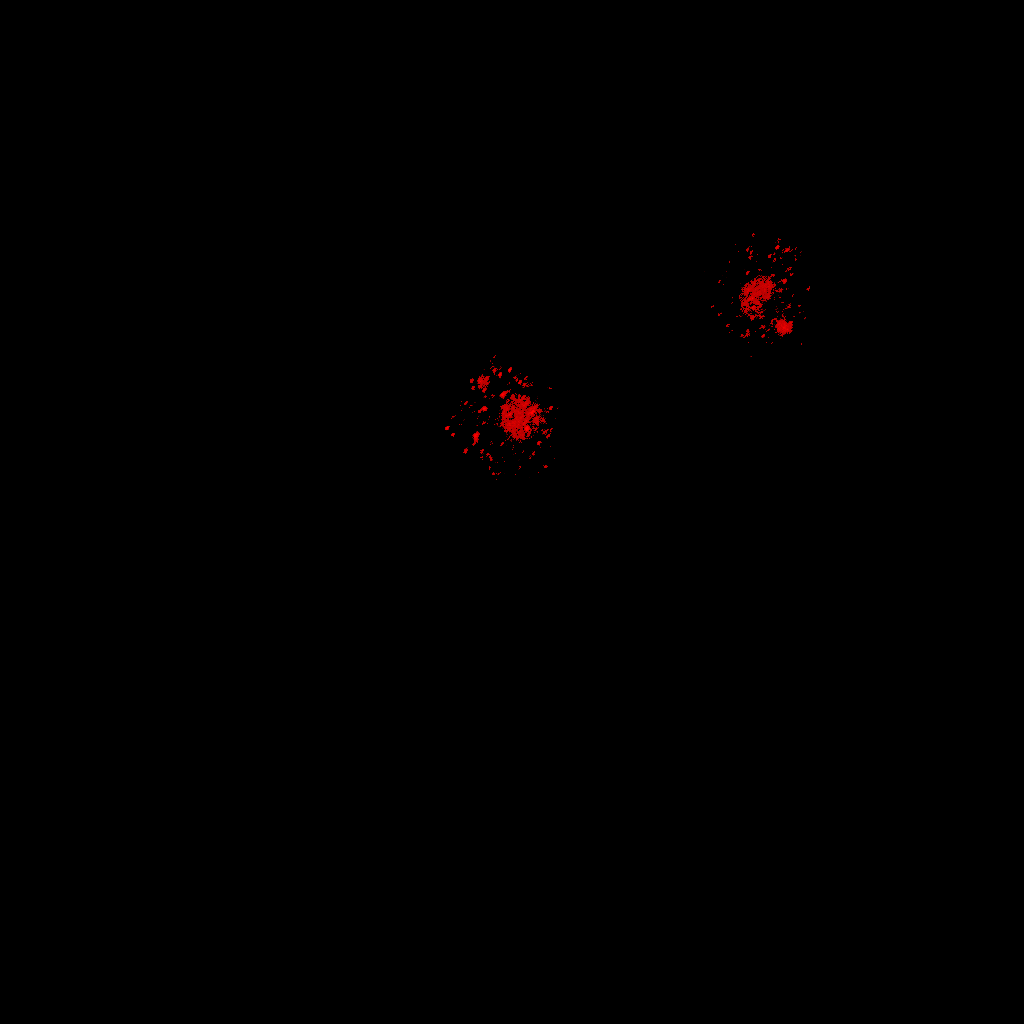

Supplement: Supplementary file 9 — Expanded View Figure and Appendix source data [file 44319_2025_513_MOESM9_ESM.zip › Expanded View Figure and Appendix source data/Expanded View Figure 1/EV 1B/TSA 1 h/RAD51.tif]

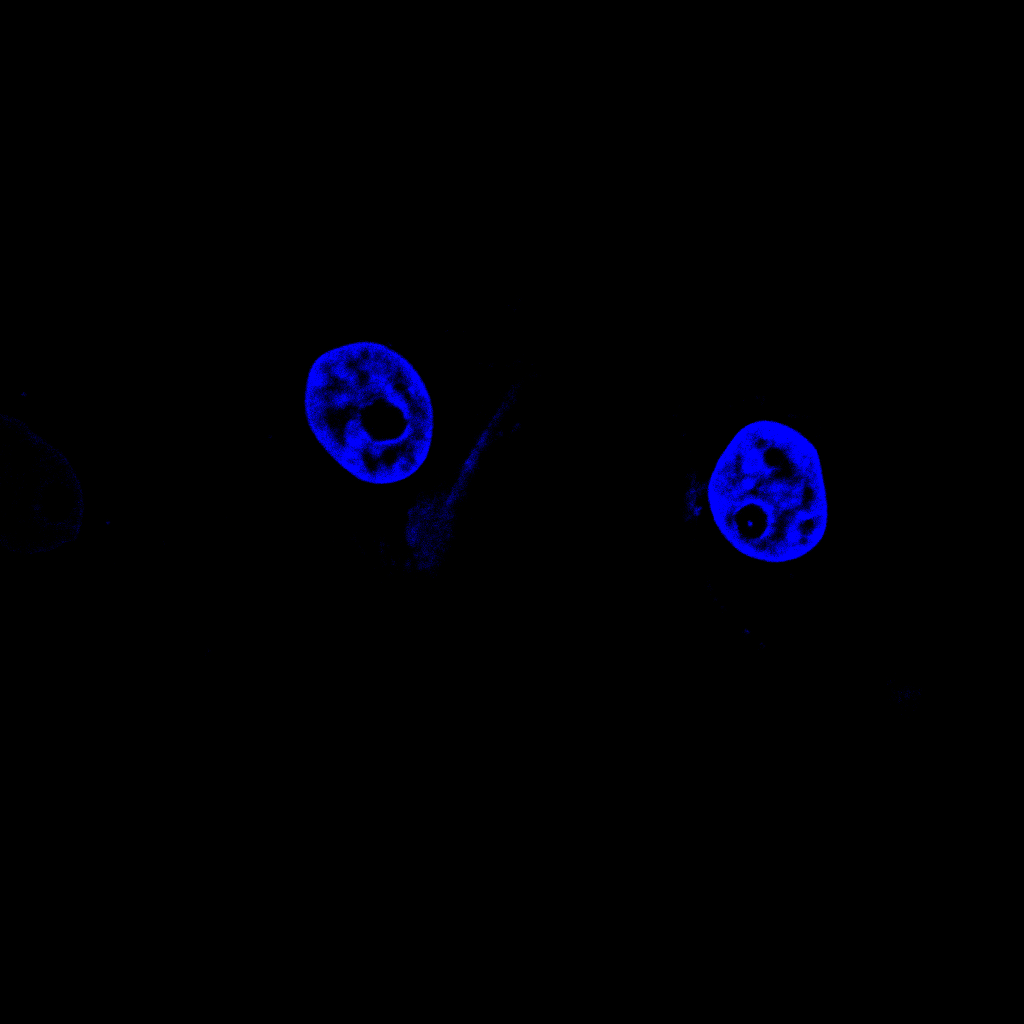

Supplement: Supplementary file 9 — Expanded View Figure and Appendix source data [file 44319_2025_513_MOESM9_ESM.zip › Expanded View Figure and Appendix source data/Expanded View Figure 1/EV 1B/TSA 4 h/DAPI.tif]

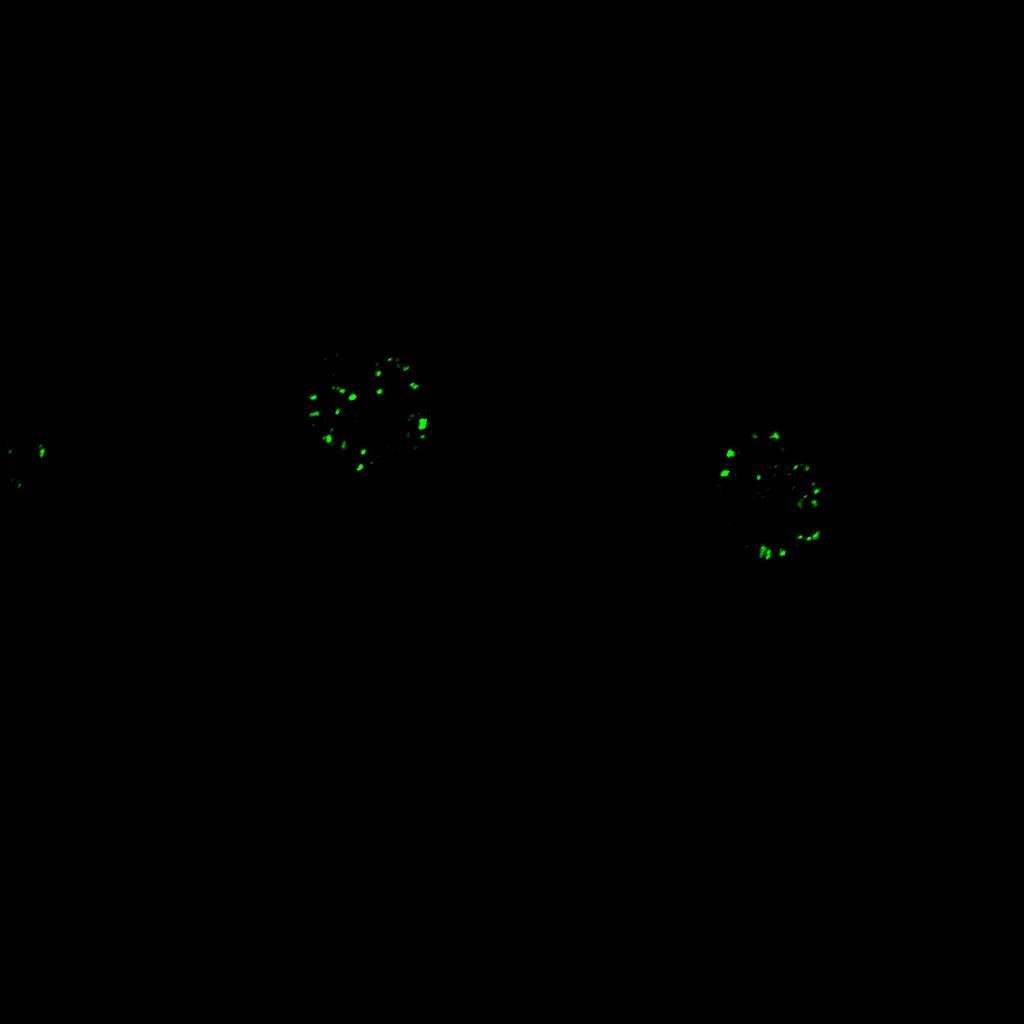

Supplement: Supplementary file 9 — Expanded View Figure and Appendix source data [file 44319_2025_513_MOESM9_ESM.zip › Expanded View Figure and Appendix source data/Expanded View Figure 1/EV 1B/TSA 4 h/GH2AX.tif]

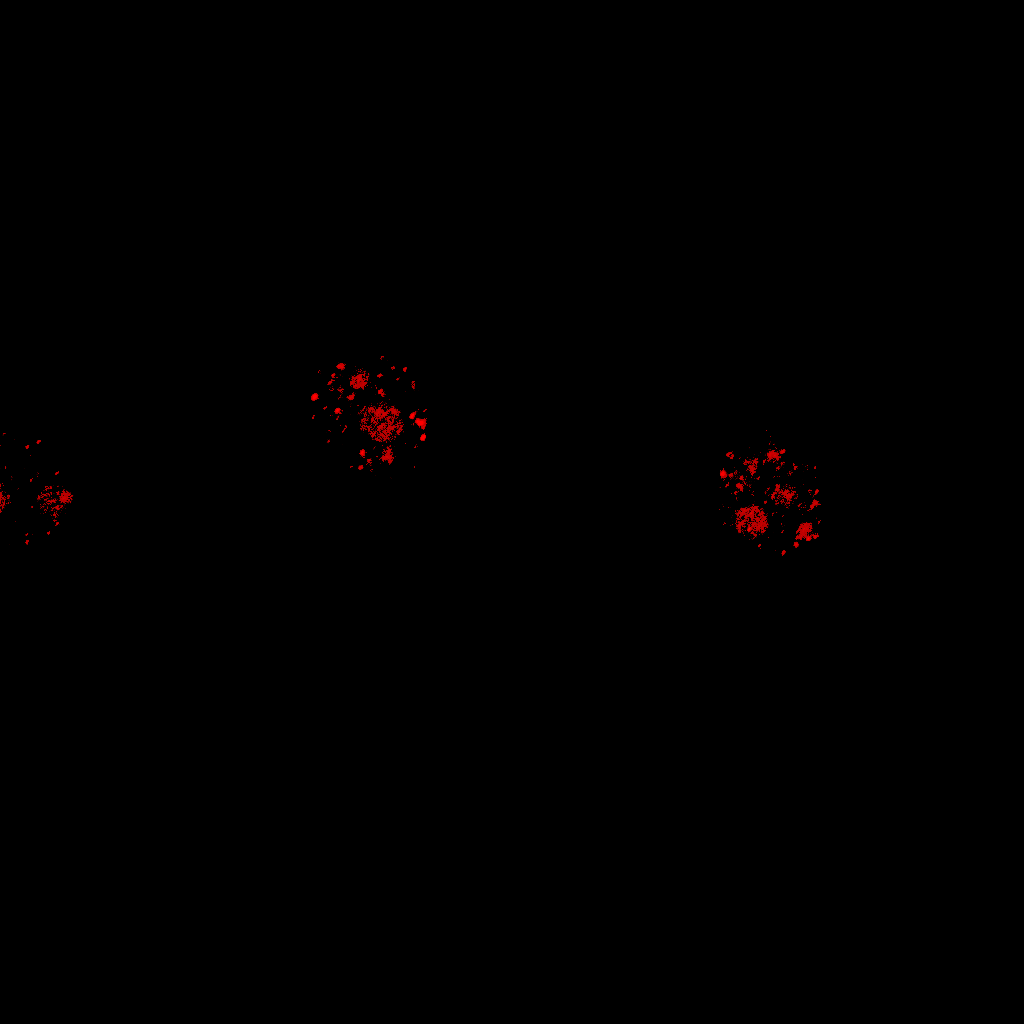

Supplement: Supplementary file 9 — Expanded View Figure and Appendix source data [file 44319_2025_513_MOESM9_ESM.zip › Expanded View Figure and Appendix source data/Expanded View Figure 1/EV 1B/TSA 4 h/RAD51.tif]

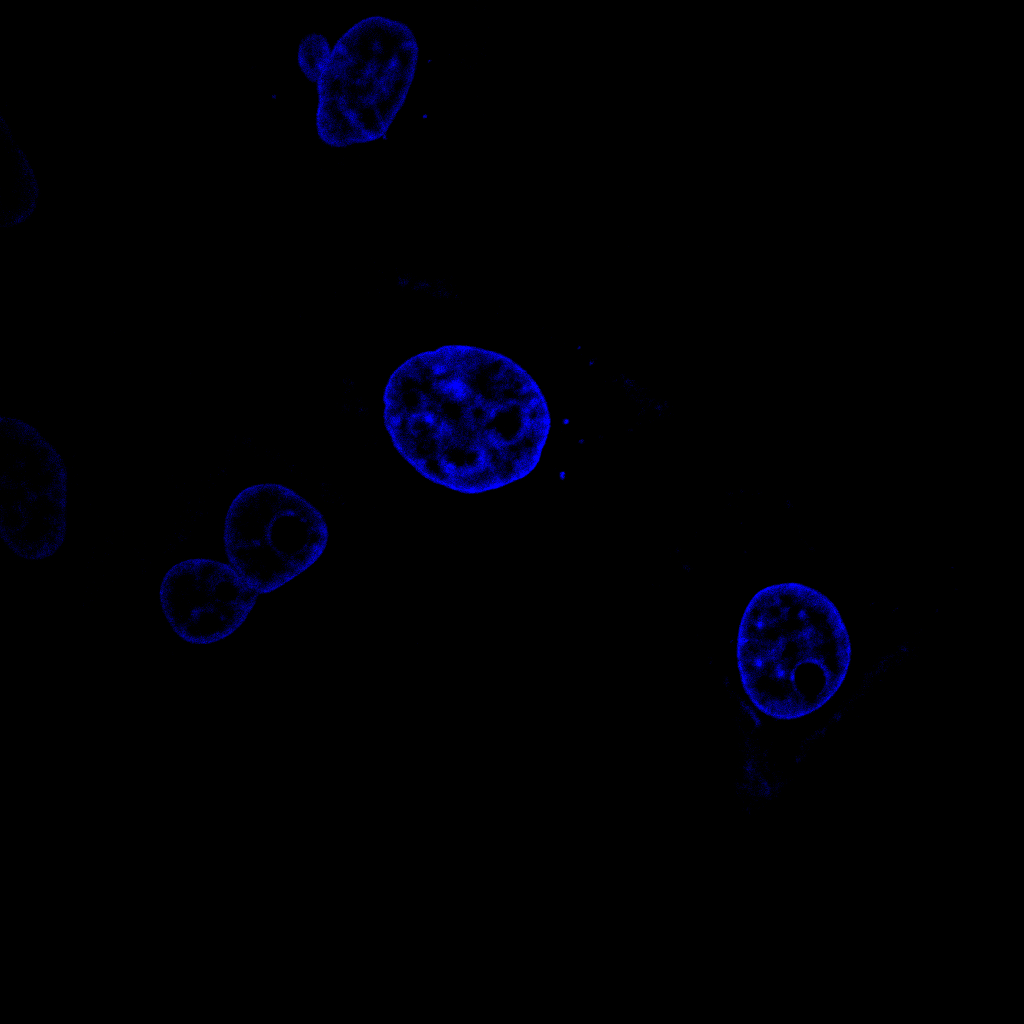

Supplement: Supplementary file 9 — Expanded View Figure and Appendix source data [file 44319_2025_513_MOESM9_ESM.zip › Expanded View Figure and Appendix source data/Expanded View Figure 1/EV 1B/TSA 8 h/DAPI.tif]

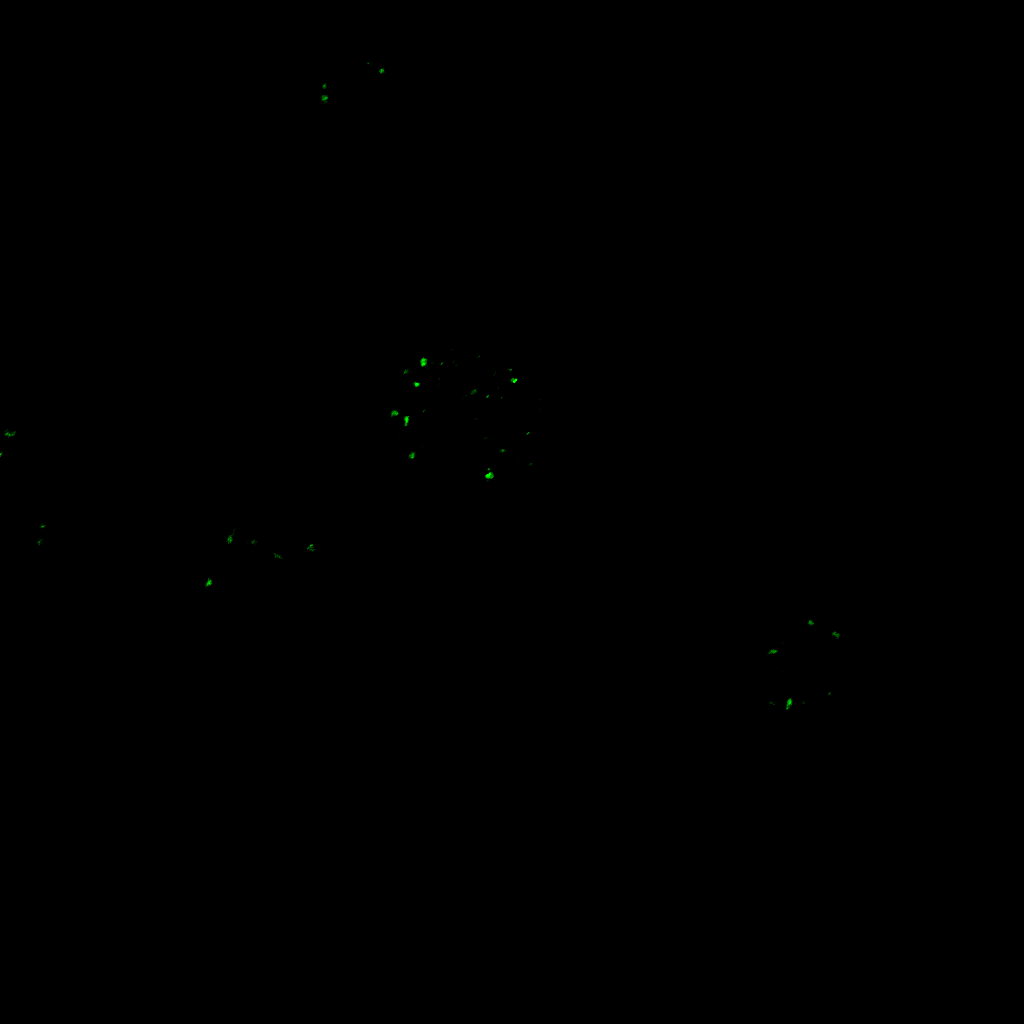

Supplement: Supplementary file 9 — Expanded View Figure and Appendix source data [file 44319_2025_513_MOESM9_ESM.zip › Expanded View Figure and Appendix source data/Expanded View Figure 1/EV 1B/TSA 8 h/GH2AX.tif]

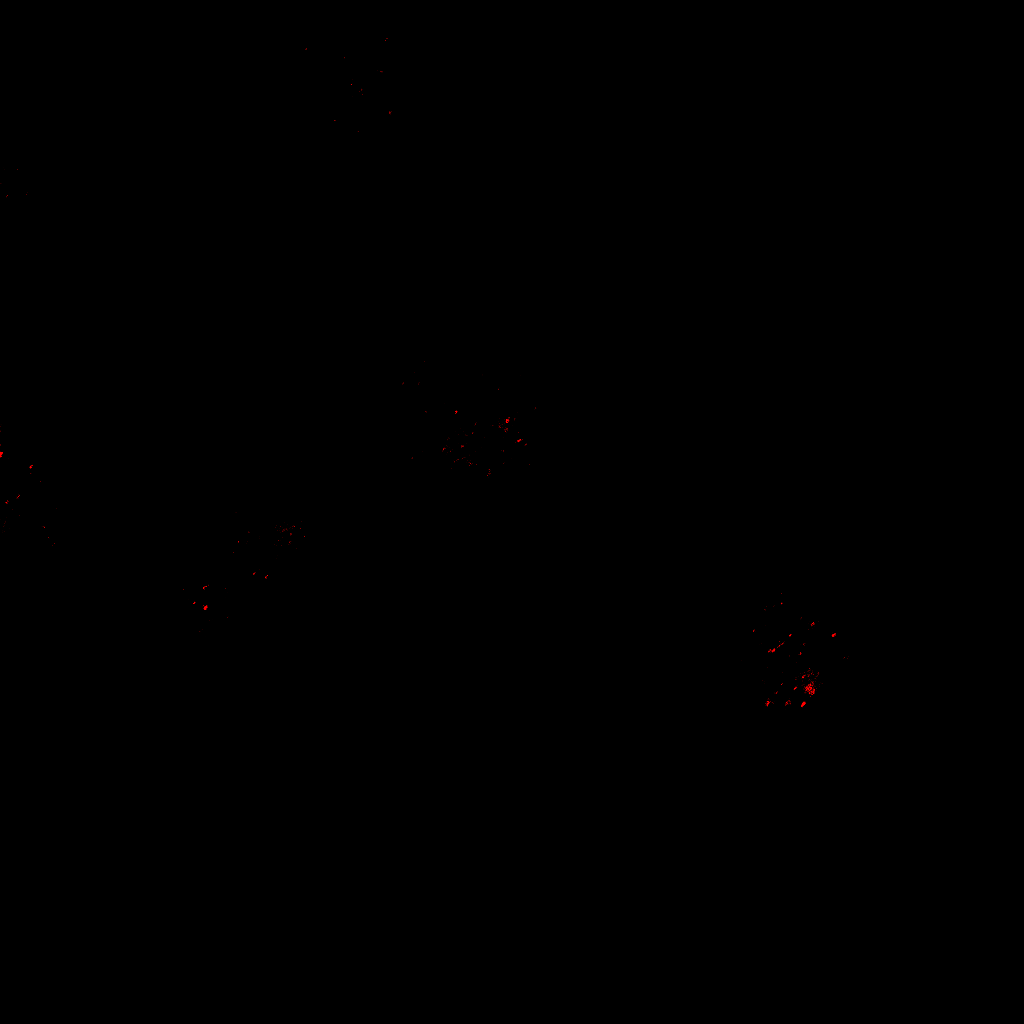

Supplement: Supplementary file 9 — Expanded View Figure and Appendix source data [file 44319_2025_513_MOESM9_ESM.zip › Expanded View Figure and Appendix source data/Expanded View Figure 1/EV 1B/TSA 8 h/RAD51.tif]

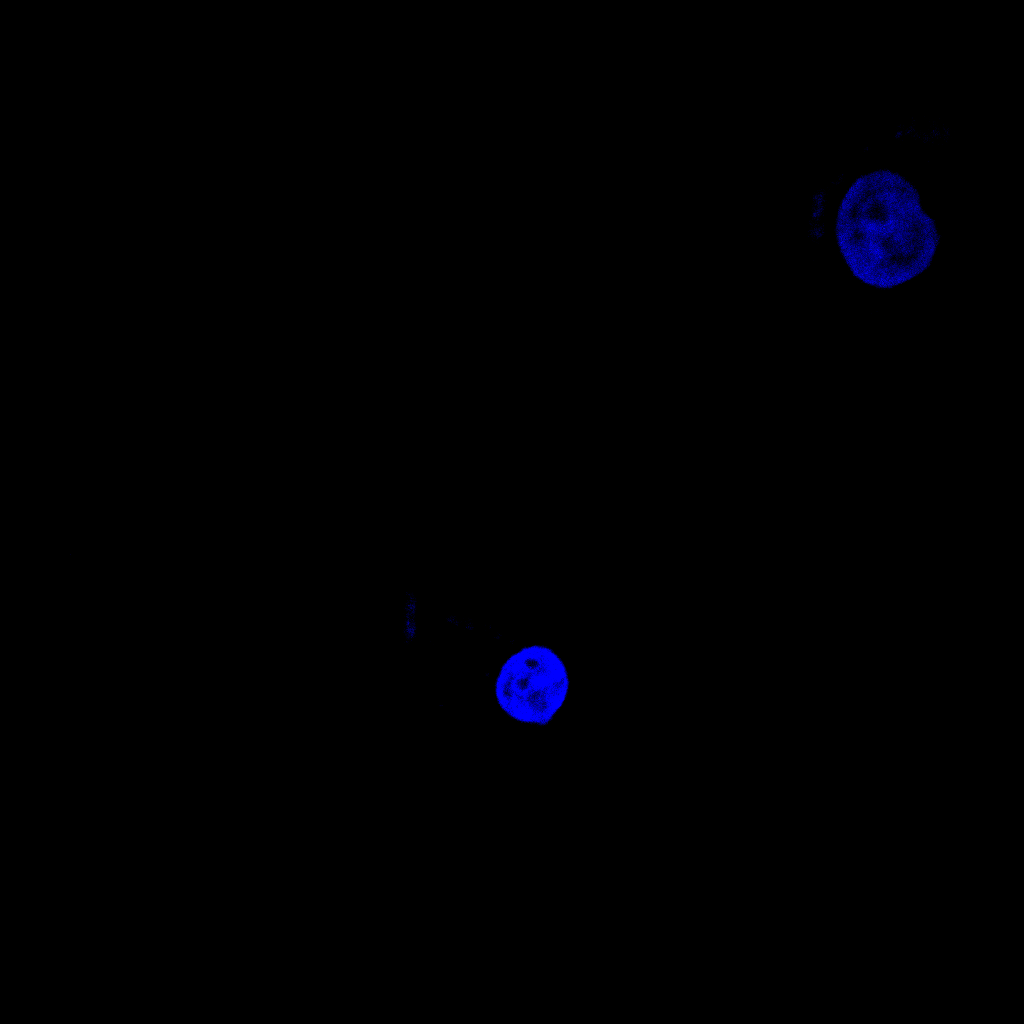

Supplement: Supplementary file 9 — Expanded View Figure and Appendix source data [file 44319_2025_513_MOESM9_ESM.zip › Expanded View Figure and Appendix source data/Expanded View Figure 1/EV 1B/TSA ETO-/DAPI.tif]

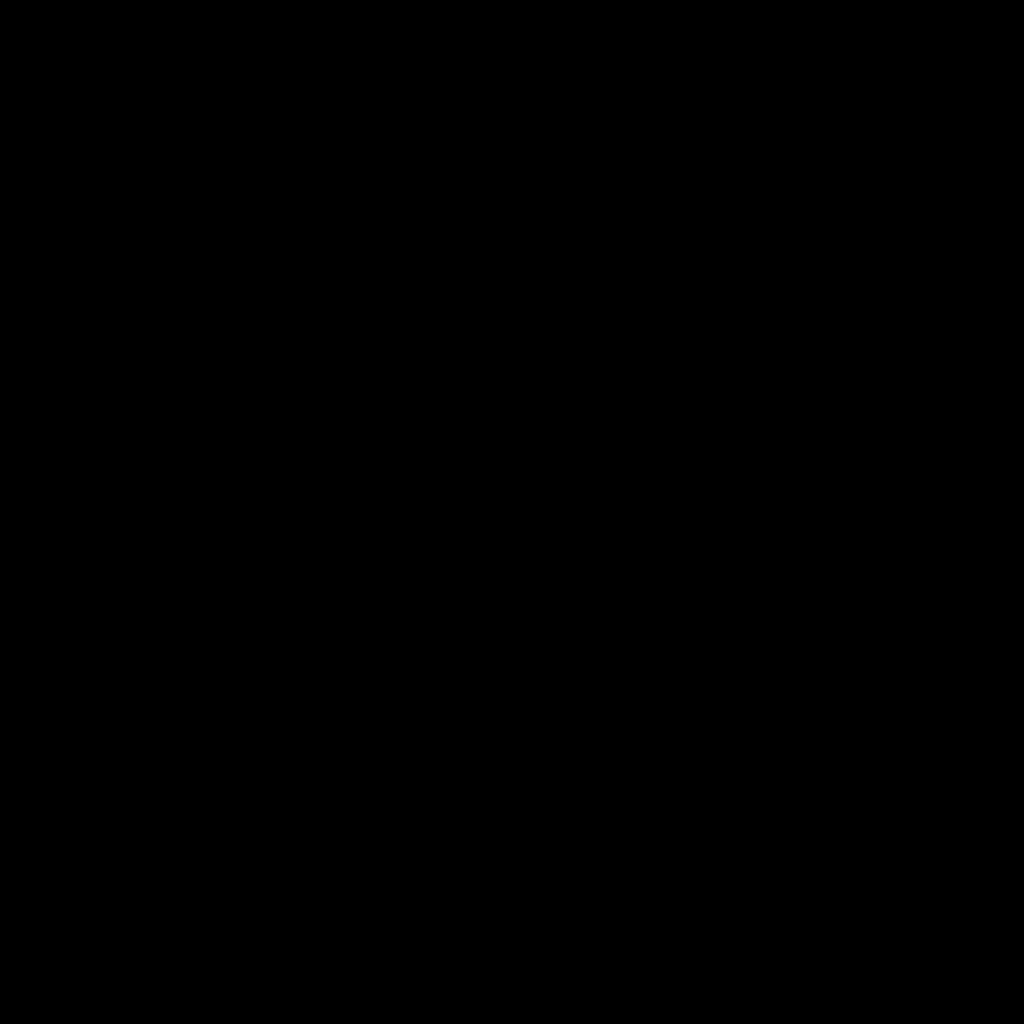

Supplement: Supplementary file 9 — Expanded View Figure and Appendix source data [file 44319_2025_513_MOESM9_ESM.zip › Expanded View Figure and Appendix source data/Expanded View Figure 1/EV 1B/TSA ETO-/GH2AX.tif]

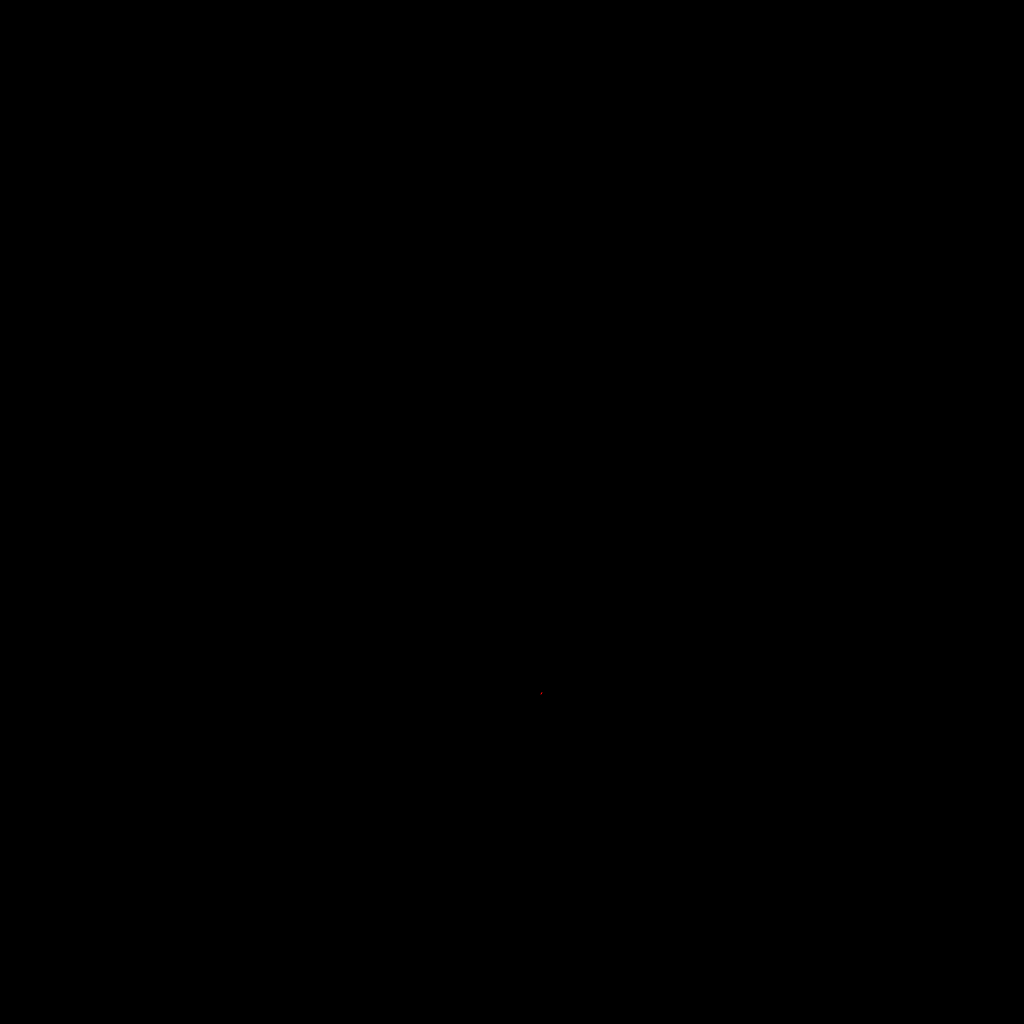

Supplement: Supplementary file 9 — Expanded View Figure and Appendix source data [file 44319_2025_513_MOESM9_ESM.zip › Expanded View Figure and Appendix source data/Expanded View Figure 1/EV 1B/TSA ETO-/RAD51.tif]

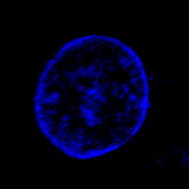

Supplement: Supplementary file 9 — Expanded View Figure and Appendix source data [file 44319_2025_513_MOESM9_ESM.zip › Expanded View Figure and Appendix source data/Expanded View Figure 4/EV 4E/5KR ETO+/DAPI.tif]

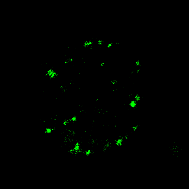

Supplement: Supplementary file 9 — Expanded View Figure and Appendix source data [file 44319_2025_513_MOESM9_ESM.zip › Expanded View Figure and Appendix source data/Expanded View Figure 4/EV 4E/5KR ETO+/GH2AX.tif]

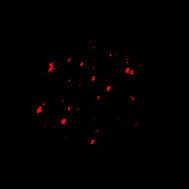

Supplement: Supplementary file 9 — Expanded View Figure and Appendix source data [file 44319_2025_513_MOESM9_ESM.zip › Expanded View Figure and Appendix source data/Expanded View Figure 4/EV 4E/5KR ETO+/RAD51.tif]

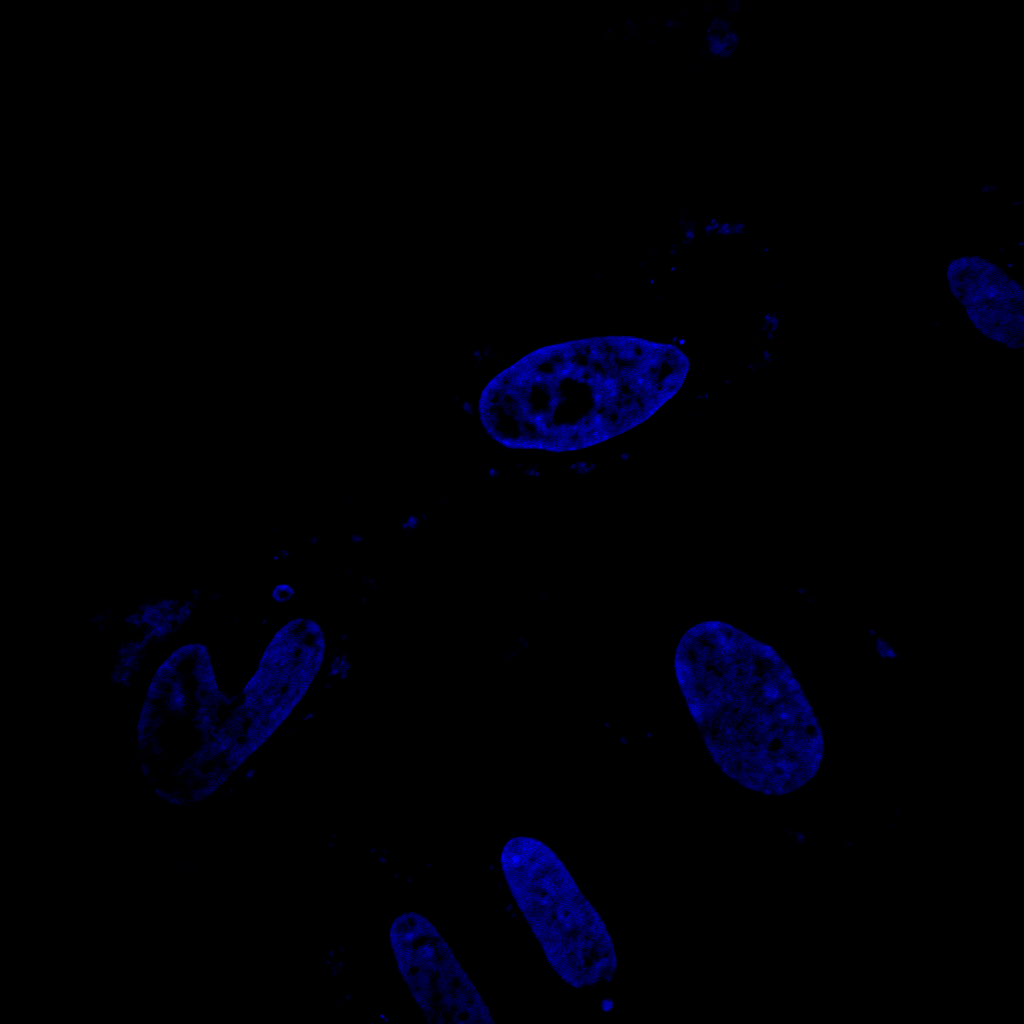

Supplement: Supplementary file 9 — Expanded View Figure and Appendix source data [file 44319_2025_513_MOESM9_ESM.zip › Expanded View Figure and Appendix source data/Expanded View Figure 4/EV 4E/5KR ETO-/DAPI.tif]

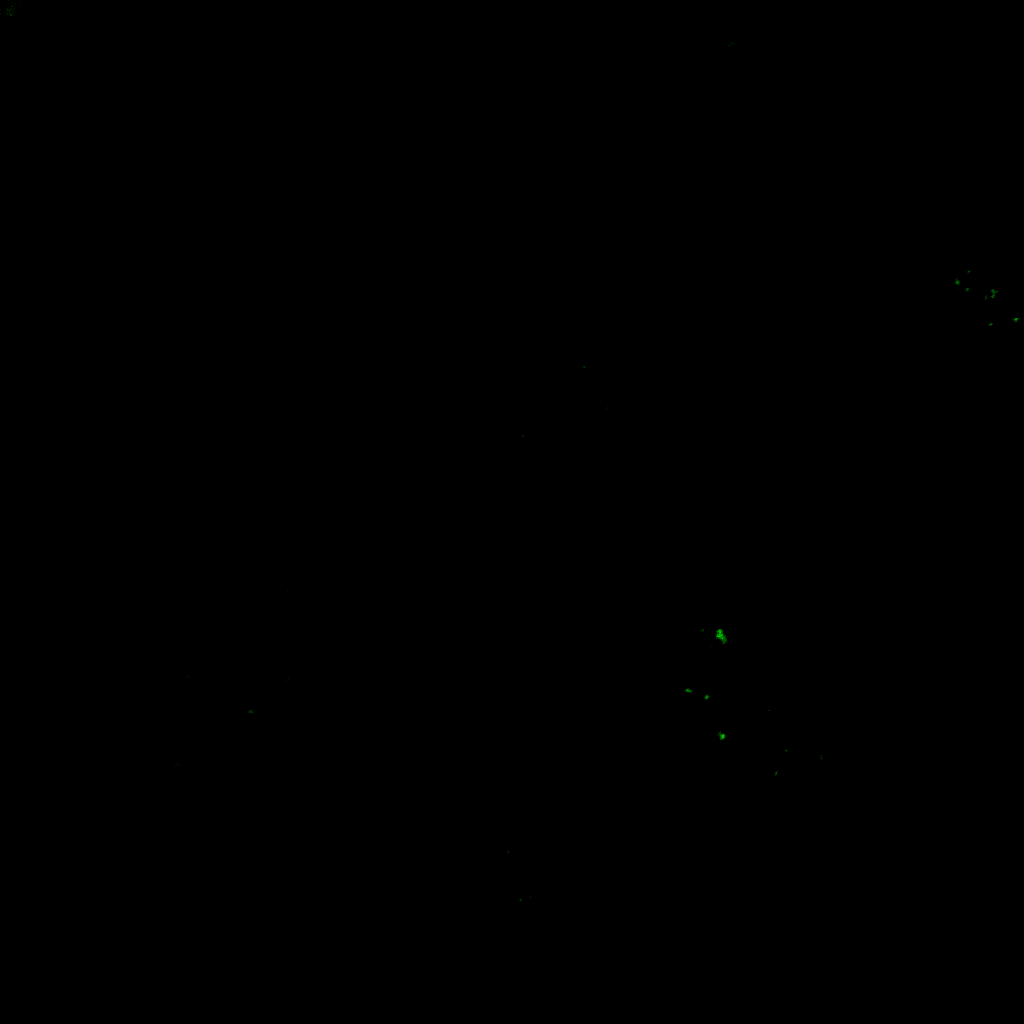

Supplement: Supplementary file 9 — Expanded View Figure and Appendix source data [file 44319_2025_513_MOESM9_ESM.zip › Expanded View Figure and Appendix source data/Expanded View Figure 4/EV 4E/5KR ETO-/GH2AX.tif]

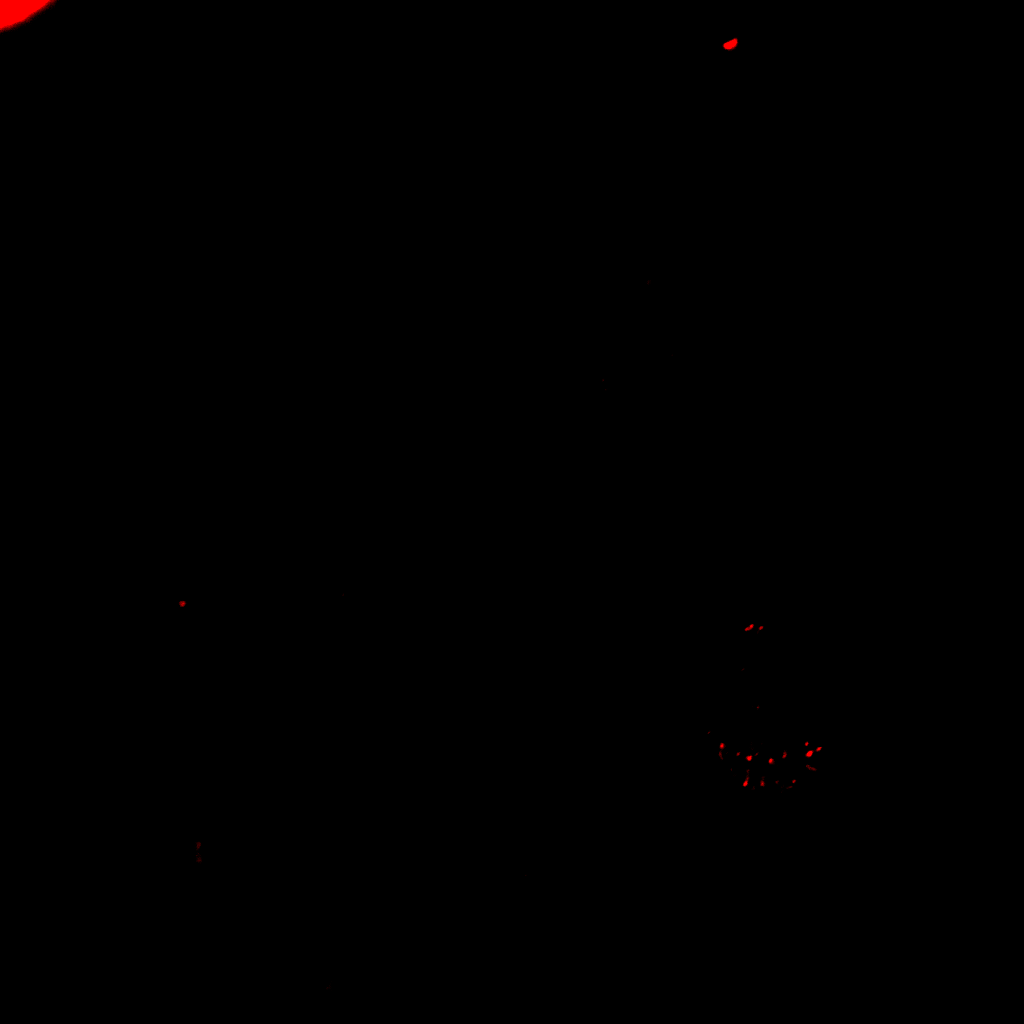

Supplement: Supplementary file 9 — Expanded View Figure and Appendix source data [file 44319_2025_513_MOESM9_ESM.zip › Expanded View Figure and Appendix source data/Expanded View Figure 4/EV 4E/5KR ETO-/RAD51.tif]

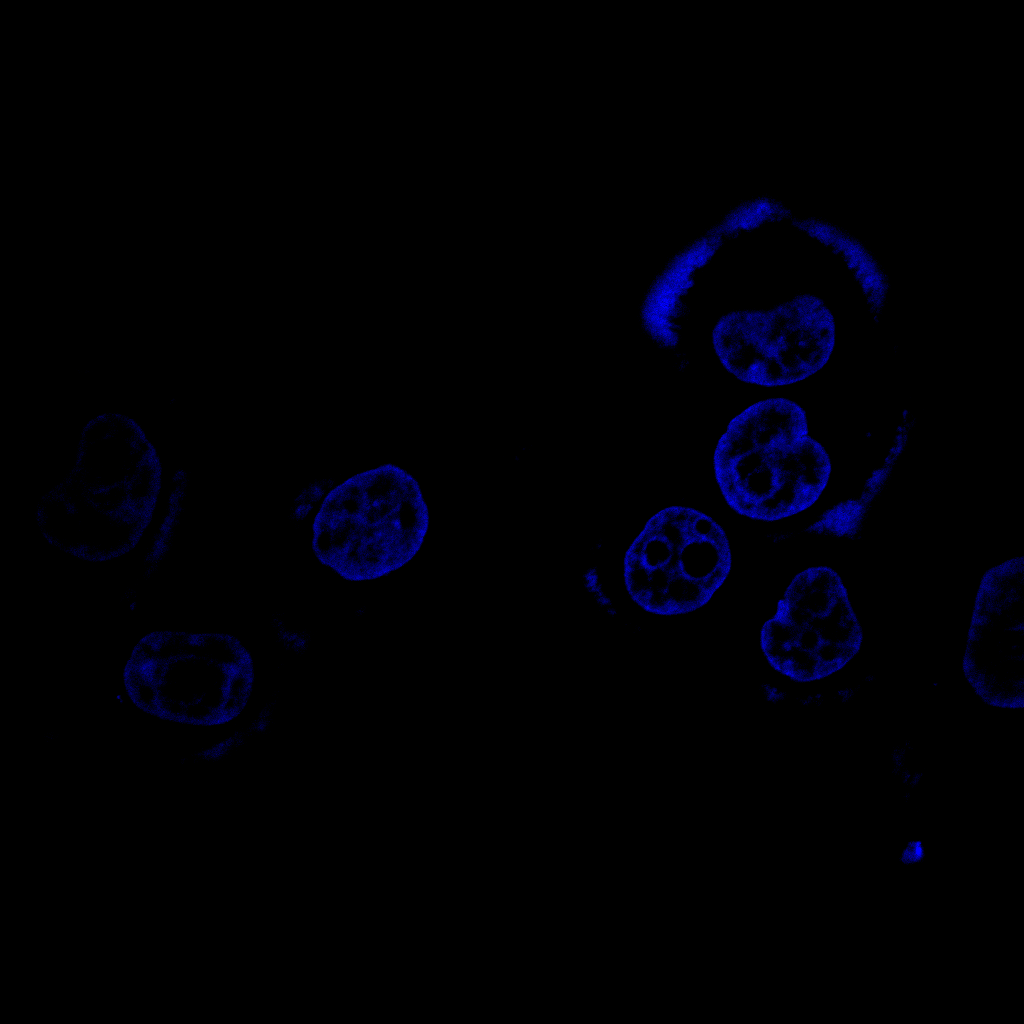

Supplement: Supplementary file 9 — Expanded View Figure and Appendix source data [file 44319_2025_513_MOESM9_ESM.zip › Expanded View Figure and Appendix source data/Expanded View Figure 4/EV 4E/EV ETO+/DAPI.tif]

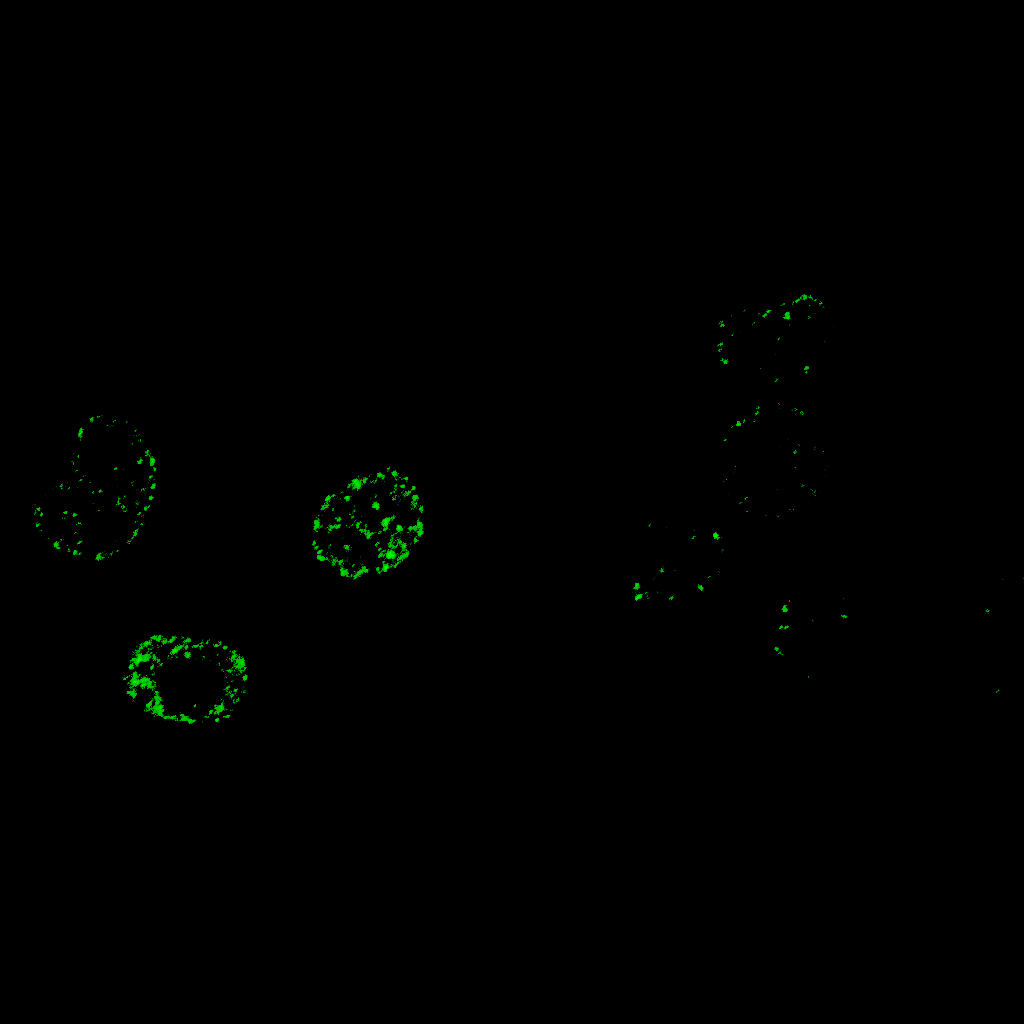

Supplement: Supplementary file 9 — Expanded View Figure and Appendix source data [file 44319_2025_513_MOESM9_ESM.zip › Expanded View Figure and Appendix source data/Expanded View Figure 4/EV 4E/EV ETO+/GH2AX.tif]

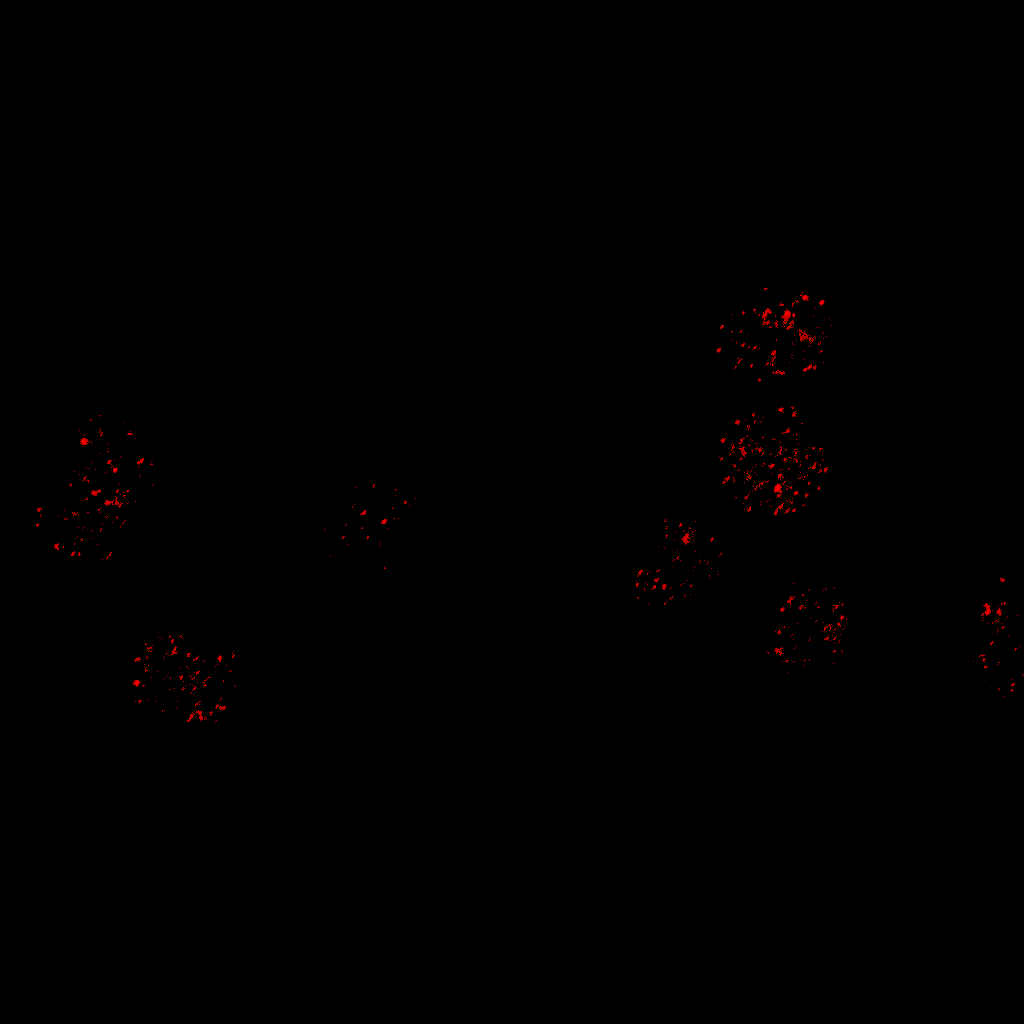

Supplement: Supplementary file 9 — Expanded View Figure and Appendix source data [file 44319_2025_513_MOESM9_ESM.zip › Expanded View Figure and Appendix source data/Expanded View Figure 4/EV 4E/EV ETO+/RAD51.tif]

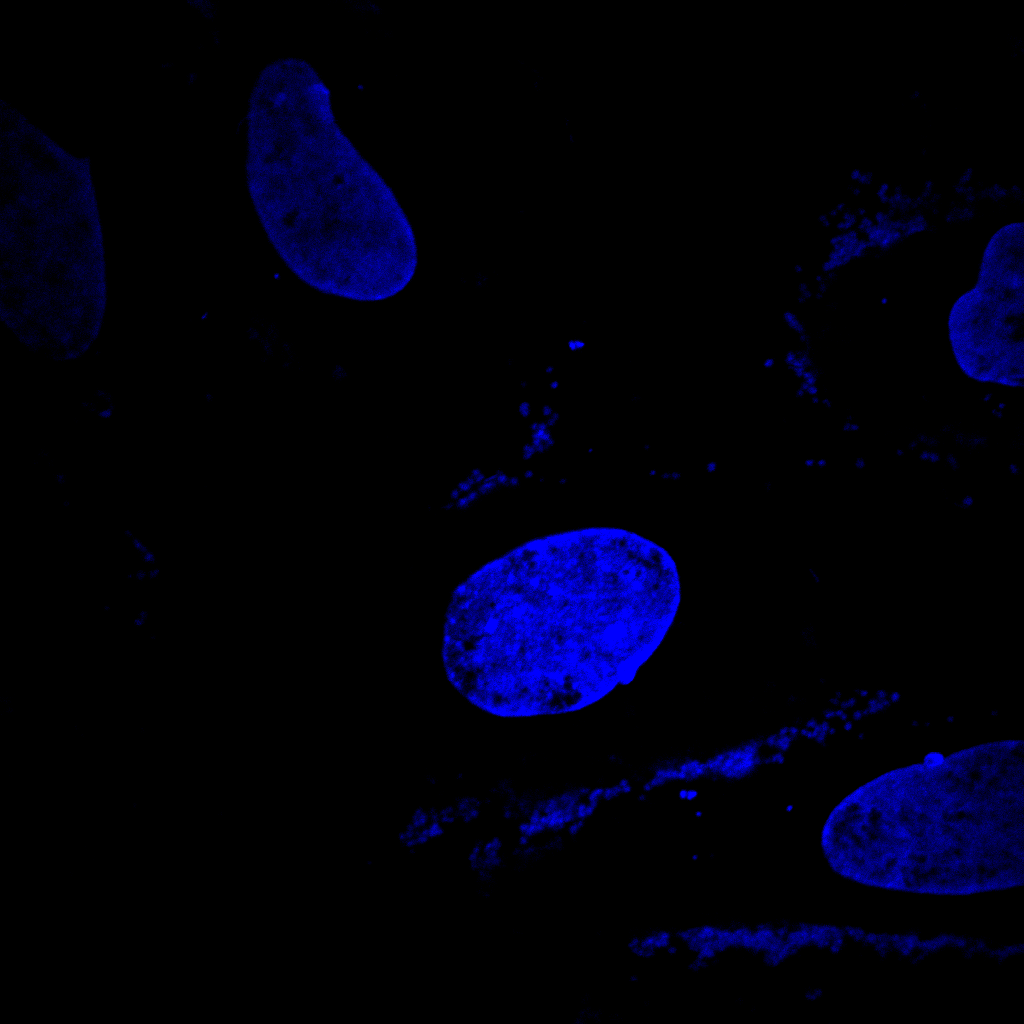

Supplement: Supplementary file 9 — Expanded View Figure and Appendix source data [file 44319_2025_513_MOESM9_ESM.zip › Expanded View Figure and Appendix source data/Expanded View Figure 4/EV 4E/EV ETO-/DAPI.tif]

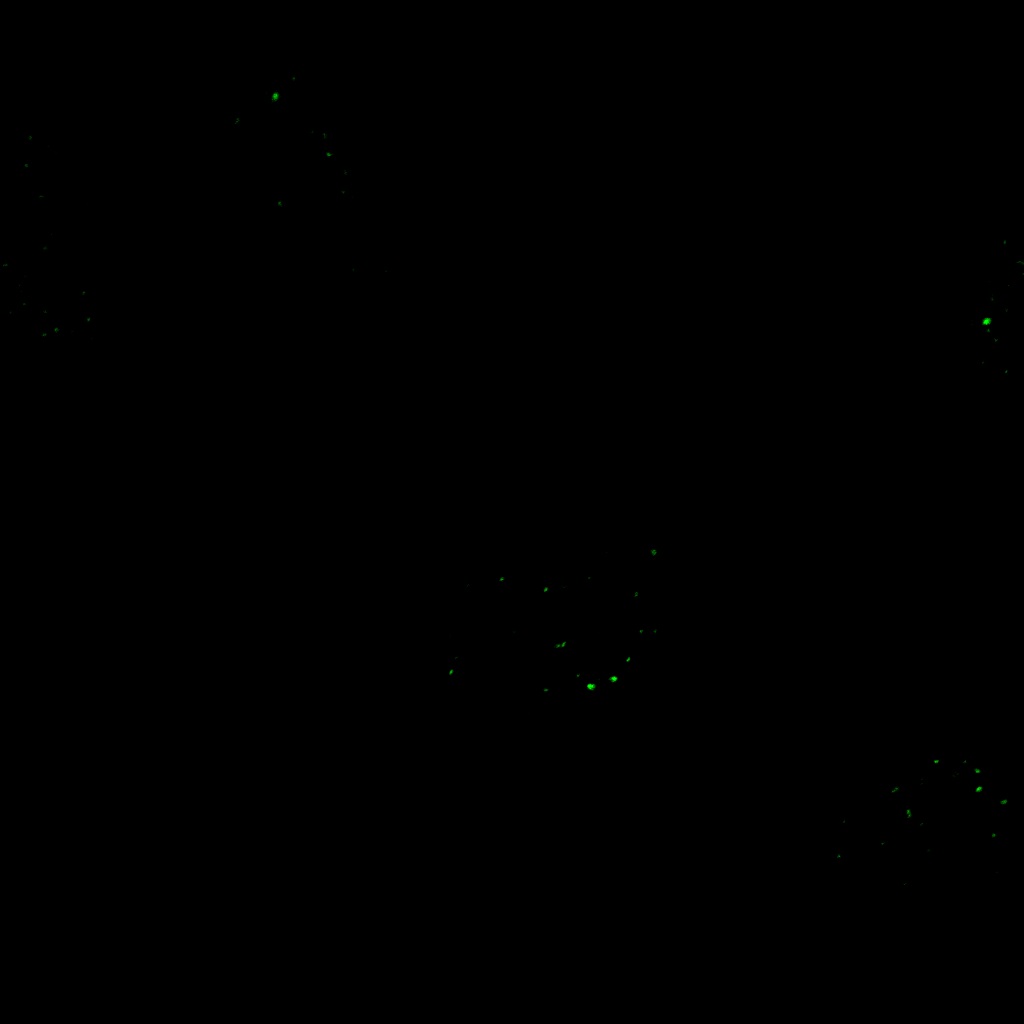

Supplement: Supplementary file 9 — Expanded View Figure and Appendix source data [file 44319_2025_513_MOESM9_ESM.zip › Expanded View Figure and Appendix source data/Expanded View Figure 4/EV 4E/EV ETO-/GH2AX.tif]

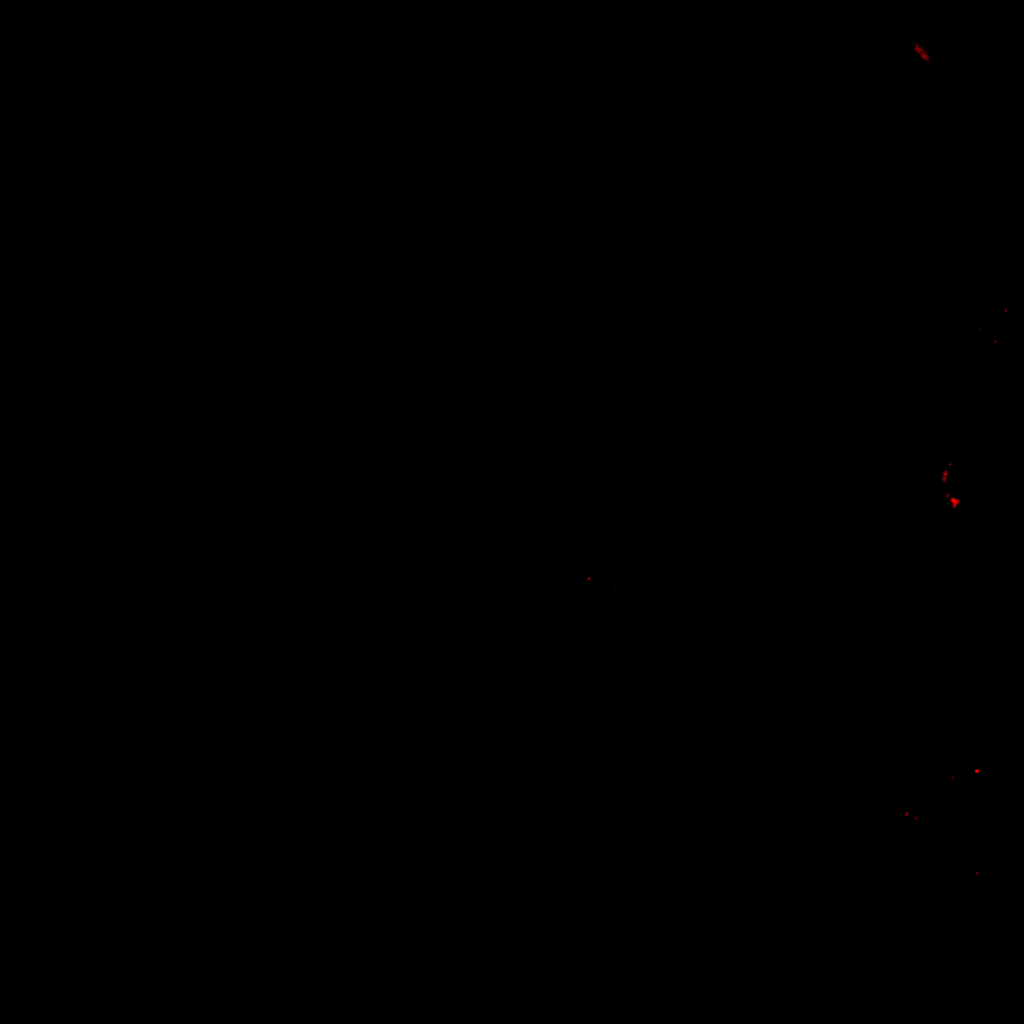

Supplement: Supplementary file 9 — Expanded View Figure and Appendix source data [file 44319_2025_513_MOESM9_ESM.zip › Expanded View Figure and Appendix source data/Expanded View Figure 4/EV 4E/EV ETO-/RAD51.tif]

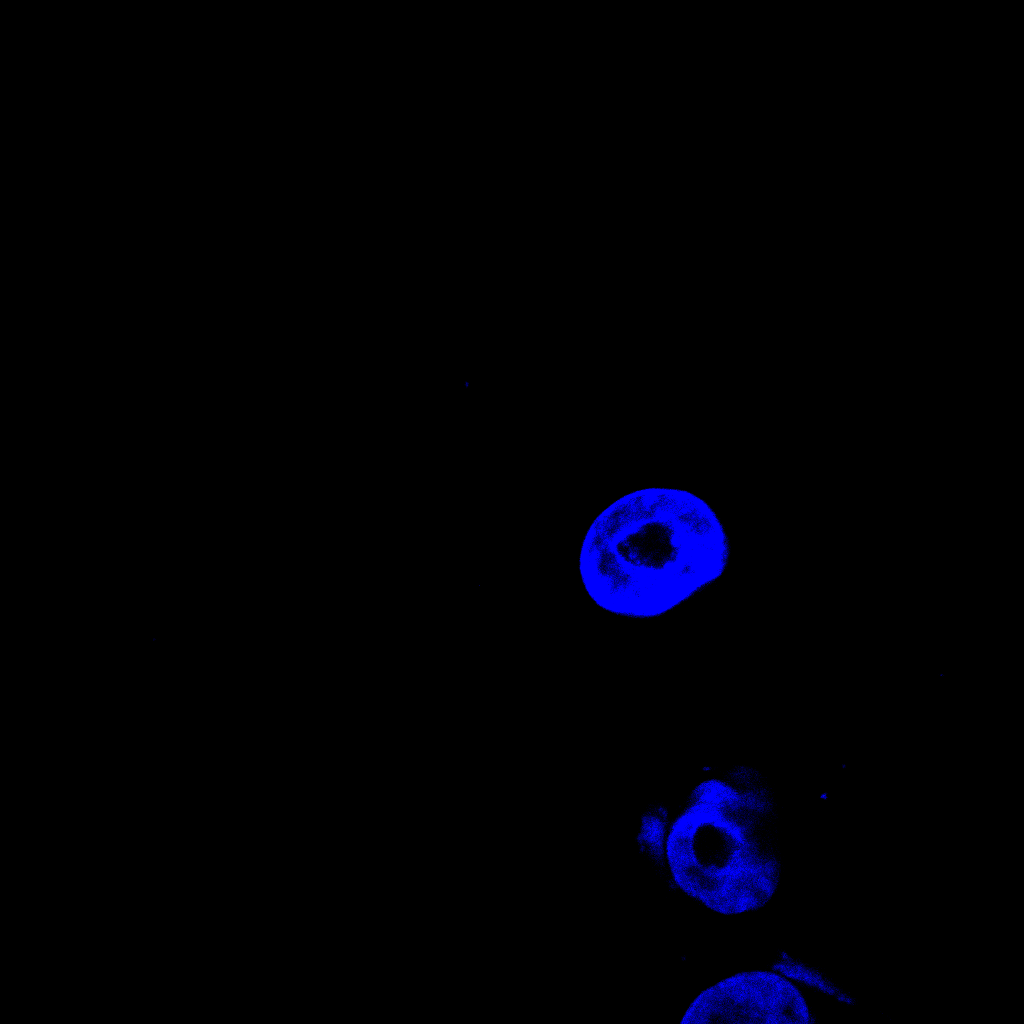

Supplement: Supplementary file 9 — Expanded View Figure and Appendix source data [file 44319_2025_513_MOESM9_ESM.zip › Expanded View Figure and Appendix source data/Expanded View Figure 4/EV 4E/NC ETO+/DAPI.tif]

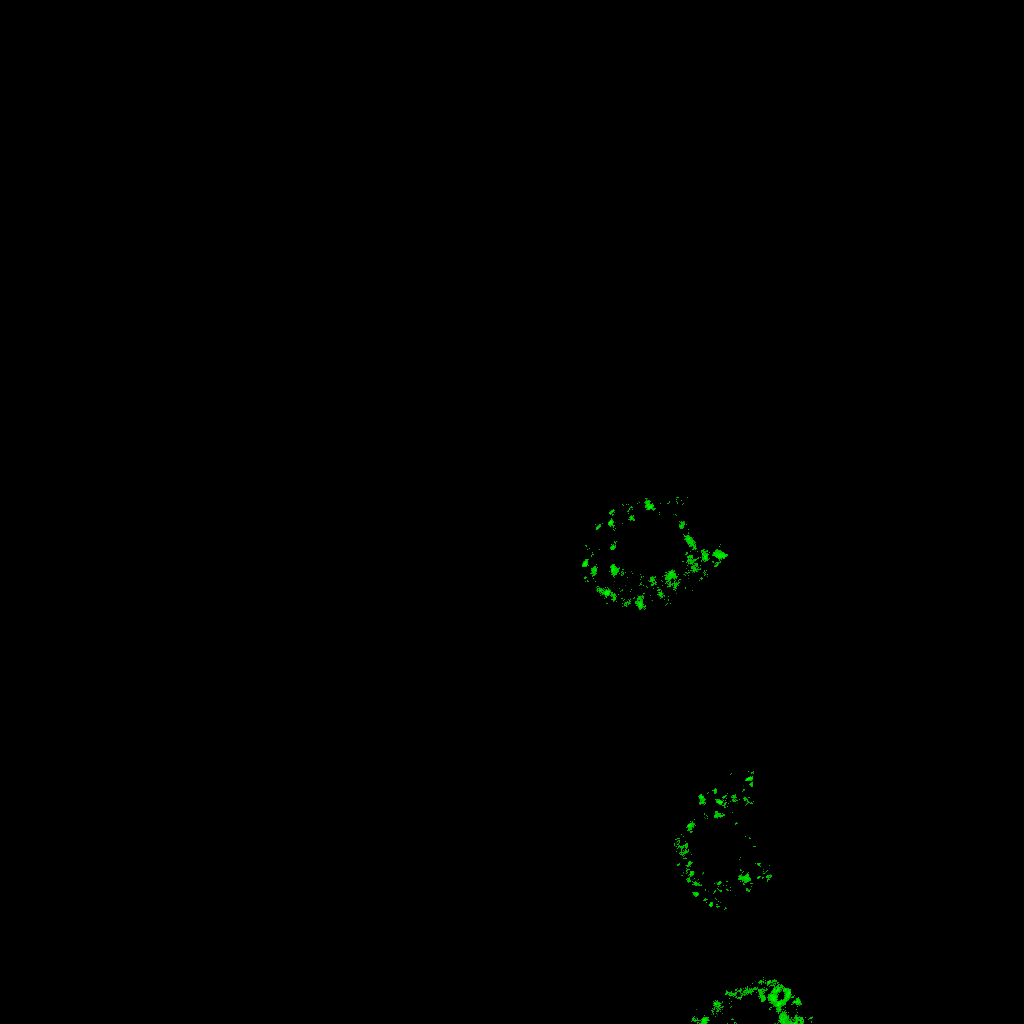

Supplement: Supplementary file 9 — Expanded View Figure and Appendix source data [file 44319_2025_513_MOESM9_ESM.zip › Expanded View Figure and Appendix source data/Expanded View Figure 4/EV 4E/NC ETO+/GH2AX.tif]

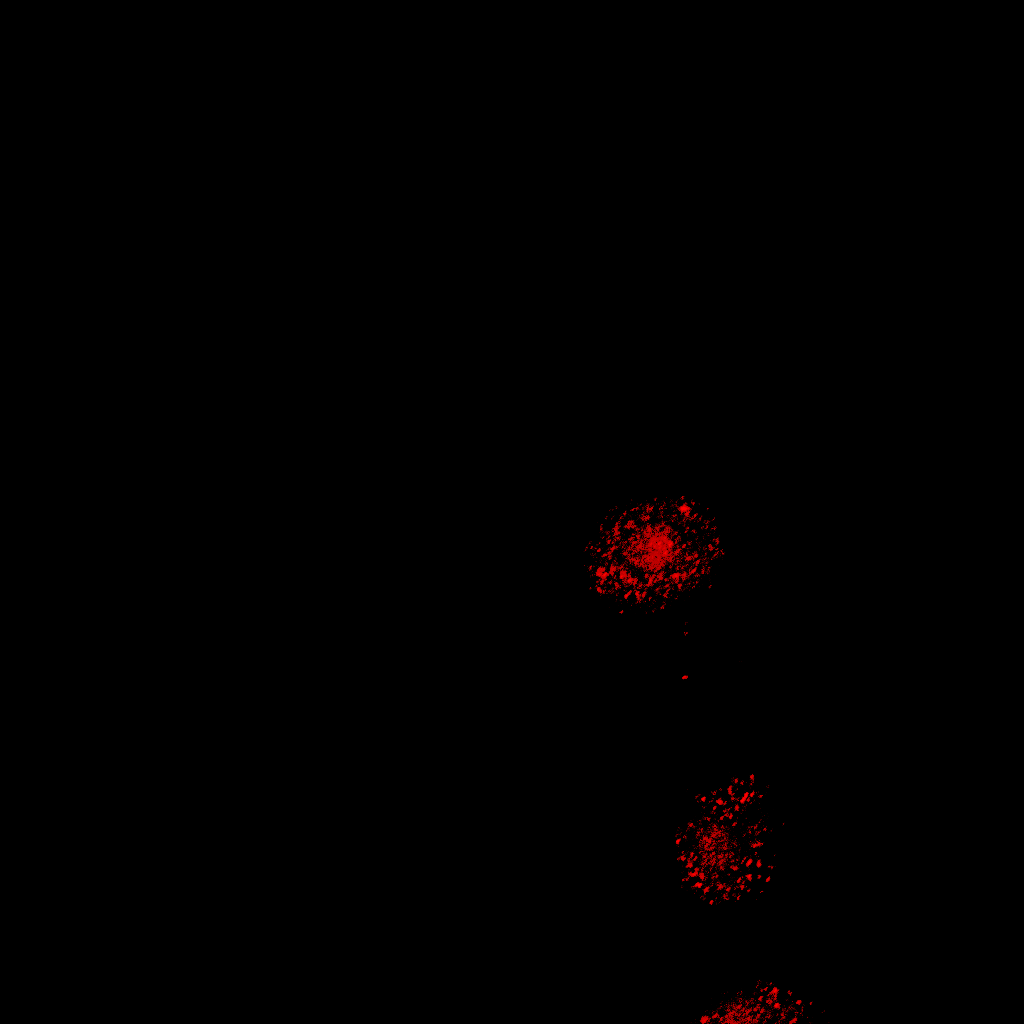

Supplement: Supplementary file 9 — Expanded View Figure and Appendix source data [file 44319_2025_513_MOESM9_ESM.zip › Expanded View Figure and Appendix source data/Expanded View Figure 4/EV 4E/NC ETO+/RAD51.tif]

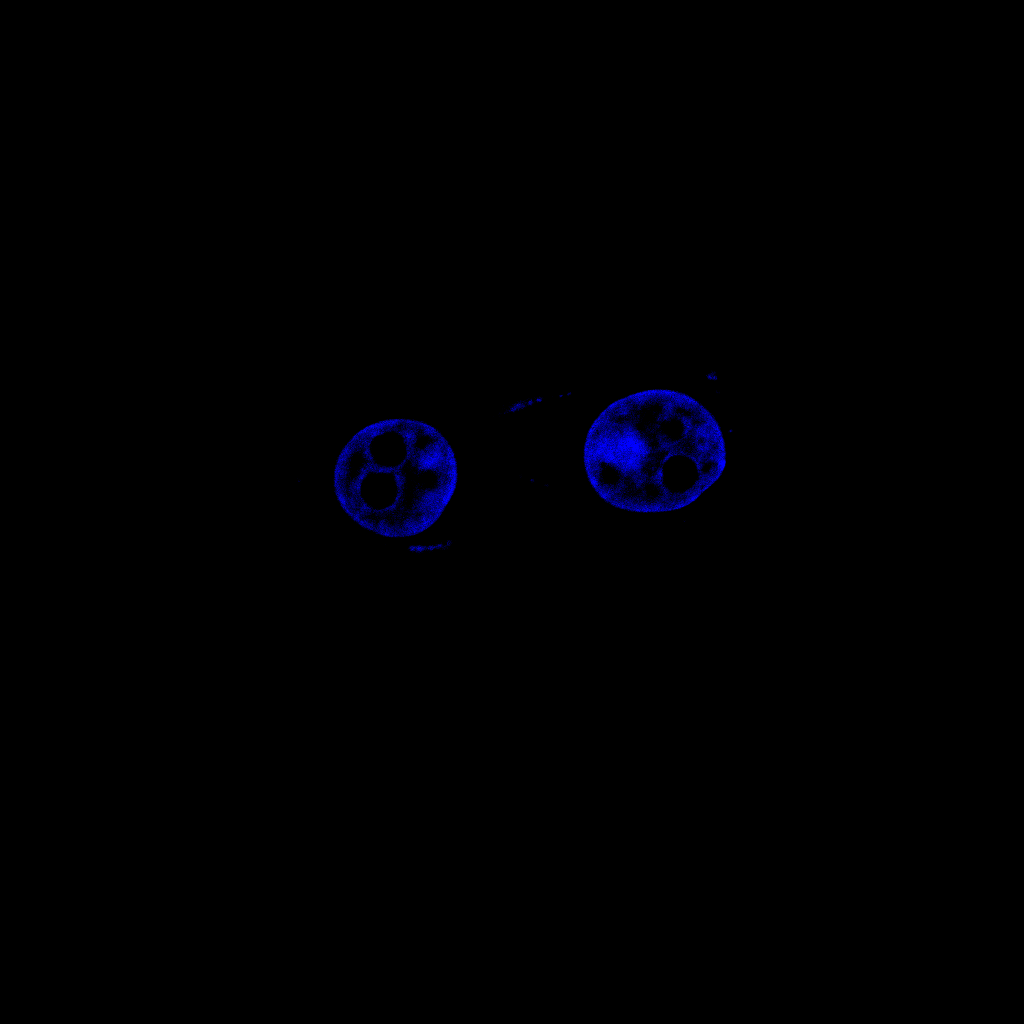

Supplement: Supplementary file 9 — Expanded View Figure and Appendix source data [file 44319_2025_513_MOESM9_ESM.zip › Expanded View Figure and Appendix source data/Expanded View Figure 4/EV 4E/NC ETO-/DAPI.tif]

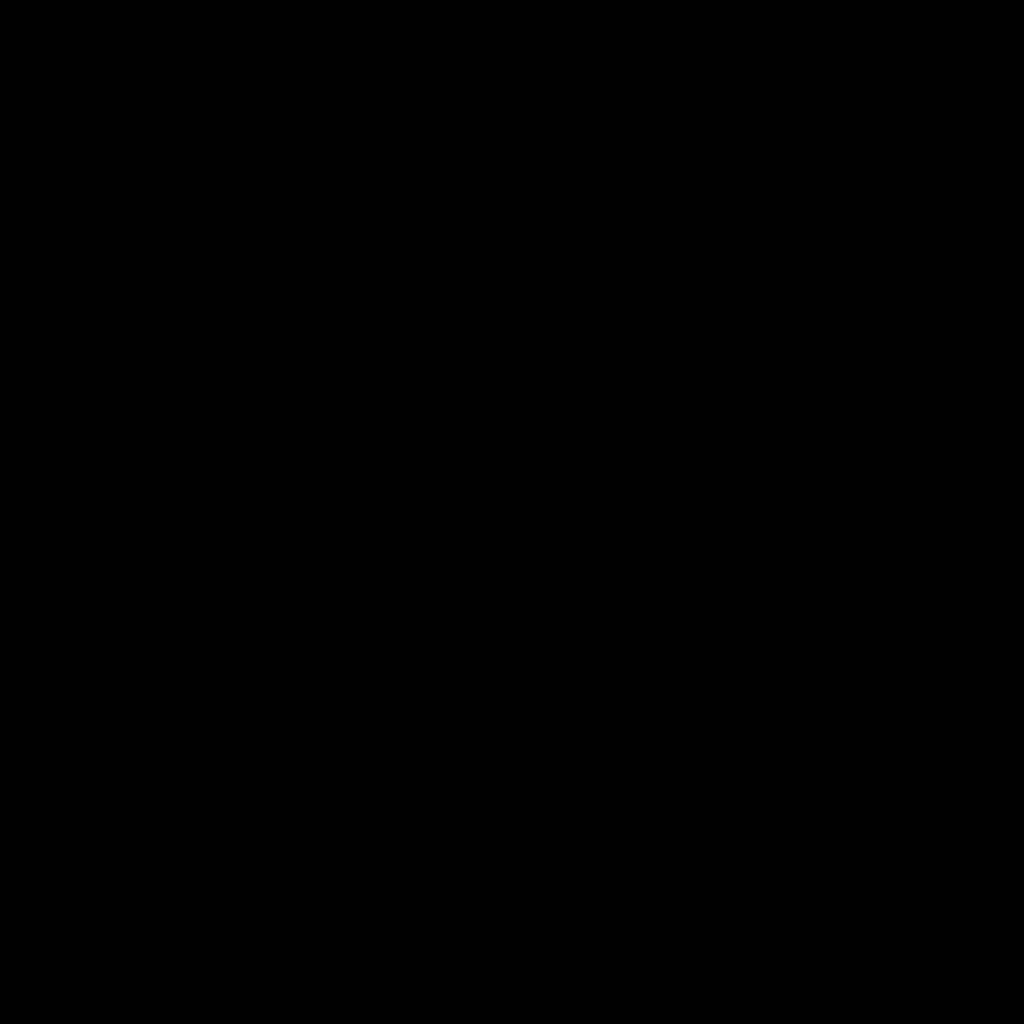

Supplement: Supplementary file 9 — Expanded View Figure and Appendix source data [file 44319_2025_513_MOESM9_ESM.zip › Expanded View Figure and Appendix source data/Expanded View Figure 4/EV 4E/NC ETO-/GH2AX.tif]

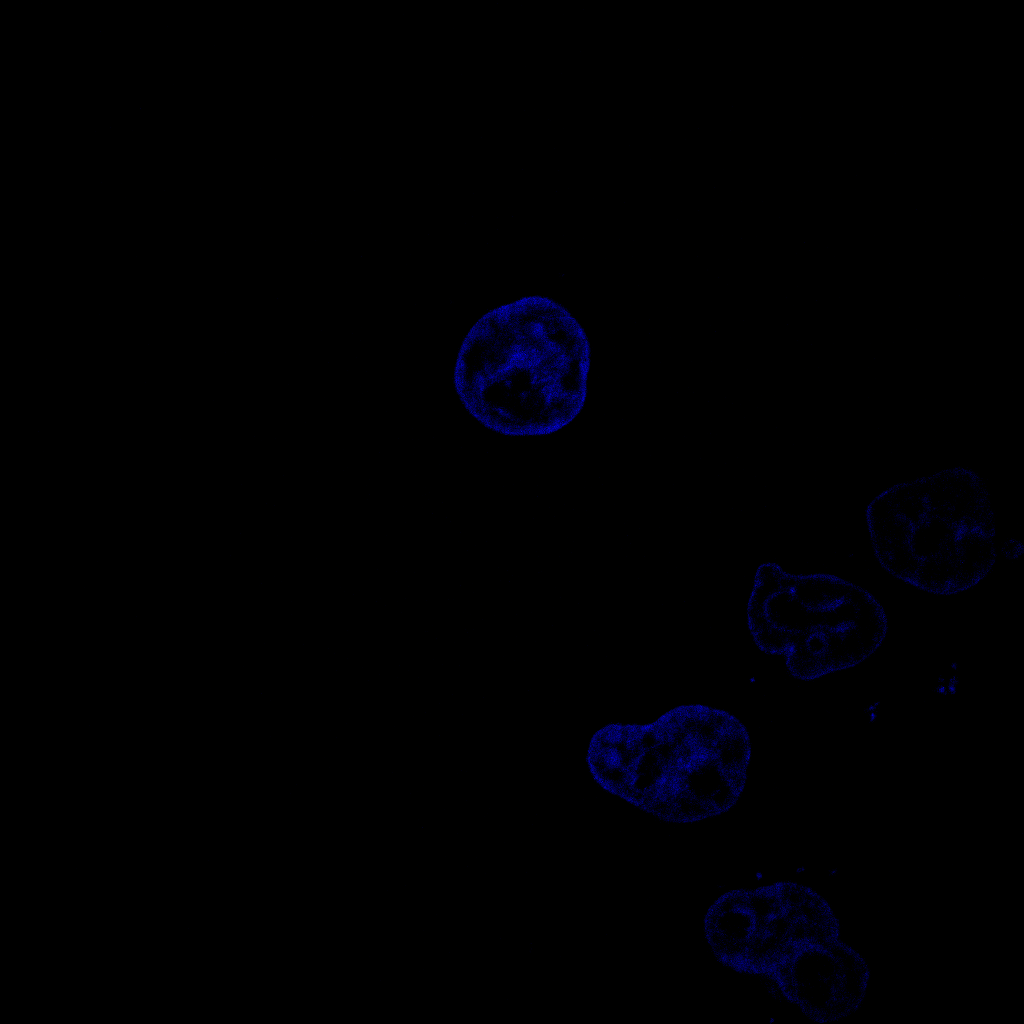

Supplement: Supplementary file 9 — Expanded View Figure and Appendix source data [file 44319_2025_513_MOESM9_ESM.zip › Expanded View Figure and Appendix source data/Expanded View Figure 4/EV 4E/WT ETO+/DAPI.tif]

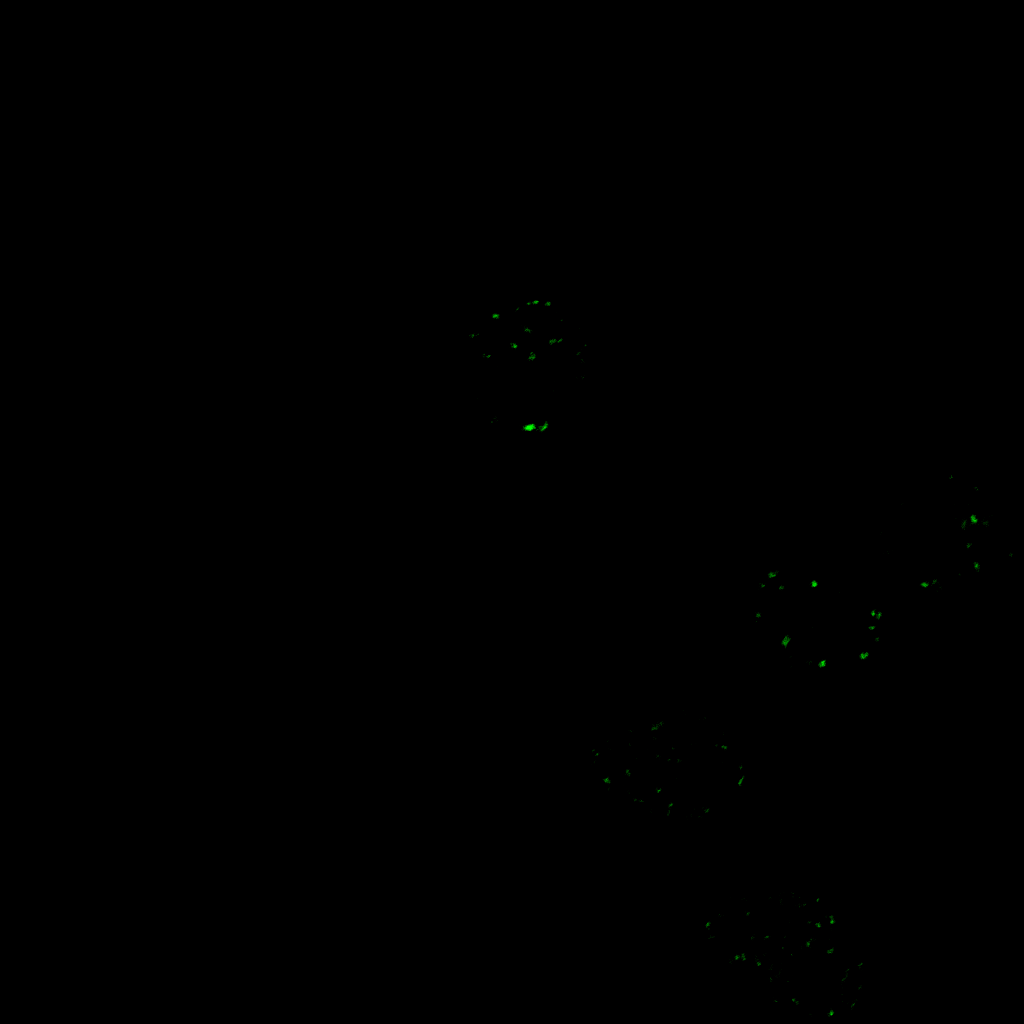

Supplement: Supplementary file 9 — Expanded View Figure and Appendix source data [file 44319_2025_513_MOESM9_ESM.zip › Expanded View Figure and Appendix source data/Expanded View Figure 4/EV 4E/WT ETO+/GH2X.tif]

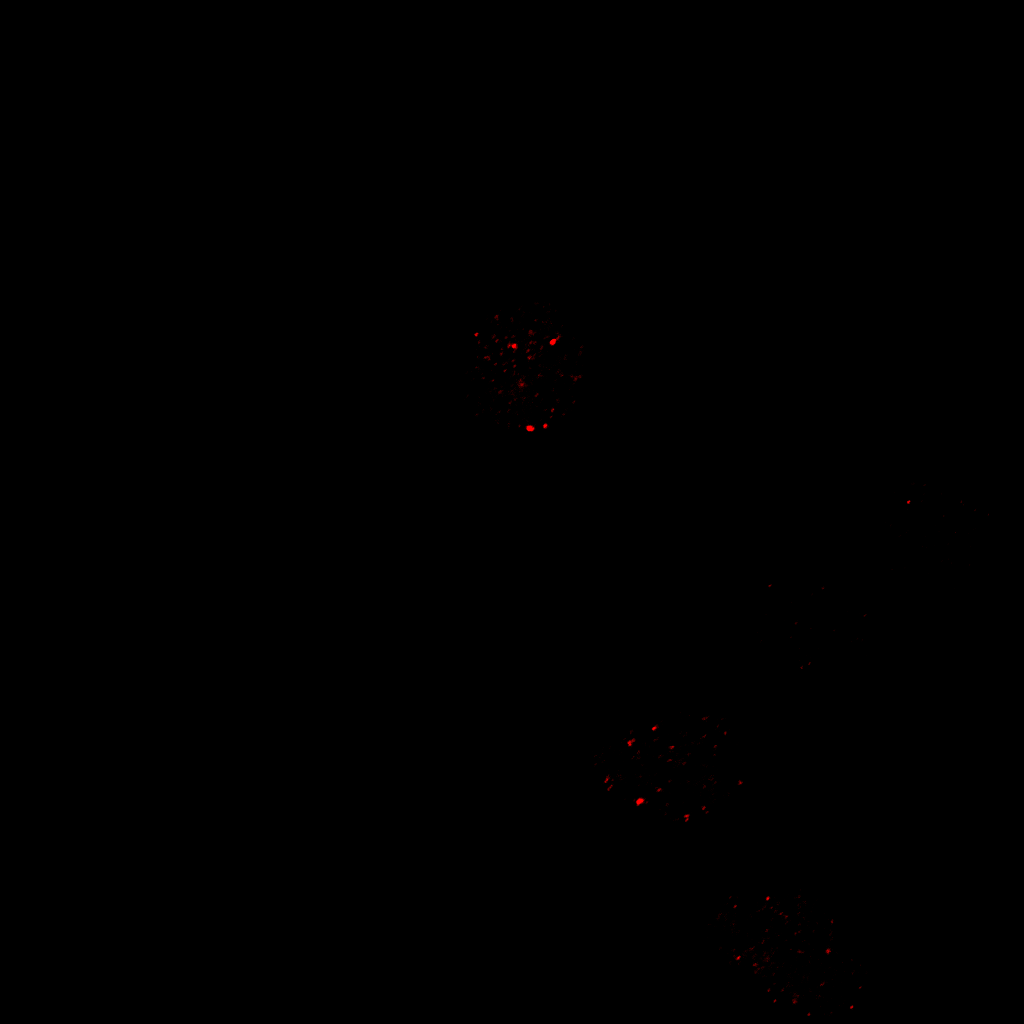

Supplement: Supplementary file 9 — Expanded View Figure and Appendix source data [file 44319_2025_513_MOESM9_ESM.zip › Expanded View Figure and Appendix source data/Expanded View Figure 4/EV 4E/WT ETO+/RAD51.tif]

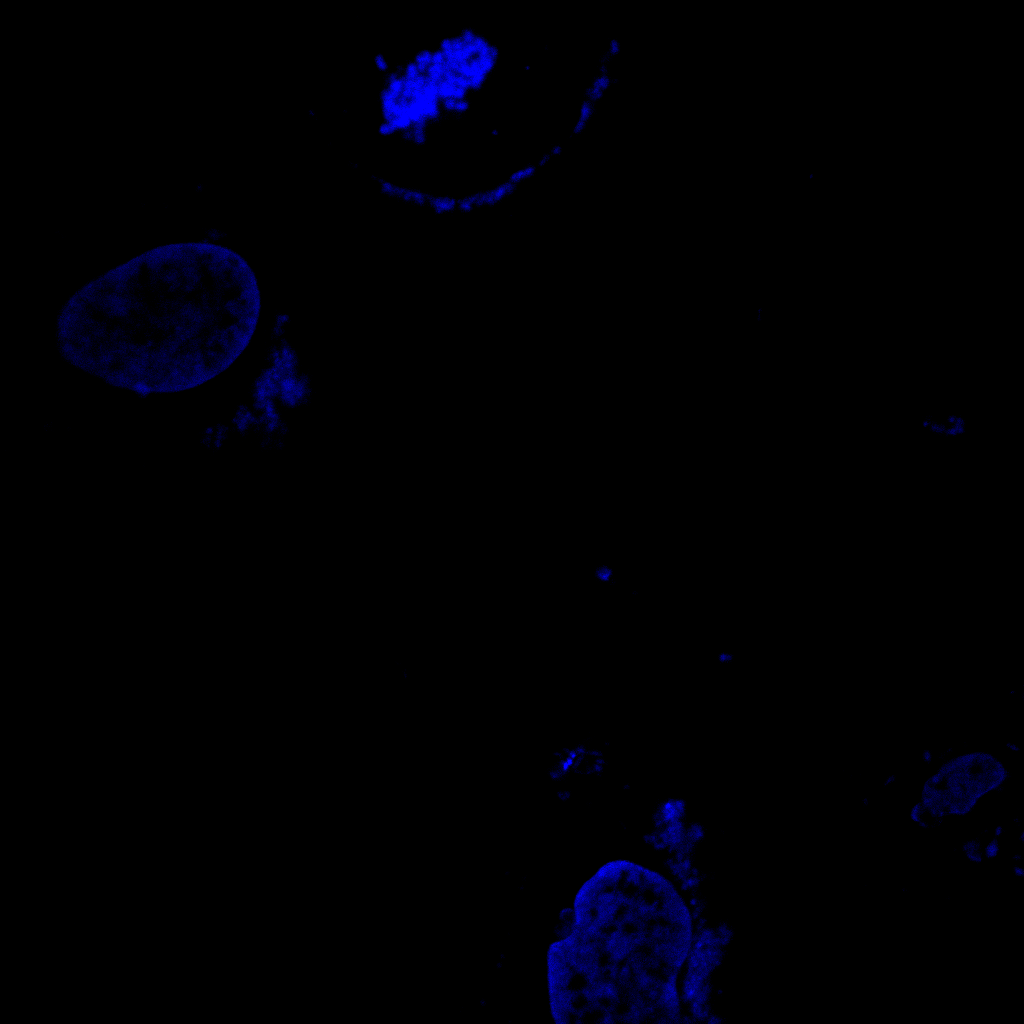

Supplement: Supplementary file 9 — Expanded View Figure and Appendix source data [file 44319_2025_513_MOESM9_ESM.zip › Expanded View Figure and Appendix source data/Expanded View Figure 4/EV 4E/WT ETO-/DAPI.tif]

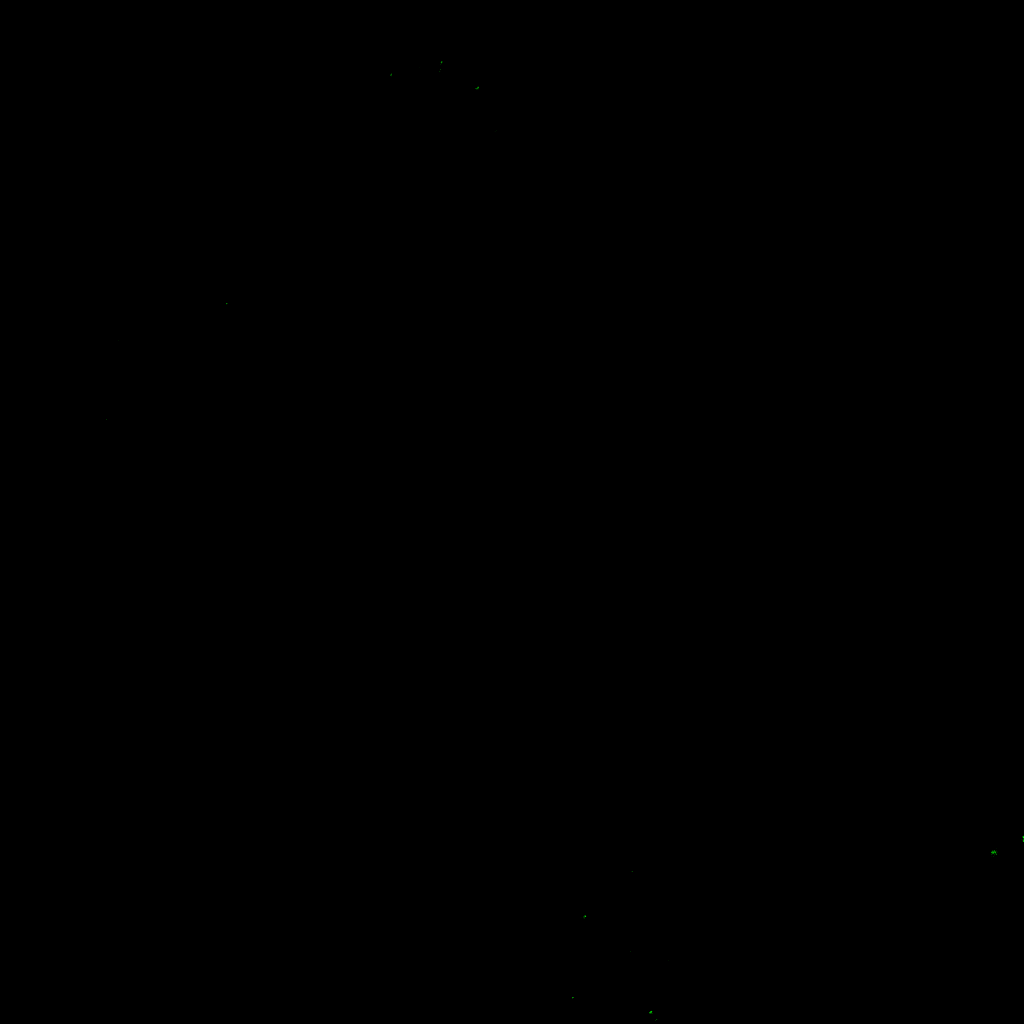

Supplement: Supplementary file 9 — Expanded View Figure and Appendix source data [file 44319_2025_513_MOESM9_ESM.zip › Expanded View Figure and Appendix source data/Expanded View Figure 4/EV 4E/WT ETO-/GH2AX.tif]

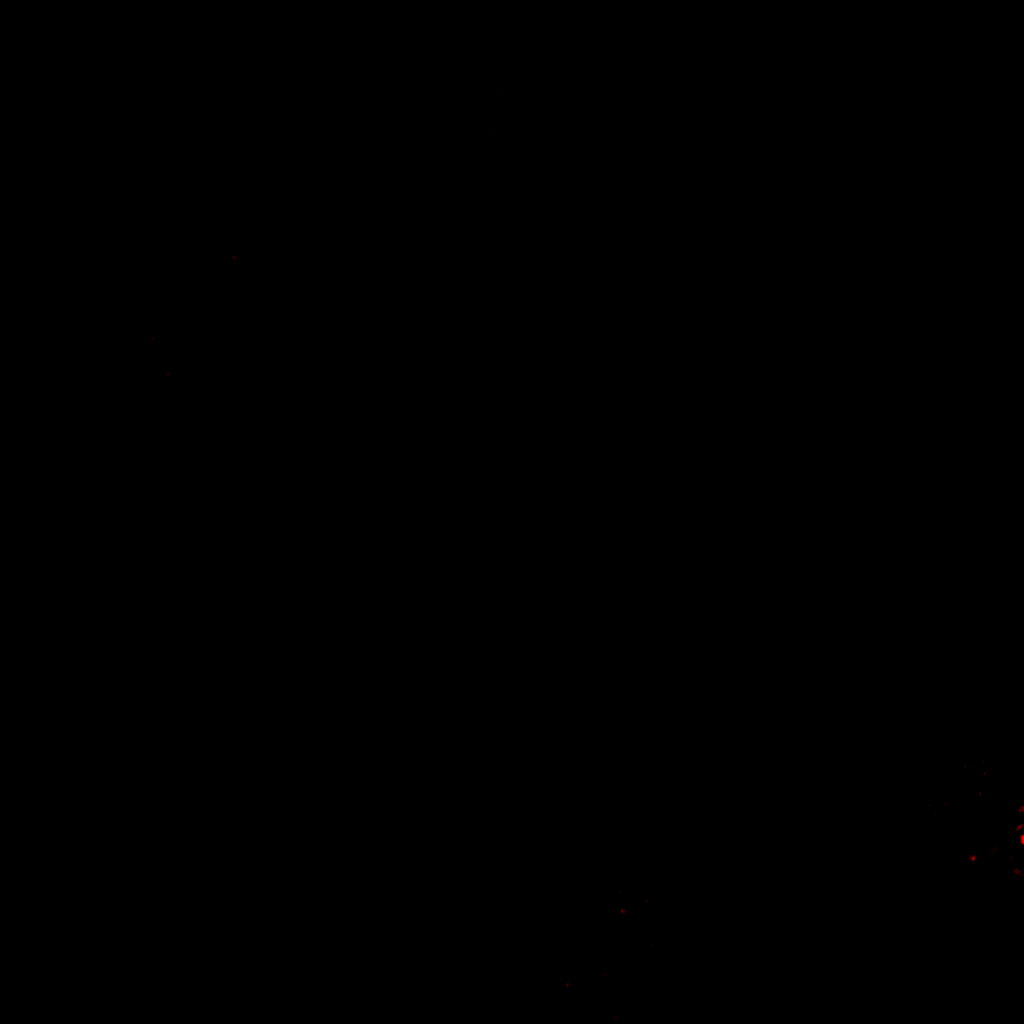

Supplement: Supplementary file 9 — Expanded View Figure and Appendix source data [file 44319_2025_513_MOESM9_ESM.zip › Expanded View Figure and Appendix source data/Expanded View Figure 4/EV 4E/WT ETO-/RAD51.tif]

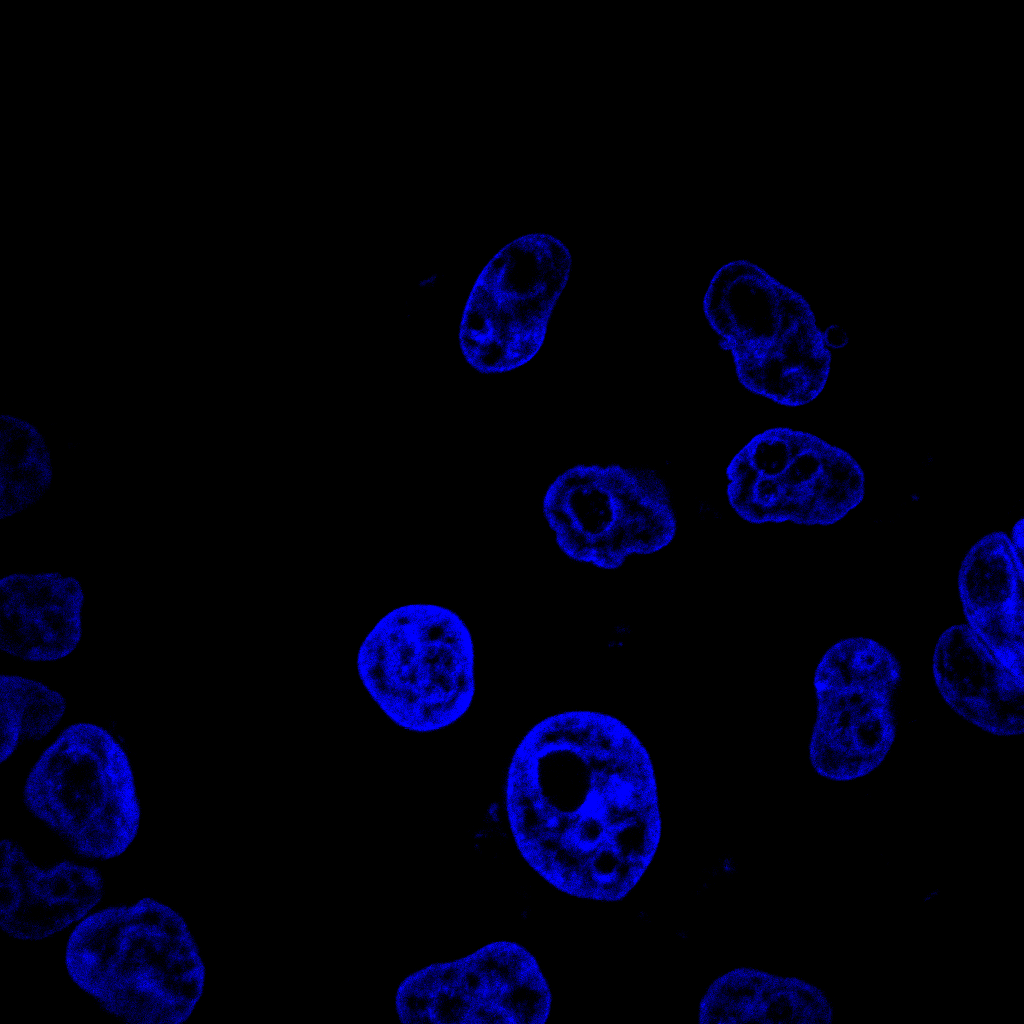

Supplement: Supplementary file 9 — Expanded View Figure and Appendix source data [file 44319_2025_513_MOESM9_ESM.zip › Expanded View Figure and Appendix source data/Expanded View Figure 5/EV 5B/NC 0 h/DAPI.tif]

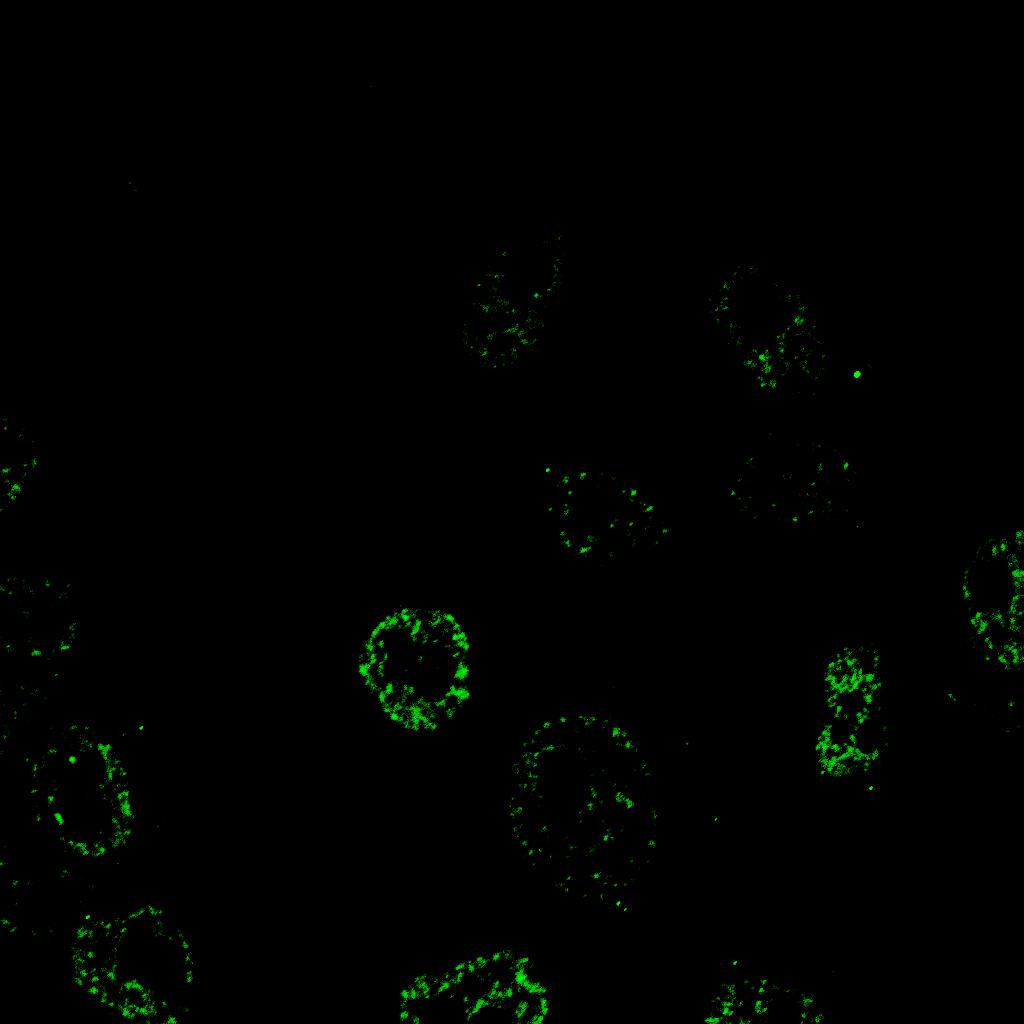

Supplement: Supplementary file 9 — Expanded View Figure and Appendix source data [file 44319_2025_513_MOESM9_ESM.zip › Expanded View Figure and Appendix source data/Expanded View Figure 5/EV 5B/NC 0 h/GH2AX.tif]

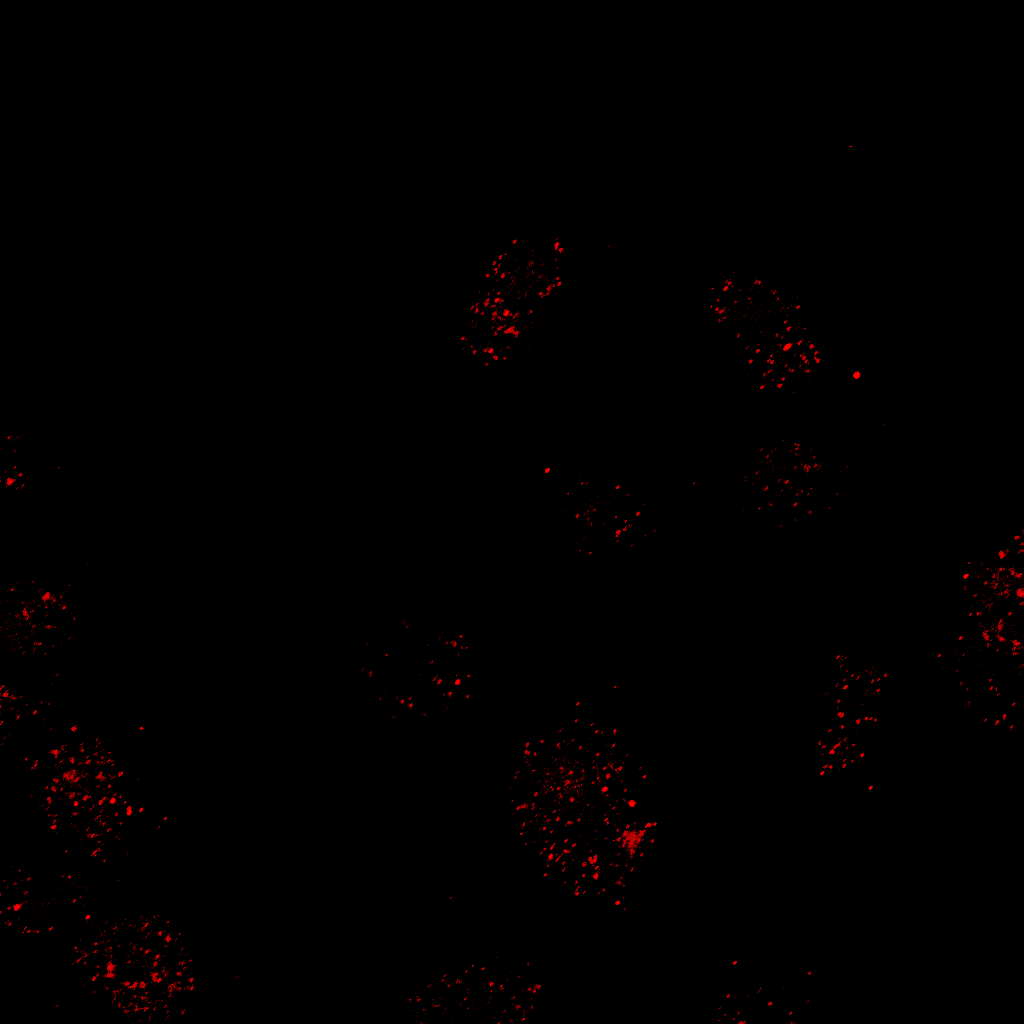

Supplement: Supplementary file 9 — Expanded View Figure and Appendix source data [file 44319_2025_513_MOESM9_ESM.zip › Expanded View Figure and Appendix source data/Expanded View Figure 5/EV 5B/NC 0 h/RAD51.tif]

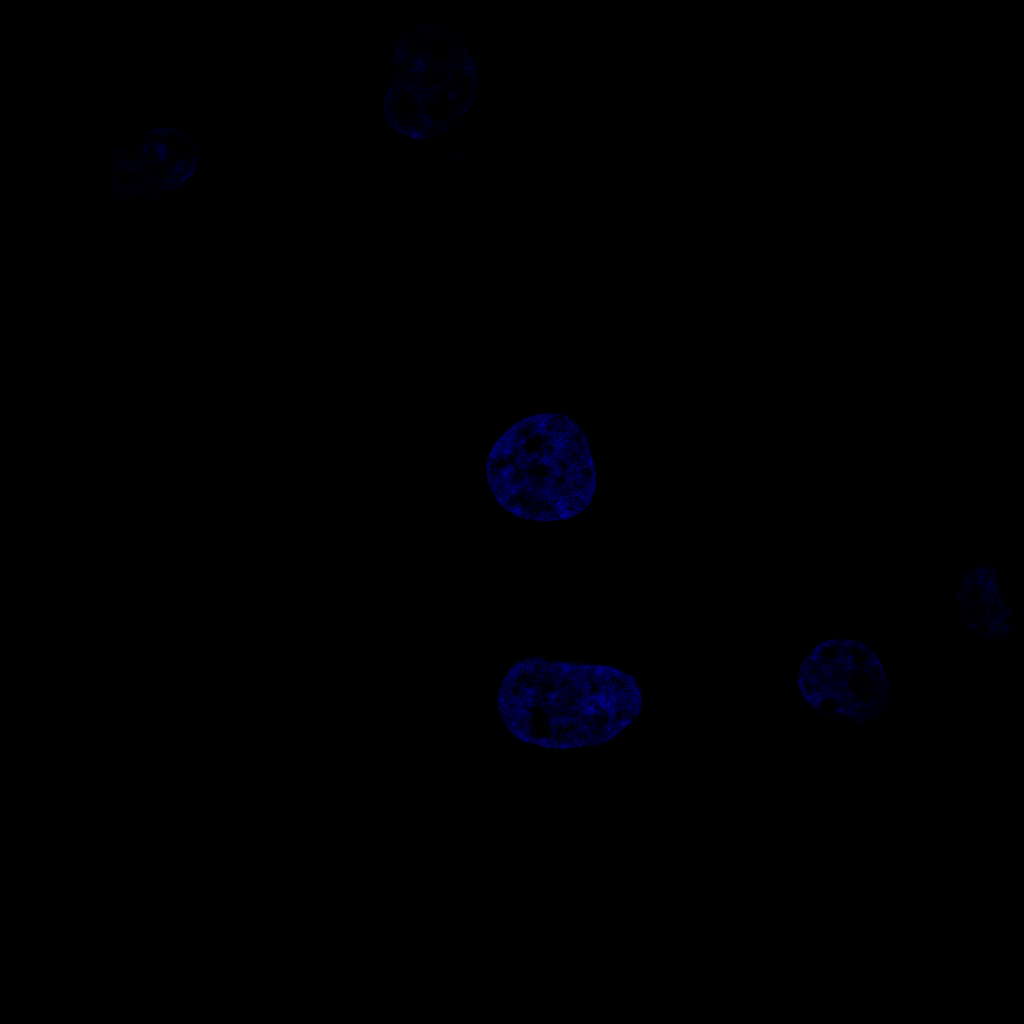

Supplement: Supplementary file 9 — Expanded View Figure and Appendix source data [file 44319_2025_513_MOESM9_ESM.zip › Expanded View Figure and Appendix source data/Expanded View Figure 5/EV 5B/NC 1 h/DAPI.tif]

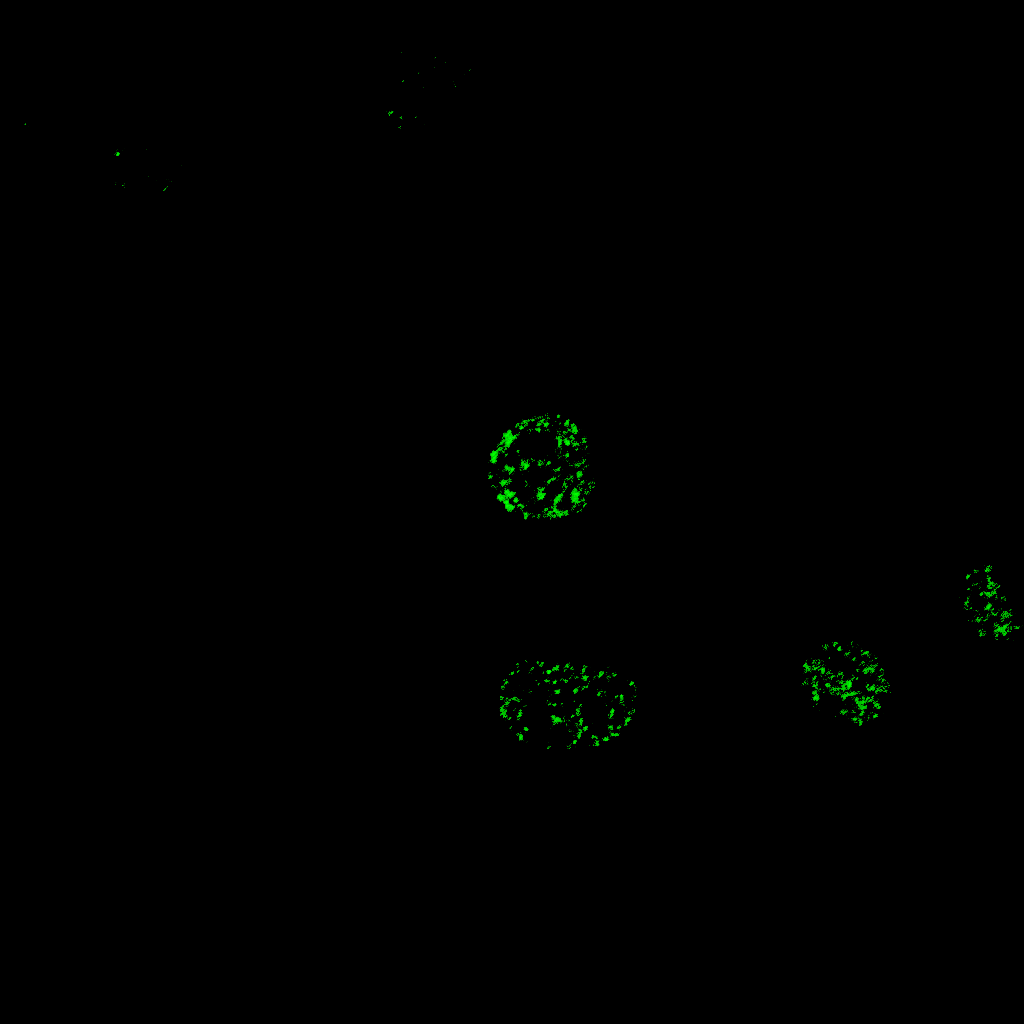

Supplement: Supplementary file 9 — Expanded View Figure and Appendix source data [file 44319_2025_513_MOESM9_ESM.zip › Expanded View Figure and Appendix source data/Expanded View Figure 5/EV 5B/NC 1 h/GH2AX.tif]

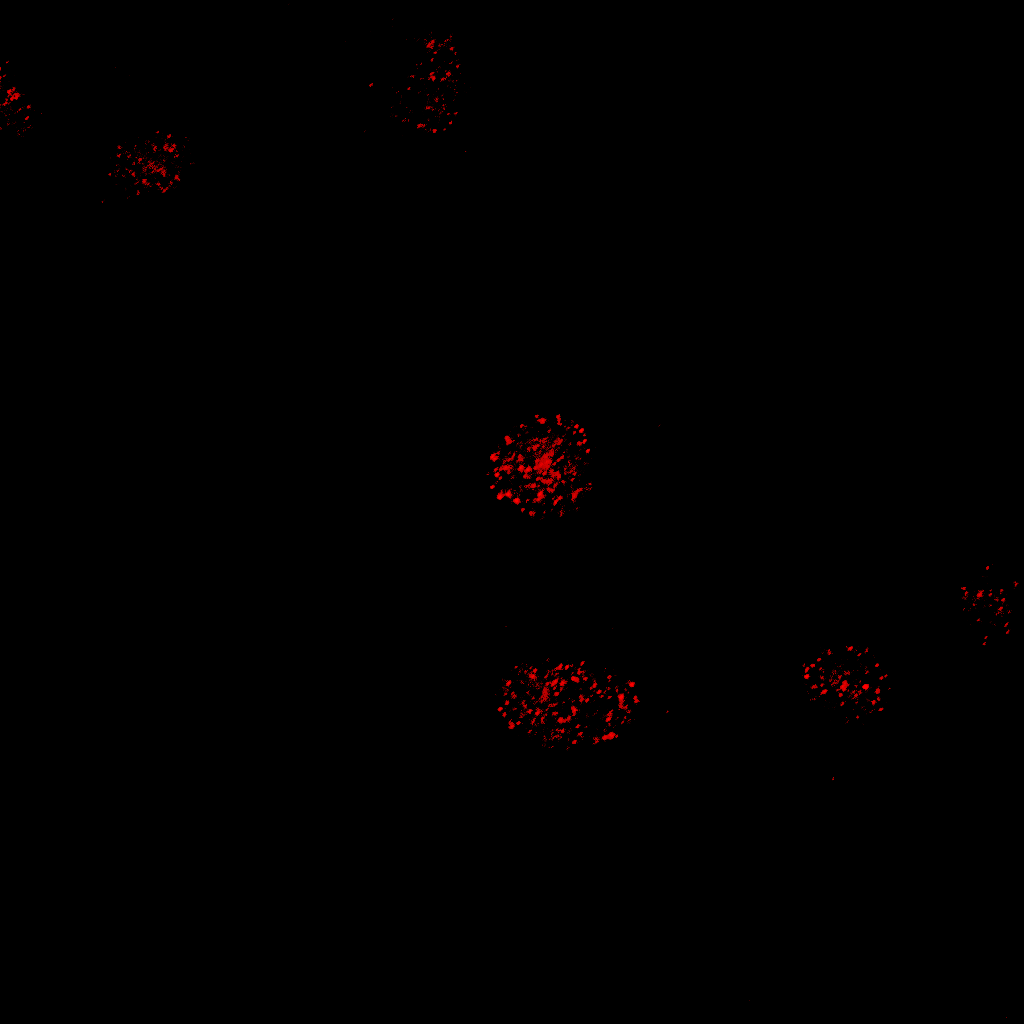

Supplement: Supplementary file 9 — Expanded View Figure and Appendix source data [file 44319_2025_513_MOESM9_ESM.zip › Expanded View Figure and Appendix source data/Expanded View Figure 5/EV 5B/NC 1 h/RAD51.tif]

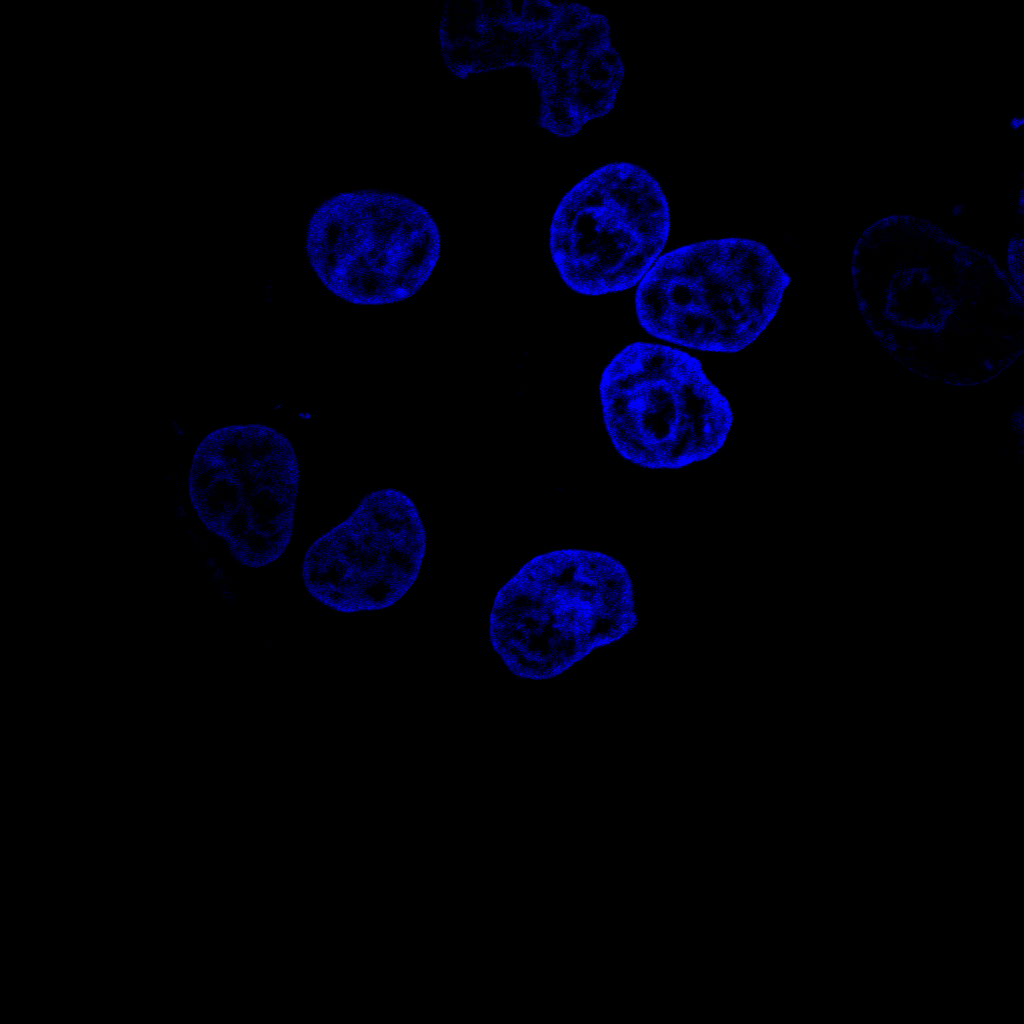

Supplement: Supplementary file 9 — Expanded View Figure and Appendix source data [file 44319_2025_513_MOESM9_ESM.zip › Expanded View Figure and Appendix source data/Expanded View Figure 5/EV 5B/NC 2 h/DAPI.tif]

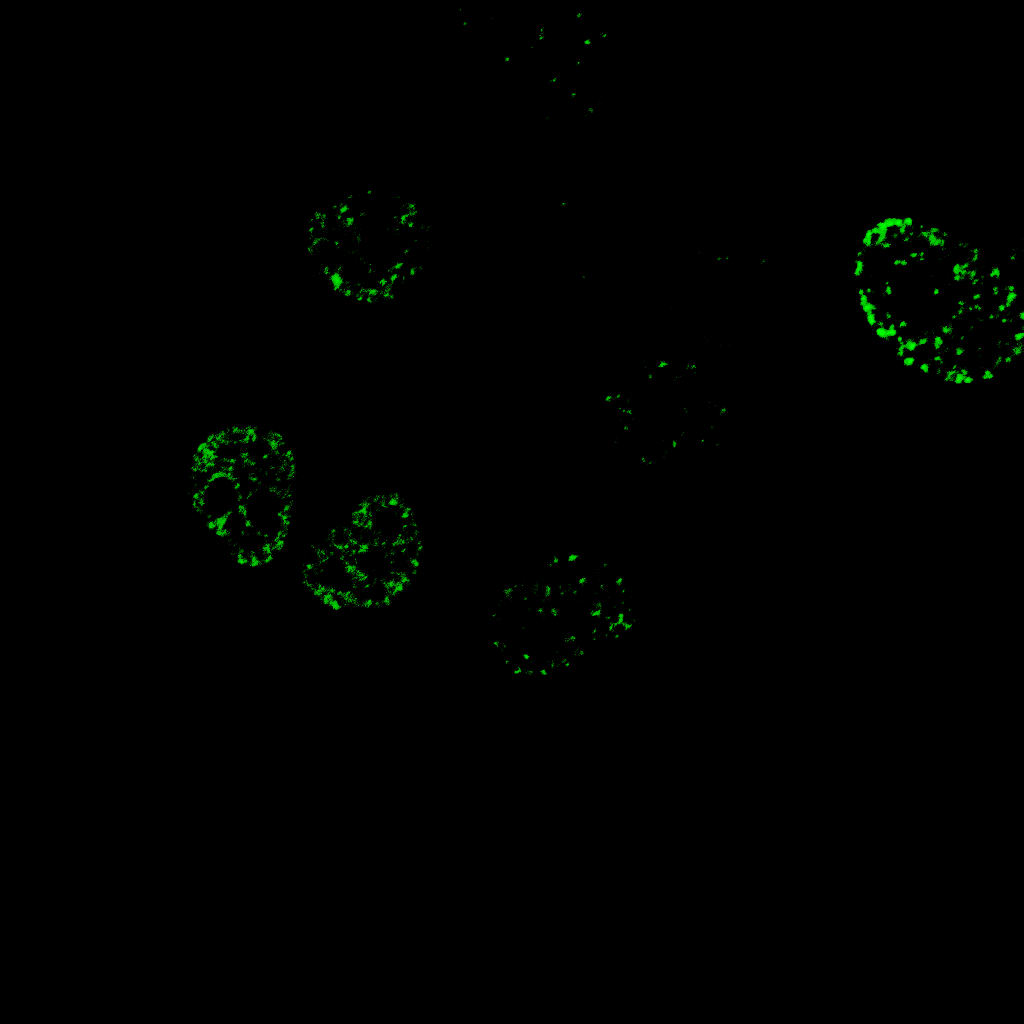

Supplement: Supplementary file 9 — Expanded View Figure and Appendix source data [file 44319_2025_513_MOESM9_ESM.zip › Expanded View Figure and Appendix source data/Expanded View Figure 5/EV 5B/NC 2 h/GH2AX.tif]

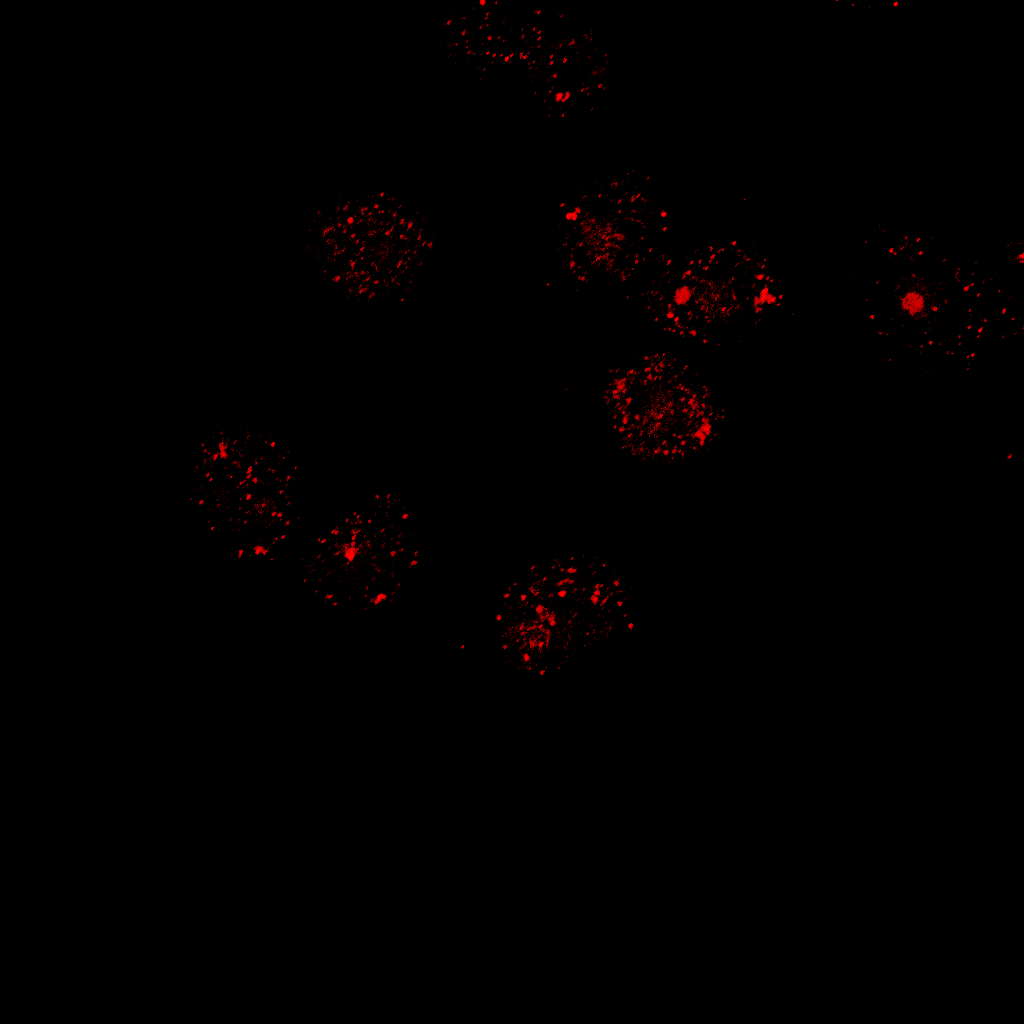

Supplement: Supplementary file 9 — Expanded View Figure and Appendix source data [file 44319_2025_513_MOESM9_ESM.zip › Expanded View Figure and Appendix source data/Expanded View Figure 5/EV 5B/NC 2 h/RAD51.tif]

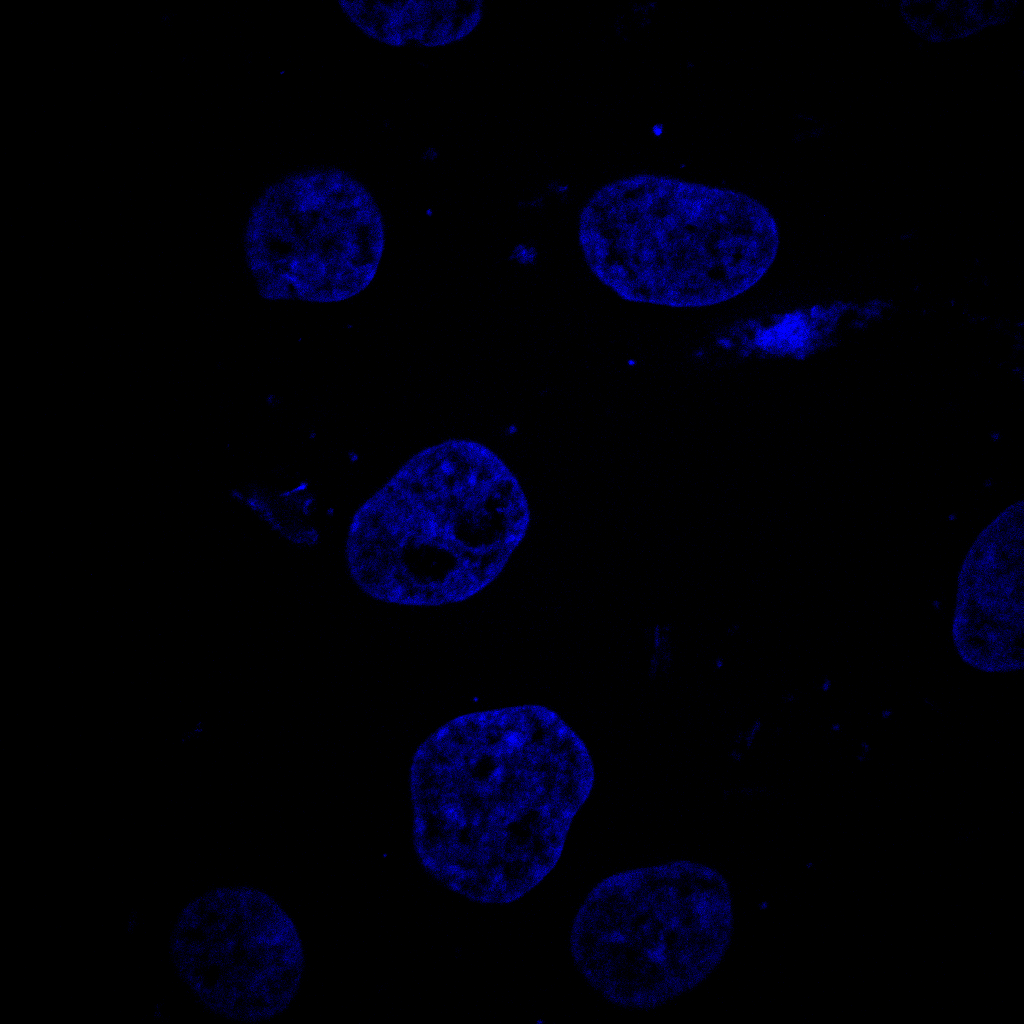

Supplement: Supplementary file 9 — Expanded View Figure and Appendix source data [file 44319_2025_513_MOESM9_ESM.zip › Expanded View Figure and Appendix source data/Expanded View Figure 5/EV 5B/NC 24 h/DAPI.tif]

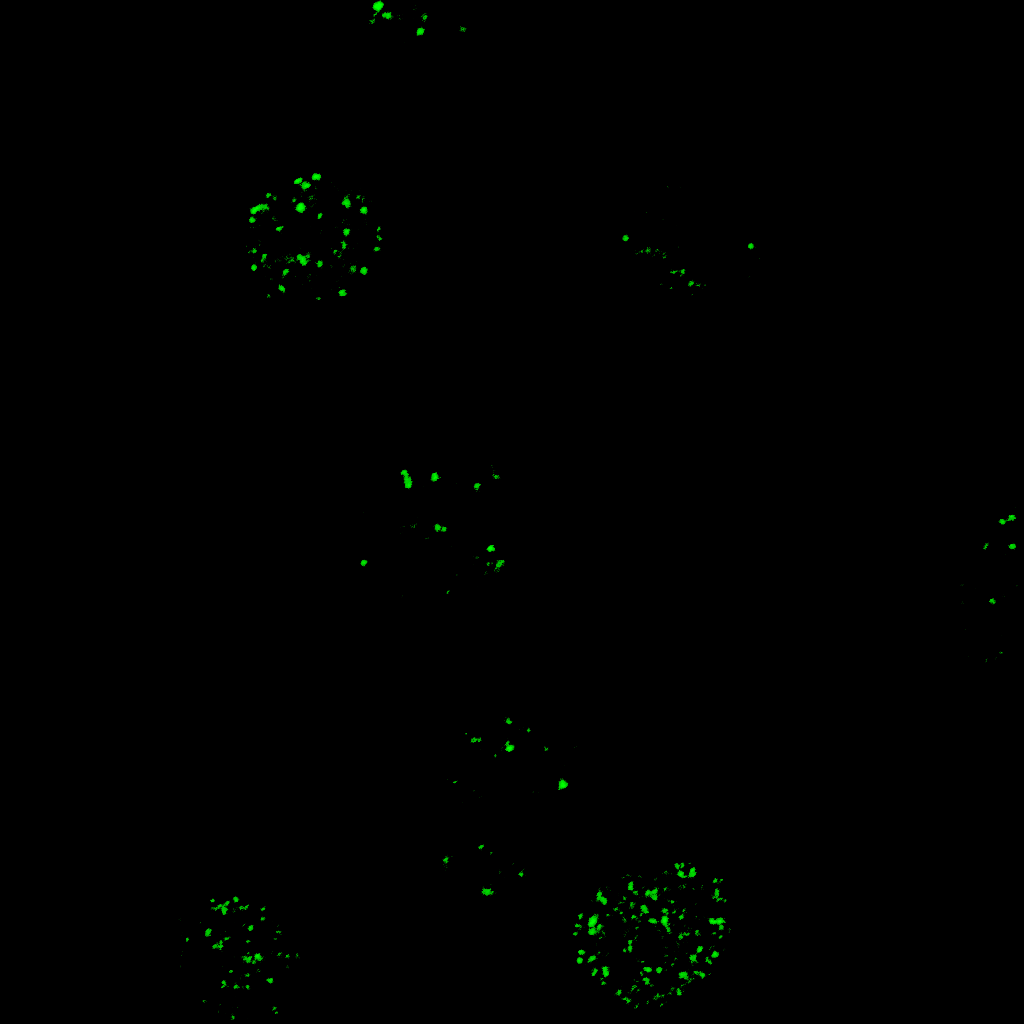

Supplement: Supplementary file 9 — Expanded View Figure and Appendix source data [file 44319_2025_513_MOESM9_ESM.zip › Expanded View Figure and Appendix source data/Expanded View Figure 5/EV 5B/NC 24 h/GH2AX.tif]

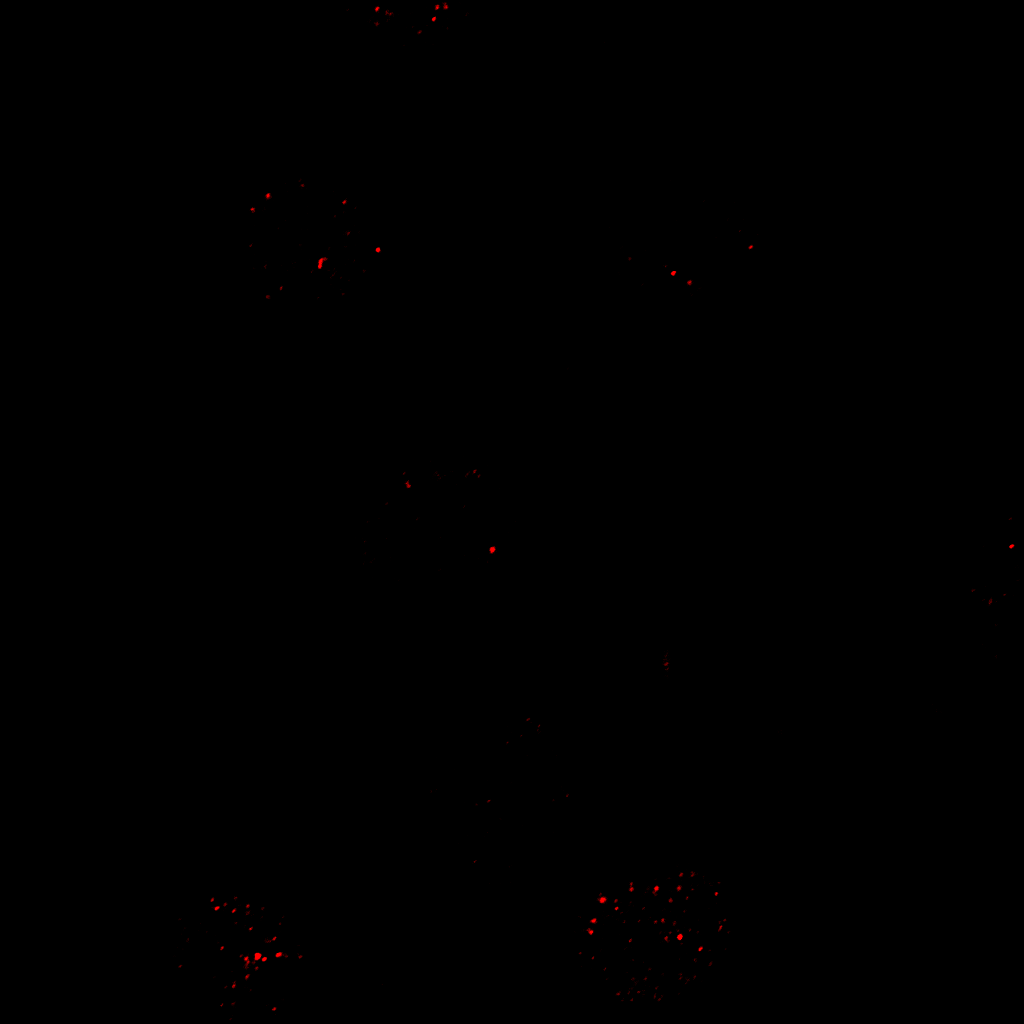

Supplement: Supplementary file 9 — Expanded View Figure and Appendix source data [file 44319_2025_513_MOESM9_ESM.zip › Expanded View Figure and Appendix source data/Expanded View Figure 5/EV 5B/NC 24 h/RAD51.tif]

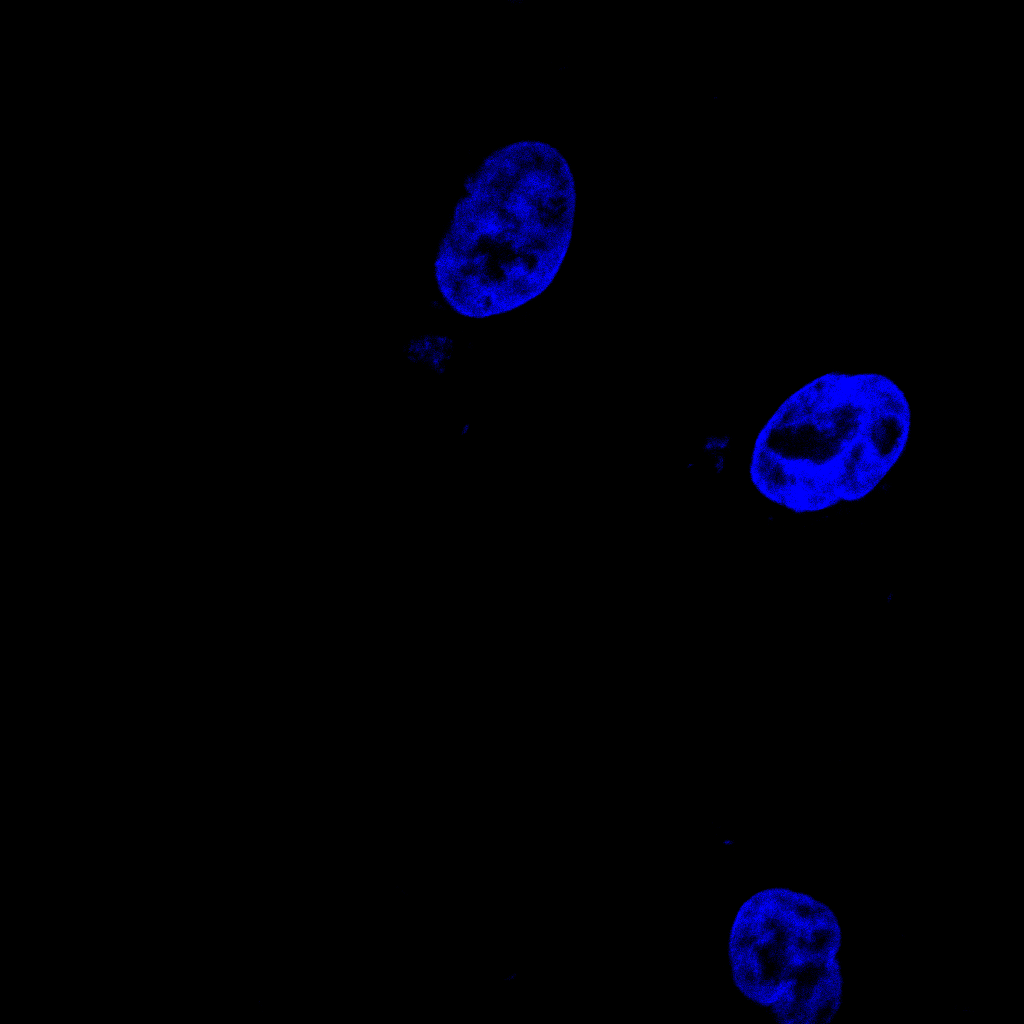

Supplement: Supplementary file 9 — Expanded View Figure and Appendix source data [file 44319_2025_513_MOESM9_ESM.zip › Expanded View Figure and Appendix source data/Expanded View Figure 5/EV 5B/NC 4 h/DAPI.tif]

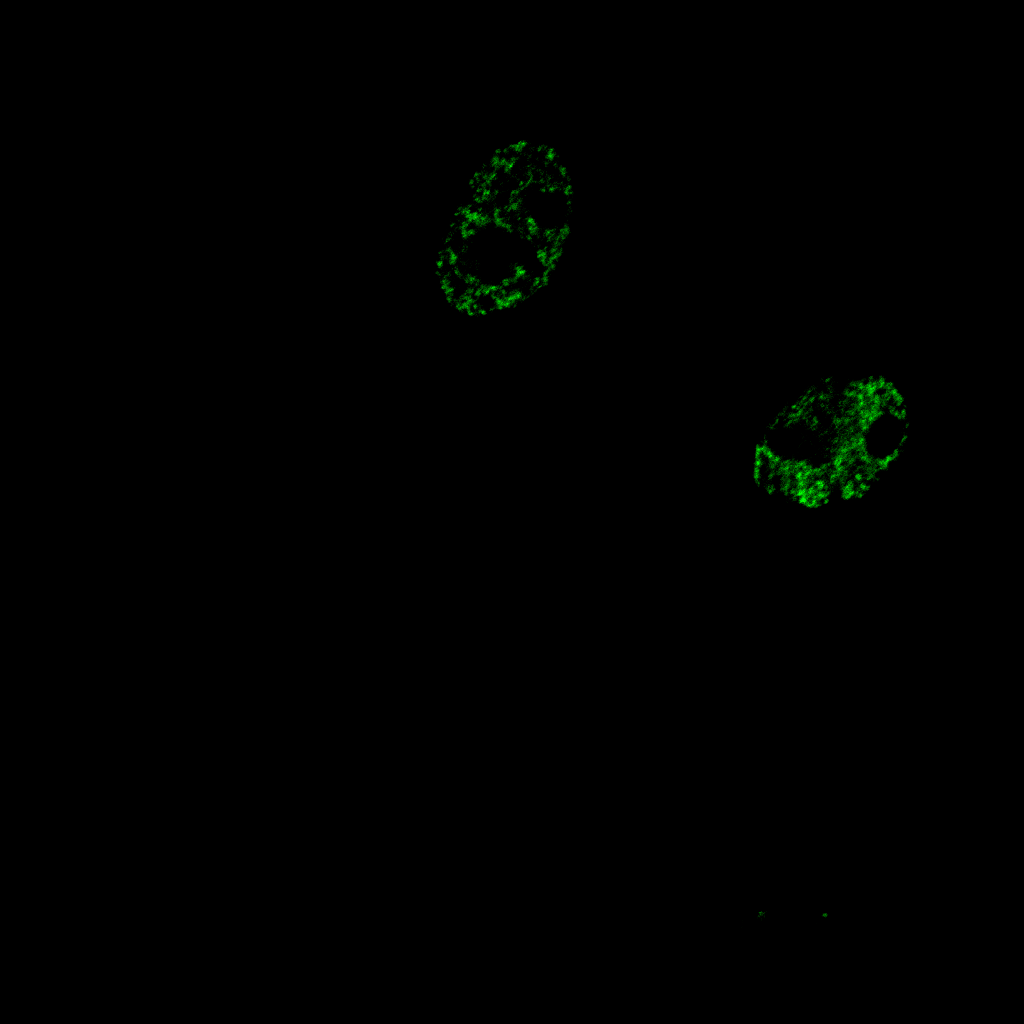

Supplement: Supplementary file 9 — Expanded View Figure and Appendix source data [file 44319_2025_513_MOESM9_ESM.zip › Expanded View Figure and Appendix source data/Expanded View Figure 5/EV 5B/NC 4 h/GH2AX.tif]

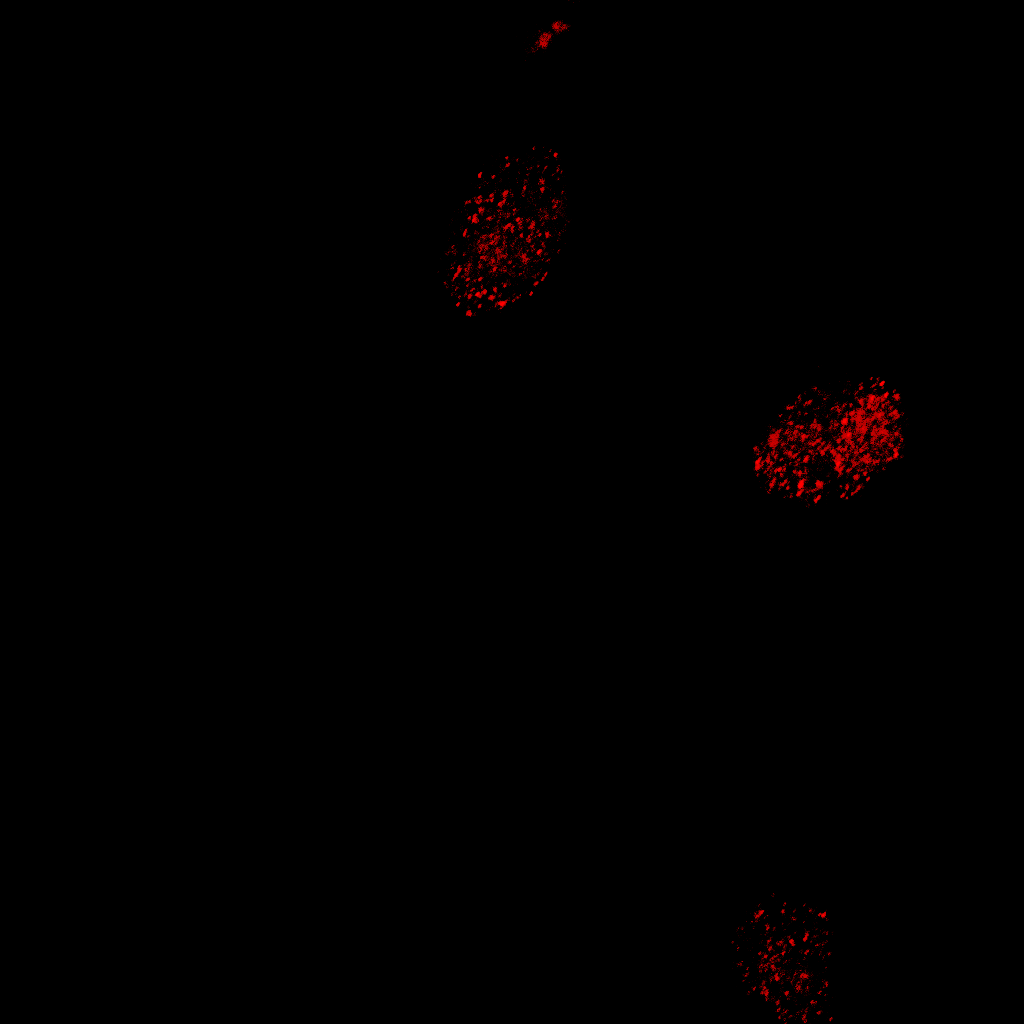

Supplement: Supplementary file 9 — Expanded View Figure and Appendix source data [file 44319_2025_513_MOESM9_ESM.zip › Expanded View Figure and Appendix source data/Expanded View Figure 5/EV 5B/NC 4 h/RAD51.tif]

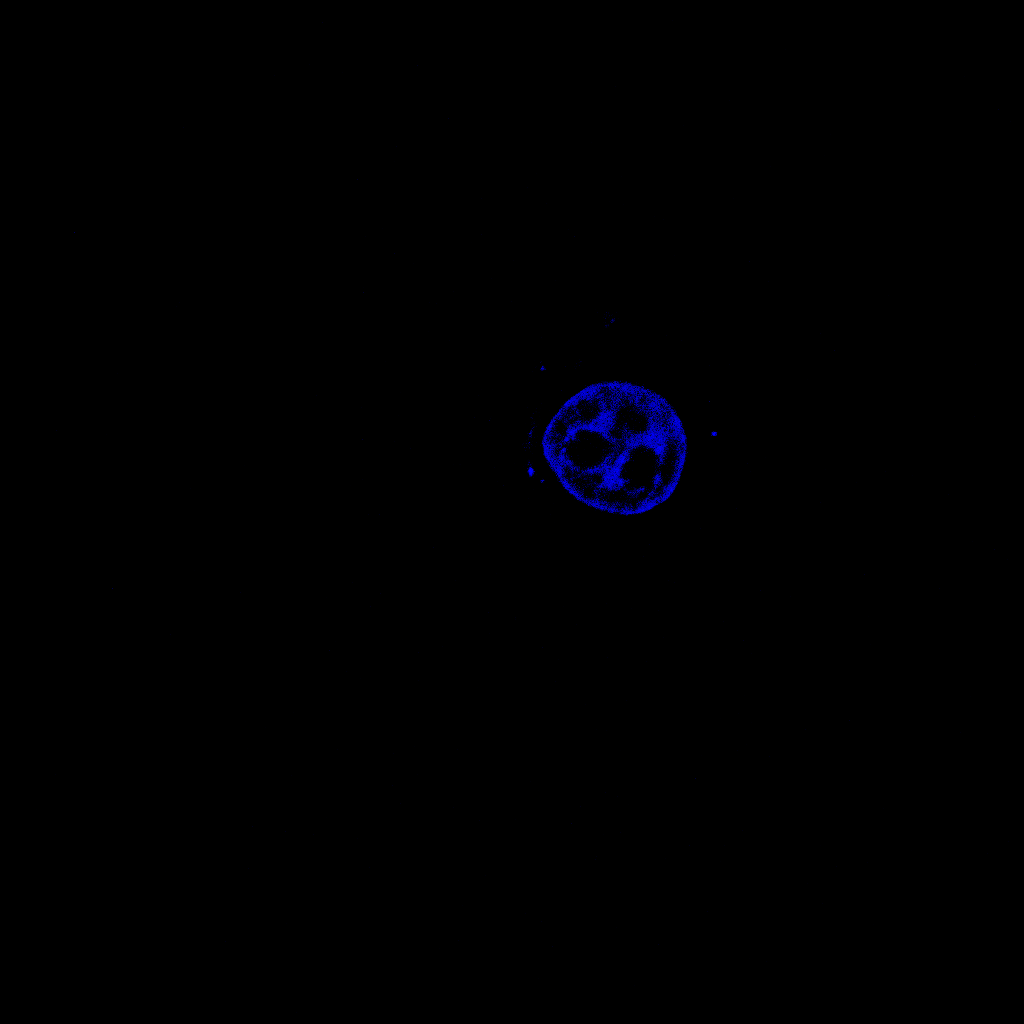

Supplement: Supplementary file 9 — Expanded View Figure and Appendix source data [file 44319_2025_513_MOESM9_ESM.zip › Expanded View Figure and Appendix source data/Expanded View Figure 5/EV 5B/NC 8 h/DAPI.tif]

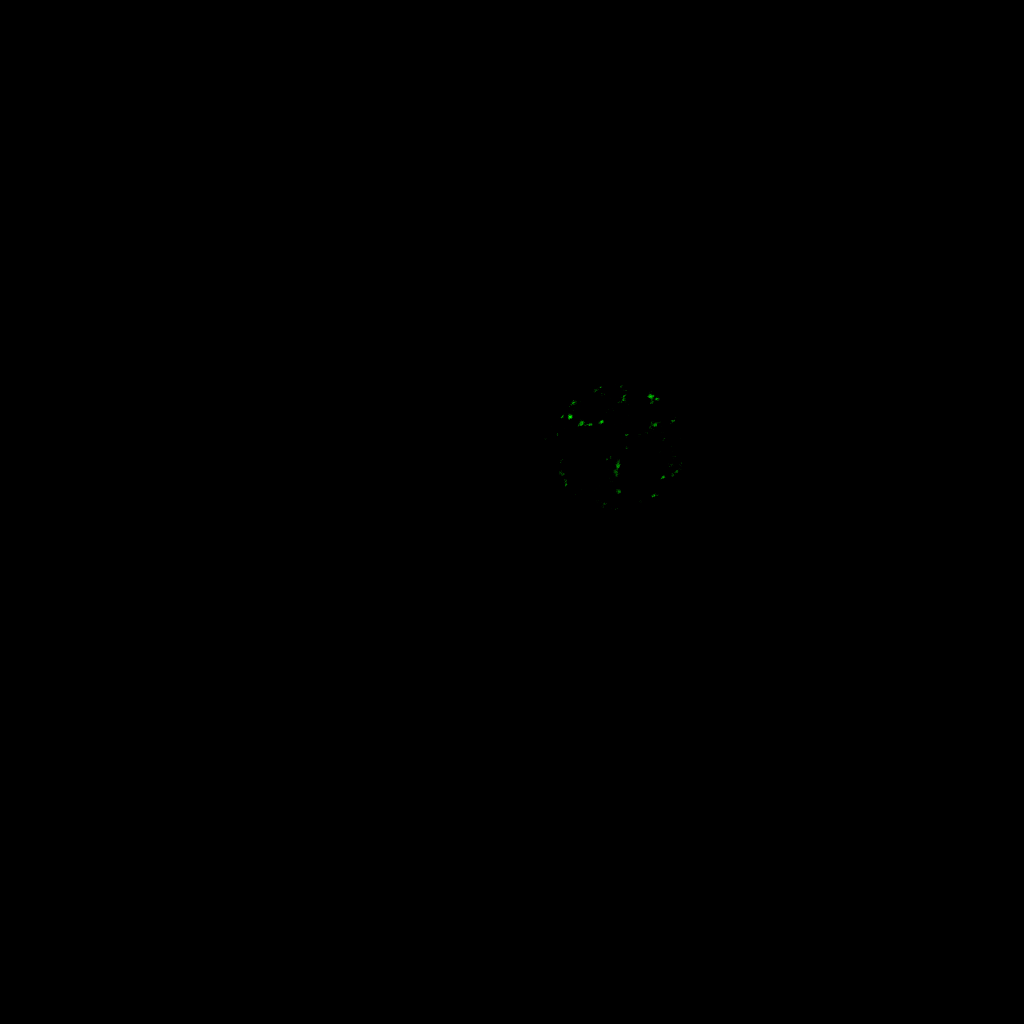

Supplement: Supplementary file 9 — Expanded View Figure and Appendix source data [file 44319_2025_513_MOESM9_ESM.zip › Expanded View Figure and Appendix source data/Expanded View Figure 5/EV 5B/NC 8 h/GH2AX.tif]

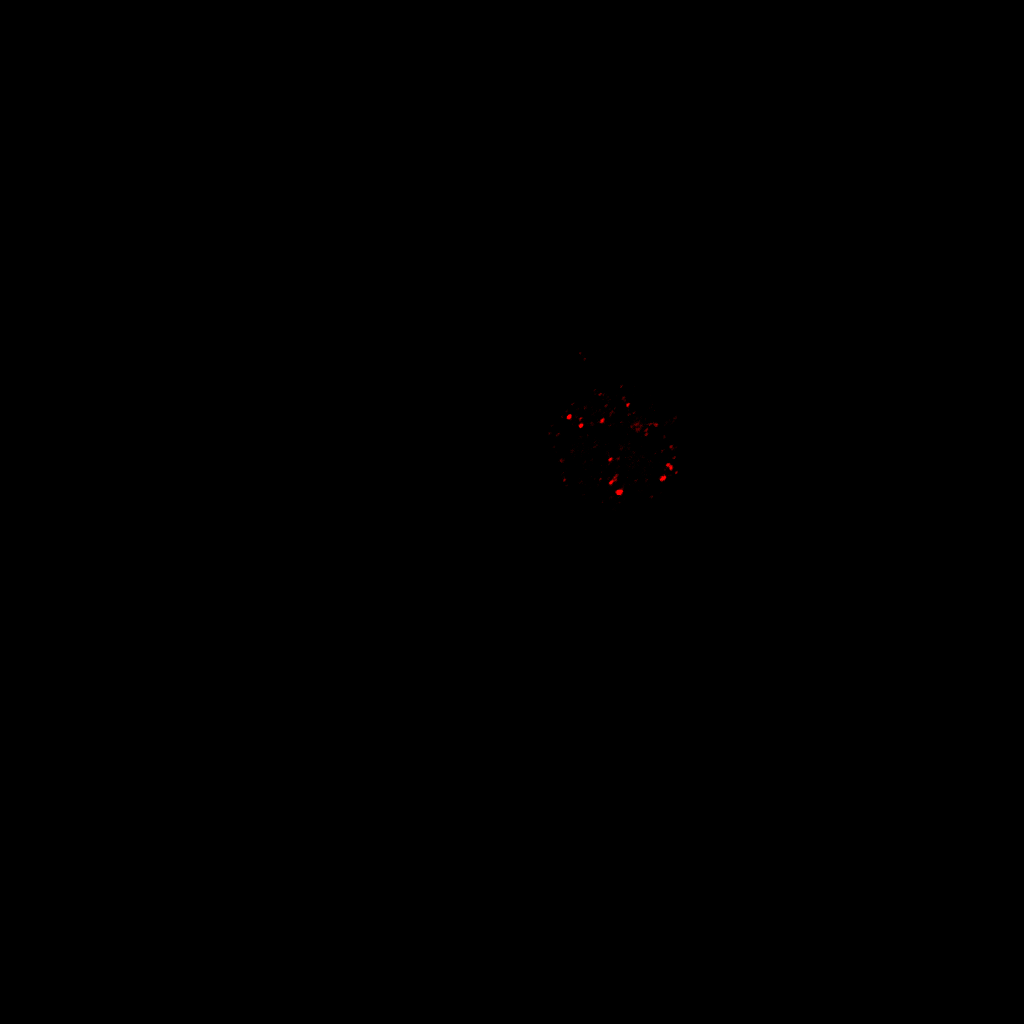

Supplement: Supplementary file 9 — Expanded View Figure and Appendix source data [file 44319_2025_513_MOESM9_ESM.zip › Expanded View Figure and Appendix source data/Expanded View Figure 5/EV 5B/NC 8 h/RAD51.tif]

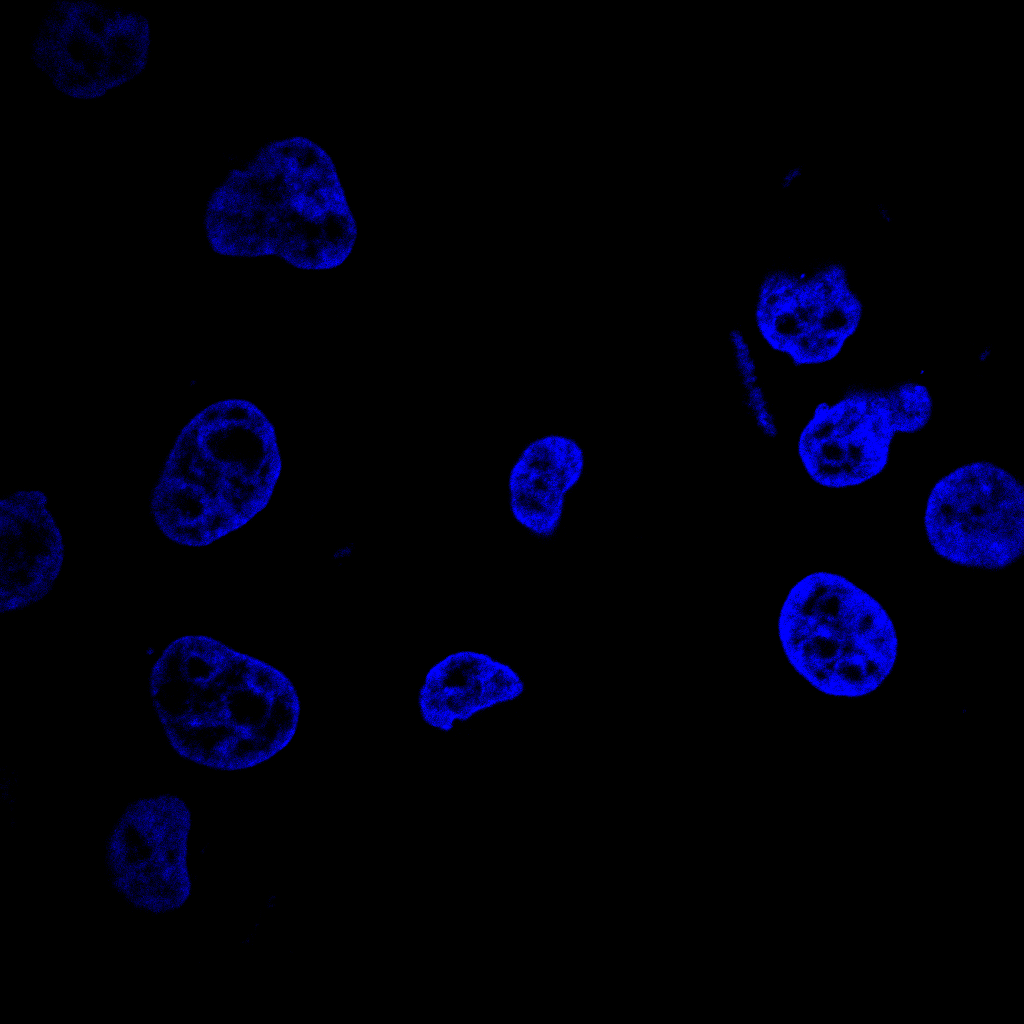

Supplement: Supplementary file 9 — Expanded View Figure and Appendix source data [file 44319_2025_513_MOESM9_ESM.zip › Expanded View Figure and Appendix source data/Expanded View Figure 5/EV 5B/NC ETO-/DAPI.tif]

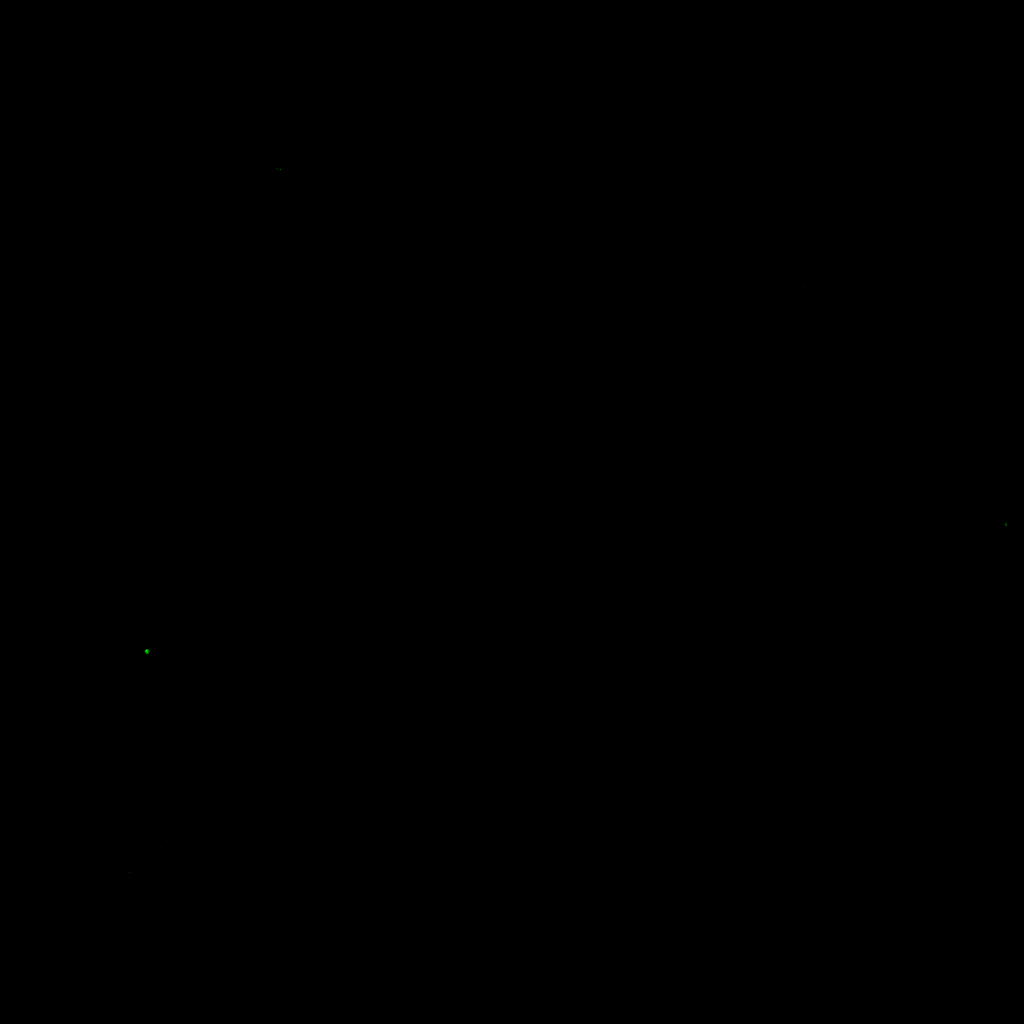

Supplement: Supplementary file 9 — Expanded View Figure and Appendix source data [file 44319_2025_513_MOESM9_ESM.zip › Expanded View Figure and Appendix source data/Expanded View Figure 5/EV 5B/NC ETO-/GH2AX.tif]

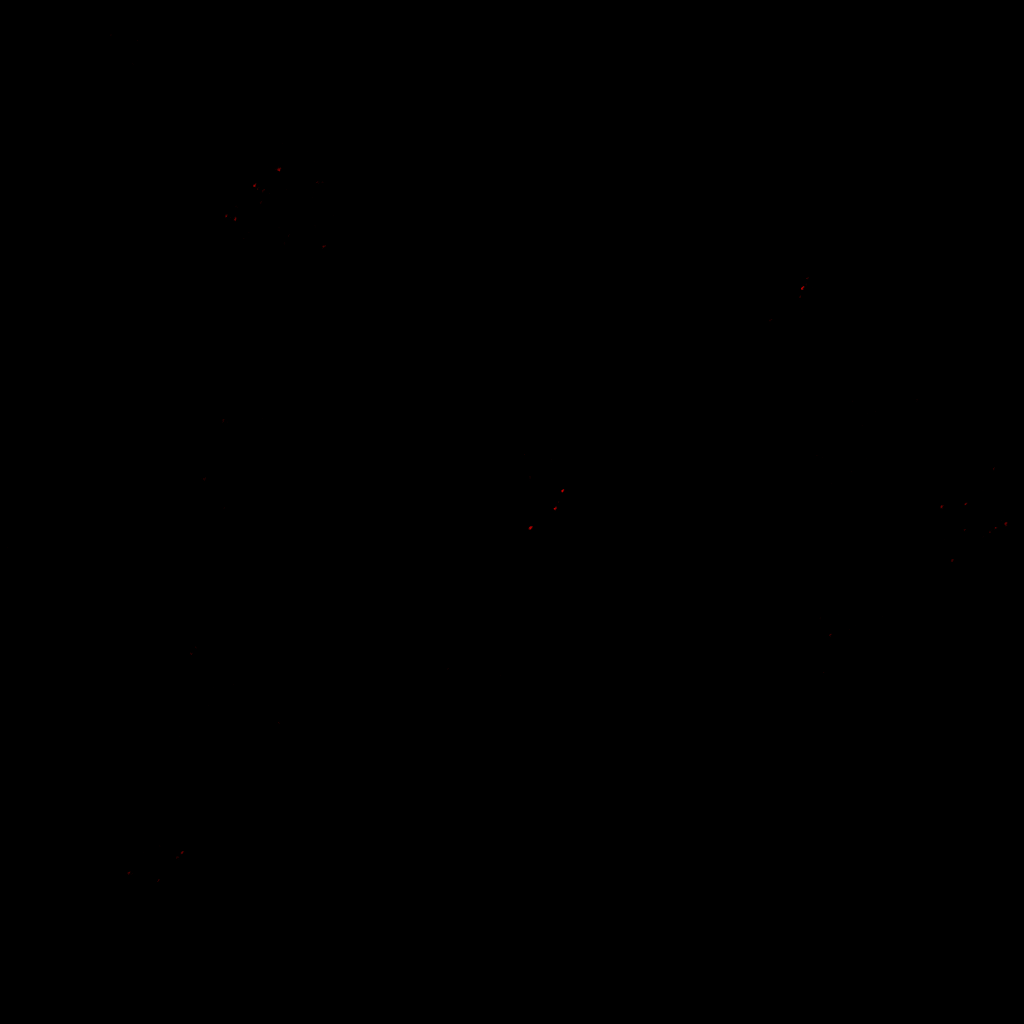

Supplement: Supplementary file 9 — Expanded View Figure and Appendix source data [file 44319_2025_513_MOESM9_ESM.zip › Expanded View Figure and Appendix source data/Expanded View Figure 5/EV 5B/NC ETO-/RAD51.tif]

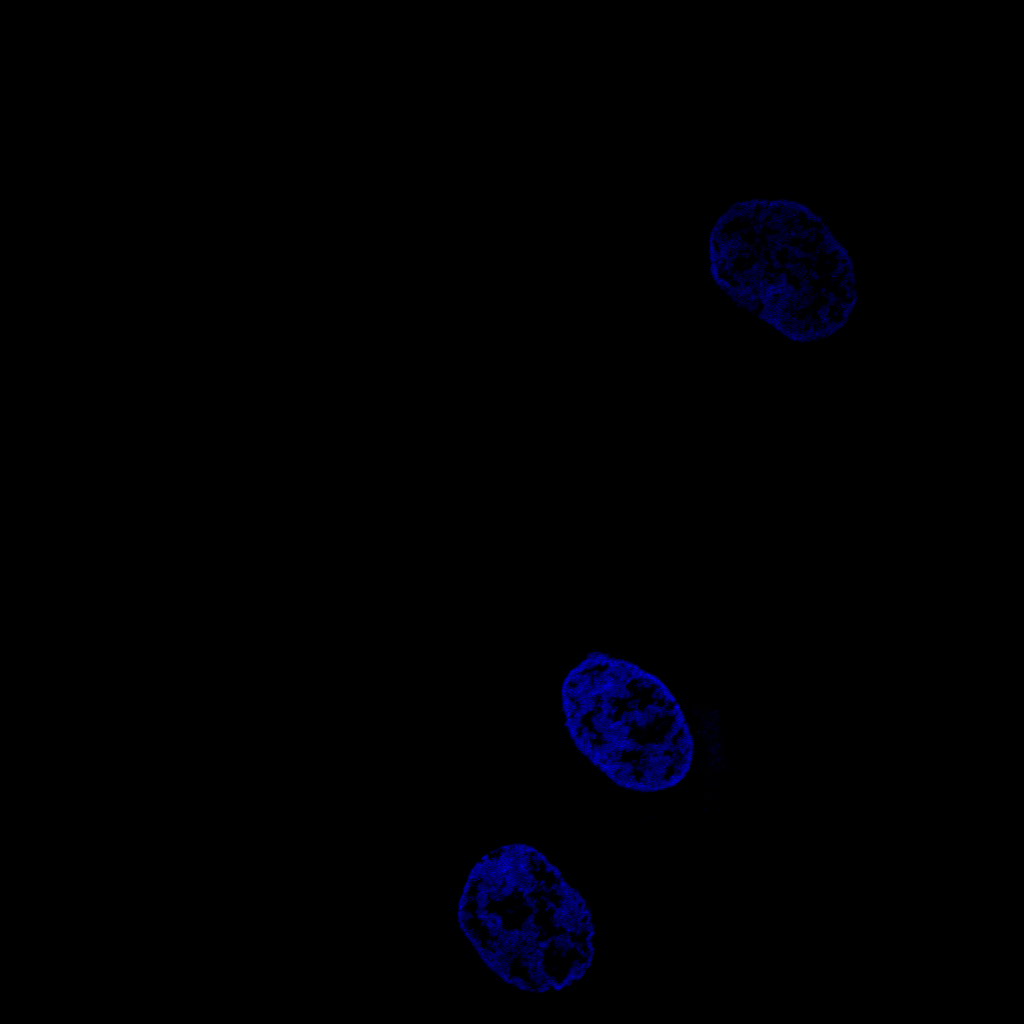

Supplement: Supplementary file 9 — Expanded View Figure and Appendix source data [file 44319_2025_513_MOESM9_ESM.zip › Expanded View Figure and Appendix source data/Expanded View Figure 5/EV 5B/PCAF 0 h/DAPI.tif]

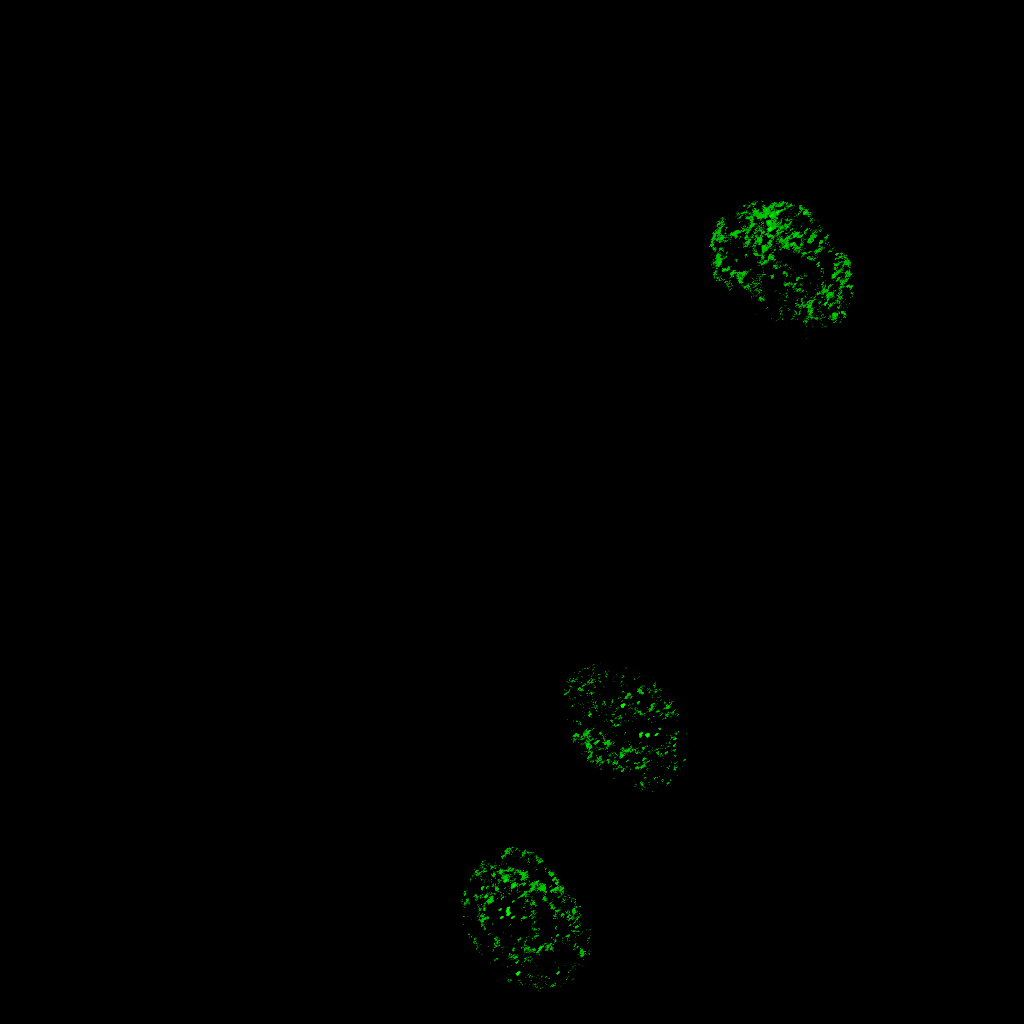

Supplement: Supplementary file 9 — Expanded View Figure and Appendix source data [file 44319_2025_513_MOESM9_ESM.zip › Expanded View Figure and Appendix source data/Expanded View Figure 5/EV 5B/PCAF 0 h/GH2AX.tif]

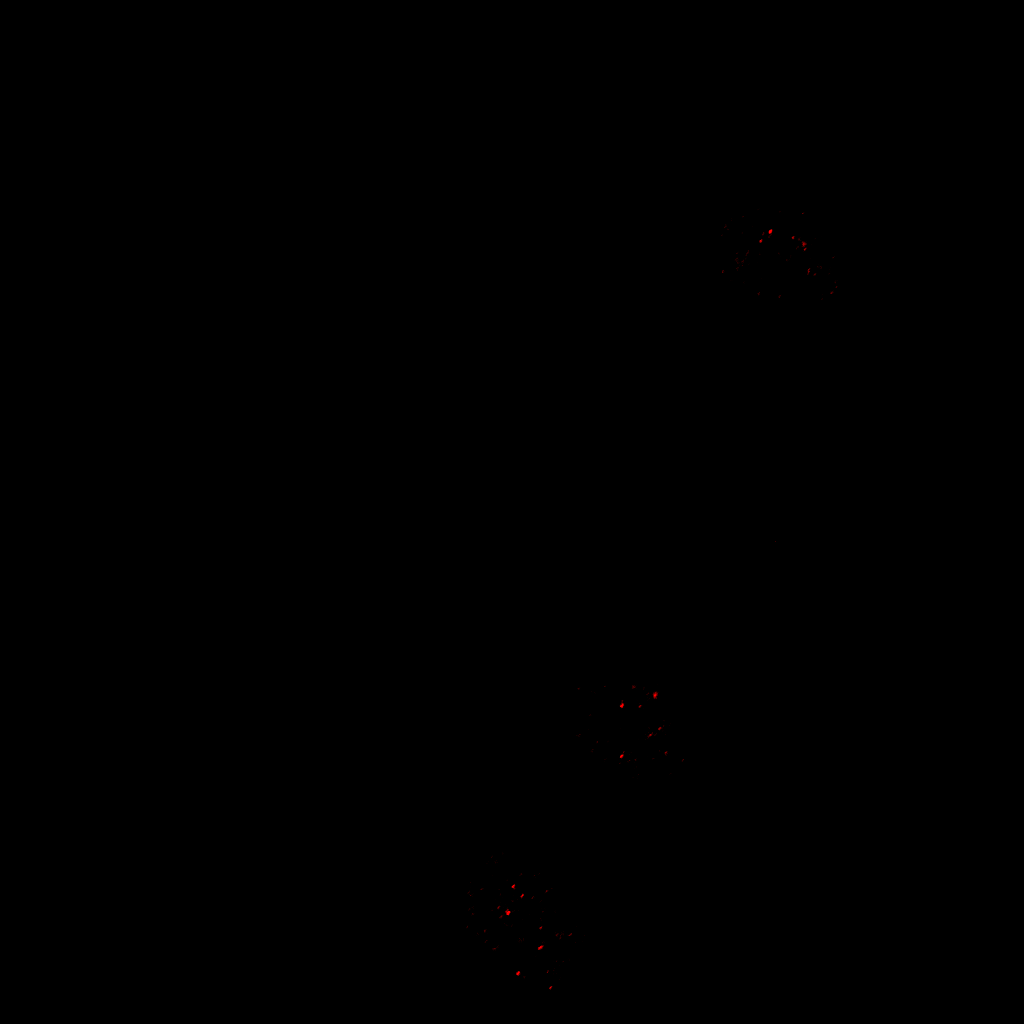

Supplement: Supplementary file 9 — Expanded View Figure and Appendix source data [file 44319_2025_513_MOESM9_ESM.zip › Expanded View Figure and Appendix source data/Expanded View Figure 5/EV 5B/PCAF 0 h/RAD51.tif]

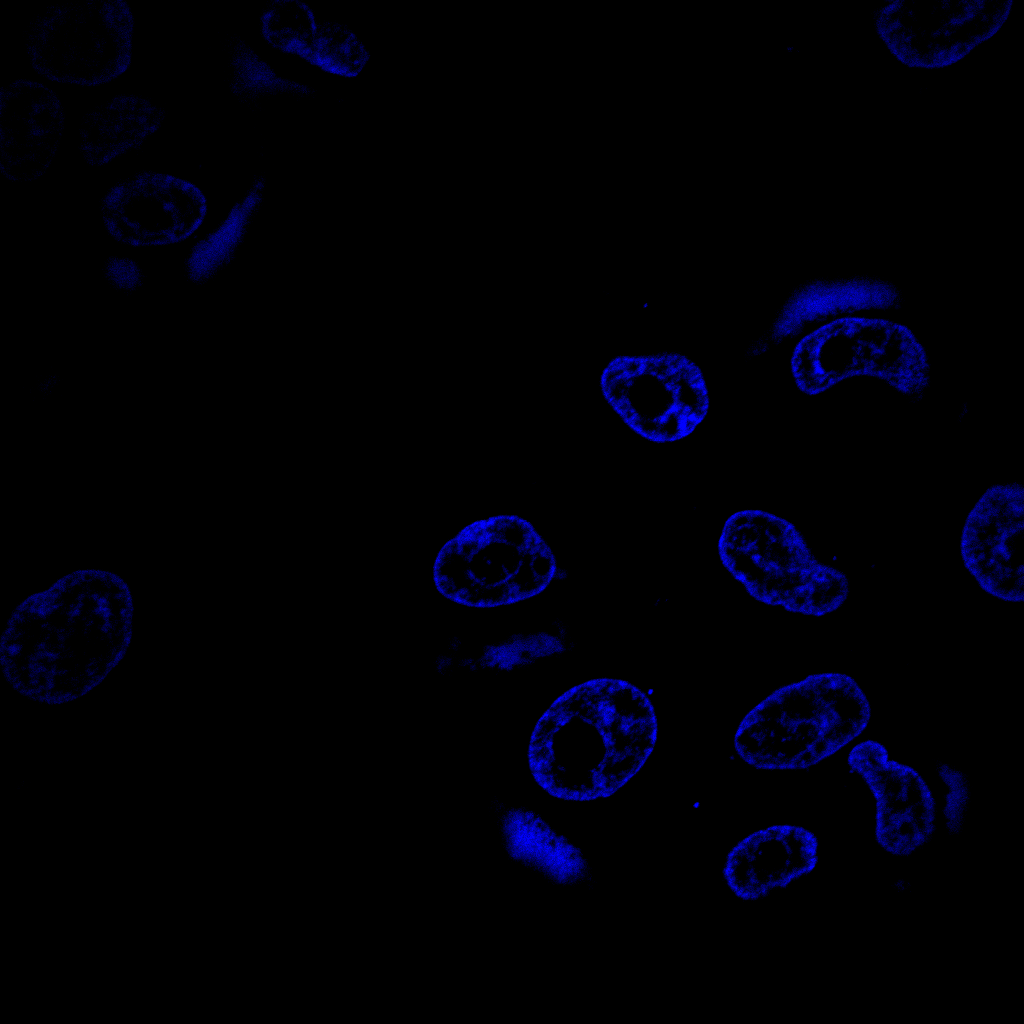

Supplement: Supplementary file 9 — Expanded View Figure and Appendix source data [file 44319_2025_513_MOESM9_ESM.zip › Expanded View Figure and Appendix source data/Expanded View Figure 5/EV 5B/PCAF 1 h/DAPI.tif]

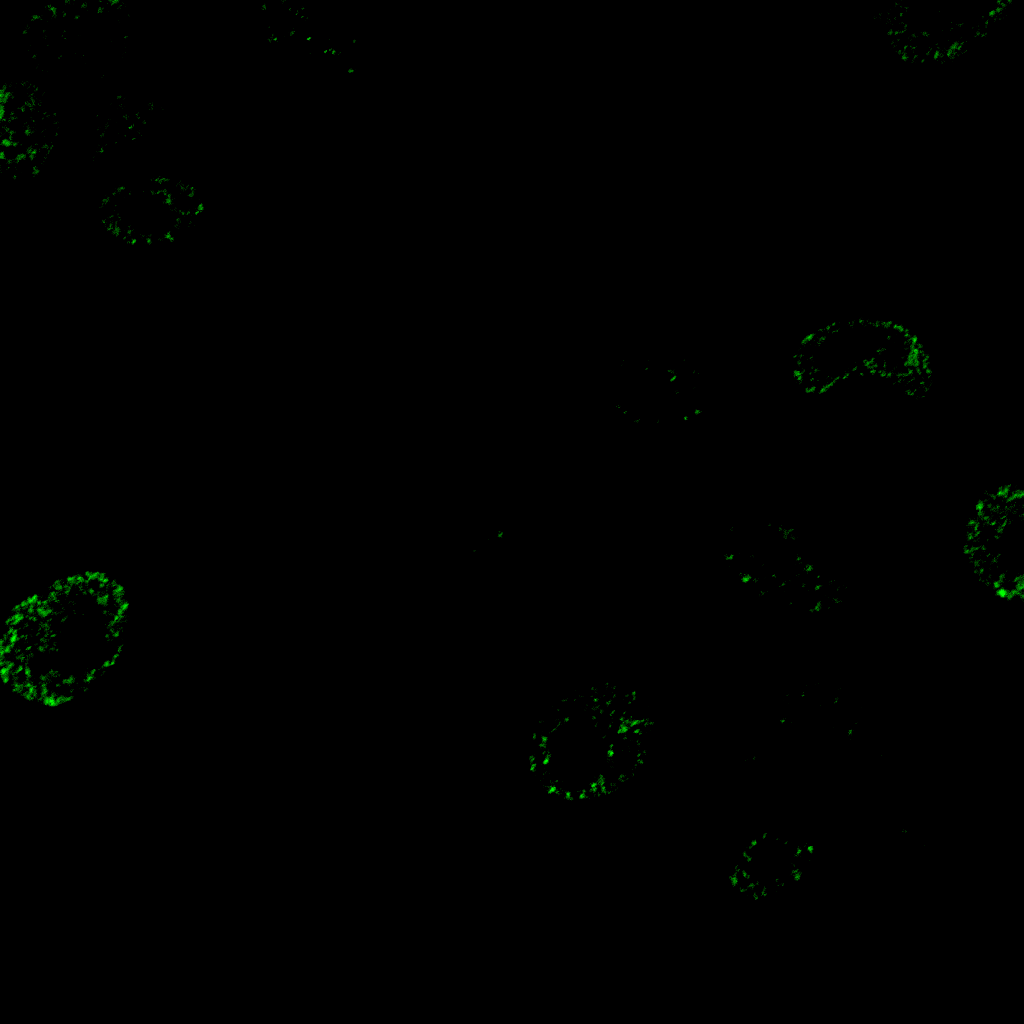

Supplement: Supplementary file 9 — Expanded View Figure and Appendix source data [file 44319_2025_513_MOESM9_ESM.zip › Expanded View Figure and Appendix source data/Expanded View Figure 5/EV 5B/PCAF 1 h/GH2AX.tif]

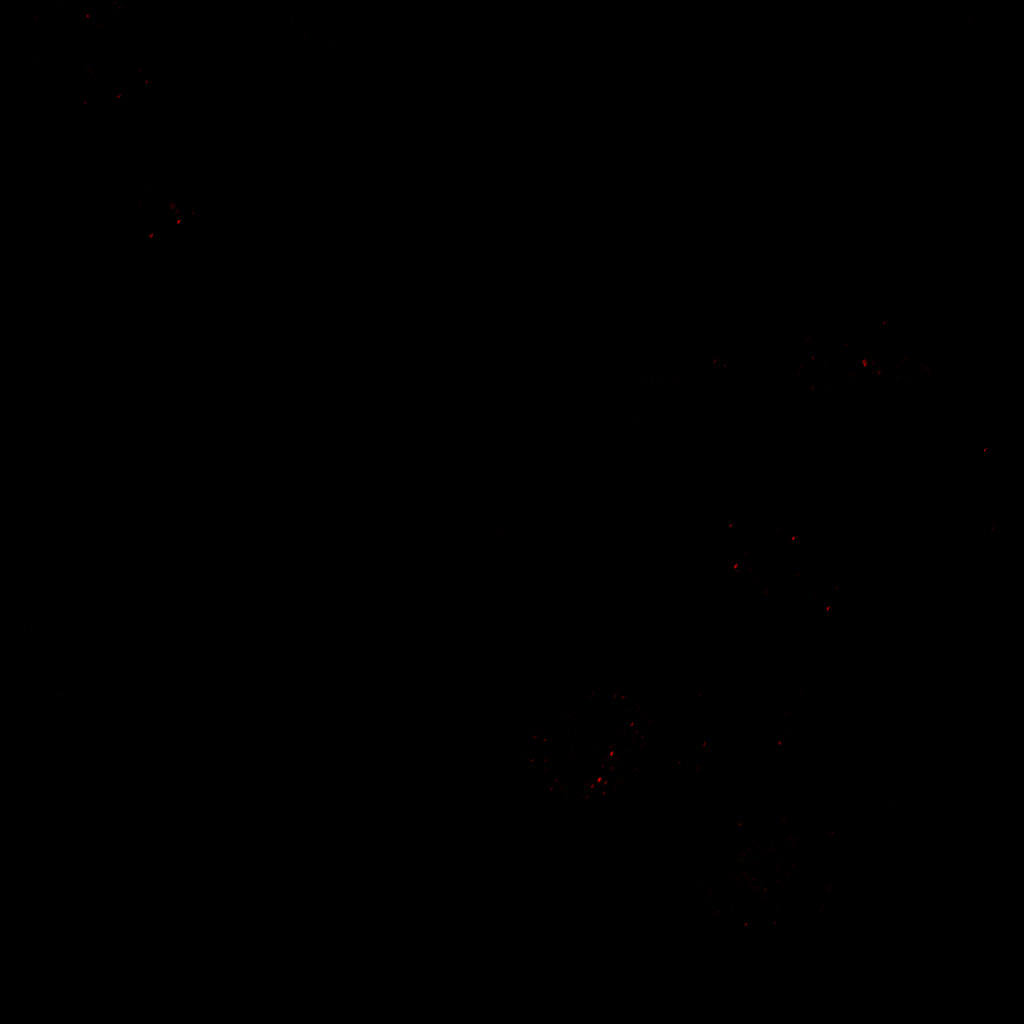

Supplement: Supplementary file 9 — Expanded View Figure and Appendix source data [file 44319_2025_513_MOESM9_ESM.zip › Expanded View Figure and Appendix source data/Expanded View Figure 5/EV 5B/PCAF 1 h/RAD51.tif]

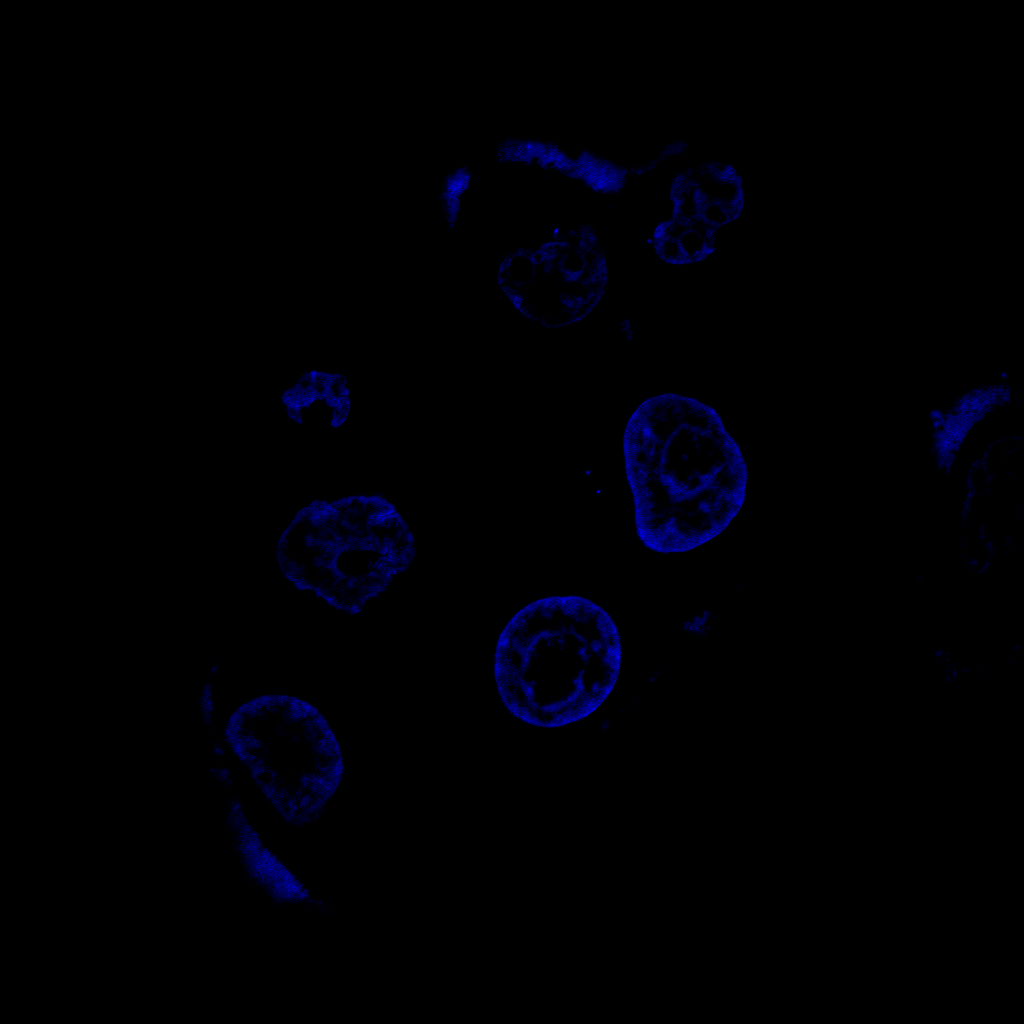

Supplement: Supplementary file 9 — Expanded View Figure and Appendix source data [file 44319_2025_513_MOESM9_ESM.zip › Expanded View Figure and Appendix source data/Expanded View Figure 5/EV 5B/PCAF 2 h/DAPI.tif]

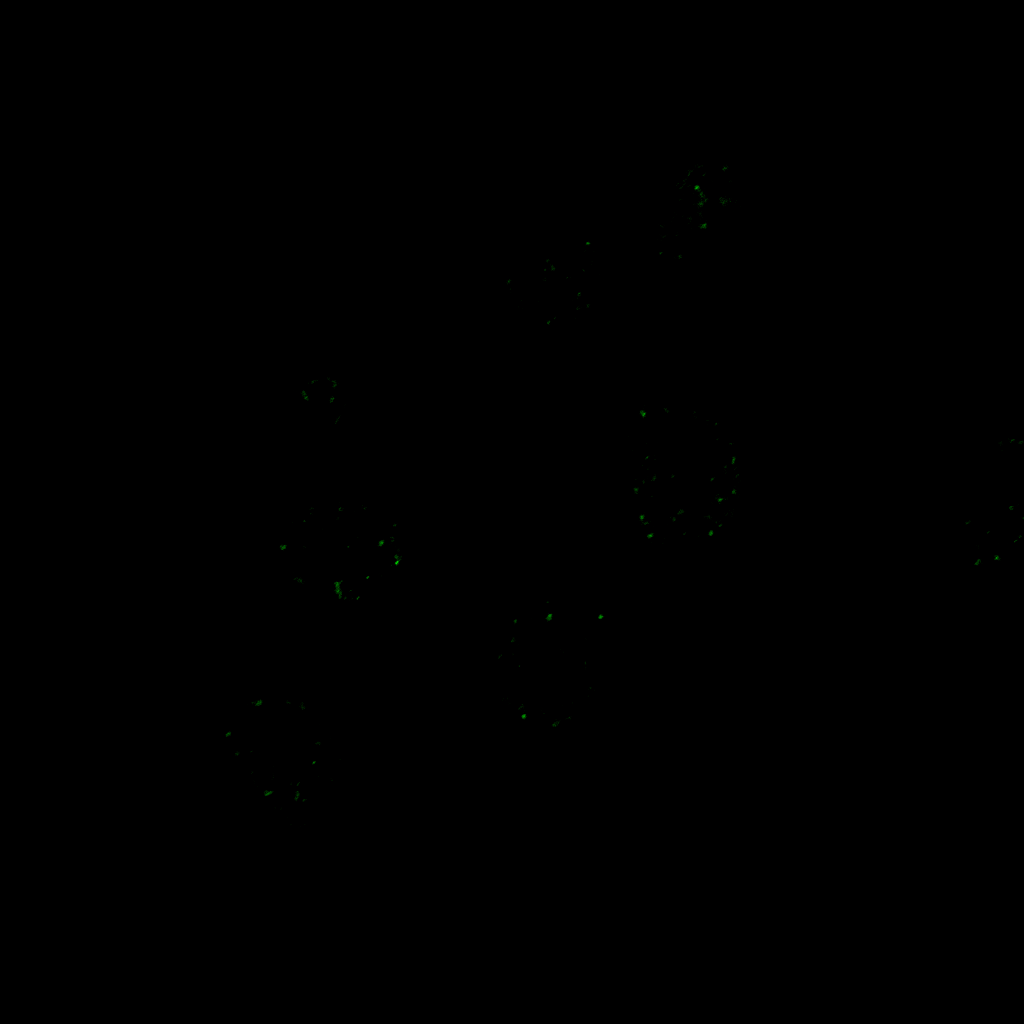

Supplement: Supplementary file 9 — Expanded View Figure and Appendix source data [file 44319_2025_513_MOESM9_ESM.zip › Expanded View Figure and Appendix source data/Expanded View Figure 5/EV 5B/PCAF 2 h/GH2AX.tif]

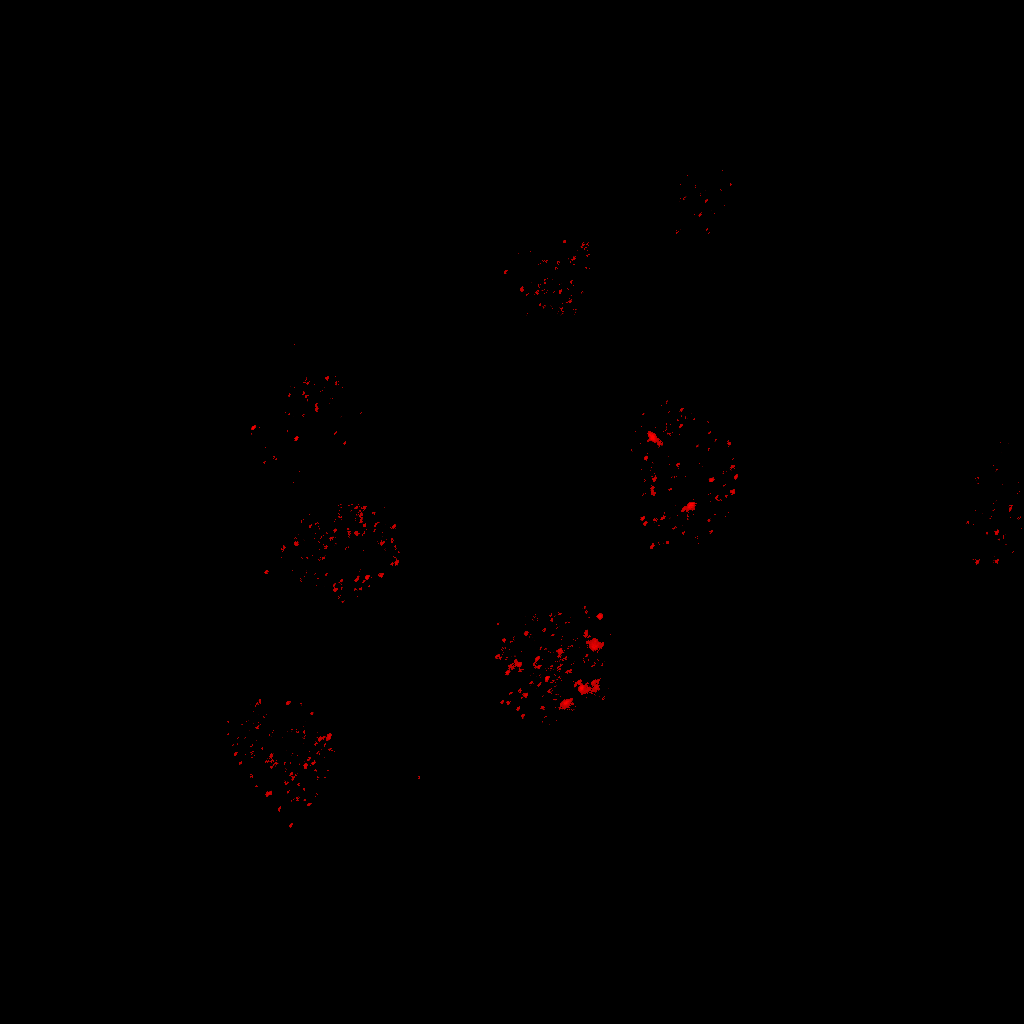

Supplement: Supplementary file 9 — Expanded View Figure and Appendix source data [file 44319_2025_513_MOESM9_ESM.zip › Expanded View Figure and Appendix source data/Expanded View Figure 5/EV 5B/PCAF 2 h/RAD51.tif]

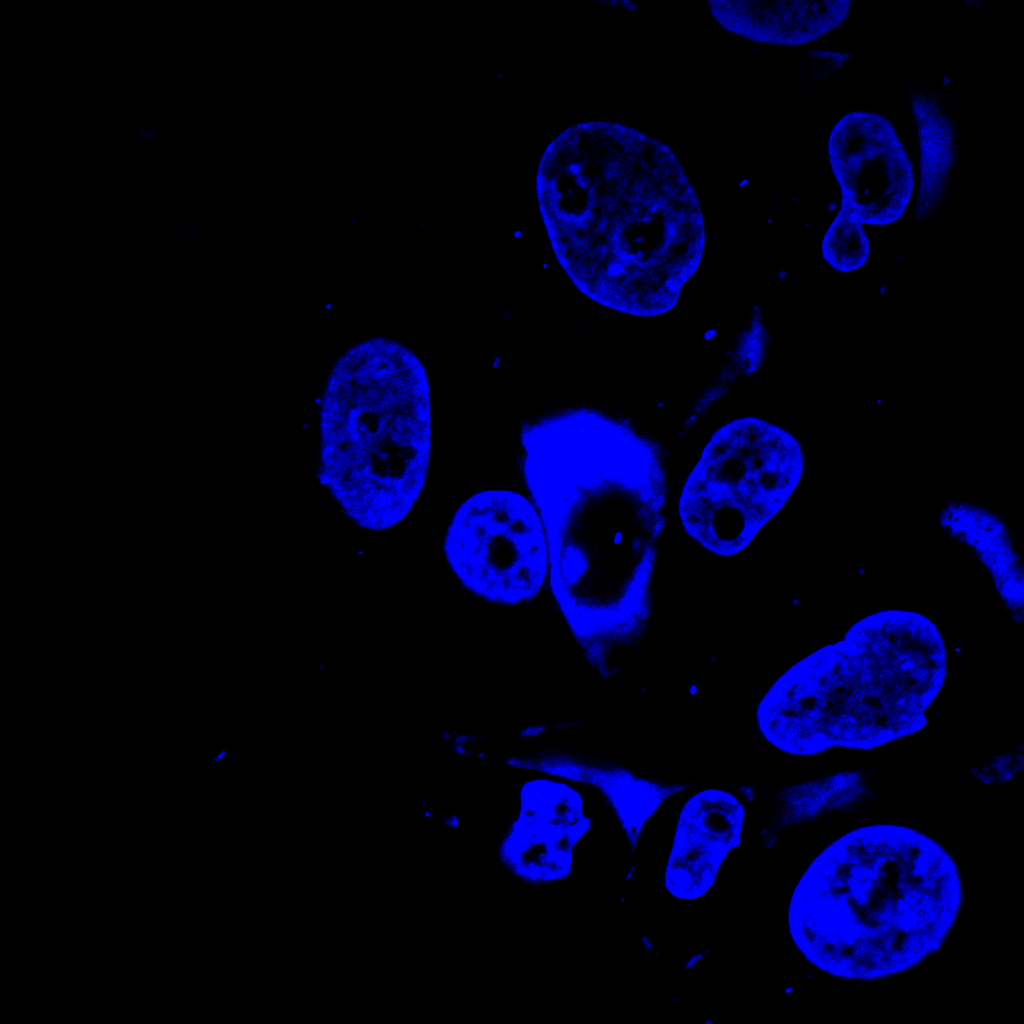

Supplement: Supplementary file 9 — Expanded View Figure and Appendix source data [file 44319_2025_513_MOESM9_ESM.zip › Expanded View Figure and Appendix source data/Expanded View Figure 5/EV 5B/PCAF 24 h/DAPI.tif]

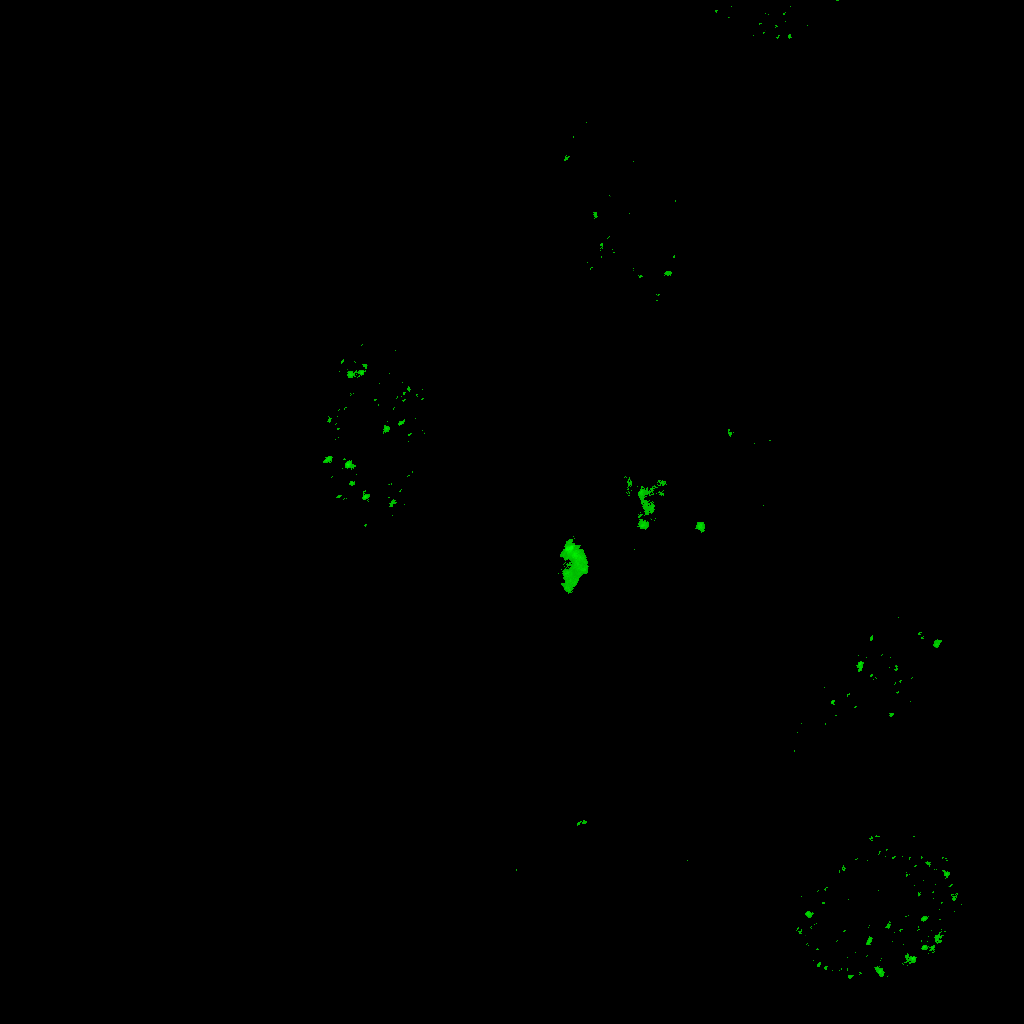

Supplement: Supplementary file 9 — Expanded View Figure and Appendix source data [file 44319_2025_513_MOESM9_ESM.zip › Expanded View Figure and Appendix source data/Expanded View Figure 5/EV 5B/PCAF 24 h/GH2AX.tif]

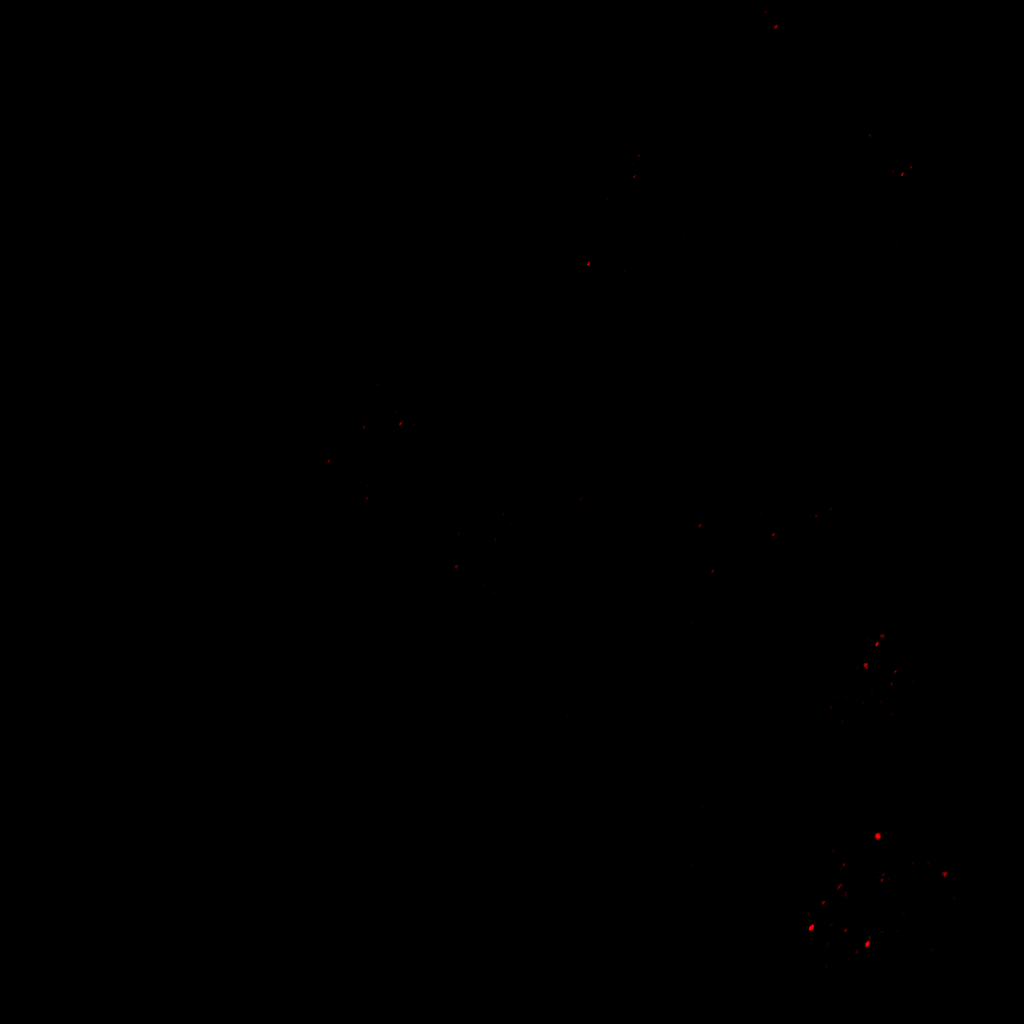

Supplement: Supplementary file 9 — Expanded View Figure and Appendix source data [file 44319_2025_513_MOESM9_ESM.zip › Expanded View Figure and Appendix source data/Expanded View Figure 5/EV 5B/PCAF 24 h/RAD51.tif]

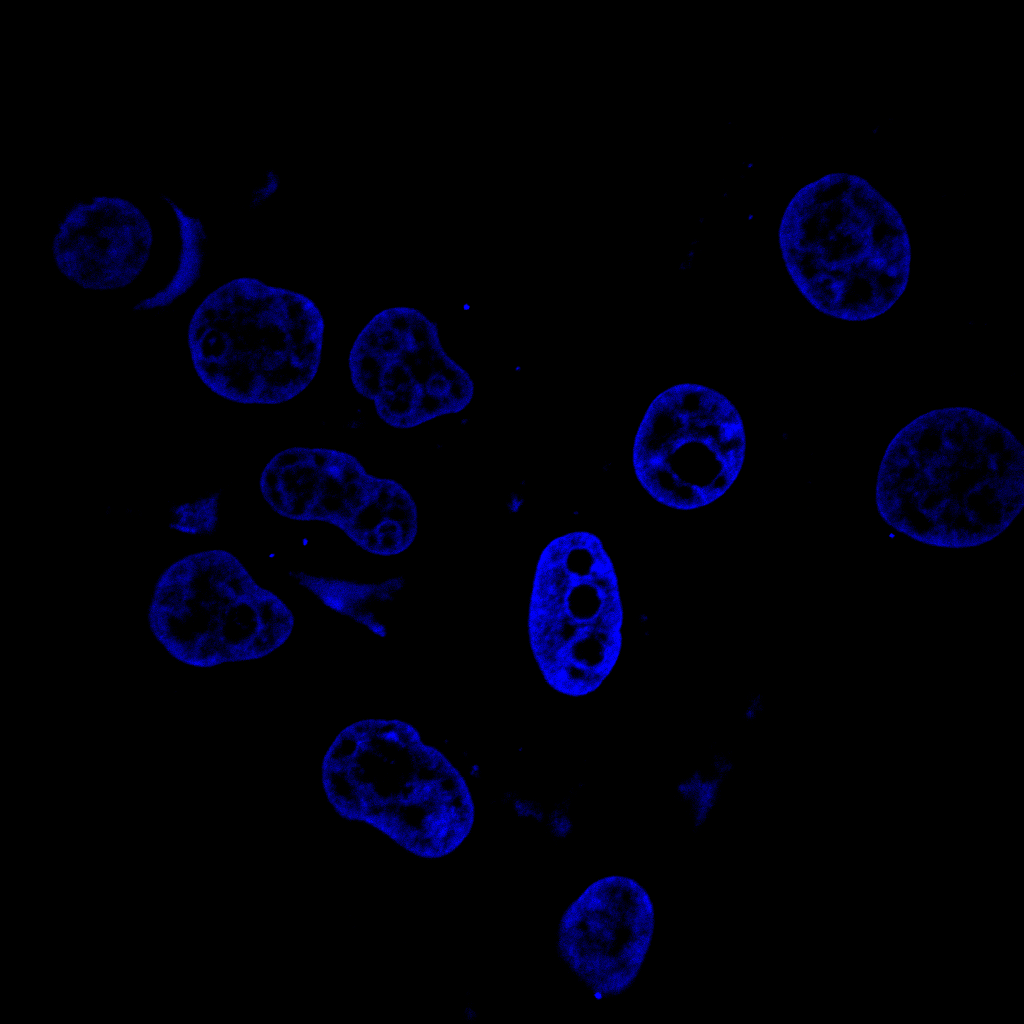

Supplement: Supplementary file 9 — Expanded View Figure and Appendix source data [file 44319_2025_513_MOESM9_ESM.zip › Expanded View Figure and Appendix source data/Expanded View Figure 5/EV 5B/PCAF 4 h/DAPI.tif]

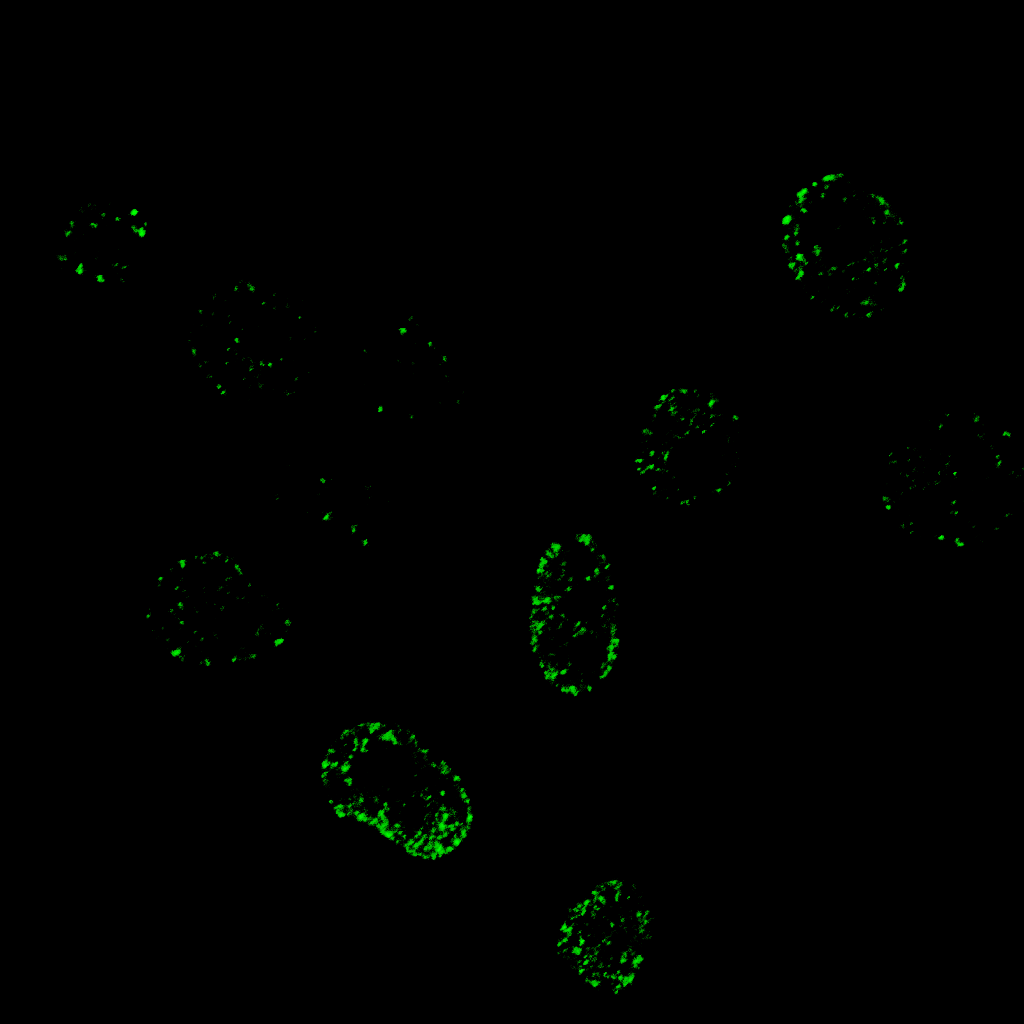

Supplement: Supplementary file 9 — Expanded View Figure and Appendix source data [file 44319_2025_513_MOESM9_ESM.zip › Expanded View Figure and Appendix source data/Expanded View Figure 5/EV 5B/PCAF 4 h/GH2AX.tif]

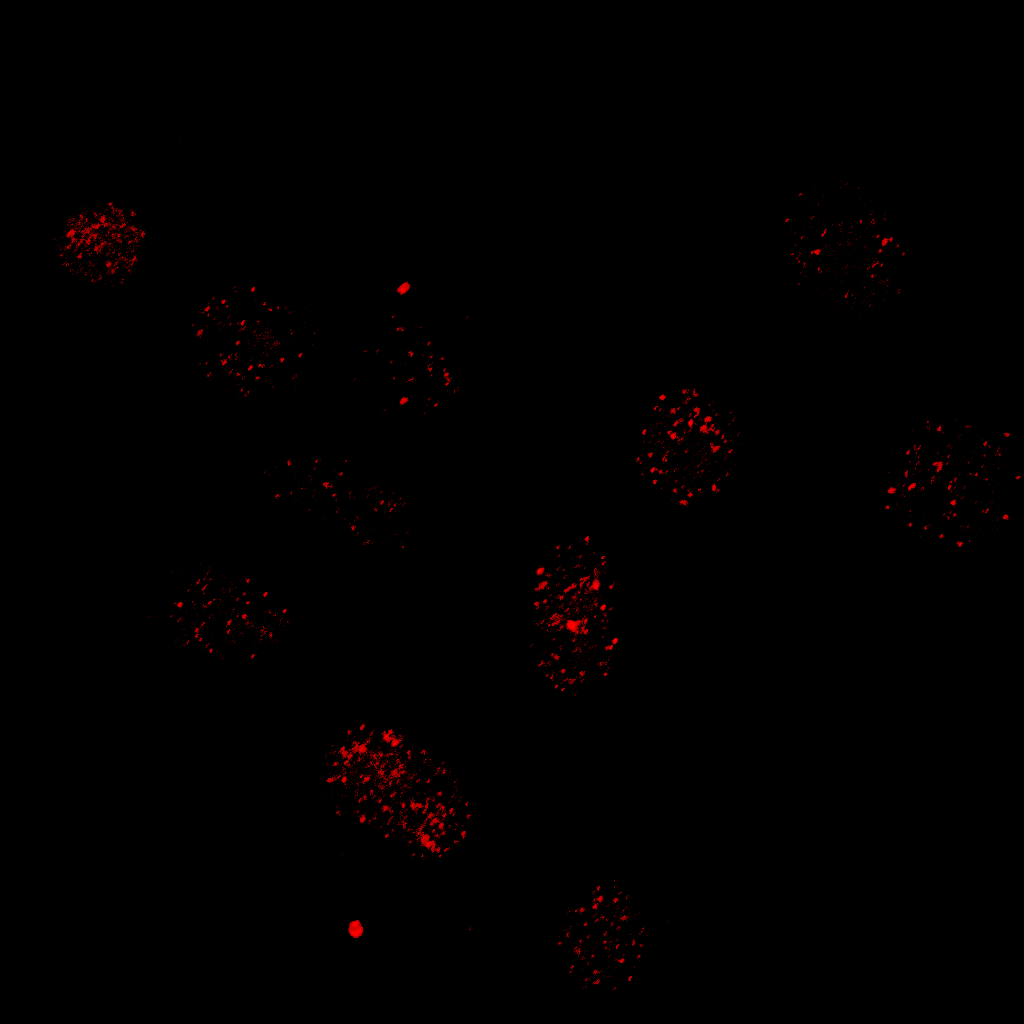

Supplement: Supplementary file 9 — Expanded View Figure and Appendix source data [file 44319_2025_513_MOESM9_ESM.zip › Expanded View Figure and Appendix source data/Expanded View Figure 5/EV 5B/PCAF 4 h/RAD51.tif]

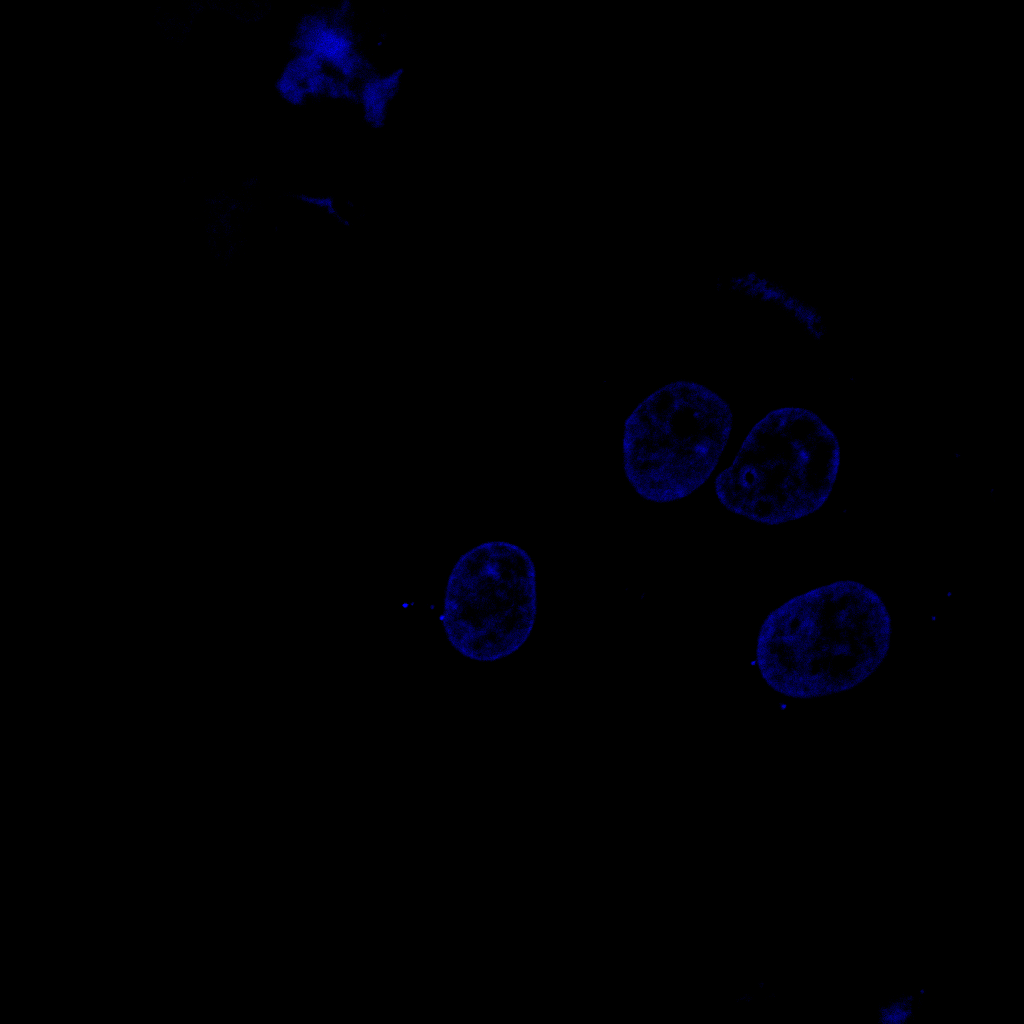

Supplement: Supplementary file 9 — Expanded View Figure and Appendix source data [file 44319_2025_513_MOESM9_ESM.zip › Expanded View Figure and Appendix source data/Expanded View Figure 5/EV 5B/PCAF 8 h/DAPI.tif]

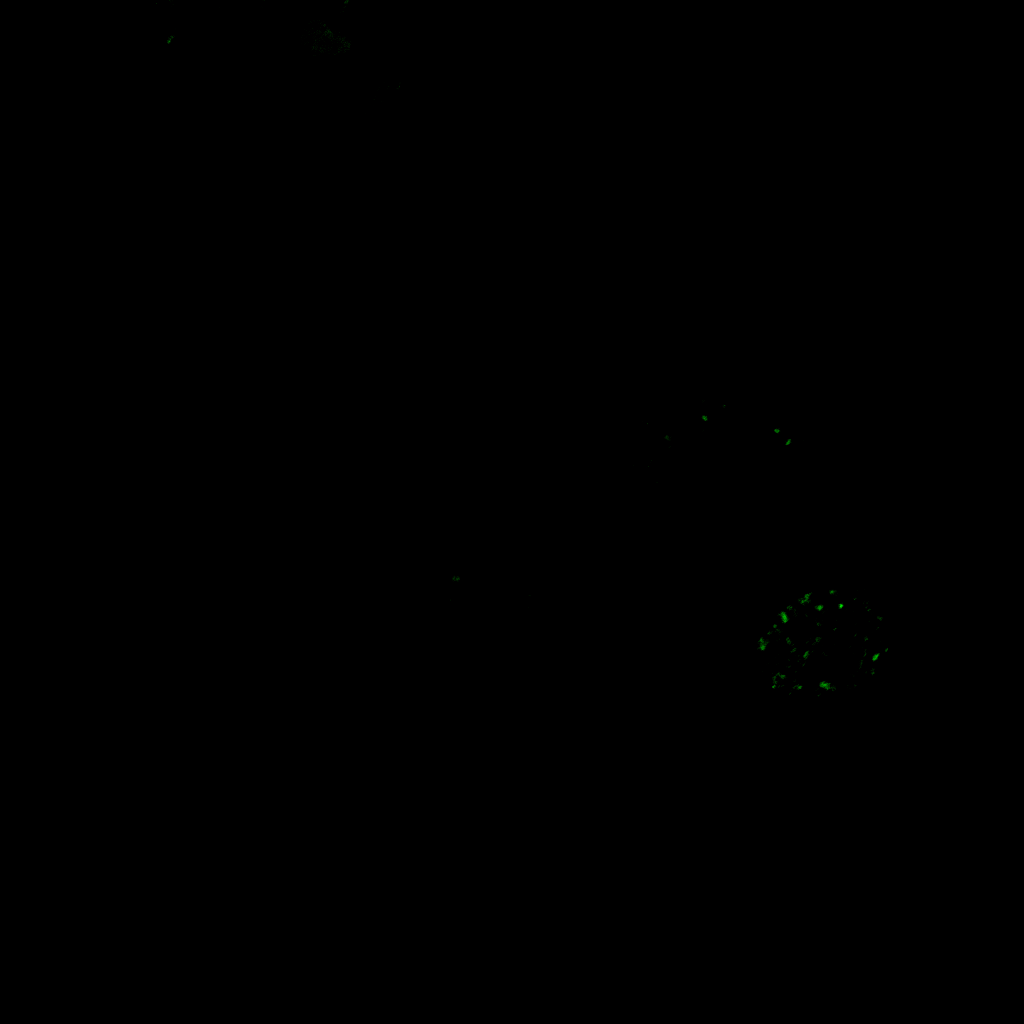

Supplement: Supplementary file 9 — Expanded View Figure and Appendix source data [file 44319_2025_513_MOESM9_ESM.zip › Expanded View Figure and Appendix source data/Expanded View Figure 5/EV 5B/PCAF 8 h/GH2AX.tif]

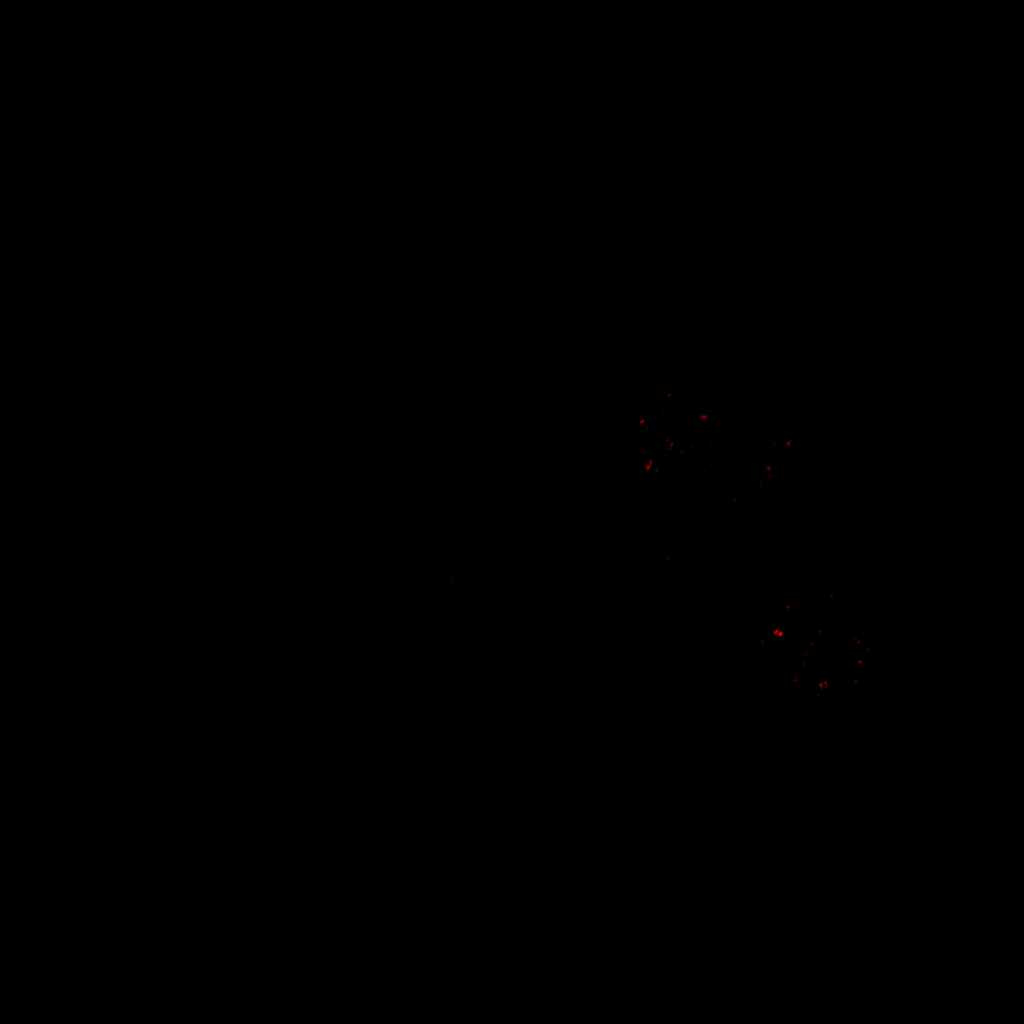

Supplement: Supplementary file 9 — Expanded View Figure and Appendix source data [file 44319_2025_513_MOESM9_ESM.zip › Expanded View Figure and Appendix source data/Expanded View Figure 5/EV 5B/PCAF 8 h/RAD51.tif]

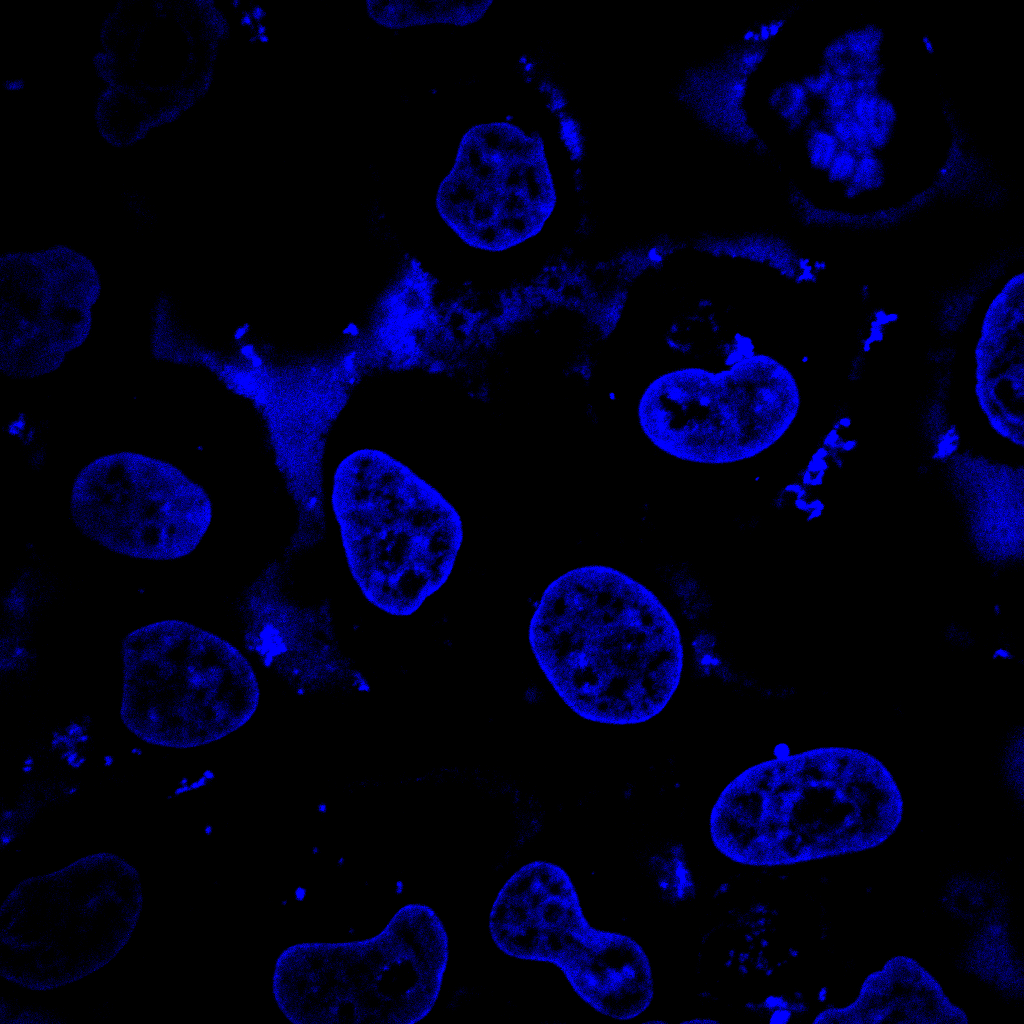

Supplement: Supplementary file 9 — Expanded View Figure and Appendix source data [file 44319_2025_513_MOESM9_ESM.zip › Expanded View Figure and Appendix source data/Expanded View Figure 5/EV 5B/PCAF ETO-/DAPI.tif]

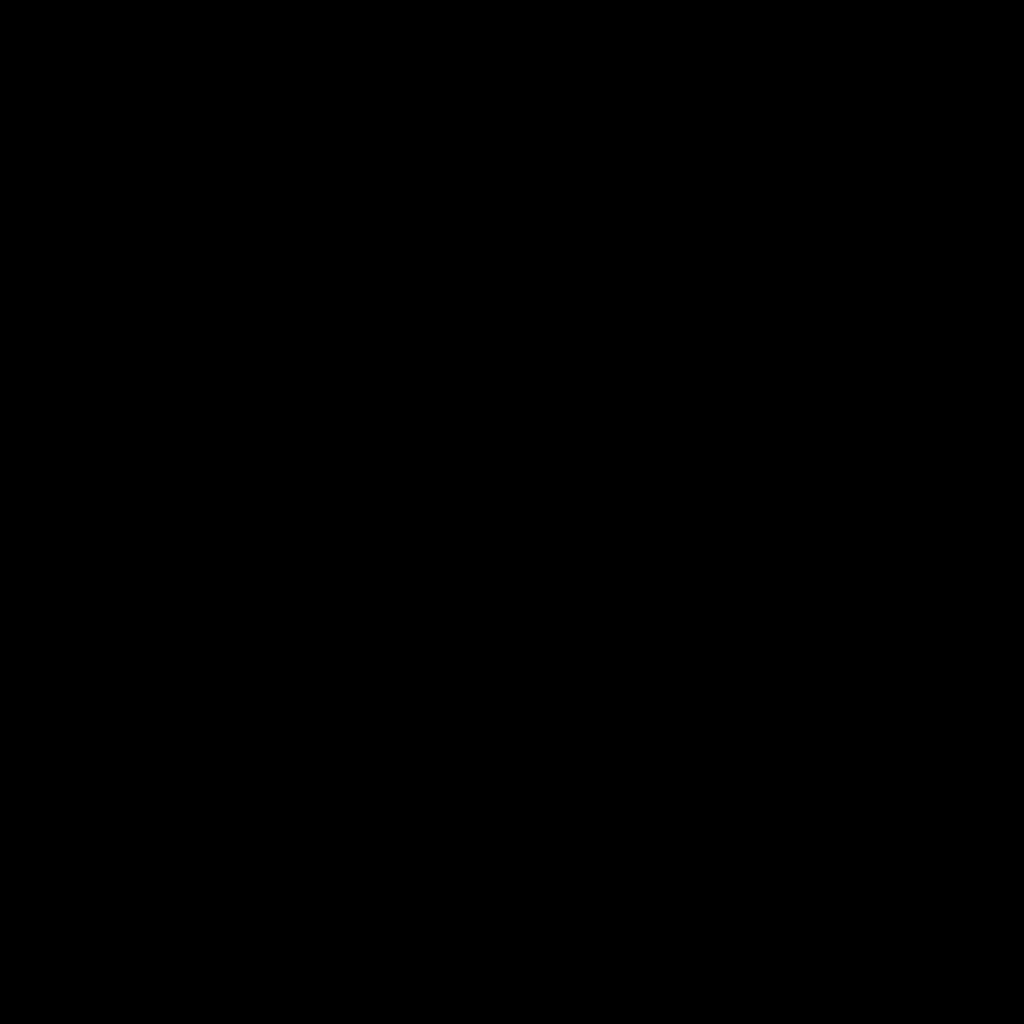

Supplement: Supplementary file 9 — Expanded View Figure and Appendix source data [file 44319_2025_513_MOESM9_ESM.zip › Expanded View Figure and Appendix source data/Expanded View Figure 5/EV 5B/PCAF ETO-/GH2AX.tif]

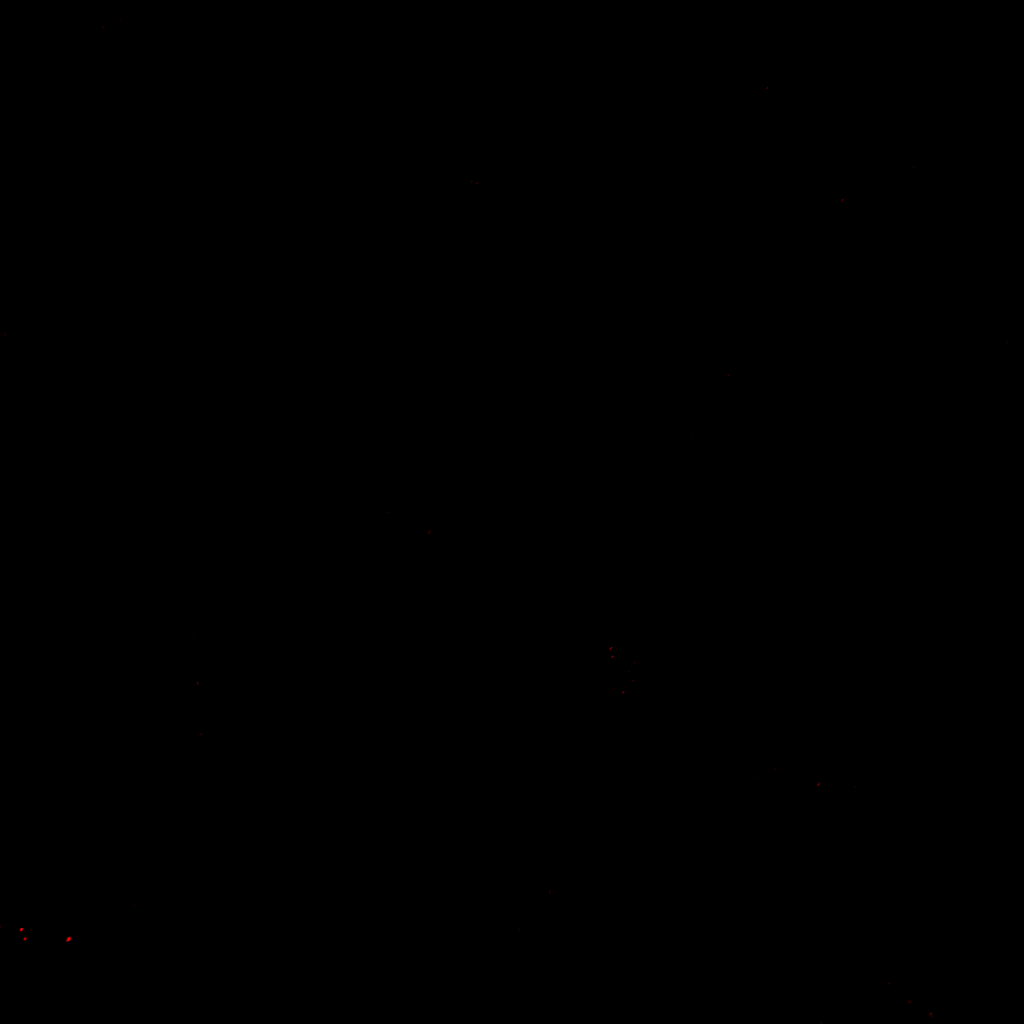

Supplement: Supplementary file 9 — Expanded View Figure and Appendix source data [file 44319_2025_513_MOESM9_ESM.zip › Expanded View Figure and Appendix source data/Expanded View Figure 5/EV 5B/PCAF ETO-/RAD51.tif]

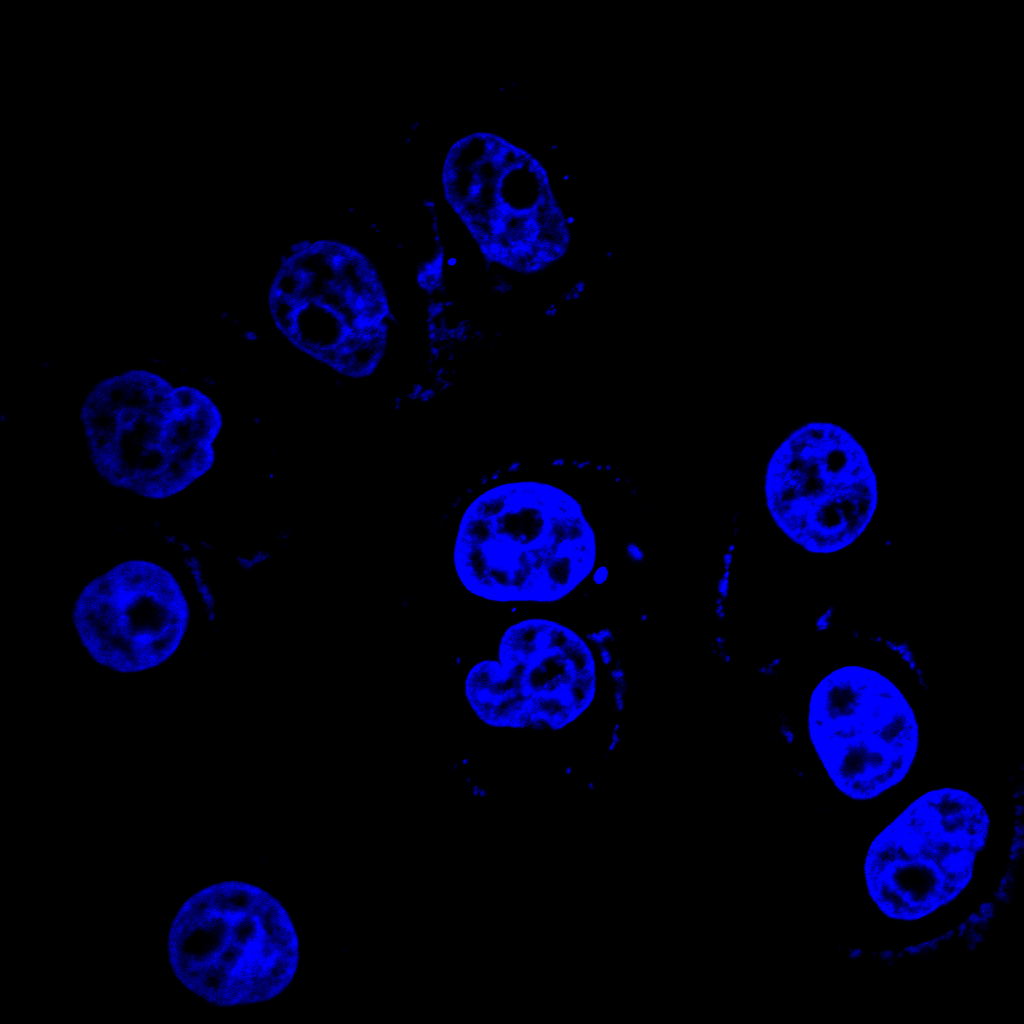

Supplement: Supplementary file 9 — Expanded View Figure and Appendix source data [file 44319_2025_513_MOESM9_ESM.zip › Expanded View Figure and Appendix source data/Expanded View Figure 5/EV 5D/sgNC 1 h/DAPI.tif]

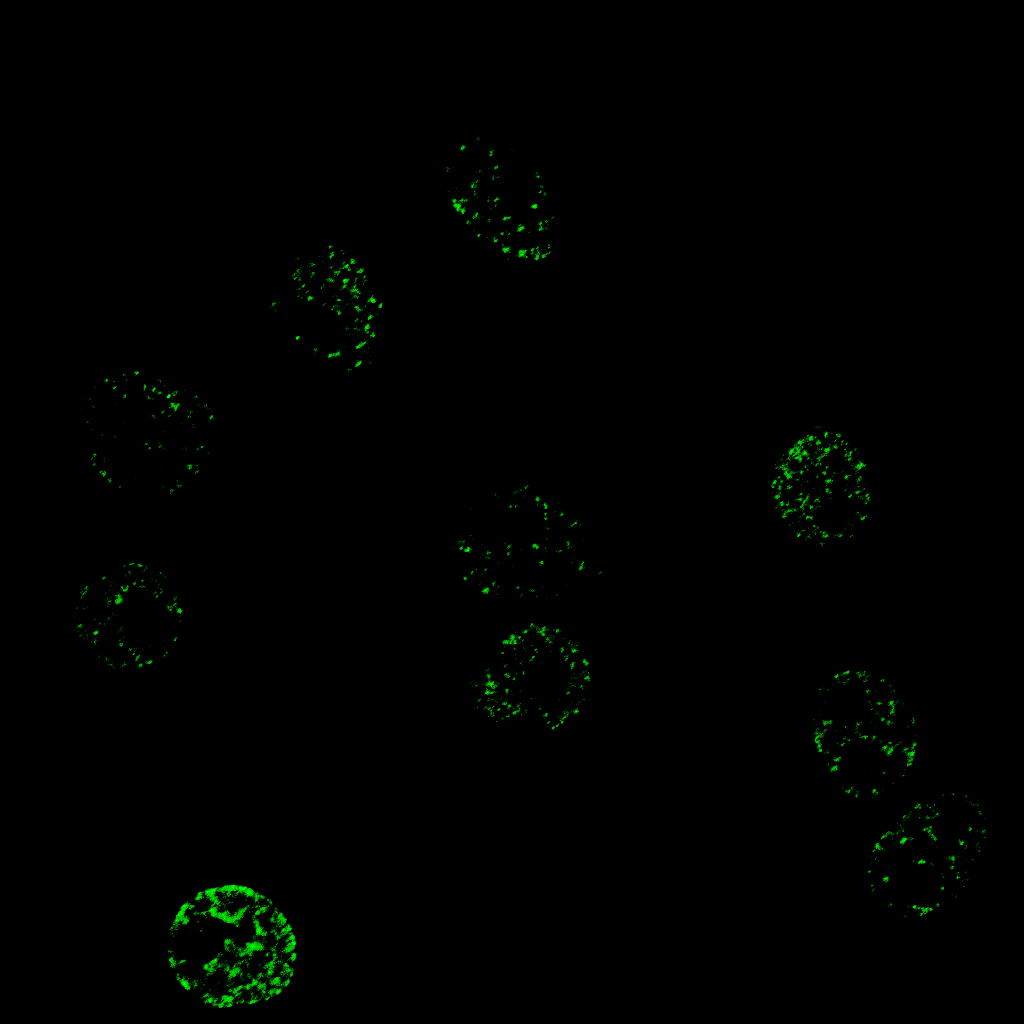

Supplement: Supplementary file 9 — Expanded View Figure and Appendix source data [file 44319_2025_513_MOESM9_ESM.zip › Expanded View Figure and Appendix source data/Expanded View Figure 5/EV 5D/sgNC 1 h/GH2AX.tif]

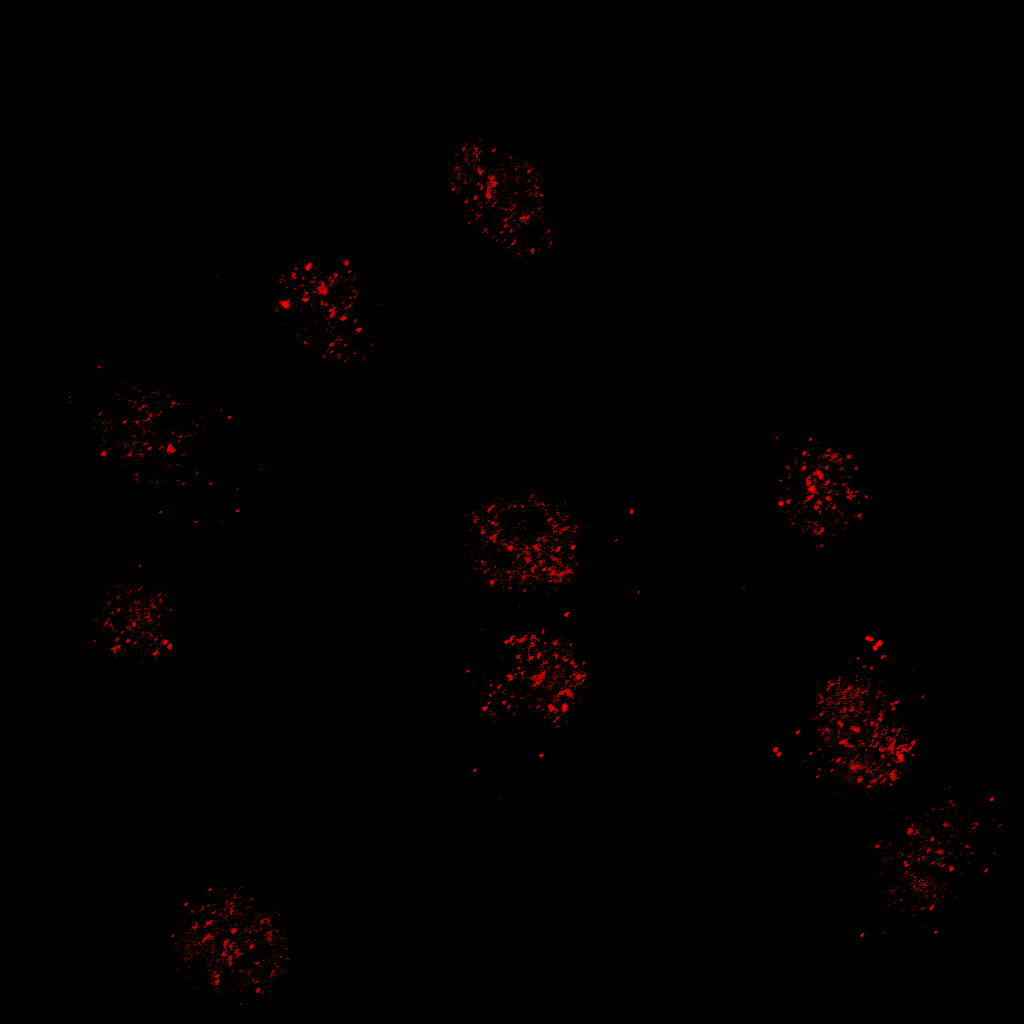

Supplement: Supplementary file 9 — Expanded View Figure and Appendix source data [file 44319_2025_513_MOESM9_ESM.zip › Expanded View Figure and Appendix source data/Expanded View Figure 5/EV 5D/sgNC 1 h/RAD51.tif]

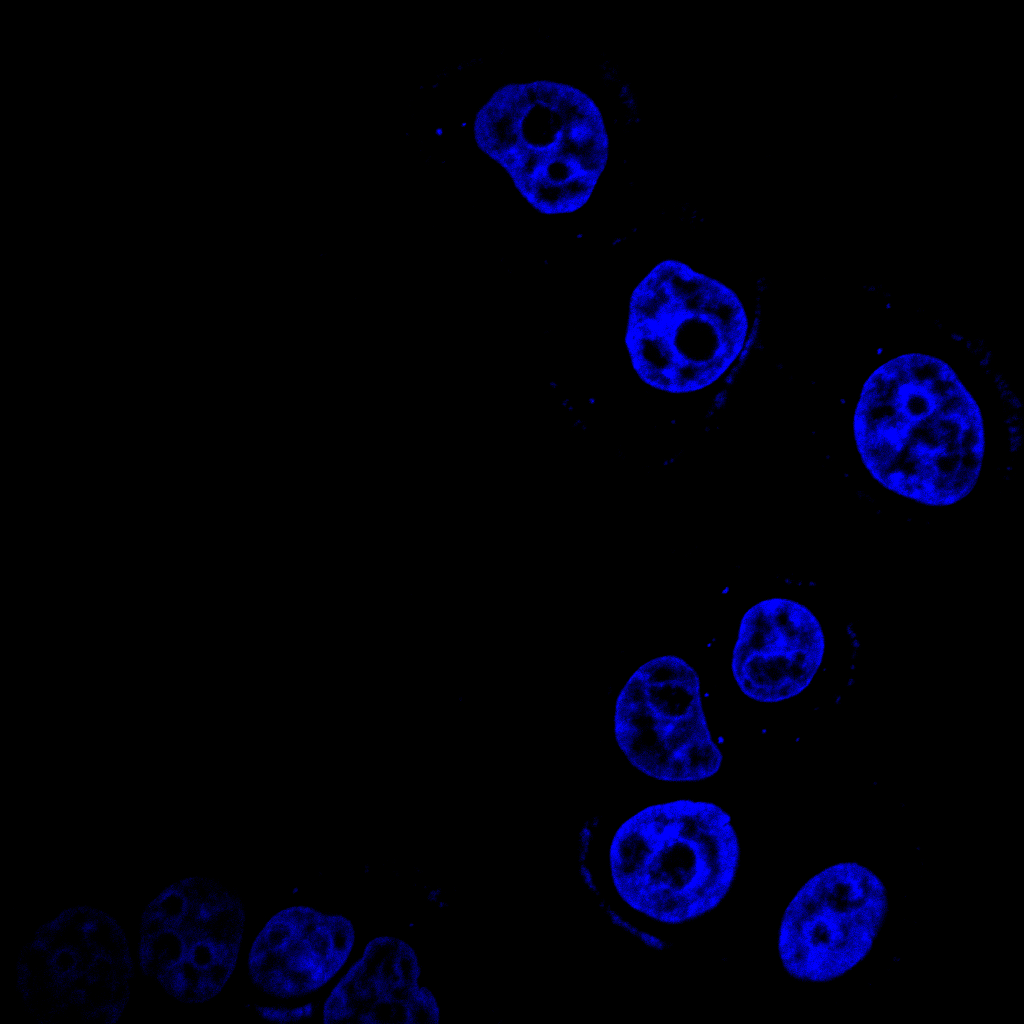

Supplement: Supplementary file 9 — Expanded View Figure and Appendix source data [file 44319_2025_513_MOESM9_ESM.zip › Expanded View Figure and Appendix source data/Expanded View Figure 5/EV 5D/sgNC 4 h/DAPI.tif]

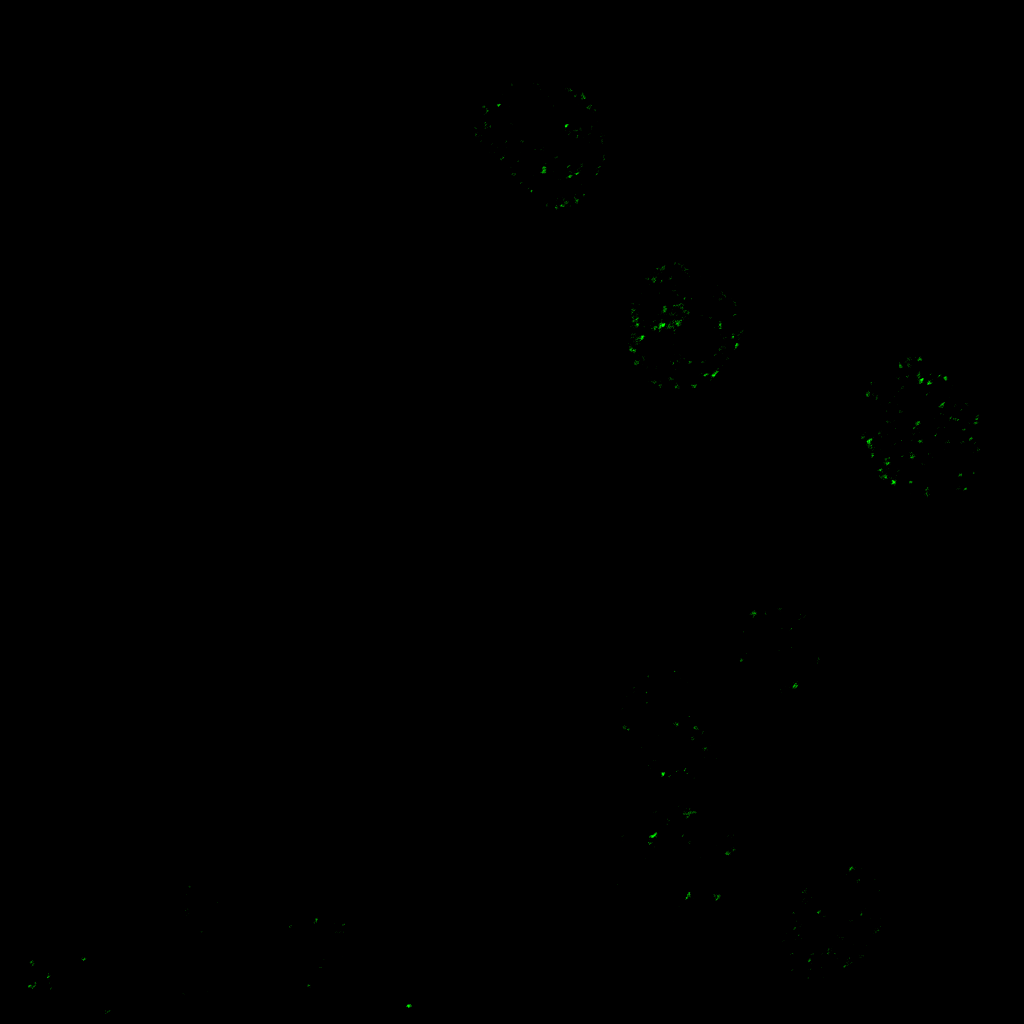

Supplement: Supplementary file 9 — Expanded View Figure and Appendix source data [file 44319_2025_513_MOESM9_ESM.zip › Expanded View Figure and Appendix source data/Expanded View Figure 5/EV 5D/sgNC 4 h/GH2AX.tif]

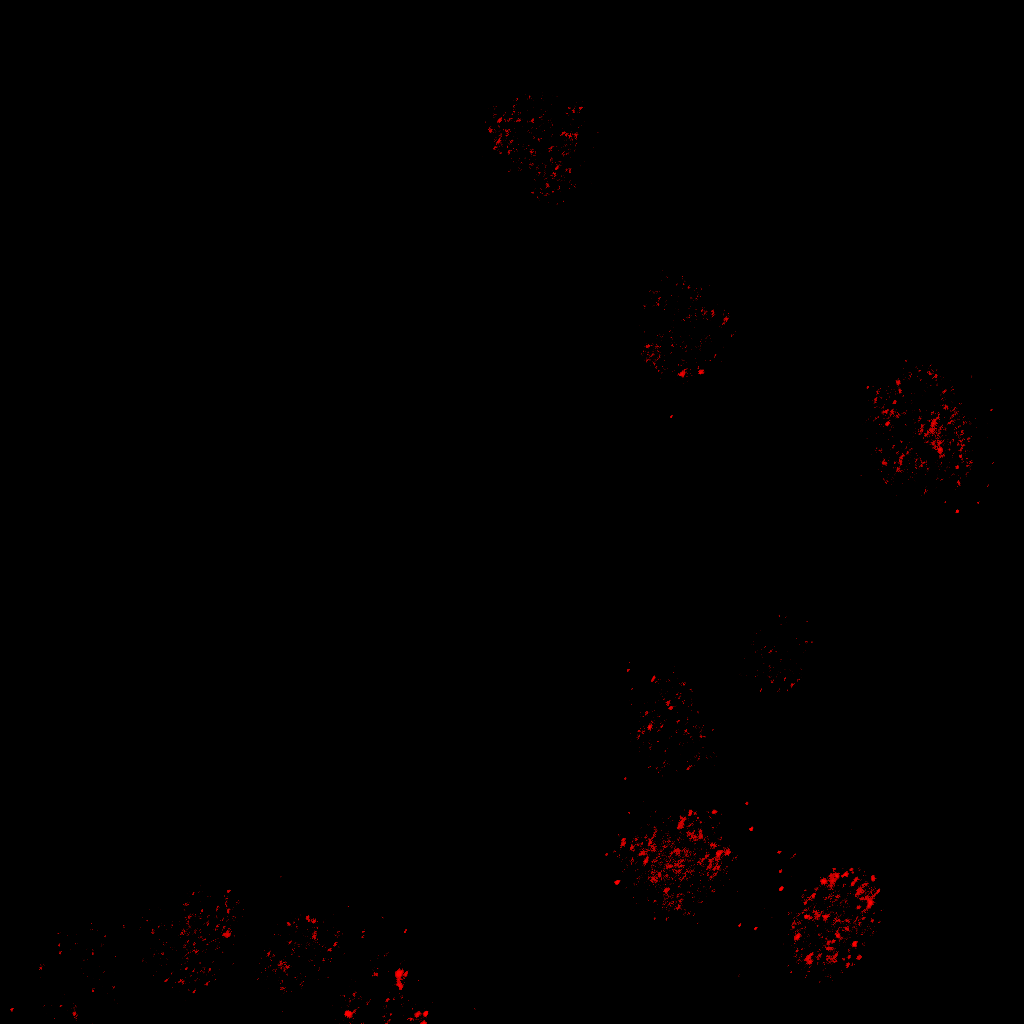

Supplement: Supplementary file 9 — Expanded View Figure and Appendix source data [file 44319_2025_513_MOESM9_ESM.zip › Expanded View Figure and Appendix source data/Expanded View Figure 5/EV 5D/sgNC 4 h/RAD51.tif]

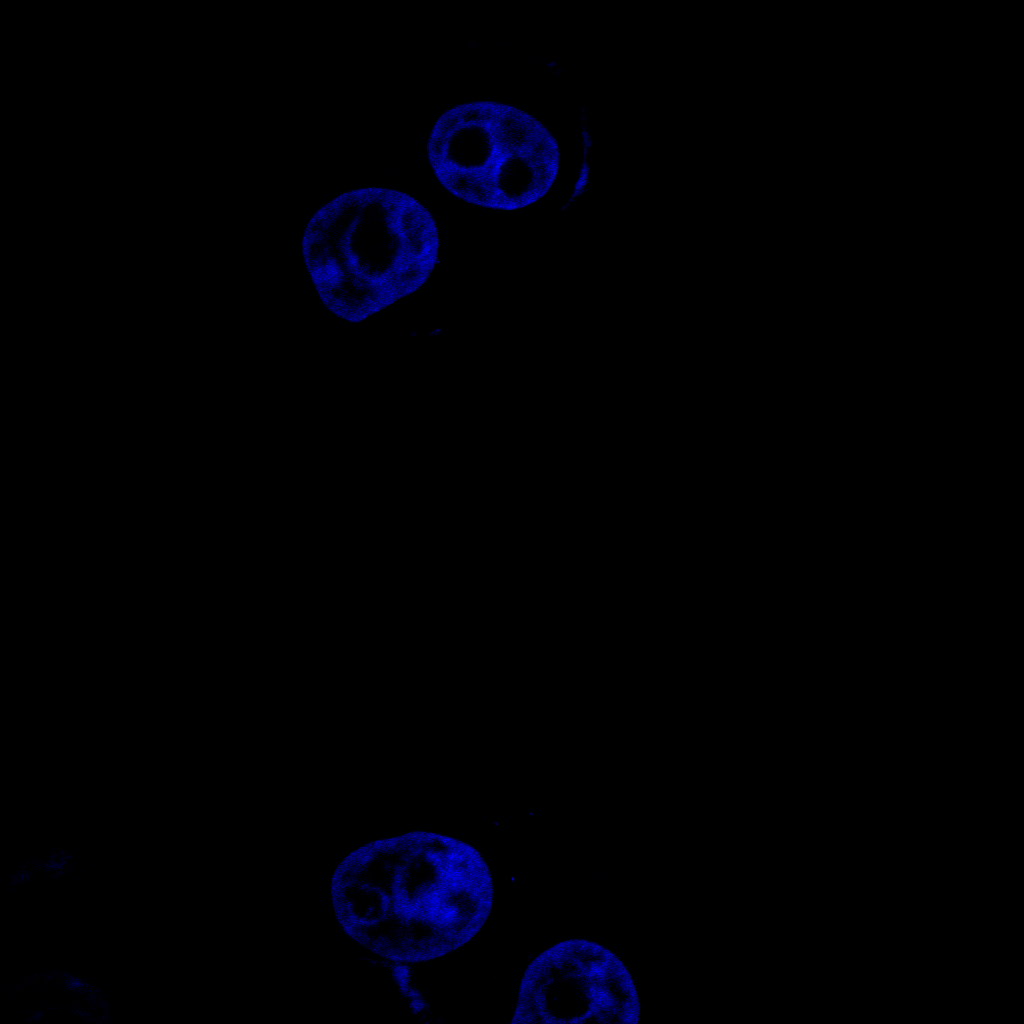

Supplement: Supplementary file 9 — Expanded View Figure and Appendix source data [file 44319_2025_513_MOESM9_ESM.zip › Expanded View Figure and Appendix source data/Expanded View Figure 5/EV 5D/sgNC 8 h/DAPI.tif]

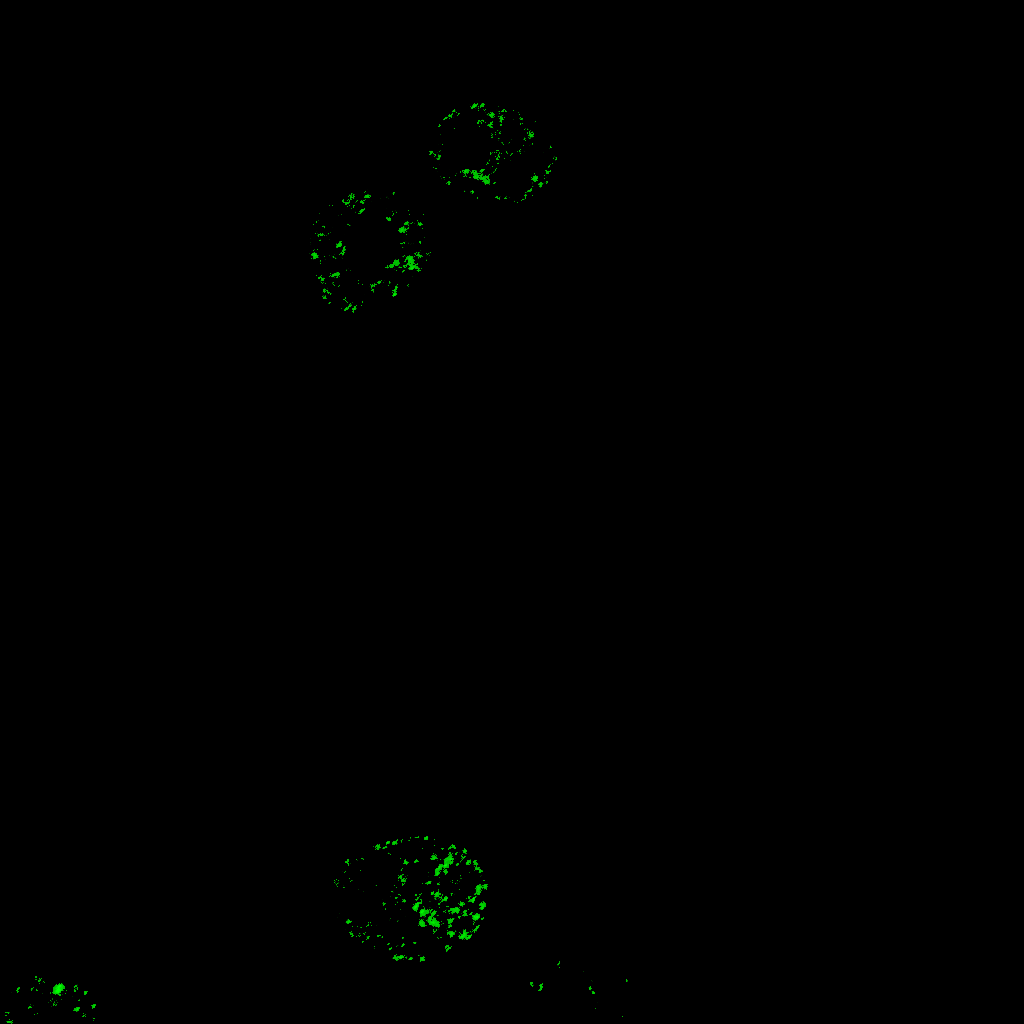

Supplement: Supplementary file 9 — Expanded View Figure and Appendix source data [file 44319_2025_513_MOESM9_ESM.zip › Expanded View Figure and Appendix source data/Expanded View Figure 5/EV 5D/sgNC 8 h/GH2AX.tif]

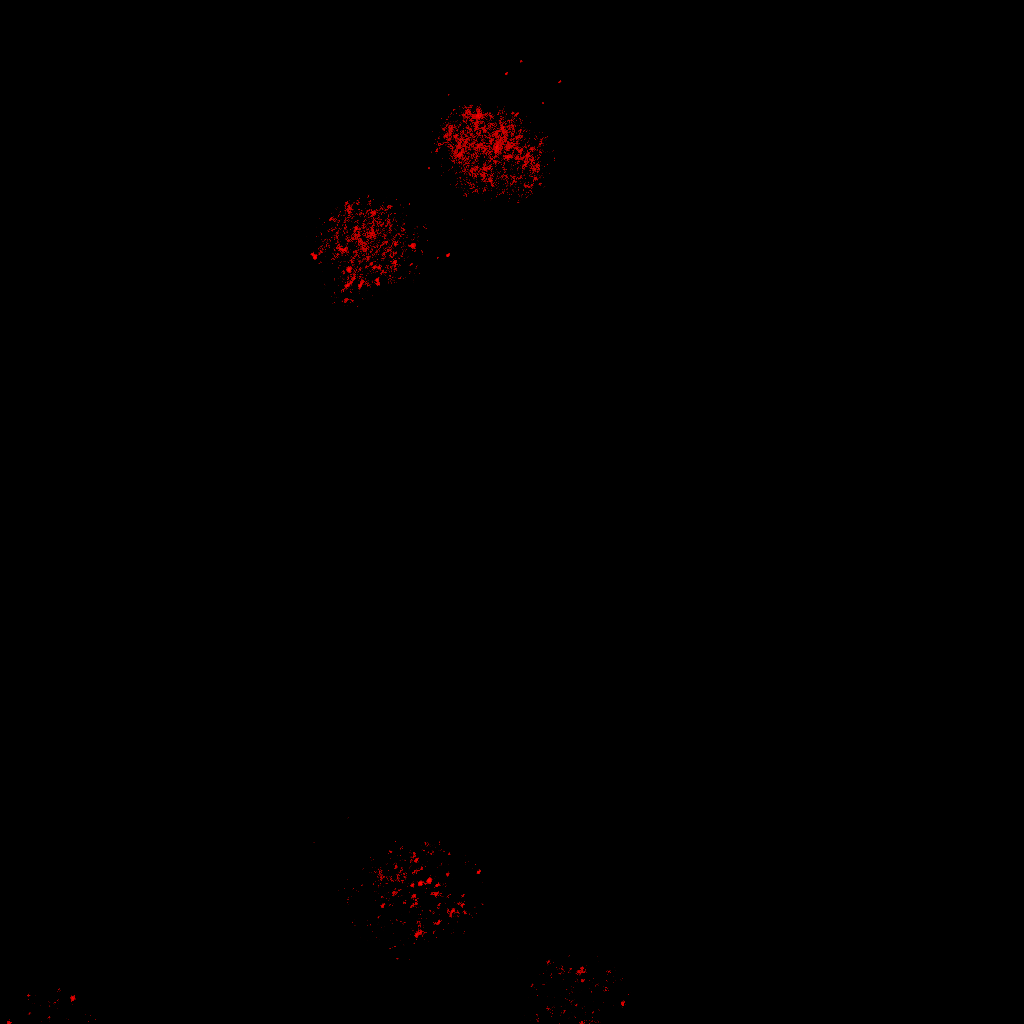

Supplement: Supplementary file 9 — Expanded View Figure and Appendix source data [file 44319_2025_513_MOESM9_ESM.zip › Expanded View Figure and Appendix source data/Expanded View Figure 5/EV 5D/sgNC 8 h/RAD51.tif]

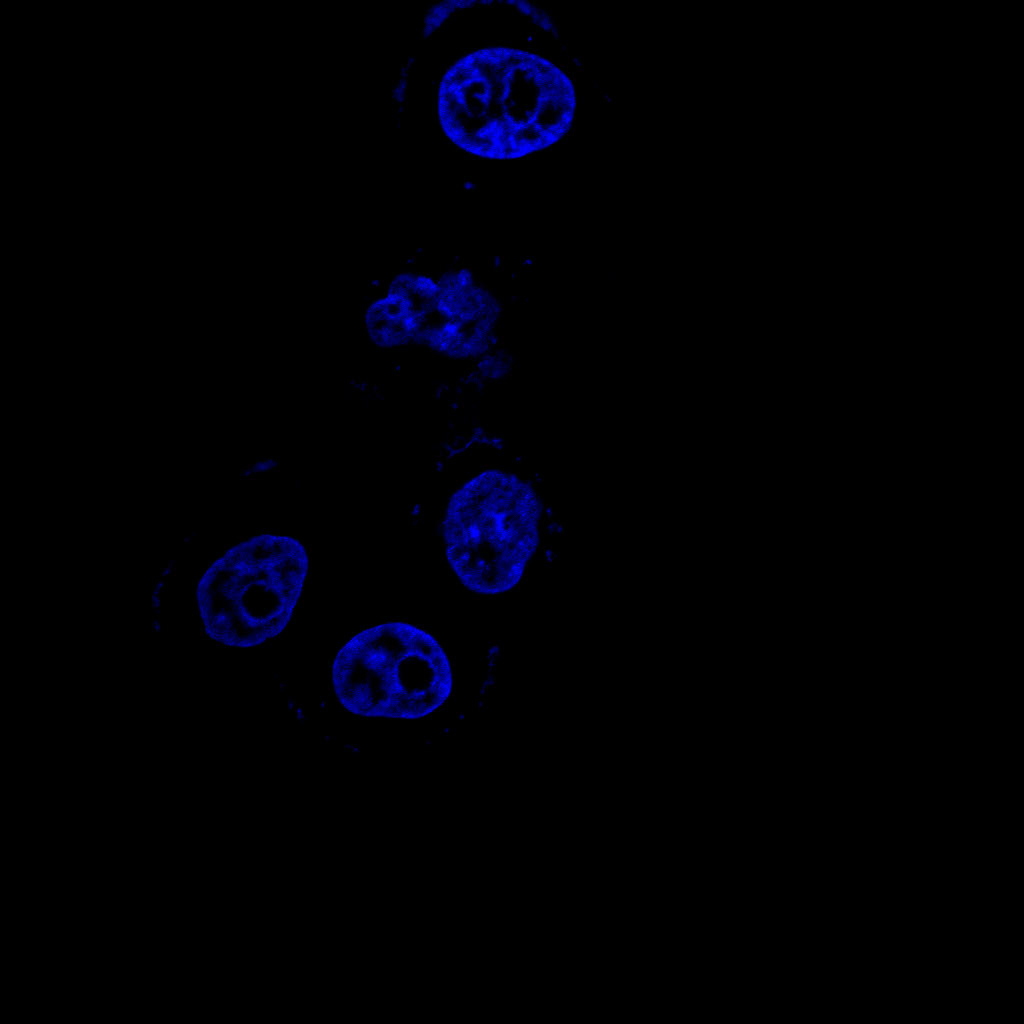

Supplement: Supplementary file 9 — Expanded View Figure and Appendix source data [file 44319_2025_513_MOESM9_ESM.zip › Expanded View Figure and Appendix source data/Expanded View Figure 5/EV 5D/sgNC ETO-/DAPI.tif]

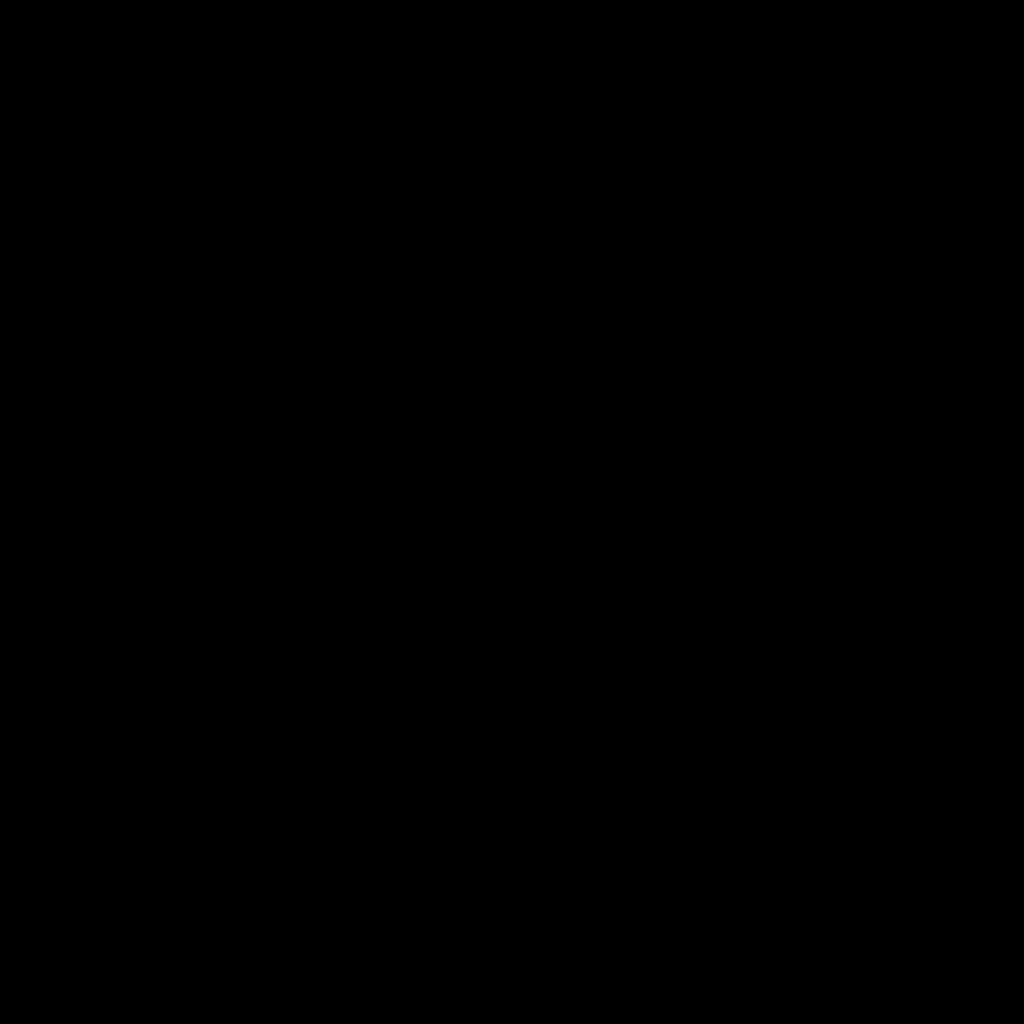

Supplement: Supplementary file 9 — Expanded View Figure and Appendix source data [file 44319_2025_513_MOESM9_ESM.zip › Expanded View Figure and Appendix source data/Expanded View Figure 5/EV 5D/sgNC ETO-/GH2AX.tif]

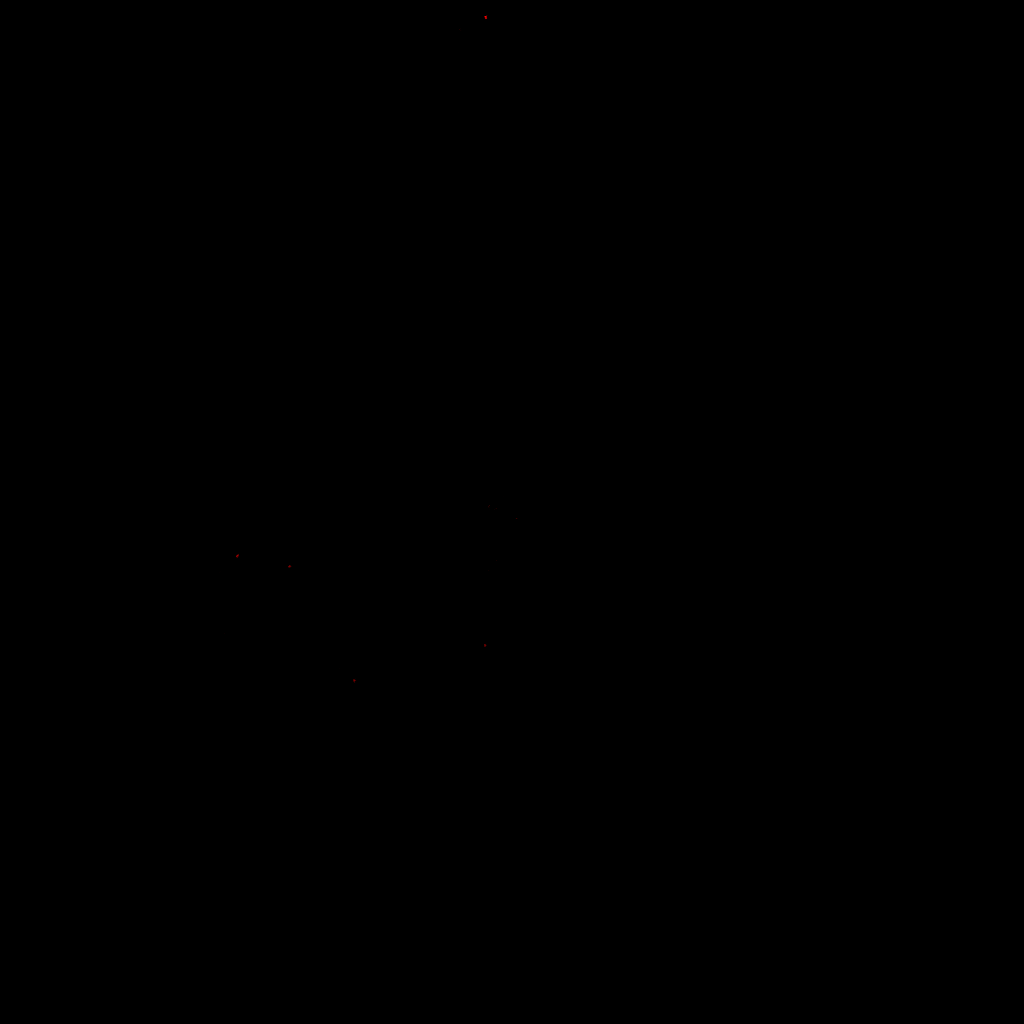

Supplement: Supplementary file 9 — Expanded View Figure and Appendix source data [file 44319_2025_513_MOESM9_ESM.zip › Expanded View Figure and Appendix source data/Expanded View Figure 5/EV 5D/sgNC ETO-/RAD51.tif]
