# Supplementary material for: PCAF-mediated acetylation regulates RAD51 dynamic localization on chromatin during HR repair
Source: EMBO Rep. 2025 Jul 15;26(16):4100–23. doi: 10.1038/s44319-025-00513-6 (PMC12373954; doi:10.1038/s44319-025-00513-6)
Supplement: Supplementary file 10 — Expanded View Figures [file 44319_2025_513_MOESM10_ESM.pdf]

Expanded View Figures

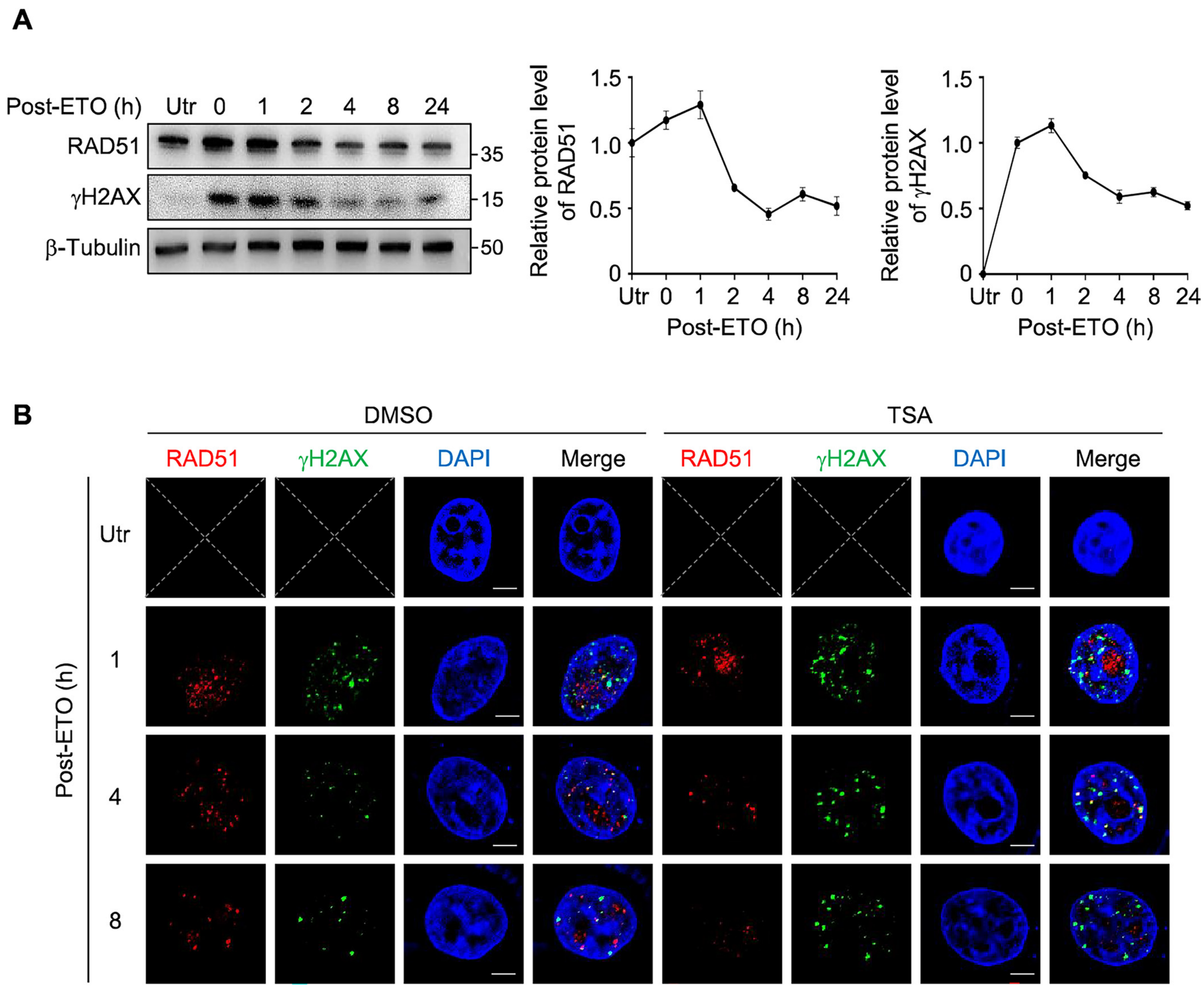

Figure EV1. RAD51 protein levels change dynamically during HR.

(**A**) Immunoblot of RAD51 and γH2AX in HeLa cells untreated or treated with 20 μM ETO for 2 h and recovered at the indicated time points (left). Quantifications of RAD51 and γH2AX are shown on the right. Data represented as mean ± SD of three independent experiments. (**B**) Representative immunofluorescence images of RAD51 (red) and γH2AX (green) foci in HeLa cells pretreated with DMSO and TSA (0.5 μM, 6 h), followed by ETO exposure (20 μM, 2 h), with cell lysates recovered at the indicated time points. DNA was stained by DAPI (blue). Scale bars, 10 μm. X indicated that with the chosen microscopy settings, no signal was obtained. Source data are available online for this figure.

 © The Author(s)

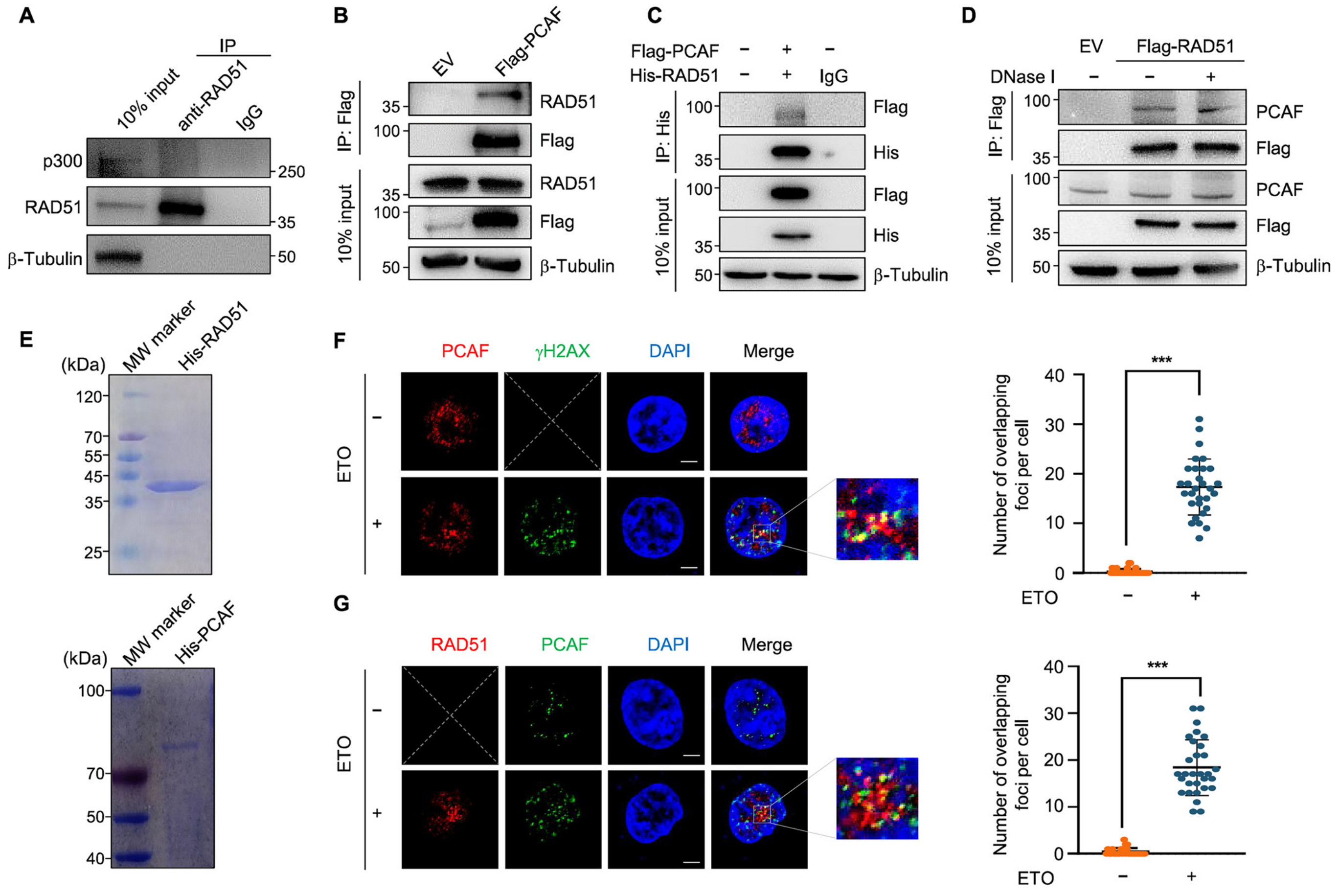

Figure EV2. PCAF interacts with RAD51.

(**A**) Immunoprecipitation to detect the interaction between RAD51 and p300 in HEK293T cells. (**B**) Immunoblot of RAD51 in anti-Flag immunoprecipitates from HEK293T cells transfected with empty vector or Flag-PCAF. (**C**) Immunoblot of Flag-PCAF in anti-His immunoprecipitates from HEK293T cells co-transfected with Flag-PCAF and His-RAD51 or empty vector. (**D**) Immunoblot of PCAF in anti-Flag-RAD51 immunoprecipitates from HEK293T cells transfected with empty vector or Flag-RAD51 and treated with or without DNase I. (**E**) Coomassie blue staining of purified His-RAD51 (upper) and His-PCAF (lower) protein. (**F**) Representative immunofluorescence images and quantification ($n = 30$) of PCAF (red) and γH2AX (green) foci in HeLa cells treated with or without ETO (20 μM, 2 h) and recovered for 1 h. Scale bars, 10 μm. X indicated that with the chosen microscopy settings, no signal was obtained. $P < 0.0001$. (**G**) Representative immunofluorescence images and quantification ($n = 30$) of RAD51 (red) and PCAF (green) foci in HeLa cells treated with or without ETO (20 μM, 2 h) and recovered for 1 h. Scale bars, 10 μm. X indicated that with the chosen microscopy settings, no signal was obtained. $P < 0.0001$. All data are represented as mean ± SD of three independent experiments. P values are from Mann–Whitney U test (**F**, **G**). ***$P < 0.001$. Source data are available online for this figure.

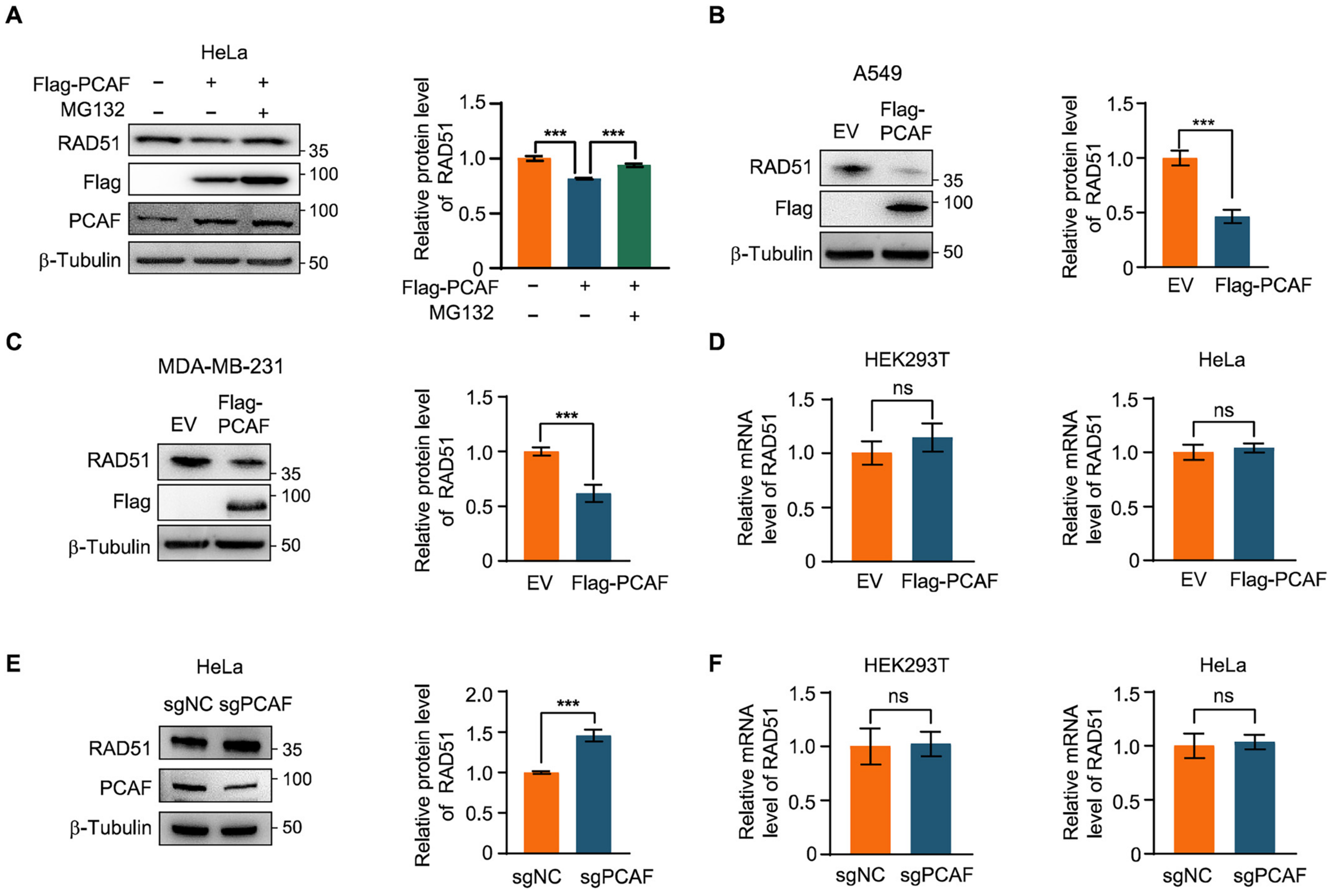

Figure EV3. PCAF does not affect the transcription of RAD51.

(**A**) Immunoblot (left) and quantification (right) of RAD51 in HeLa cells transfected with or without Flag-PCAF first, then treated with or without 10 μM MG132 for 6 h. EV vs Flag-PCAF ($P = 0.0002$), Flag-PCAF vs Flag-PCAF + MG132 ($P = 0.0002$). (**B**, **C**) Immunoblot (left) and quantification (right) of RAD51 in A549 (**B**) and MDA-MB-231 (**C**) cells transfected with or without Flag-PCAF. (**B**) ($P = 0.0006$), (**C**) ($P = 0.00097$). (**D**) Relative mRNA level of RAD51 in HEK293T (left) and HeLa (right) cells transfected with empty vector or Flag-PCAF for 48 h. HEK293T ($P = 0.7592$), HeLa ($P = 0.5225$). (**E**) Immunoblot (left) and quantification (right) of RAD51 in HeLa cells transfected with sgNC or sgPCAF. $P = 0.0004$. (**F**) Relative mRNA level of RAD51 in HEK293T (left) and HeLa (right) cells transfected with sgNC or sgPCAF. HEK293T ($P = 0.6320$), HeLa ($P = 0.5095$). All data are represented as mean ± SD of three independent experiments. *P* values are from Student's *t* tests. $^{***}P < 0.001$; ns not significant (**A**–**F**).

 © The Author(s)

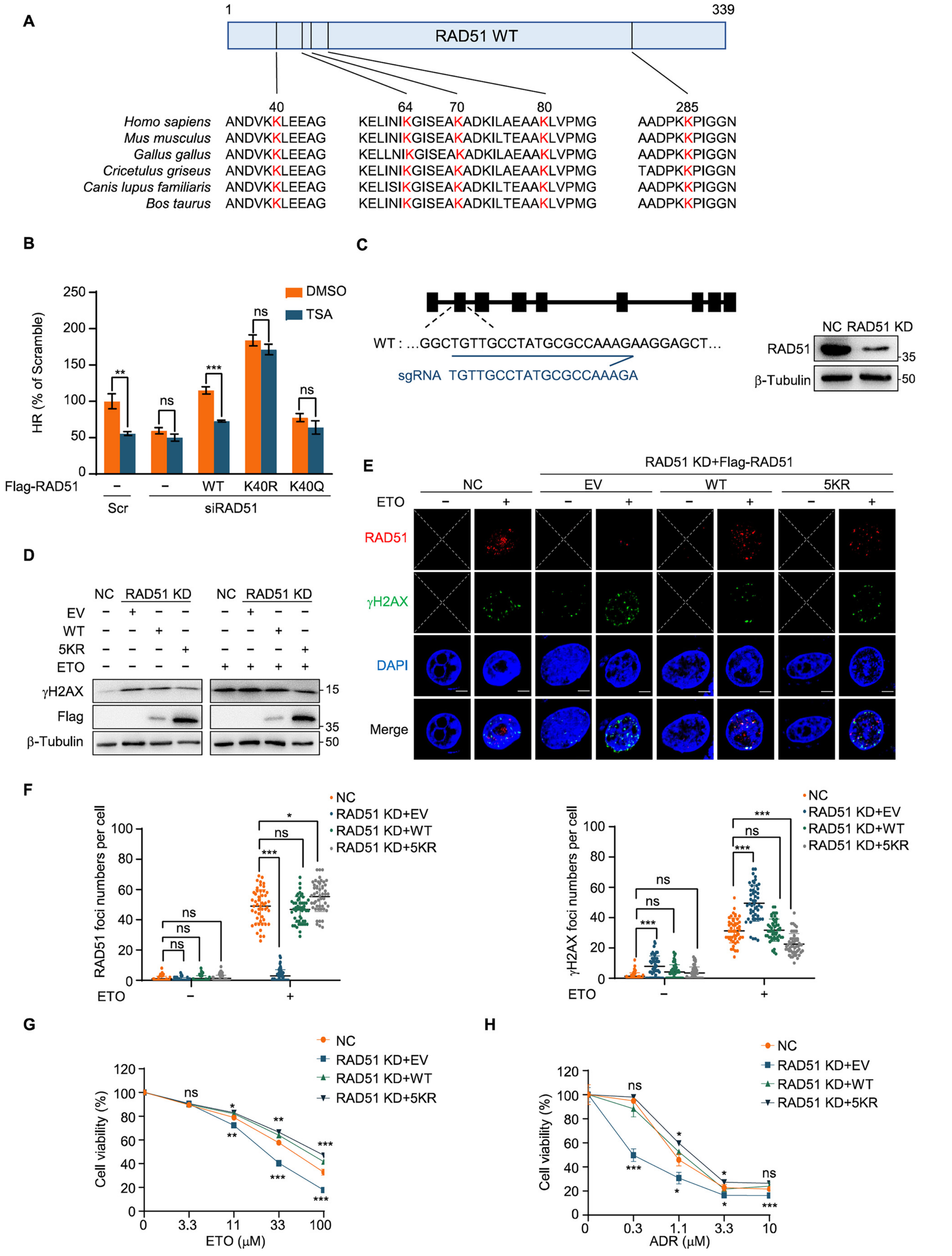
A
1
339
RAD51 WT
40
64
70
80
285
Homo sapiens ANDVKKLEEAG KELINIKGISEAKADKILAEAAKLVPMG AADPKKPIGGN
Mus musculus ANDVKKLEEAG KELINIKGISEAKADKILTEAAKLVPMG AADPKKPIGGN
Gallus gallus ANDVKKLEEAG KELLNIKGISEAKADKILAEAAKLVPMG AADPKKPIGGN
Cricetulus griseus ANDVKKLEEAG KELINIKGISEAKADKILAEAAKLVPMG TADPKKPIGGN
Canis lupus familiaris ANDVKKLEEAG KELISIKGISEAKADKILTEAAKLVPMG AADPKKPIGGN
Bos taurus ANDVKKLEEAG KELINIKGISEAKADKILTEAAKLVPMG AADPKKPIGGN
B
HR (% of Scramble)
DMSO
TSA
Flag-RAD51
Scr
siRAD51
WT
K40R
K40Q
C
WT : ...GGCTGTTGCCTATGCGCCAAAGAAGGAGCT...
sgRNA TGTTGCCTATGCGCCAAAGA
NC RAD51 KD
RAD51
β-Tubulin
D
NC RAD51 KD NC RAD51 KD
EV
WT
5KR
ETO
γH2AX
Flag
β-Tubulin
E
RAD51 KD+Flag-RAD51
NC
EV
WT
5KR
ETO
RAD51
γH2AX
DAPI
Merge
F
NC
RAD51 KD+EV
RAD51 KD+WT
RAD51 KD+5KR
RAD51 foci numbers per cell
γH2AX foci numbers per cell
ETO
G
NC
RAD51 KD+EV
RAD51 KD+WT
RAD51 KD+5KR
Cell viability (%)
ETO (μM)
H
NC
RAD51 KD+EV
RAD51 KD+WT
RAD51 KD+5KR
Cell viability (%)
ADR (μM)

Figure EV4. RAD51 acetylation regulates HR.

(**A**) Diagram showing the sequence of acetylation sites of RAD51. (**B**) HR efficiency in U2OS cells transfected with scramble RNA or siRAD51 for 24 h, followed by transfection with the indicated Flag-RAD51 mutations for another 24 h, and treated with DMSO and TSA (0.5 μM, 6 h). Scr ($P = 0.001898$), siRAD51 ($P = 0.066415$), WT ($P = 0.000142$), K40R ($P = 0.103938$), K40Q ($P = 0.096139$). (**C**) Schematic diagram of CRISPR/Cas9 targeting RAD51 (left) and immunoblot (right) of RAD51 in RAD51 KD HeLa cells. (**D**) Immunoblot of γH2AX in RAD51 KD HeLa cells transfected with indicated Flag-RAD51 mutations and treated with or without ETO (20 μM, 2 h). (**E**) Representative immunofluorescence images of RAD51 (red) and γH2AX (green) foci in RAD51 KD HeLa cells transfected with different Flag-RAD51 mutations and treated with or without ETO (20 μM, 2 h). DNA was stained by DAPI (blue). Scale bars, 10 μm. X indicated that with the chosen microscopy settings, no signal was obtained. (**F**) Quantification of RAD51 (right) or γH2AX (left) foci per cell from (**E**) ($n = 50$). RAD51, ETO − , NC vs RAD51 KD + EV ($P = 0.1333$), NC vs RAD51 KD + WT ($P = 0.7142$), NC vs RAD51 KD + 5KR ($P = 0.4249$). RAD51, ETO + , NC vs RAD51 KD + EV ($P < 0.0001$), NC vs RAD51 KD + WT ($P = 0.2425$), NC vs RAD51 KD + 5KR ($P = 0.0013$). γH2AX, ETO − , NC vs RAD51 KD + EV ($P < 0.0001$), NC vs RAD51 KD + WT ($P = 0.3234$), NC vs RAD51 KD + 5KR ($P = 0.165271$). γH2AX, ETO + , NC vs RAD51 KD + EV ($P < 0.0001$), NC vs RAD51 KD + WT ($P = 0.8152$), NC vs RAD51 KD + 5KR ($P < 0.0001$). (**G**, **H**) Cell survival assay was performed in RAD51 KD HeLa cells transfected with indicated Flag-RAD51 mutations in response to different doses of ETO (**G**) and ADR (**H**). ETO, NC vs RAD51 KD + 5KR, 3.3 μM ($P = 0.237876$), 11 μM ($P = 0.021469$), 33 μM ($P = 0.001285$), 100 μM ($P = 0.00063$). NC vs RAD51 KD + EV, 3.3 μM ($P = 0.58183$), 11 μM ($P = 0.005927$), 33 μM ($P = 0.00068$), 100 μM ($P = 0.000489$). ADR, NC vs RAD51 KD + 5KR, 0.3 μM ($P = 0.020424$), 1.1 μM ($P = 0.008277$), 3.3 μM ($P = 0.060957$), 10 μM ($P = 0.002279$). NC vs RAD51 KD + EV, 0.3 μM ($P = 0.000134$), 1.1 μM ($P = 0.019614$), 3.3 μM ($P = 0.016448$), 10 μM ($P = 0.000114$). All data are represented as mean ± SD of three independent experiments. P values are from Mann–Whitney U test (**F**) or Student's t tests (**B**, **G**, **H**). *$P < 0.05$, **$P < 0.01$, ***$P < 0.001$, ns not significant. Source data are available online for this figure.

 © The Author(s)

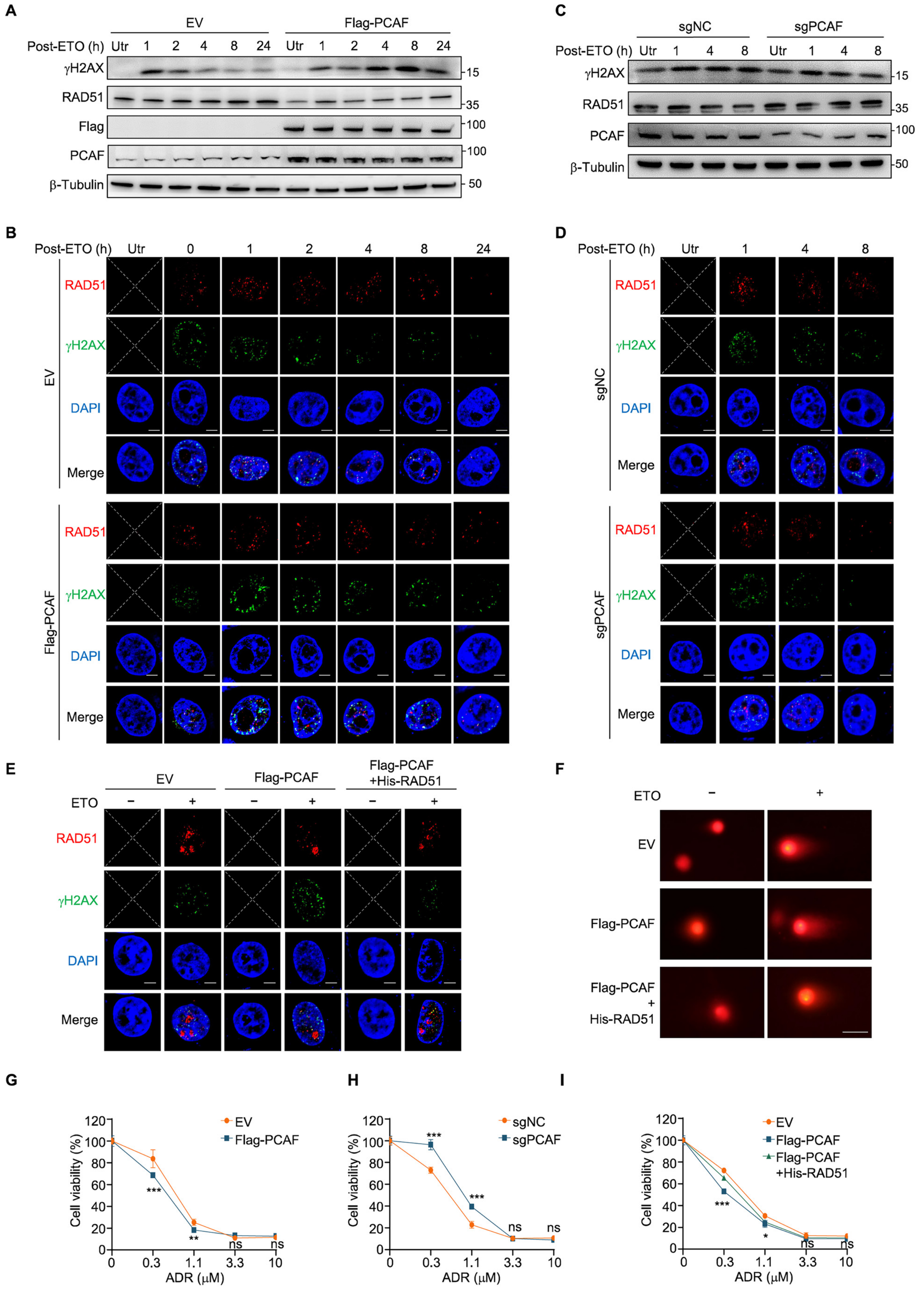

A
EV
Flag-PCAF
Post-ETO (h) Utr 1 2 4 8 24 Utr 1 2 4 8 24
γH2AX
RAD51
Flag
PCAF
β-Tubulin
15
35
100
100
50
B
Post-ETO (h) Utr 0 1 2 4 8 24
EV
Flag-PCAF
RAD51
γH2AX
DAPI
Merge
C
sgNC
sgPCAF
Post-ETO (h) Utr 1 4 8 Utr 1 4 8
γH2AX
RAD51
PCAF
β-Tubulin
15
35
100
50
D
Post-ETO (h) Utr 1 4 8
sgNC
sgPCAF
RAD51
γH2AX
DAPI
Merge
E
EV
Flag-PCAF
Flag-PCAF +His-RAD51
ETO − + − + − +
RAD51
γH2AX
DAPI
Merge
F
ETO − +
EV
Flag-PCAF
Flag-PCAF + His-RAD51
G
EV
Flag-PCAF
Cell viability (%)
ADR (μM)
0 0.3 1.1 3.3 10
***
**
ns
ns
H
sgNC
sgPCAF
Cell viability (%)
ADR (μM)
***
***
ns
ns
I
EV
Flag-PCAF
Flag-PCAF +His-RAD51
Cell viability (%)
ADR (μM)
***
*
ns
ns

Figure EV5. PCAF is involved in HR.

(**A**) Immunoblot of γH2AX and RAD51 in HeLa cells transfected with empty vector or Flag-PCAF, treated with 20 μM ETO for 2 h and recovered at the indicated time points. (**B**) Representative immunofluorescence images of RAD51 (red) and γH2AX (green) foci in HeLa cells transfected with empty vector or Flag-PCAF, followed by ETO exposure (20 μM, 2 h), with cell lysates recovered at the indicated time points. DNA was stained by DAPI (blue). Scale bars, 10 μm. X indicated that with the chosen microscopy settings, no signal was obtained. (**C**) Immunoblot of γH2AX and RAD51 in HeLa cells transfected with sgNC or sgPCAF, treated with 20 μM ETO for 2 h and recovered at the indicated time points. (**D**) Representative immunofluorescence images of RAD51 (red) and γH2AX (green) foci in HeLa cells transfected with sgNC or sgPCAF, followed by ETO exposure (20 μM, 2 h), with cell lysates recovered at the indicated time points. DNA was stained by DAPI (blue). Scale bars, 10 μm. X indicated that with the chosen microscopy settings, no signal was obtained. (**E**) Representative immunofluorescence images of RAD51 (red) and γH2AX (green) foci in HeLa cells transfected with Flag-PCAF or Flag-PCAF and His-RAD51, followed by ETO exposure (20 μM, 2 h), with cell lysates recovered at the indicated time points. DNA was stained by DAPI (blue). Scale bars, 10 μm. X indicated that with the chosen microscopy settings, no signal was obtained. (**F**) Neutral comet assay in HeLa cells overexpressing PCAF or co-transfected with Flag-PCAF and His-RAD51, with or without ETO treatment (20 μM, 2 h). Scale bars, 100 μm. X indicated that with the chosen microscopy settings, no signal was obtained. (**G**) Cell survival assay was performed in HeLa cells transfected with empty vector or Flag-PCAF in response to different doses of ADR. 0.3 μM ($P < 0.0001$), 1.1 μM ($P = 0.00228$), 3.3 μM ($P = 0.287791$), 10 μM ($P = 0.522845$). (**H**) Cell survival assay was performed in HeLa cells transfected with sgNC or sgPCAF in response to different doses of ADR. 0.3 μM ($P = 0.000153$), 1.1 μM ($P = 0.000848$), 3.3 μM ($P = 0.371428$), 10 μM ($P = 0.24121$). (**I**) Cell survival assay was performed in HeLa cells transfected with PCAF or co-transfected with Flag-PCAF and His-RAD51 in response to different doses of ADR. EV vs Flag-PCAF, 0.3 μM ($P = 0.000107$), 1.1 μM ($P = 0.029975$), 3.3 μM ($P = 0.192603$), 10 μM ($P = 0.166067$). All data are represented as mean ± SD of three independent experiments. *P* values are from Student's *t* tests (**G–I**). $^{**}P < 0.01$, $^{***}P < 0.001$, ns: not significant. Source data are available online for this figure.

 © The Author(s)
